# Estimating Orientation of Flying Fruit Flies

**DOI:** 10.1371/journal.pone.0132101

**Published:** 2015-07-14

**Authors:** Xi En Cheng, Shuo Hong Wang, Zhi-Ming Qian, Yan Qiu Chen

**Affiliations:** 1 School of Computer Science, Shanghai Key Laboratory of Intelligent Information Processing, Fudan University, Shanghai, China; 2 Jingdezhen Ceramic Institute, Jingdezhen, China; Universitaet Regensburg, GERMANY

## Abstract

The recently growing interest in studying flight behaviours of fruit flies, *Drosophila melanogaster*, has highlighted the need for developing tools that acquire quantitative motion data. Despite recent advance of video tracking systems, acquiring a flying fly’s orientation remains a challenge for these tools. In this paper, we present a novel method for estimating individual flying fly’s orientation using image cues. Thanks to the line reconstruction algorithm in computer vision field, this work can thereby focus on the practical detail of implementation and evaluation of the orientation estimation algorithm. The orientation estimation algorithm can be incorporated into tracking algorithms. We rigorously evaluated the effectiveness and accuracy of the proposed algorithm by running experiments both on simulation data and on real-world data. This work complements methods for studying the fruit fly’s flight behaviours in a three-dimensional environment.

## Introduction

The recently increasing interest in studying flight behaviours of fruit flies, *Drosophila melanogaster*, has heightened the need for developing tools that obtain quantitative motion data [1]. Previous studies have made contributions on obtaining trajectories of individuals using images from multiple cameras, such as fruit flies [2–9] and midges [10, 11]. Due to large number of individuals, insect swarms usually spread across wide space. When imaged, each target only takes up tens of pixels in the images since we have to film the entire swarms in the cameras’ field-of-view (FOV). That is, such images can only provide limited visual cues (or features) for tracking algorithms to estimate motion data. On one hand, the state-of-the-art methods [5–11] obtained the successive locations of each target, but neglected the orientation of targets. On the other hand, biological studies have demonstrated that the orientation is a key source of information on studying behaviours of fruit flies [12–17].

Due to lack of data, previous studies [18–20] usually represented a fruit fly’s orientation by its motion direction. However, a fly’s orientation is not the same as its motion direction, such as the elevation of a fly’s orientation tends to be 45° from horizontal [21]. Further, a fly’s orientation may be entirely different to the motion direction when it takes maneuvers [12, 13, 22, 23], such as saccades.

In this paper, we present a novel method for estimating individual fruit fly’s orientation when many flies are flying in a laboratory arena. Thanks to the line reconstruction algorithm in computer vision field [24], this work can thereby focus on the practical detail of implementation and evaluation of the orientation estimation algorithm. The orientation estimation algorithm can be incorporated into tracking algorithms. We rigorously evaluated the effectiveness and accuracy of the algorithm by running experiments both on simulation data and on real-world data. The presented method powerfully complements methods for studying flight behaviour in a three-dimensional (3D) environment. The source code is provided as the supporting information (S1 File).

## Methods

In order to obtain 3D motion data, multiple synchronized and geometrically calibrated cameras are adopted. Although in principle, two cameras are sufficient for stereo imaging. Three or more cameras are typically required to resolve the ambiguities between targets and to avoid false identifications. Further, because of the perspective effect, three or more cameras are required for estimating a fruit fly’s orientation accurately.

### 2.1 Orientation estimation problem

#### 2.1.1 Modeling fruit flies

**Fig 1:**
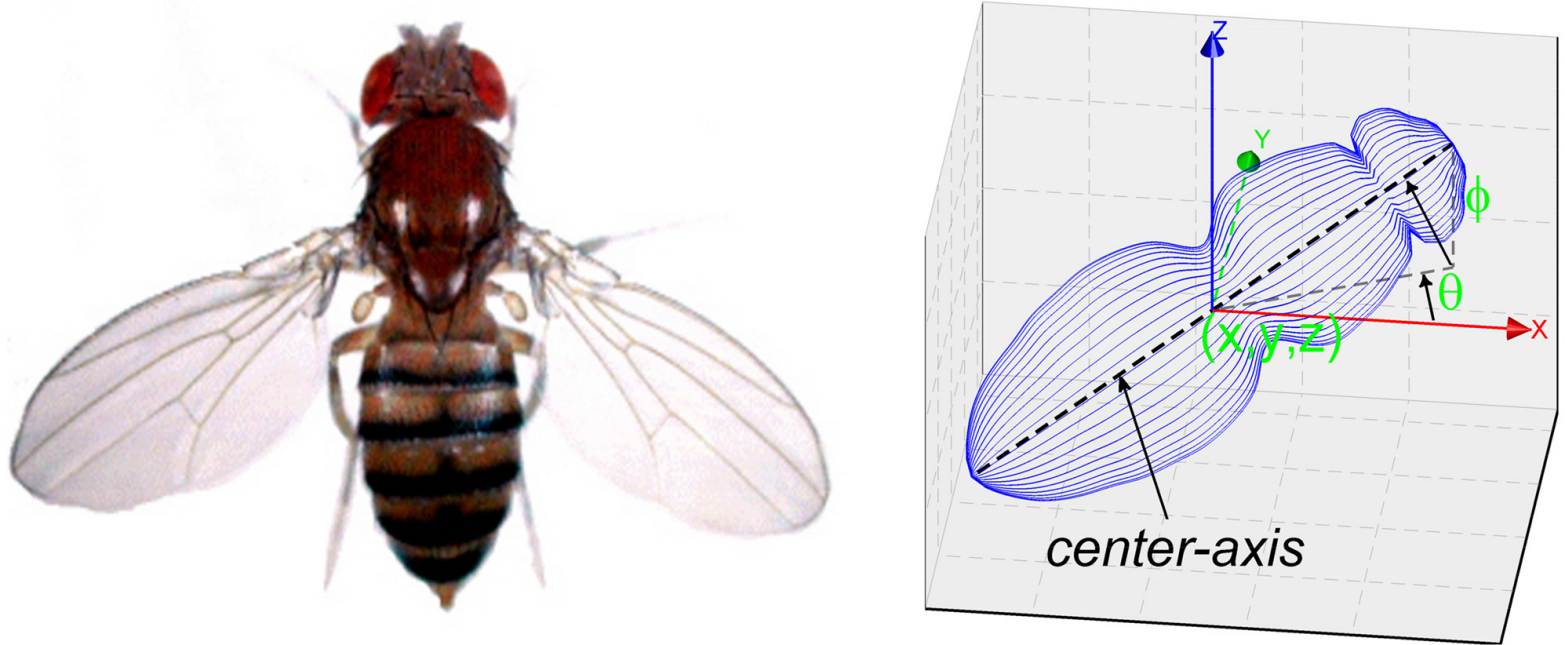
Orientation of a fruit fly. (a) The front image of an adult fruit fly. (b) The fruit fly locates at (*x*, *y*, *z*). Its orientation is defined by two angles (*θ*, *ϕ*) against the coordinate system. *θ* is an angle from x-axis and *ϕ* is an angle from horizontal (the x-y plane). Concatenating the location (*x*, *y*, *z*) and orientation (*θ*, *ϕ*), it defines a directed line-segment in 3D space, the *center-axis* of the target.

Given the location of a fruit fly *X* = (*x*, *y*, *z*)^T^, the fly’s orientation is defined by two angles *O* = (*θ*, *ϕ*)^T^ against the world coordinate system (see Fig 1b), where *θ* ∈ [−180°,180°], *ϕ* ∈ [−90°,90°]. These two angles are known as azimuth and elevation. Concatenating the location (*x*, *y*, *z*) and the orientation (*θ*, *ϕ*), the tuple (*x*, *y*, *z*, *θ*, *ϕ*) defines a directed line-segment in 3D, the center-axis of the fly’s body. On the other hand, it is well known that the flight attitude of a flight object is defined by three angles (yaw, pitch, and roll). The angles azimuth and elevation correspond to yaw and pitch, respectively. Here we ignore the roll angle since we do not have sufficient visual cues for estimating the roll angle. Previous studies [25] have demonstrated that the roll angle can be determined by using the symmetrical position of wings, if there are ample visual cues.

#### 2.1.2 Line reconstruction algorithm

According to the aforementioned model of fruit flies, a fly’s motion state at a certain moment can be defined by (*x*, *y*, *z*, *θ*, *ϕ*) which includes both the fly’s location and its orientation. Since we ignored the roll angle of a fruit fly’s body, a fly’s orientation is defined by a line-segment in 3D (given the center-axis of a fruit fly, we have developed the generative shape model to represent its body, see S2 Fig). In computer vision field, there is a line reconstruction algorithm for multi-camera systems [24]. Suppose a line *l*_3_ in 3D space is projected to lines in two views as *l*_2_ and $l_{2}^{\prime }$. The line *l*_3_ can be reconstructed by back-projecting each image line (*l*_2_ and $l_{2}^{\prime }$) to give a plane in 3D space, and intersecting the planes:

$$\begin{array}{c} l_{3}=[\begin{array}{c} P^{T}l_{2}\\ P^{{}^{\prime }T}l_{2}^{\prime } \end{array}] \end{array}$$ (1)

where **P** and **P**′ denotes the projection matrix of two cameras; and the planes defined by the back-projecting of image lines are **P** ^**T**^ *l*_2_ and ${\text{P}}^{\prime \text{T}}l_{2}^{\prime }$. This equation can be theoretically generalized into more cameras. But it needs to evaluate the Maximum Likelihood to estimate the solution while the equation is generalized for three or more cameras [24].

In order to recover a fly’s orientation, which is defined by its center-axis (a line-segment), we have to detect image lines in camera views, in which each image line is the projection of the center-axis. According to the line reconstruction algorithm and considering the efficiency, using two image lines in two camera views is preferable.

### 2.2 Body detection

**Fig 2:**
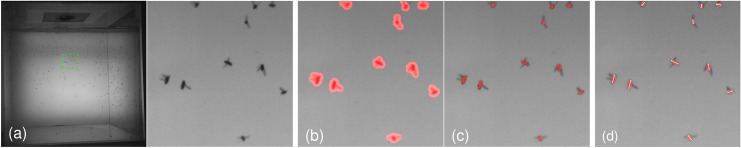
Body detection. (a) The image at the left was filmed by a back-lit camera and the patch marked by the green rectangle was zoomed at the right. (b) The patch was overlaid with red blobs which were detected using background subtraction. (c) The patch ass overlaid with refined blobs in which the pixels of wings were removed. (d) Each refined blob is fitted by an ellipse.

Previous studies [8, 10] have demonstrated that the illumination was usually provided by front-lighting. The front-lighting means lights and cameras are placed at the same side of the targets. It has the advantage of showing the rich texture of targets and background. The rich texture however makes target detection and separating pixels corresponding to a target’s body very difficult. The back-lighting is the opposite, i.e. the targets are imaged as silhouettes against plain white background. In our problem, we moved the lights to the side of targets opposite the cameras, meaning that targets were back-lit in camera views (see S1 Fig, which shows the experimental system). Fig 2a shows an image in which fruit flies were back-lit in a camera view. We present a simple and efficient detecting approach in this paper. It makes full use of the different levels of transparency between the wings and body of a fruit fly, in which the pixels of wings have higher intensities than pixels of body. The detecting approach segments an image into blobs. Each blob is an image area with pixels’ locations and intensities. To simplify notation, all definitions in this section ignore the subscripts for cameras and moments.

#### 2.2.1 Segmenting blobs

In the first step, we subtract a Gaussian background model from an acquired image. Given a pixel *i* which denotes the location of a certain pixel in a certain camera view, let *μ*(*i*) denote its mean intensity and *σ*(*i*) denote its standard deviation of intensities through time. We compute *μ*(*i*) and *σ*(*i*) according to a sequence of consecutively acquired images in the camera view. That is, *μ* and *σ* define the Gaussian background model in the camera view.

The pixel *i* in a certain image is segmented as a foreground pixel if its intensity satisfies the constraint:

$$\begin{array}{c} \frac{I(i)-\mu (i)}{\sigma (i)}<-C_{1} \end{array}$$ (2)

where *I* denotes the acquired image at a certain moment. Connected foreground pixels are clustered as blobs *b* ∈ {1, …, *b*_*n*_}. We chose the constant value *C*_1_ = 1.5 which guarantees that each blob including pixels of a fly’s wings and body. Fig 2b shows the segmented blobs of the image patch.

#### 2.2.2 Removing wings pixels

In the second step, we use local Gaussian models to remove the pixels of wings from blobs. Given a blob *b*, let *μ*_*b*_ denote its mean intensity and *σ*_*b*_ denote its standard deviation of intensities. These variables, *μ*_*b*_ and *σ*_*b*_, are computed using the pixels in the blob *b*. The local Gaussian model of the blob *b* is thereby defined by *μ*_*b*_ and *σ*_*b*_. This second step is a refinement step and is computed as

$$\begin{array}{c} blob\left(b,i\right)=\{\begin{array}{cc} 1 & \frac{I\left(i\right)-\mu_{b}}{\sigma_{b}}<-C_{2}\\ 0 & otherwise \end{array} \end{array}$$ (3)

where *b* ∈ {1, …, *b*_*n*_} denotes the blobs and *i* ∈ {*i*_*b*,1_, …, *i*_*b*, *n*_} denotes the pixels of the blob *b*. If *blob*(*b*, *i*) is equals to 0, the pixel *i* is removed from the blob *b*. We choose the constant value *C*_2_ = 1.5 which guarantees most of pixels of a fly’s body being preserved after refinement. Fig 2c shows the image patch overlaid with the refined blobs. This step makes full use of the different levels of transparency between a fruit fly’s wings and its body (*i.e*. the pixels of wings have higher intensities than those of body). This is achieved thanks to the fruit flies were back-lit in camera views.

#### 2.2.3 Fitting blob with ellipse

In order to fit a blob with an ellipse accurately, it is usually that fitting a 2D Gaussian to the locations of all pixels in the blob. Given the parameters of the best-fitting Gaussian, the parameters of the ellipse can be computed. Instead of just computing the mean and covariance of all pixels in the blob, we compute a weighted mean and covariance. Let *w*(*i*) denote the weight of pixel *i*, the weight *w*(*i*) is defined as the normalized distance of the pixel *i*’s intensity to the background model defined by *μ* and *σ*:

$$\begin{array}{c} w\left(i\right)=\frac{\left|I(i)-\mu (i)\right|}{\sigma (i)} \end{array}$$ (4)

Therefore, the weighted mean and covariance of the pixels of a blob *b* are computed as:

$$\begin{array}{ccc} \mu_{b}^{\prime } & = & \frac{1}{W}\sum_{i}w\left(i\right)*i,\;i\in \left\{i_{b,1},...,i_{b,n}\right\}\\ \Sigma_{b}^{\prime } & = & \frac{1}{W}\sum_{i}w\left(i\right)*\left(i-\mu_{b}^{\prime }\right){\left(i-\mu_{b}^{\prime }\right)}^{T},\;i\in \left\{i_{b,1},...,i_{b,n}\right\} \end{array}$$ (5)

where *W* = ∑_*i*_ *w*(*i*), *i* ∈ {*i*_*b*,1_, …, *i*_*b*, *n*_} defines the weight’s normalization constant, and *i* ∈ {*i*_*b*,1_, …, *i*_*b*, *n*_} denotes the pixels of the blob *b*. The weighted mean and covariance not only improve the robustness on non-uniform illumination (*e.g*. a single threshold for all images), but also provide us with the sub-pixel accuracy on fitting ellipses. The mean $\mu_{b}^{\prime }$ defines the center of the ellipse, and the covariance $\Sigma_{b}^{\prime }$ defines the axis and the direction of the ellipse. Fig 2d shows the results of fitting each blob with an ellipse.

#### 2.2.4 Measurements definition

To simplify notation, we refer to a blob and the ellipse fitted to it as a ***measurement***. Let *χ* denote a ***measurement***, each measurement includes two components:

$$\begin{array}{c} \chi =\{b,elp(b)\} \end{array}$$ (6)

where *b* denotes the blob with both pixels’ locations and intensities, and *elp*(*b*) defines the ellipse fitted to the blob. We use 3 cameras in our experiments, and thereby the set of all available measurements at a certain moment was defined as $\left\{\chi_{i}^{v}\;|\;v\in \{1,2,3\},\;i\in \{1,...,i_{v,n}\}\right\}$.

### 2.3 Orientation estimation

**Fig 3:**
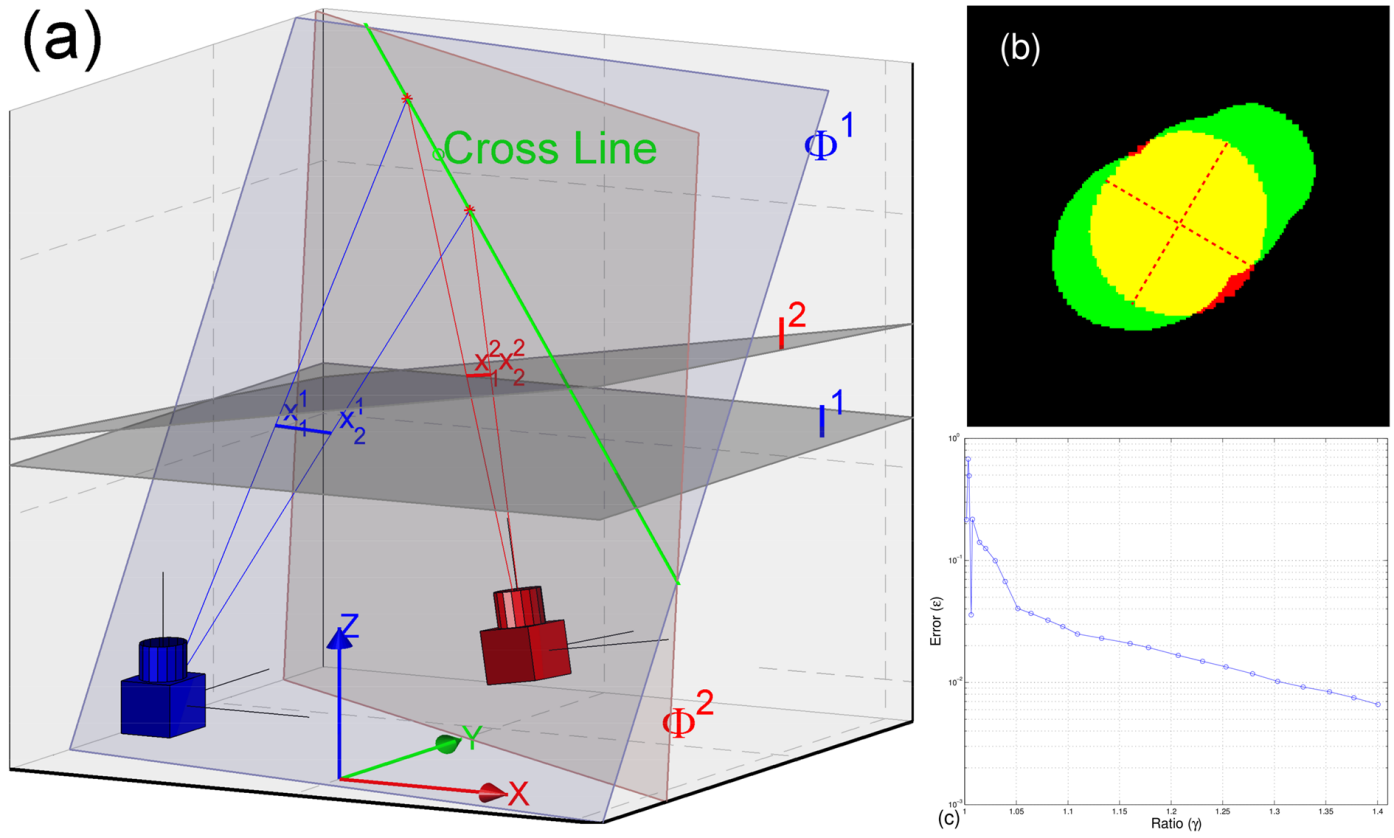
Orientation estimation. (a) The illustration of Algorithm 1. Here *I* ^1^ and *I* ^2^ denote the image plane of *Camera 1* (blue) and *Camera 2* (red) respectively. The line-segment $\left\langle x_{1}^{1},x_{2}^{1}\right\rangle$ (blue) denotes the ellipse’s major axis in *I* ^1^. And the line-segment $\left\langle x_{1}^{2},x_{2}^{2}\right\rangle$ (red) denotes the ellipse’s major axis in *I* ^2^. The projection rays of those end-points define two plane Φ^1^ and Φ^2^ respectively, *e.g*. two blue rays define Φ^1^. The orientation *O* is computed from the direction of the cross line (the longer green line) between two planes Φ^1^ and Φ^2^. Here all definitions are defined in the world coordinate system. (b) The orientation is problematic. The measurement is drawn in red, and red dashed lines are the major-axis and minor-axis of its ellipse. A generative shape according to the fly’s location *X* and orientation *O* is re-projected into the camera view (see S2 Fig for the generative shape model.). The green pixels are re-projected pixels. Yellow pixels are identical pixels. (c) The error *ϵ* as a function of the ratio *γ*. Here *γ* defines the length ratio between the major-axis and the minor-axis of an ellipse.

Given the measurement of a certain fruit fly in a certain camera view, measurements of the fly in other camera views can be matched according to several constraints, *e.g*. the epipolar constraint [24]. We name a pair of matched measurements across views as **m**atched **m**easurement **p**air (MMP for short). An MMP includes just one measurement in each camera view. The ellipses of the MMP are employed to estimate the fly’s orientation according to the pinhole camera model in Euclid geometry. According to Eq (1), it uses two lines as infinite, and does not use the endpoints [24]. In practice, we prefer to use the major-axis’s endpoints of the ellipses in an MMP. The algorithm for estimating orientation is presented as Algorithm 1. The interpretation is sketched in Fig 3a.

**Algorithm 1** The algorithm for estimating a fly’s orientation.

**Input**: The parameters of camera 1: *P* ^1^ = ((*K* ^1^)_3×3_, (*T* ^1^)_3×1_); The parameters of camera 2: *P* ^2^ = ((*K* ^2^)_3×3_, (*T* ^2^)_3×1_); The measurements from camera 1 and camera 2: *χ* ^1^(*b*, *elp*(*b*)) and *χ* ^2^(*b*, *elp*(*b*));

**Output**: The orientation (*θ*, *ϕ*)

1: Get end-points of the major-axis of ellipse *χ* ^1^(*elp*(*b*)): $x_{1}^{1}(i,j)$ and $x_{2}^{1}(i,j)$;

2: Compute the direction of the projection ray of each end-point:

3: ${\vec{d}}_{1}^{1}={\left(K^{1}\right)}^{-1}*{\left(x_{1}^{1}(i,j),1\right)}^{\text{T}}$ and ${\vec{d}}_{2}^{1}={\left(K^{1}\right)}^{-1}*{\left(x_{2}^{1}(i,j),1\right)}^{\text{T}}$;

4: Get end-points of the major-axis of ellipse *χ* ^2^(*elp*(*b*)): $x_{1}^{2}(i,j)$ and $x_{2}^{2}(i,j)$;

5: Compute the direction of the projection ray of each end-point:

6: ${\vec{d}}_{1}^{2}={\left(K^{2}\right)}^{-1}*{\left(x_{1}^{2}(i,j),1\right)}^{\text{T}}$ and ${\vec{d}}_{2}^{2}={\left(K^{2}\right)}^{-1}*{\left(x_{2}^{2}(i,j),1\right)}^{\text{T}}$;

7: Compute the normal of plane Φ^1^: ${\vec{n}}^{1}={\vec{d}}_{1}^{1}\times {\vec{d}}_{2}^{1}$;

8: Compute the normal of plane Φ^2^: ${\vec{n}}^{2}={\vec{d}}_{1}^{2}\times {\vec{d}}_{2}^{2}$;

9: Compute the direction of the cross line between Φ^1^ and Φ^2^: $\vec{d}={\vec{n}}^{1}\times {\vec{n}}^{2}$;

10: Compute the azimuth *θ* and the elevation *ϕ* of $\vec{d}$;

11: **if** *ϕ* < 0 **then**

12: $\vec{d}=-\vec{d}$;

13: Compute the azimuth *θ* and the elevation *ϕ* of $\vec{d}$;

14: **end if**

15: **return** (*θ*, *ϕ*)

#### 2.3.1 Determining the abdomen to head direction

Algorithm 1 presented the orientation estimation algorithm. The computing phase (step 1 to step 10) of Algorithm 1 can also be written as an equation, given the algorithm’s input (two ellipses from different camera views). At step 10 of Algorithm 1, a fly’s orientation is computed. However, the result may be an inverse direction to the real one, since we have no cues to determine the direction of abdomen to head. Fortunately, the characteristics of the flight patterns of fruit flies provide us with cues for determining the abdomen to head direction. In order to determine the direction of abdomen to head for a fruit fly’s orientation, we follow the rules:

1. **The elevation *ϕ* of a fruit fly’s orientation *O*(*θ*, *ϕ*) tends to be 45° from horizontal**.
2. **A fruit fly’s head usually points towards the sky**.

That is, the estimated orientation is an inverse direction to the real one if the elevation *ϕ* < 0 (see step 11 to step 14 of Algorithm 1, the rectifying phase). These rules are derived from previous studies [15, 21]. Previous studies have demonstrated the aerodynamics of flight maneuvers of fruit flies, in which the fruit fly tends to preserve the pitch angle of its flight attitude at ≈ 45°. Besides, the fruit fly probably had to head sky polarization for navigating itself [15].

#### 2.3.2 Choosing two ellipses

Fig 3a illustrates the proposed algorithm. It shows the fly’s orientation *O*(*θ*, *ϕ*) is computed from the direction of the cross line between two planes Φ^1^ and Φ^2^. However, the orientation may become problematic while measurements are close to circular. If the measurements of an MMP are close to circular, the major-axis of ellipses might have nothing to do with the plane in which the *center-axis* of a fruit fly’s body lies (as shown in Fig 3b). Let *γ* define the ratio between the major-axis and the minor-axis of an ellipse, *i.e*. *γ* measures the level of circular for a measurement. Let *α* define the angle between the estimated orientation and the ground truth, we compute the error *ϵ* = 1 − *cos*(*α*). Fig 3c shows the error *ϵ* as a function of ratio *γ*. It suggests that the error *ϵ* is less than 0.01 if *γ* satisfies *γ* ≥ 1.3. In our problem, we can easily choose two ellipses from an MMP (it includes 3 measurements from 3 camera views), which all satisfy *γ* ≥ 1.3. That is, the orientation *O* can be estimated accurately.

### 2.4 Incorporating into a tracking algorithm

We have developed a tracking algorithm based on the Bayesian inference framework (particle filtering [26]). The orientation estimation algorithm was incorporated into the tracking algorithm.

**Fig 4:**
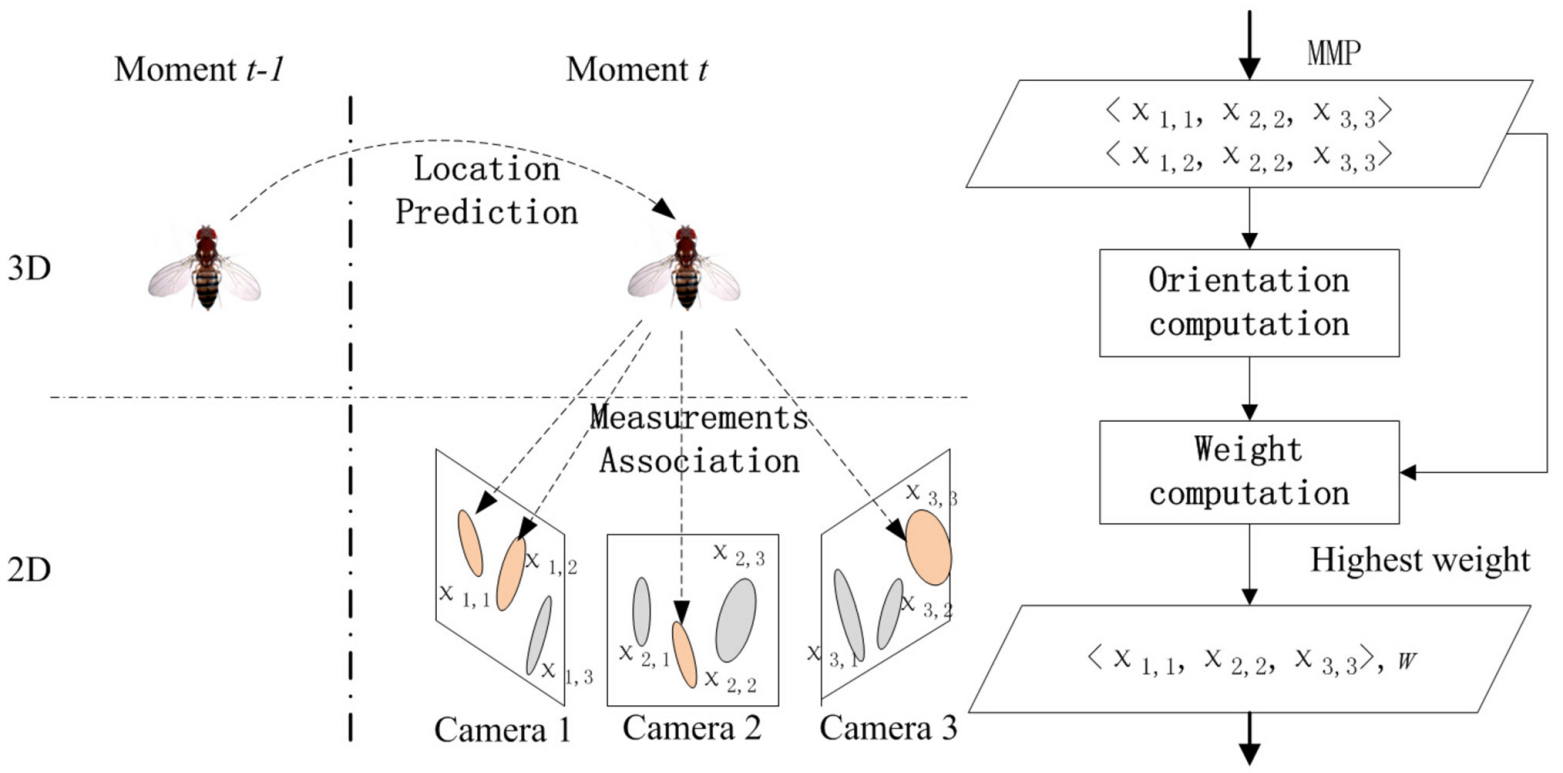
Overview of the tracking algorithm. The fruit fly is flying in 3D space. By exploiting the particle filtering solution, there are *N* (we set in our experiments *N* = 200) particles sampling the posterior distribution of the location of a target at moment *t*. Each particle includes the location’s prediction at moment *t*. The tracking algorithm computes the orientation and weight of each particle using the MMP associated with the particle at moment *t*. If there are more than one MMP (*e.g*. since the particle is associated with two measurements in *Camera 1*, the algorithm thereby generates two MMPs (*χ*_1,1_, *χ*_2,2_, *χ*_3,3_) and (*χ*_1,2_, *χ*_2,2_, *χ*_3,3_)), the algorithm choose the MMP having highest weight (*e.g*. (*χ*_1,1_, *χ*_2,2_, *χ*_3,3_)) and the orientation computed by the MMP.

The location of a target at moment *t* is predicted *N* times using the dynamic model and the target’s location at moment *t* − 1. Each prediction is included in a particle with another component, orientation. Each particle needs to be assigned at least one measurement in each camera view; and then the orientation component is computed using those associated measurements. Then, the probability of each particle is evaluated using the temporal consistency on appearance and orientation. The target’s motion state is the expectation of these *N* particles weighted by their probability. Therefore, the motion state of each target includes two components, location (*x*, *y*, *z*) and orientation (*θ*, *ϕ*). Fig 4 shows the overview of the tracking algorithm.

## Experiments and Results

The proposed method was implemented in the MATLAB™ environment. The source code was provided as supporting information (S1 File). We have done the performance evaluation both on simulation data and on real-world data.

### 3.1 Evaluation on simulation data

#### 3.1.1 Simulation data

The first step of simulation is to create the ground truth. Firstly, the 3D trajectory of each target could be generated by using a motion model, *e.g*. boids [27]. Secondly, given a certain target, we computed its motion direction at each moment, *M*(*θ*, *ϕ*). Lastly, the target’s orientation *O*(*θ*, *ϕ*) was computed at each moment as

$$\begin{array}{c} O=(\begin{array}{c} M(\theta )\\ \frac{\pi }{4} \end{array})+n,n∼N\left(0,\Sigma \right) \end{array}$$ (7)

where *n* denotes a small white noise. Targets’ trajectories, their motion direction, and their orientation formed the ground truth.

**Fig 5:**
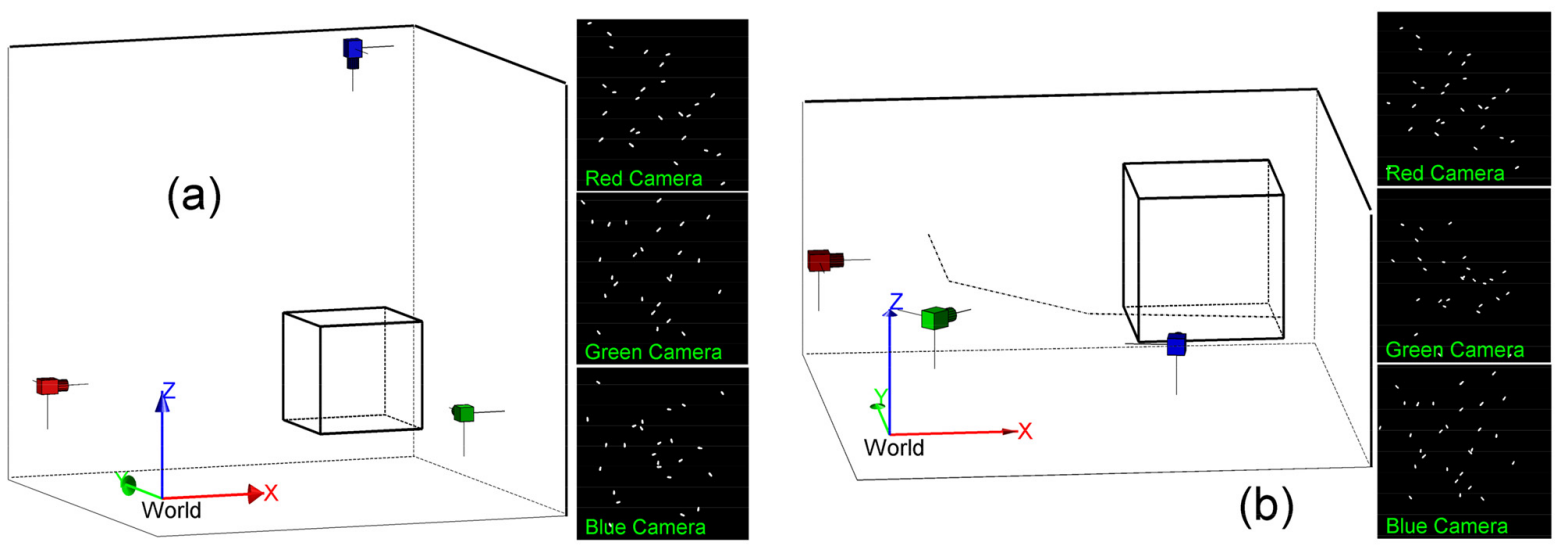
Cameras arrangement and snapshots. (a) Three cameras are orthogonally placed. (b) Three cameras are non-orthogonally placed.

The second step is to simulate the multi-camera system and to generate video datasets. Given a target’s location and orientation, a shape which represents the target was generated (see S2 Fig, the generative shape model). At each moment, all targets represented by shapes were filmed by simulated cameras. Images which were filmed by simulated cameras through time formed the dataset. Fig 5 shows the arrangement of cameras and the snapshot from each camera. The arrangements of cameras are different: orthogonally placed cameras (see Fig 5a) and non-orthogonally placed cameras (see Fig 5b). We applied the “Machine Vision Toolbox” [28] to simulate the three-camera system, and generated two image datasets. These two datasets were provided with source code (S1 File).

#### 3.1.2 Evaluation results

**Fig 6:**
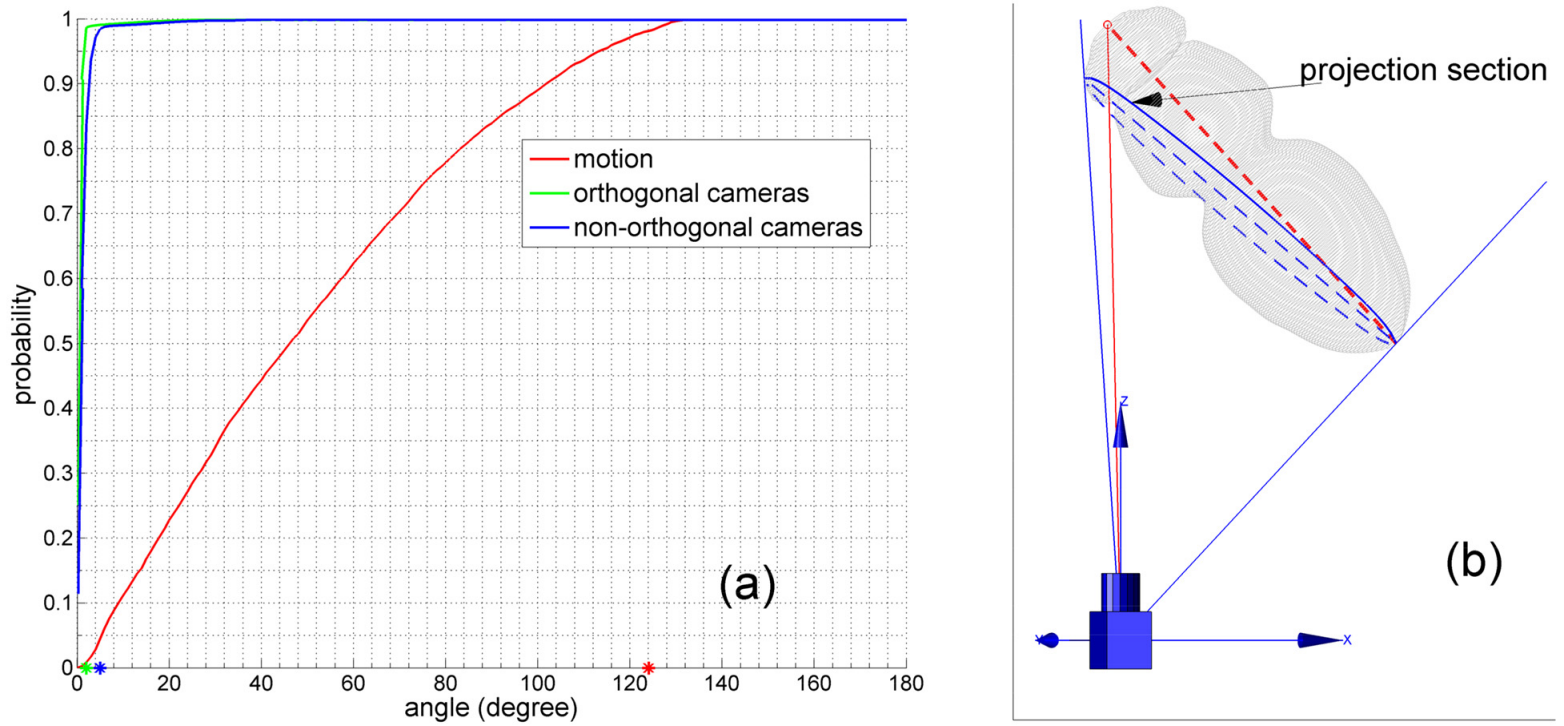
Performance evaluation on simulation data. (a) CDFs of angles distributions. Asterisks denote the 98% accuracy threshold, 2° (green), 5° (blue), and 124° (red). (b) The red dashed-line is the *center-axis* of a target. The solid red line connects the camera’s center and endpoint of the *center-axis*. The projection section is an intersection between the target’s body and a plane which is perpendicular to the plane determined by the two red lines. The projection section is drawn in blue. The blue dashed-line is the center axis of the projection section.

We evaluate the performance of the orientation estimation algorithm using the angle between the estimated orientation and the ground truth as metric. This metric measures accuracy of the estimated orientation. Given a certain target at moment *t*, we computed the angle between its estimated orientation and the ground truth. We repeated the computation for all targets through moments and collected all the angles. By evaluating the probability distribution of these angles, Fig 6a shows the cumulative distribution function (CDF) of the angles distribution. As expected, there is a large difference between a target’s orientation and its motion direction. If all target’s orientation were represented by their motion direction, Fig 6a shows that 98% angles belong to the interval [0°,124°], which is obviously a large error interval. It is known that vectors’ direction are inverse to each other if the angle between two vectors is > 90°. Fig 6a shows that 20% angles are > 90°, in which each is the angle between a target’s orientation and its motion direction. Therefore, data may be severely noised when we represented a target’s orientation by its motion direction.

Despite the arrangement of cameras, the estimated orientation is much more accurate. Moreover, the result on orthogonally placed cameras is slightly more accurate than the result on non-orthogonally placed cameras. Fig 6a shows that the former has an error interval [0°,2°] while the latter has an error interval [0°,5°], in order to achieve 98% accuracy. The reason, why the former is better, can be reasoned from the perspective effect.

Because of the perspective effect, there exists a projection section when a target was filmed by cameras (pinhole model, central cameras). Pixels of the target in an image are just the part of its body between the section and a certain camera. The projection section is illustrated in Fig 6b as a blue ellipse outline. The estimated orientation is indeed the orientation of the center axis of the projection section (see Fig 6b, the blue dashed-line). There is a slight difference between the center axis of the projection section and the *center-axis* of the target (see Fig 6b, the red dashed-line). Fig 6a shows that errors caused by perspective effect are slightly reduced if cameras were orthogonally placed.

### 3.2 Evaluation on real data

We housed fruit flies in a cubic transparent flight arena of side length 360 mm. The fruit flies were back-lit by planar lights. Three monochrome CMOS cameras were placed approximately 900 mm away from the arena (see S1 Fig, the experimental system). These cameras were geometrically calibrated and hardware synchronized. All cameras were set to 100 fps and the resolution of each camera was 2040*v* × 2048*h*. The supplements S1 Video, S2 Video, and S3 Video demonstrate the evaluation results.

**Fig 7:**
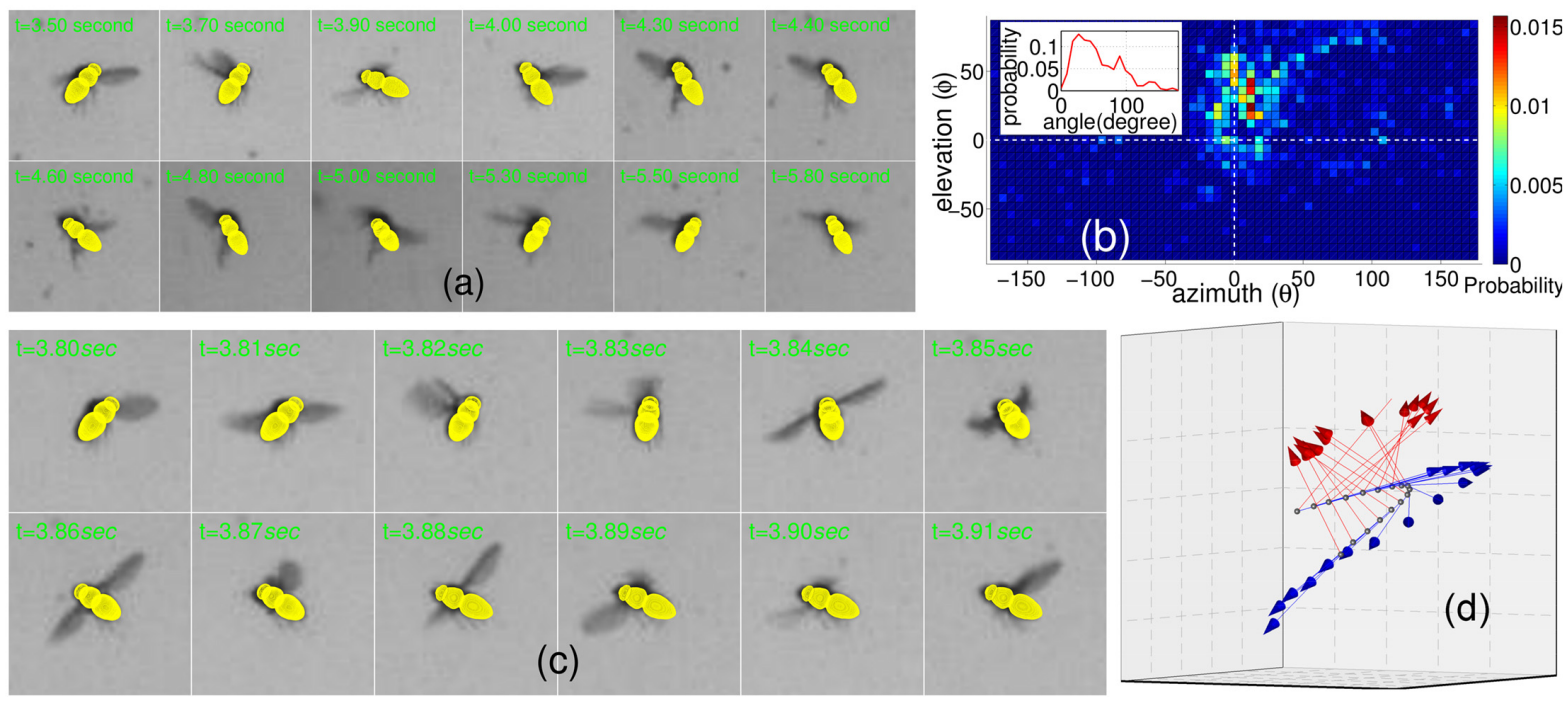
Performance evaluation on real data. (a) The snapshots of a fruit fly through time, in which shapes generated using the fly’s location *X*(*x*, *y*, *z*) and orientation *O*(*θ*, *ϕ*) are projected into the camera view and overlaid the zoomed image patches. (b) The distribution of angular direction of flies’ orientation with respect to their motion direction. The PDF represented by the 2D histogram is normalized. The inset is the PDF of the distribution of all angles between a fly’s orientation and its motion direction through time. (c) The snapshots of a fruit fly’s maneuver in twelve consecutive frames. Shapes are projected into the camera view and overlaid the zoomed image patches. (d) Three-dimensional comparison between the fly’s orientation and its motion direction during those twelve consecutive frames. Red arrows denote the fly’s orientation and blue arrows denote the motion direction.

Fig 7a shows the snapshots of a fruit fly through time. It shows the fruit fly often changed its orientation. The difference between a fly’s orientation and its motion direction can be measured by the angular direction of a fly’s orientation with respect to its motion direction. The angular direction is also defined by two angles, azimuth *θ* and elevation *ϕ*. Given a fruit fly *i*, we computed the angular direction of a fly’s orientation ${\vec{O}}_{i}$ with respect to its motion direction (velocity ${\vec{v}}_{i}$). That is, we measured the angular direction of a fly’s orientation against the coordinate system defined by rotating the x-axis of the world coordinate system to ${\vec{v}}_{i}$. The angular direction ⟨0°,0°⟩ always denotes the motion direction of a fruit fly through time. Fig 7b shows the probability density function (PDF) of the distribution of angular directions, in which the PDF is represented by the normalized 2D histogram. Each bin is of size 6° × 6°. The central bin which locates at point (0°,0°) denotes a fly’s orientation is the same as it motion direction. Fig 7b shows that events represented by the central bin happened under a very small probability.

On the other hand, though this probability distribution shows an obvious cluster, it disperses across a wide area. The fluctuation of elevation Δ*ϕ* between flies’ orientation and their motion direction is mainly Δ*ϕ* ∈ [−20°,60°]; and the fluctuation of azimuth Δ*θ* is mainly Δ*θ* ∈ [−30°,50°]. That is, angles between flies’ orientation and their motion direction should also disperse across a large interval. The angle between a fly’s orientation and its motion direction can also measure the difference between them. The inset of Fig 7b shows the PDF of the angle distribution, in which each angle is the angle between a fly’s orientation and its motion direction. The PDF is widely dispersive. It suggests that the difference between a fly’s orientation and its motion direction is too large to represent a fly’s orientation by its motion direction at moments.

Moreover, previous studies [18, 29] have demonstrated the flight trajectories of many fly species consist of straight flight sequences interspersed with rapid changes in heading termed saccades. Fig 7c shows a fruit fly’s maneuvers in twelve consecutive frames, in which the fruit fly probably took a saccade. And Fig 7d shows the variation of the fly’s orientation and its motion direction during the course of the saccade. It shows clearly that the angle between a fly’s orientation and its motion direction is large in the period. That is, data may be severely noised when representing a fly’s orientation by its motion direction.

## Conclusions

In this paper, we proposed a method to estimate a fruit fly’s orientation using image cues. The orientation estimation algorithm can be incorporated into tracking algorithms. The computing phase (step 1 to step 10 of Algorithm 1) of the orientation estimation algorithm can be written as one equation and is consistent with the line reconstruction algorithm in computer vision field [24]. Computing a fly’s orientation needs in practice only dozens of microseconds, if the the input of the algorithm is provided. Because of the perspective effect, the estimated orientation is slightly different to the real one. Though the orthogonally placed cameras produced the best result, our experiments demonstrate that the arrangement of cameras has little influence on the performance of accuracy. Further, the orientation estimation algorithm is suitable for any targets if their body can be represented by ellipsoids.

## Supporting Information

S1 Fig The illustration of the equipment arrangement. (PDF)

S2 Fig The generative shape model of the body of *Drosophila*. (PDF)

S1 File The raw code. (ZIP)

S1 Video Demo video of orientation estimation. (MOV)

S2 Video Demo video of orientation estimation. (MOV)

S3 Video Demo video of orientation estimation. (MOV)
