# Supplementary material for: Estimating Orientation of Flying Fruit Flies
Source: PLoS One. 2015 Jul 14;10(7):e0132101. doi: 10.1371/journal.pone.0132101 (PMC4501570; doi:10.1371/journal.pone.0132101)
Supplement: S1 Fig — (PDF) [file pone.0132101.s001.pdf]

The cubic flight arena was a Lucite container of size $360mm \times 360mm \times 360mm$ (inner). The arena was glued by five mutually patched transparent acrylic planks by the chloroform. A feeding-tube matched open-top sunroof in $50mm$ diameter circle was handled on escape-proof inner besieged flange. Three monochrome high-speed digital video cameras (IO Industries Canada, Flare 4M 180-CL, $2040v \times 2048h$ pixels at 100 fps) were placed outside of the cubic arena against two orthogonally placed back-lit cool-running daylight fluorescent lamp arrays ($200W$). Each lamp array was covered by a diffusion sheet to generate gentle and flicker-free planar illumination. The cameras were mounted with $17-35mm$ lens.

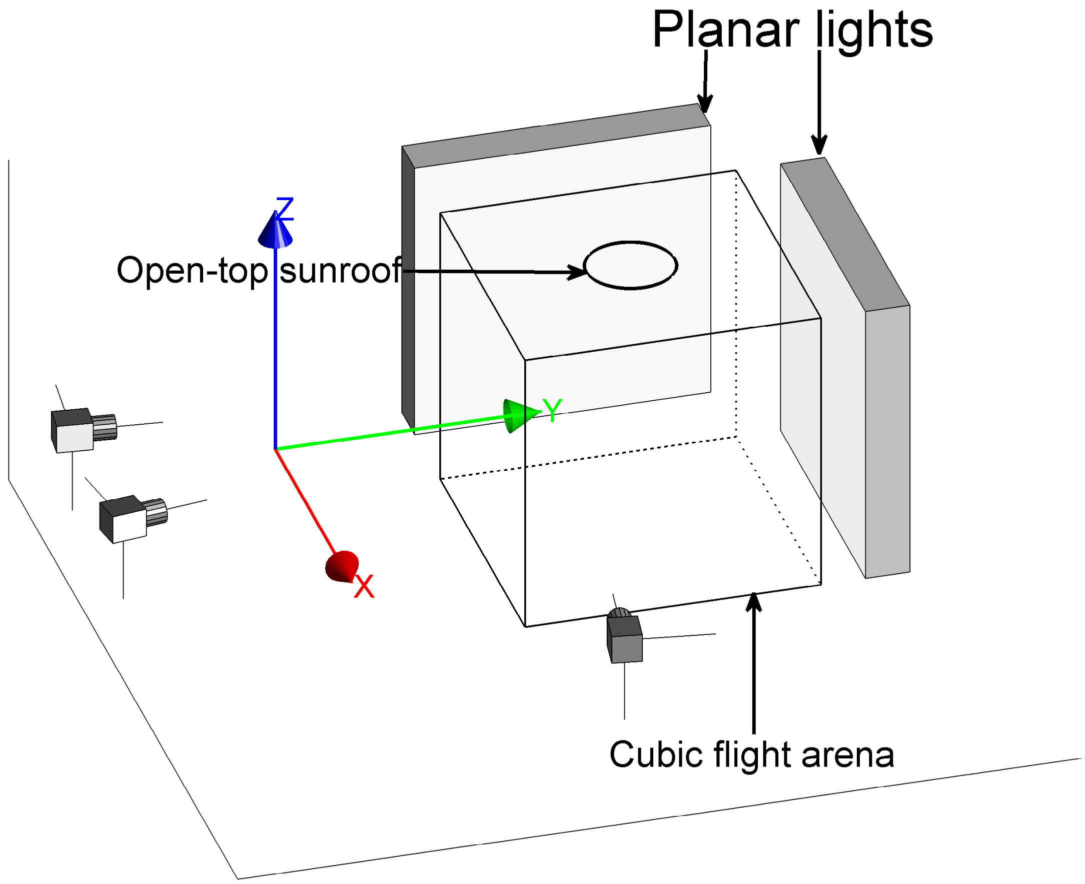

Figure 1. The illustration of the equipment arrangement. Two high-speed cameras were placed at one side of the arena. Another camera was placed at another side of the arena. These three cameras were geometrically calibrated and hardware synchronized. The two orthogonally placed back-lit cool-running daylight fluorescent lamp arrays were placed at the side of the arena opposite the cameras. The world's coordinate system is color coded.
