# Supplementary material for: Estimating Orientation of Flying Fruit Flies
Source: PLoS One. 2015 Jul 14;10(7):e0132101. doi: 10.1371/journal.pone.0132101 (PMC4501570; doi:10.1371/journal.pone.0132101)
Supplement: S2 Fig — (PDF) [file pone.0132101.s002.pdf]

We implemented a parameterized generative shape $\Upsilon^3(X_t, O_t, \tilde{l})$ in term of a profile curve revolving around the *center-axis*. The *center-axis* is a line-segment which is defined by $\{X_t(x, y, z), O_t(\theta, \phi), \tilde{l}\}$. A similar idea of generating shape model had been presented by Fontaine *et al.* [1].

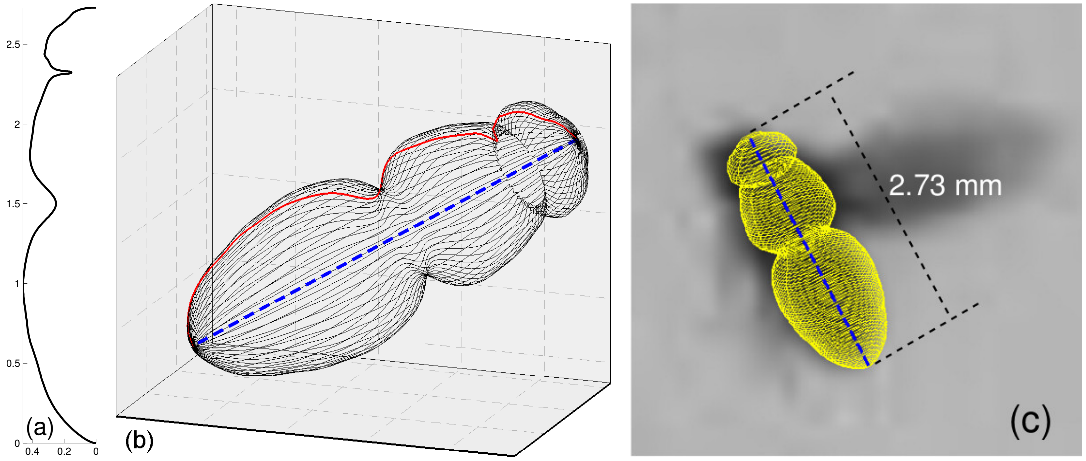

Figure 1. The generative shape model of *Drosophila*. (a) The profile curve, denotes by ρ. (b) The shape Υ^3 is generated by the profile curve ρ revolving around the *center axis* in 3D space. (c) An image of a fruit fly overlaid by the shape model. The average body length is $\tilde{l} = 2.73$ mm in our experiments.

References

1. Fontaine EI, Zabala F, Dickinson MH, Burdick JW (2009) Wing and body motion during flight initiation in *Drosophila* revealed by automated visual tracking. J Exp Biol 212: 1307-1323. 1
