# Supplementary figures and images for: Estimating Orientation of Flying Fruit Flies (part 2 of 2)
Source: PLoS One. 2015 Jul 14;10(7):e0132101. doi: 10.1371/journal.pone.0132101 (PMC4501570; doi:10.1371/journal.pone.0132101)

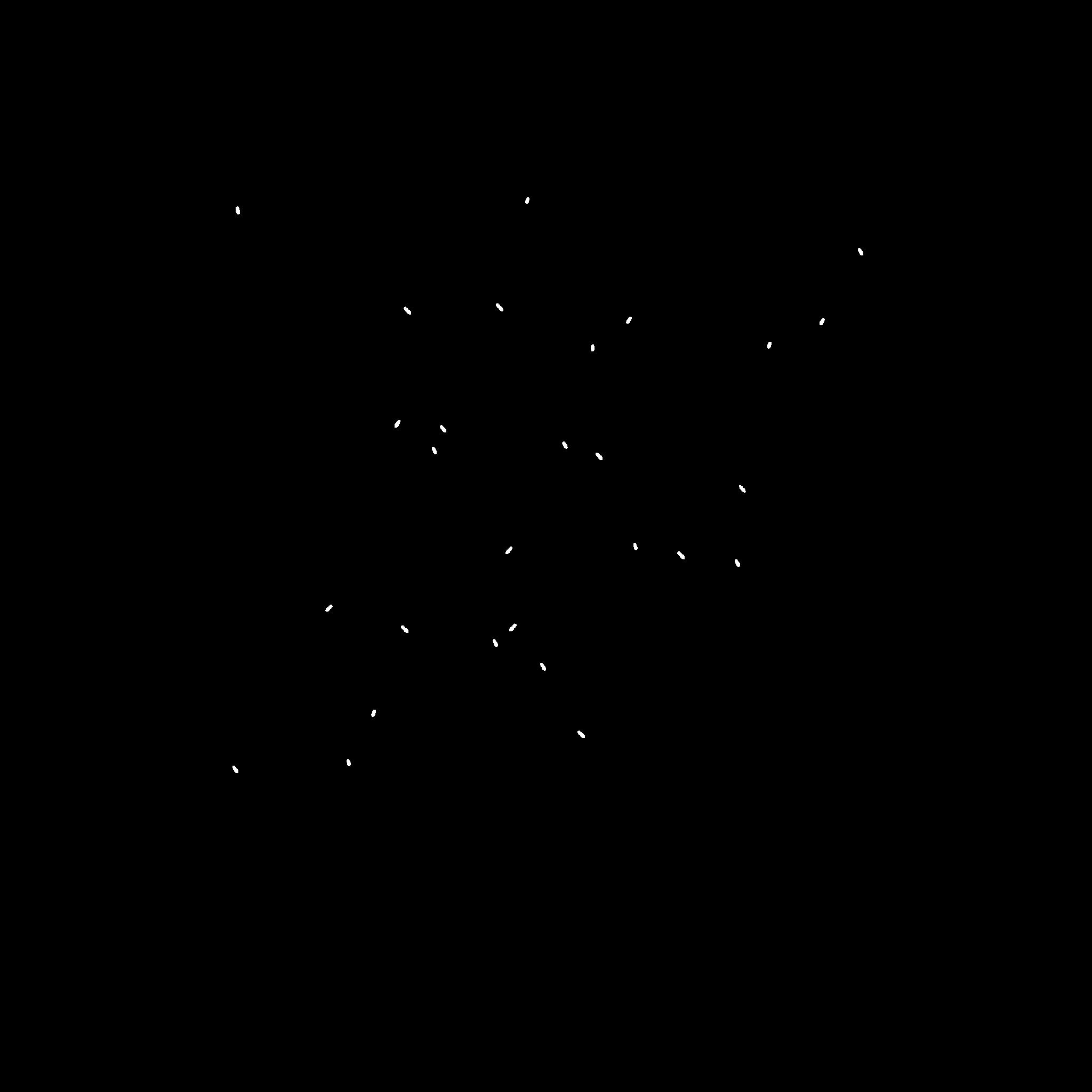

Supplement: S1 File — (ZIP) [file pone.0132101.s003.zip › ORsrc/nonortho/simu028/camx/imx103.jpg]

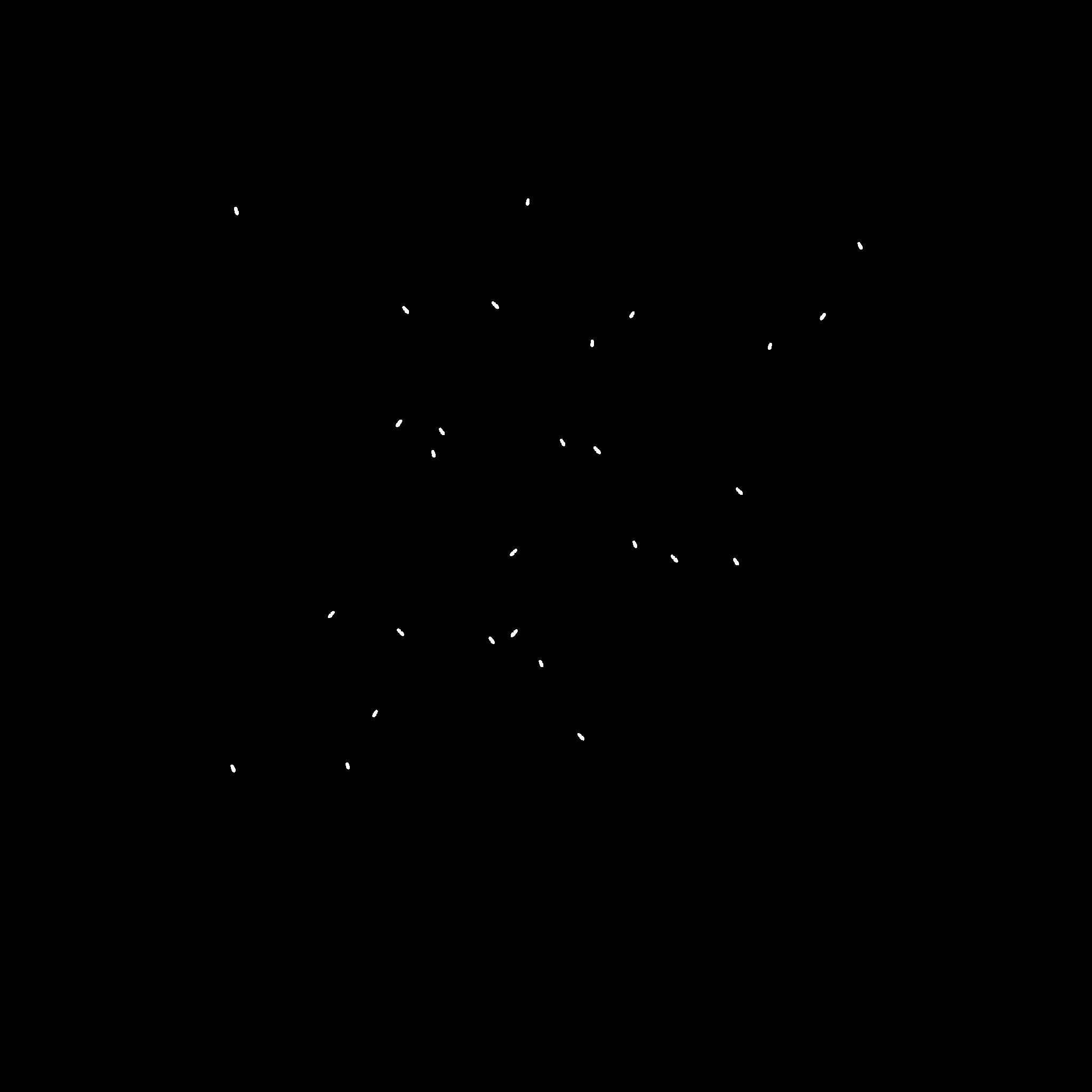

Supplement: S1 File — (ZIP) [file pone.0132101.s003.zip › ORsrc/nonortho/simu028/camx/imx104.jpg]

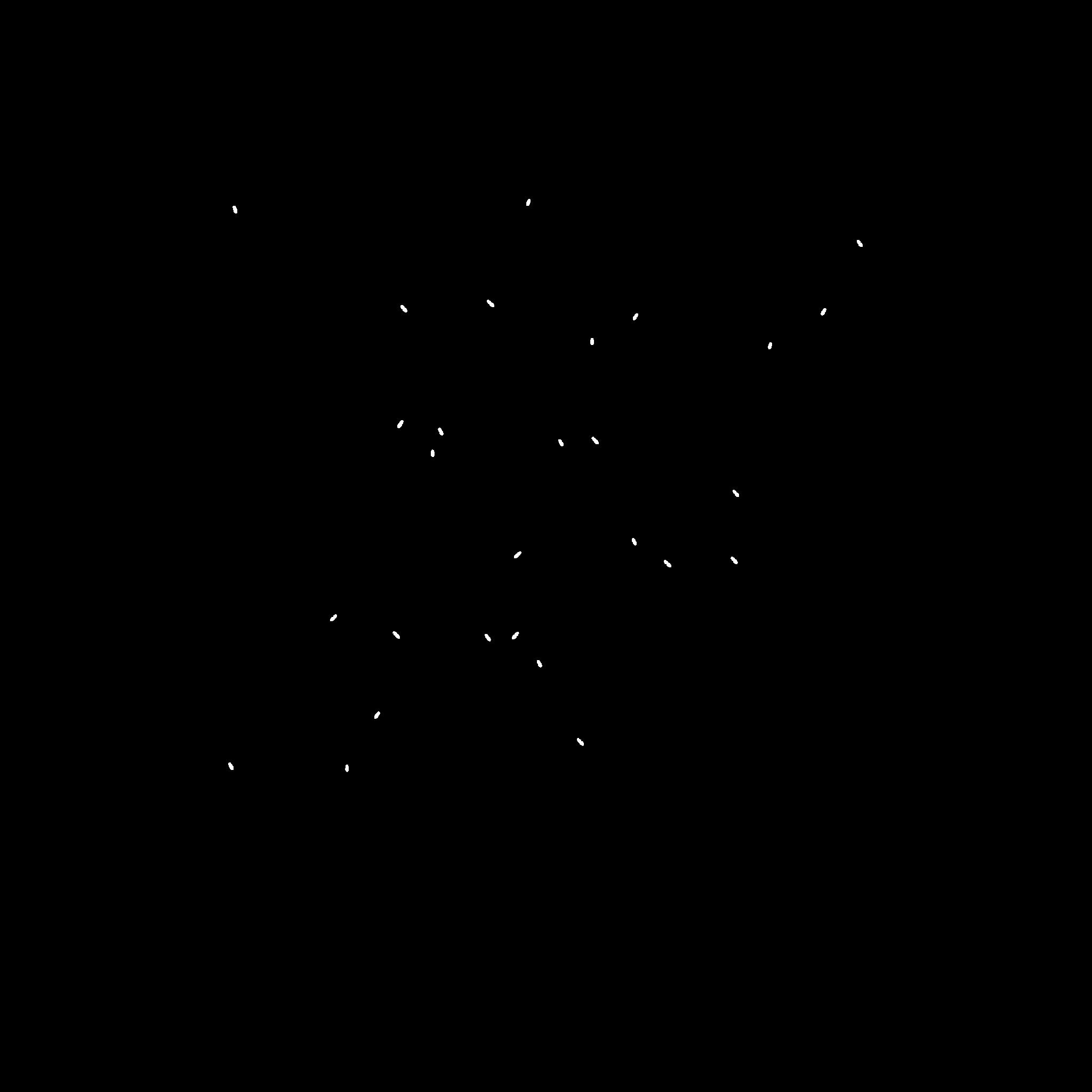

Supplement: S1 File — (ZIP) [file pone.0132101.s003.zip › ORsrc/nonortho/simu028/camx/imx105.jpg]

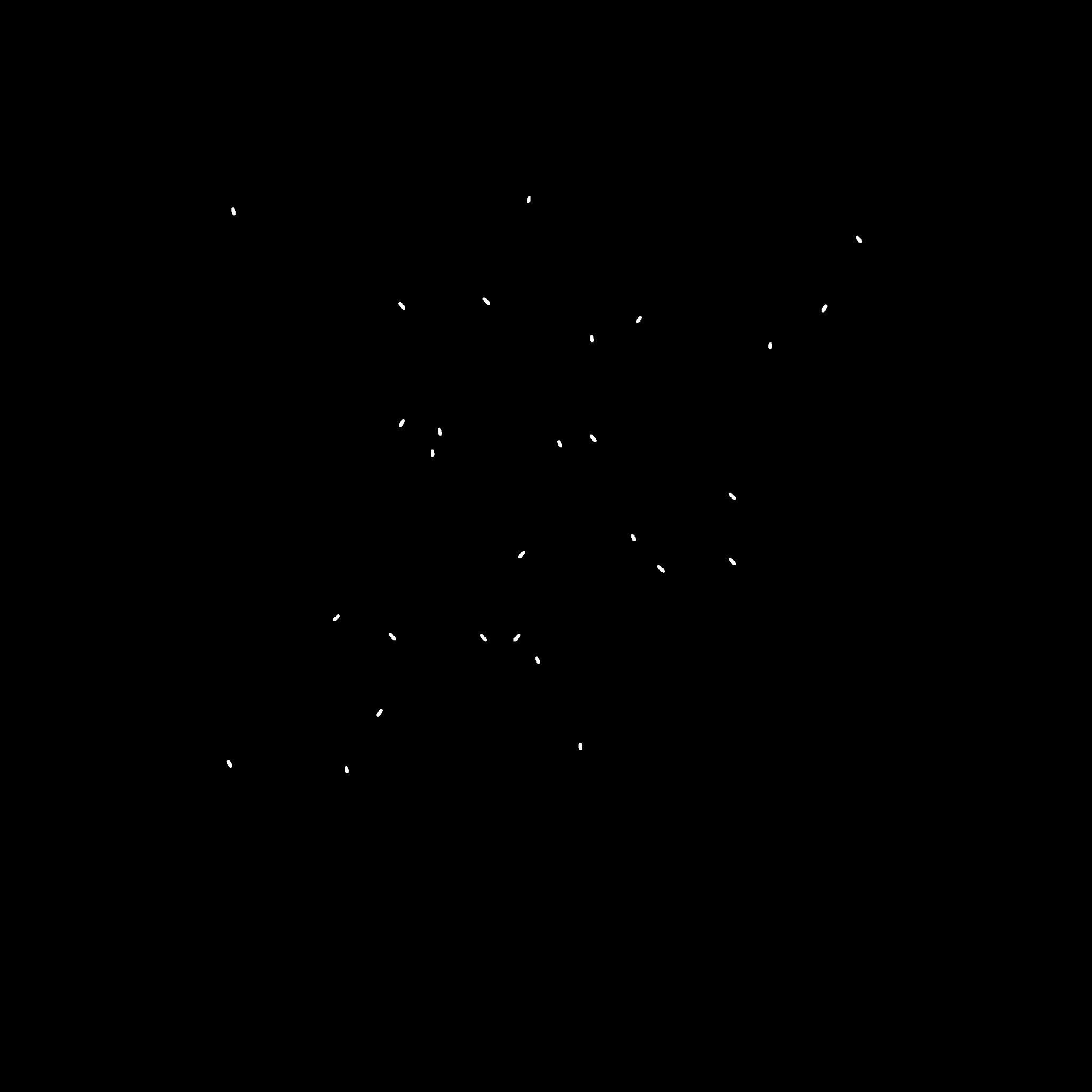

Supplement: S1 File — (ZIP) [file pone.0132101.s003.zip › ORsrc/nonortho/simu028/camx/imx106.jpg]

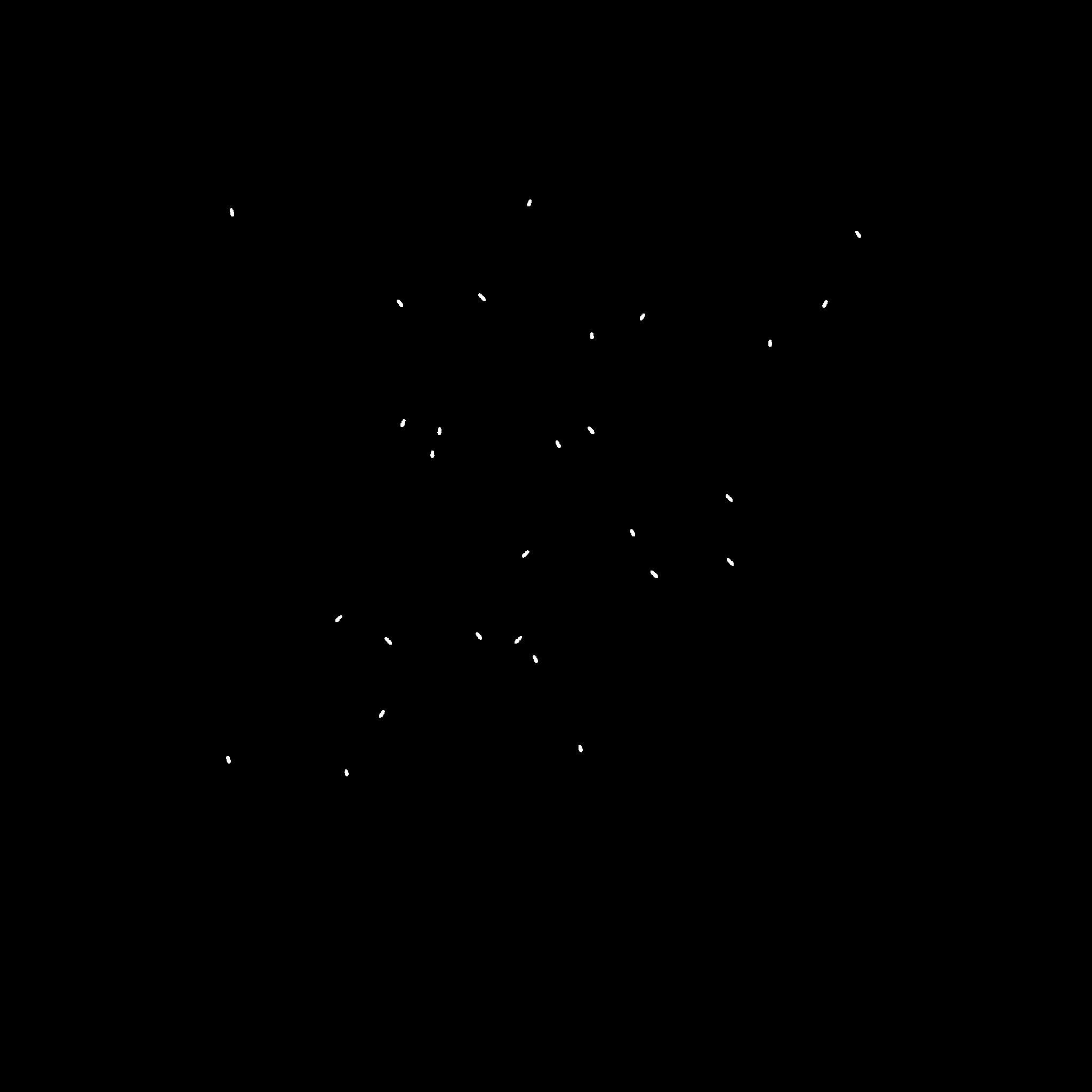

Supplement: S1 File — (ZIP) [file pone.0132101.s003.zip › ORsrc/nonortho/simu028/camx/imx107.jpg]

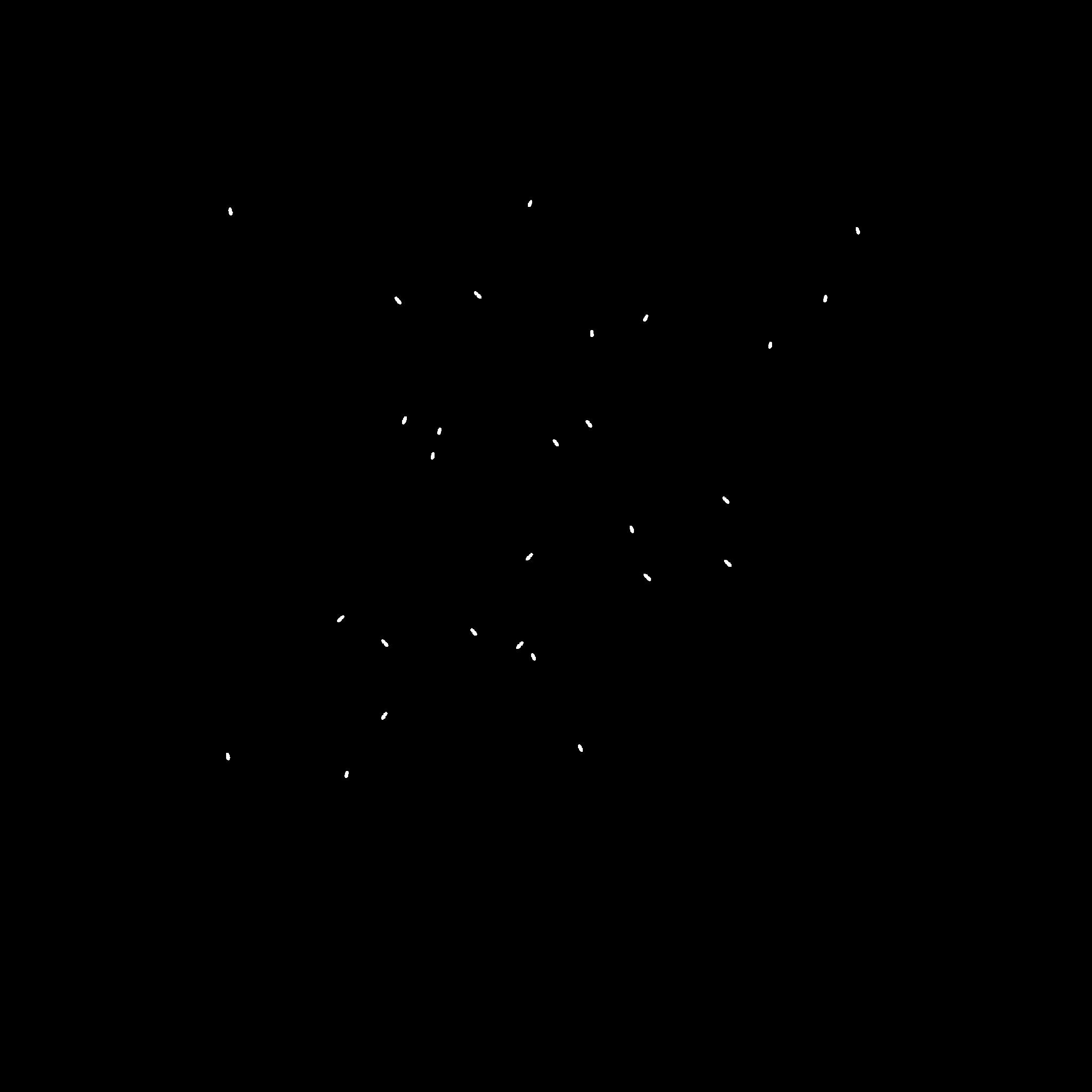

Supplement: S1 File — (ZIP) [file pone.0132101.s003.zip › ORsrc/nonortho/simu028/camx/imx108.jpg]

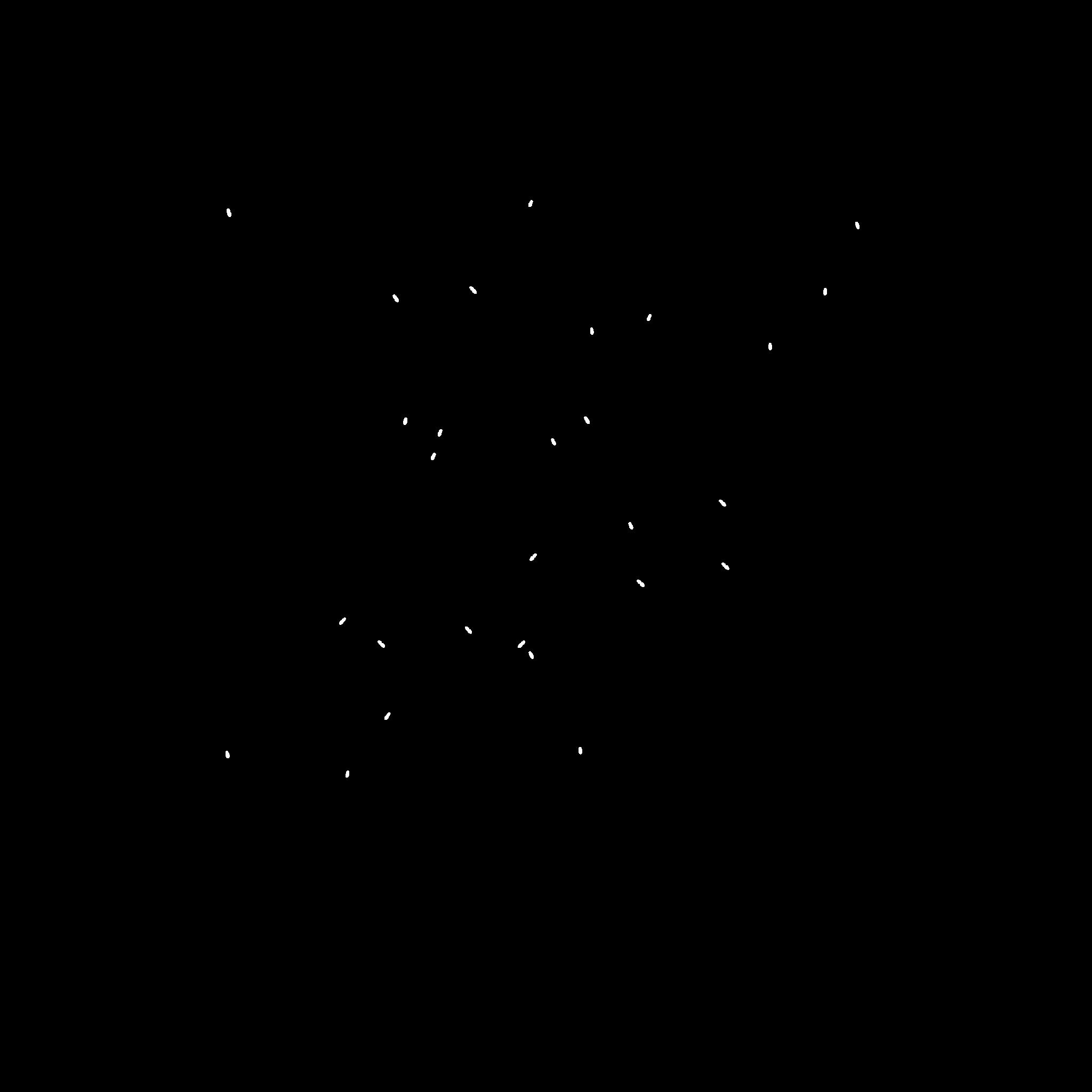

Supplement: S1 File — (ZIP) [file pone.0132101.s003.zip › ORsrc/nonortho/simu028/camx/imx109.jpg]

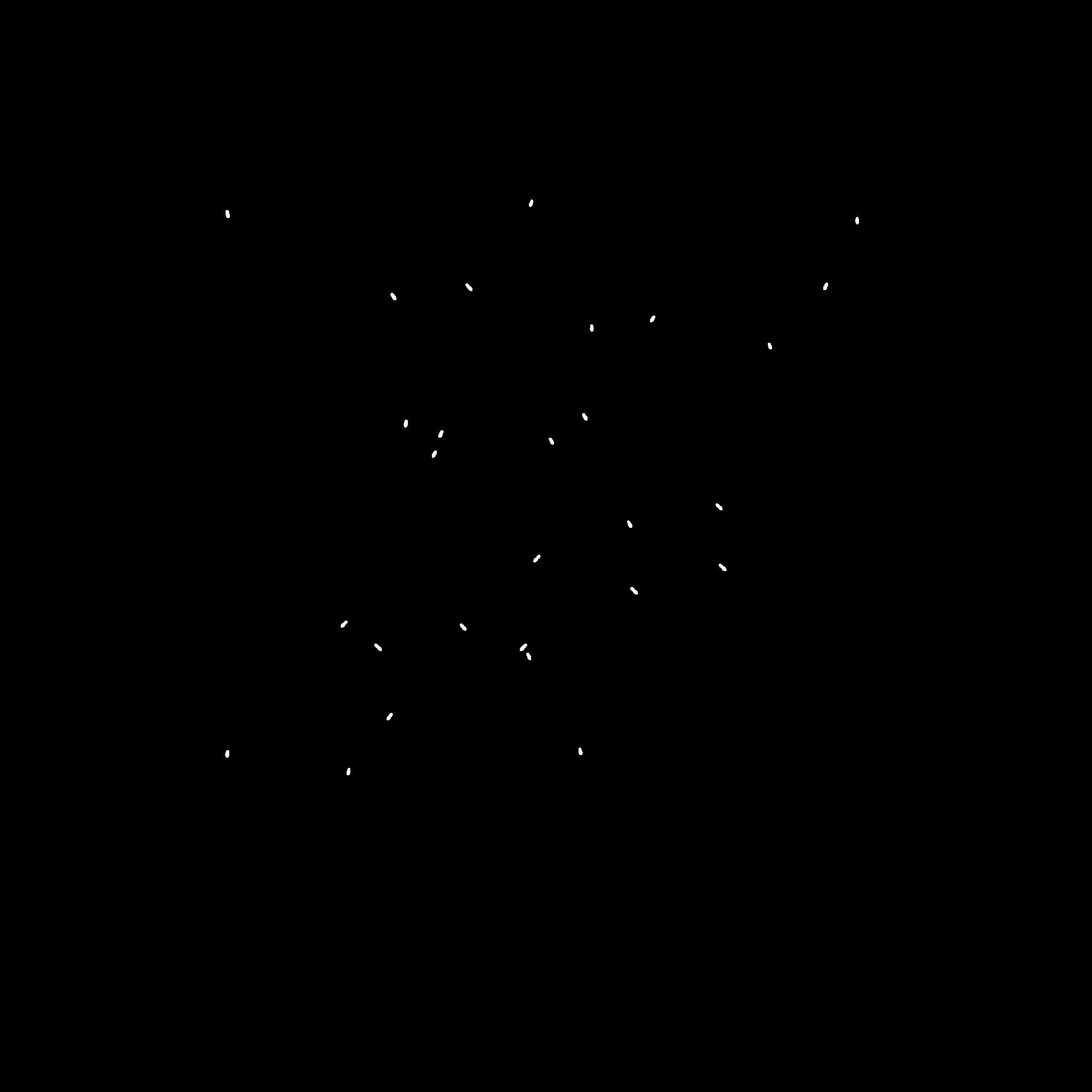

Supplement: S1 File — (ZIP) [file pone.0132101.s003.zip › ORsrc/nonortho/simu028/camx/imx110.jpg]

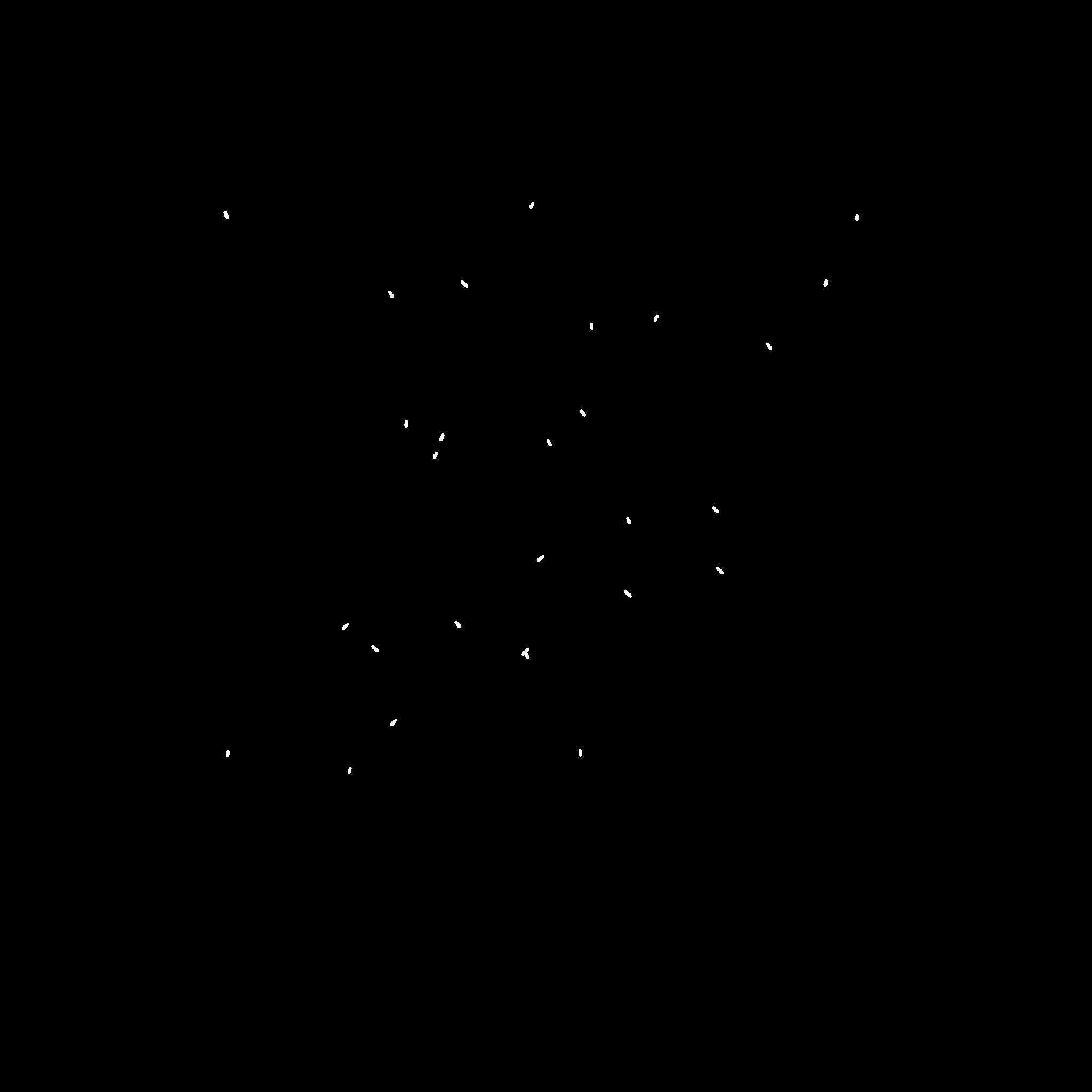

Supplement: S1 File — (ZIP) [file pone.0132101.s003.zip › ORsrc/nonortho/simu028/camx/imx111.jpg]

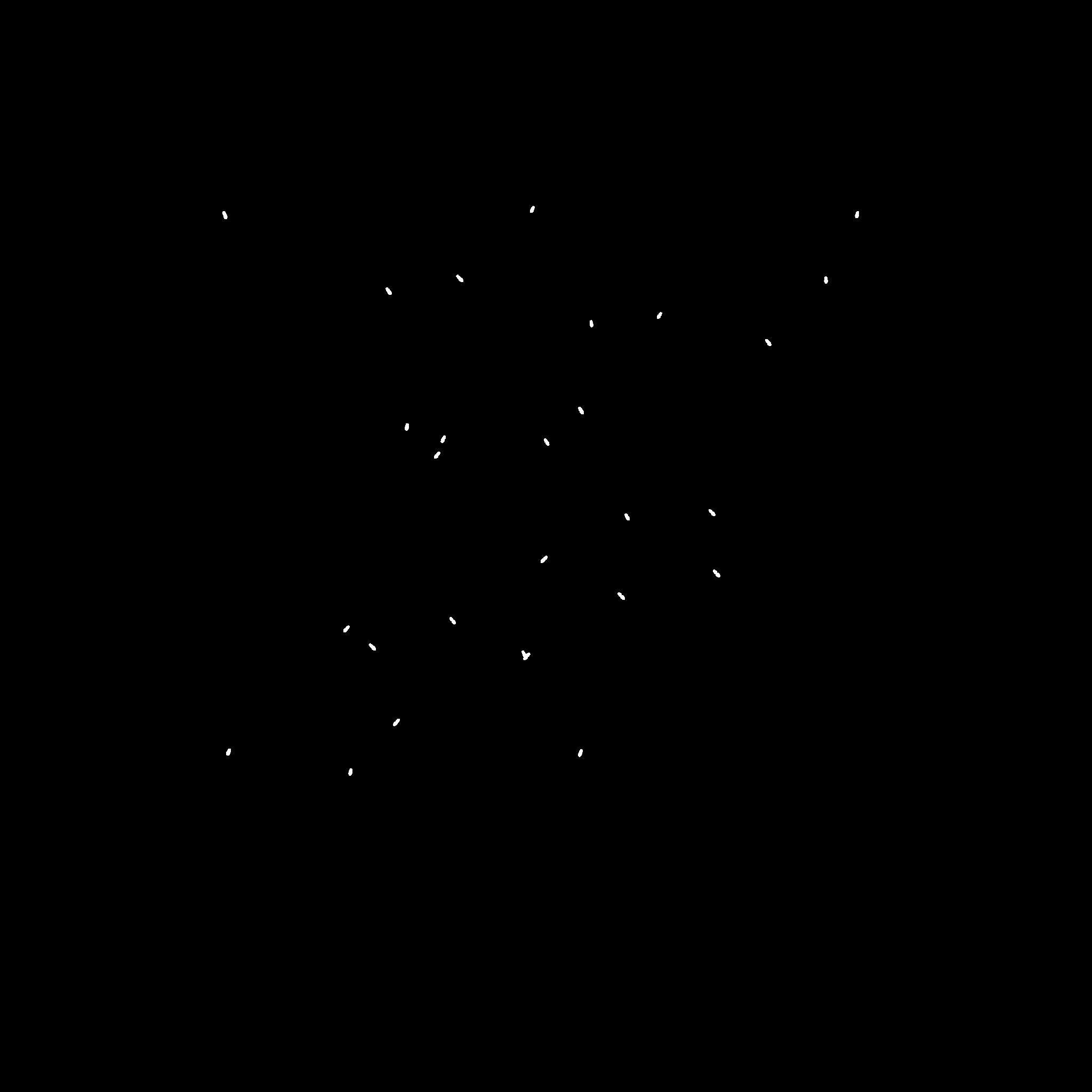

Supplement: S1 File — (ZIP) [file pone.0132101.s003.zip › ORsrc/nonortho/simu028/camx/imx112.jpg]

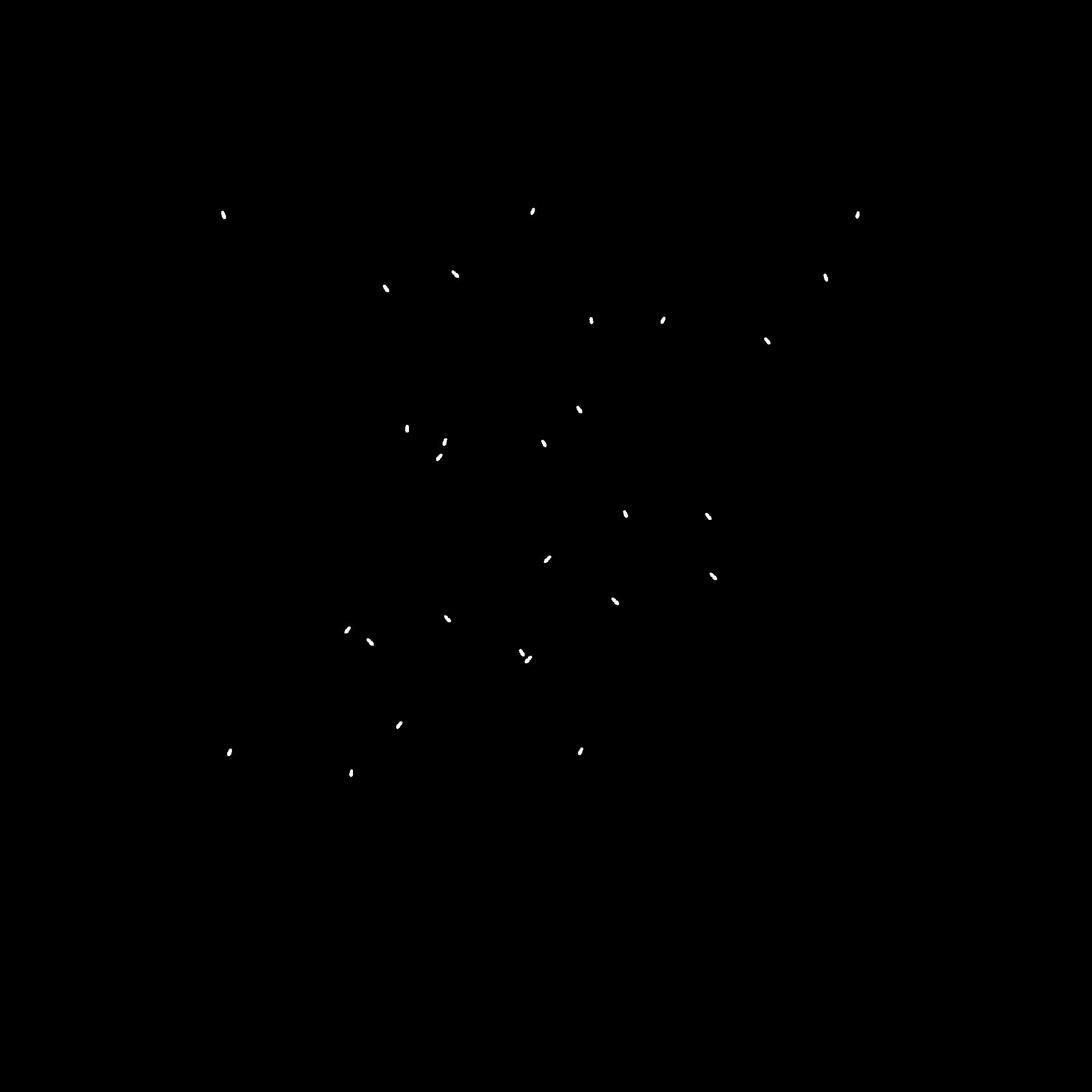

Supplement: S1 File — (ZIP) [file pone.0132101.s003.zip › ORsrc/nonortho/simu028/camx/imx113.jpg]

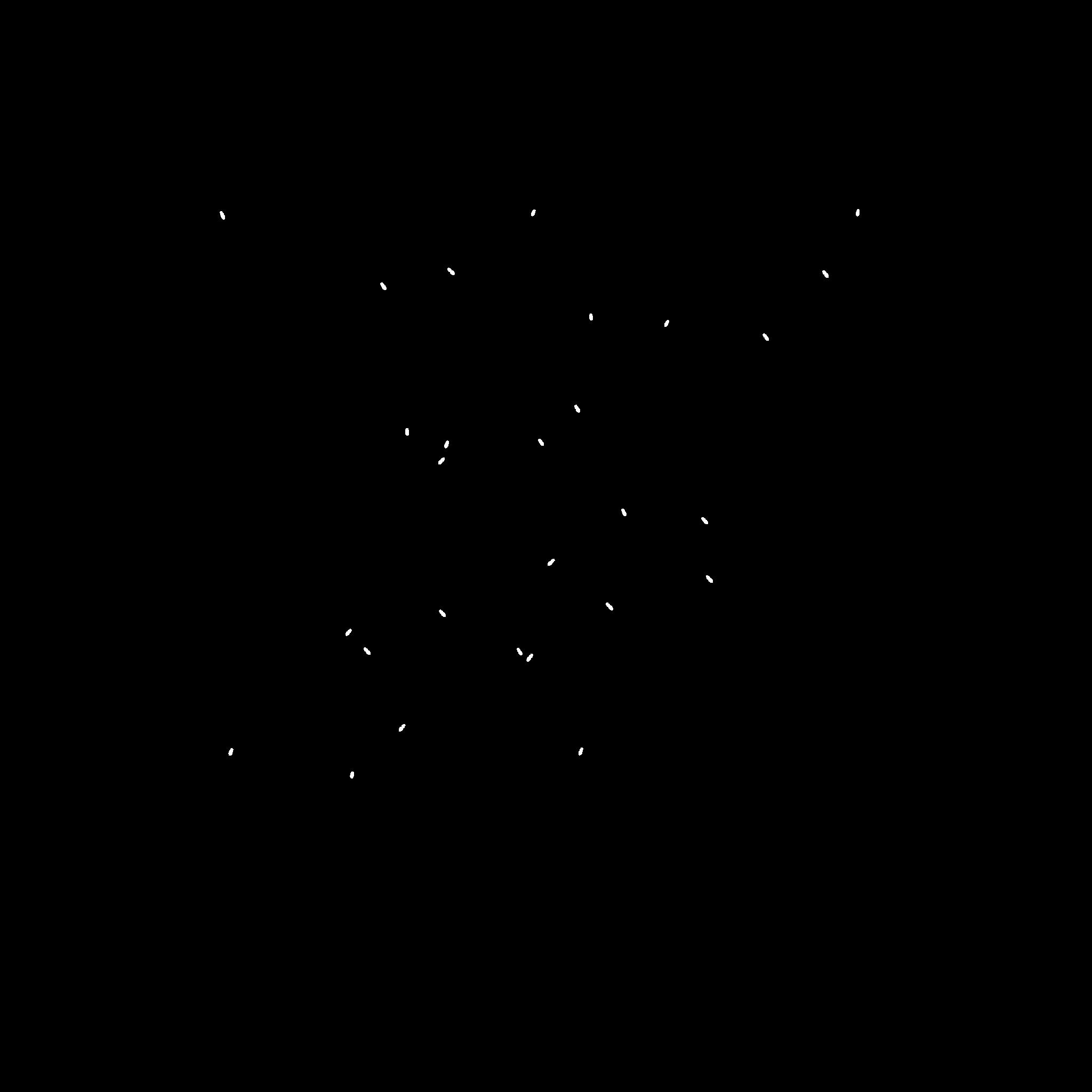

Supplement: S1 File — (ZIP) [file pone.0132101.s003.zip › ORsrc/nonortho/simu028/camx/imx114.jpg]

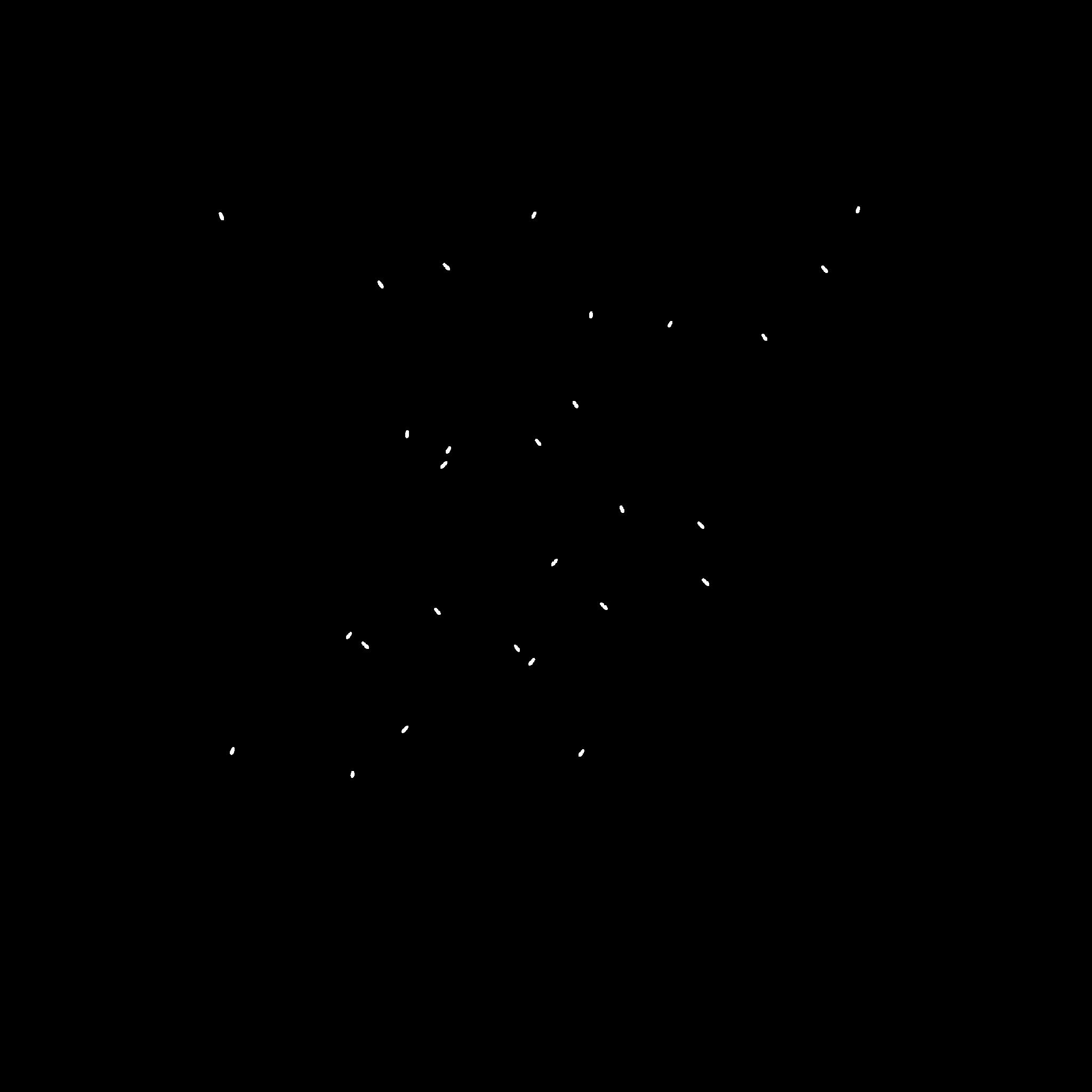

Supplement: S1 File — (ZIP) [file pone.0132101.s003.zip › ORsrc/nonortho/simu028/camx/imx115.jpg]

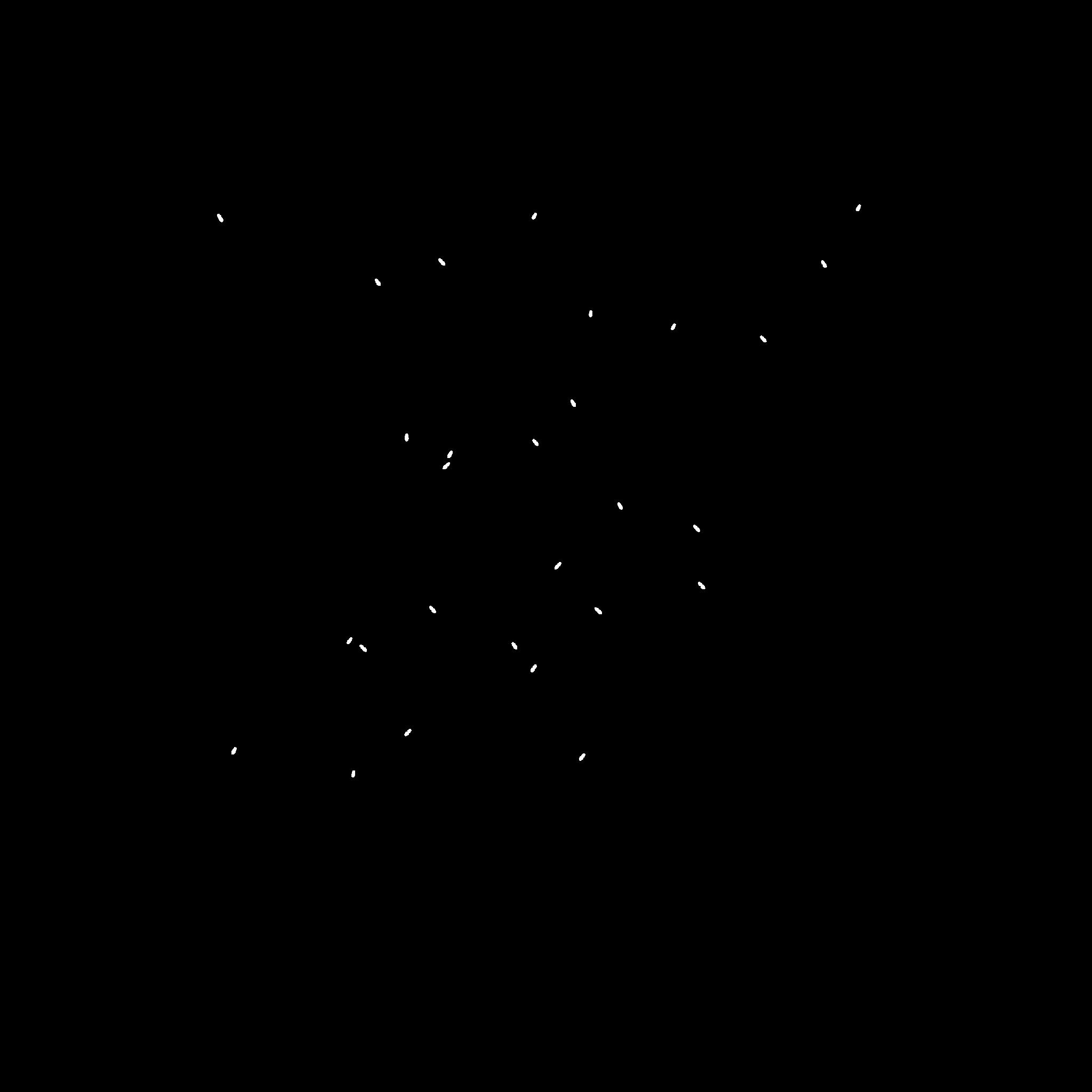

Supplement: S1 File — (ZIP) [file pone.0132101.s003.zip › ORsrc/nonortho/simu028/camx/imx116.jpg]

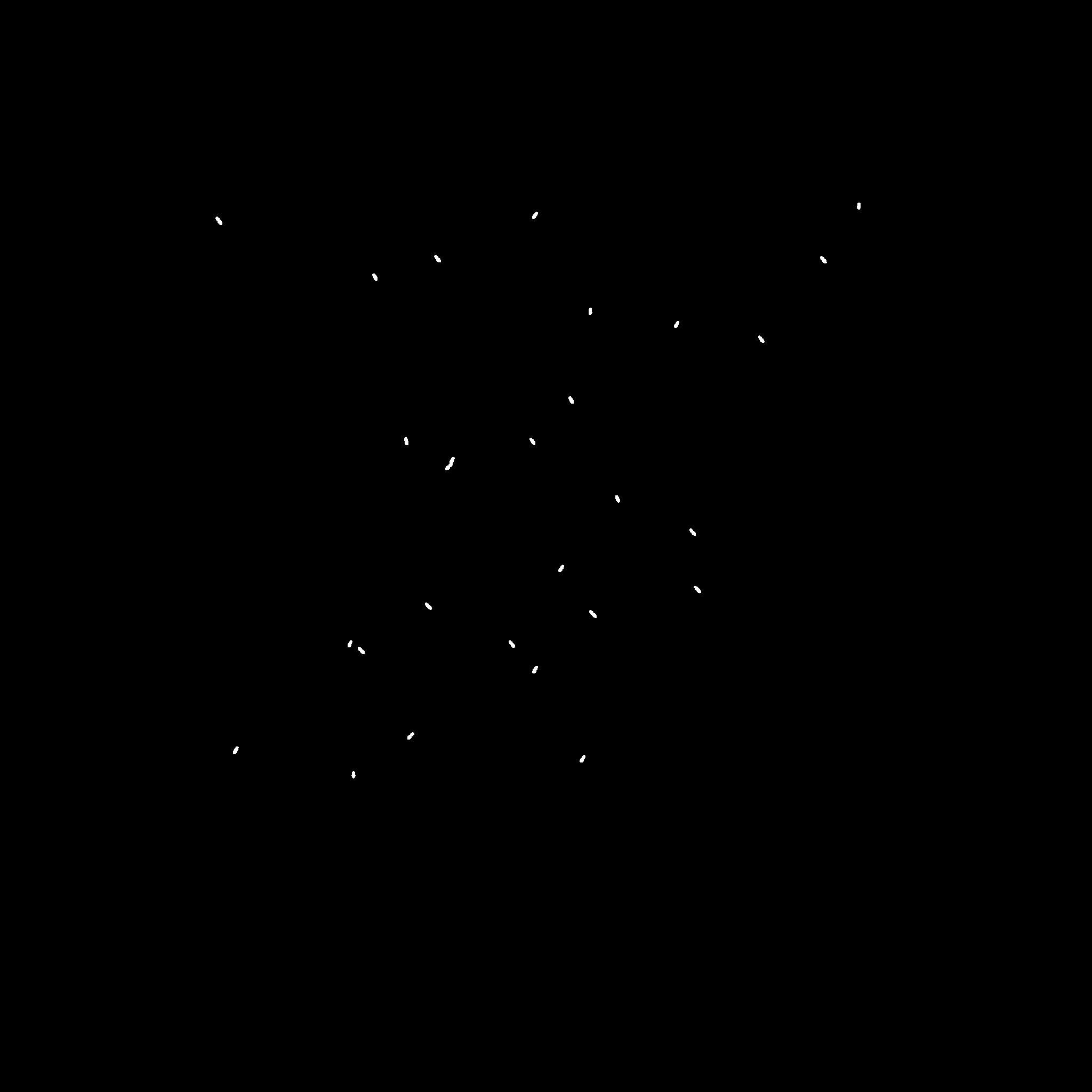

Supplement: S1 File — (ZIP) [file pone.0132101.s003.zip › ORsrc/nonortho/simu028/camx/imx117.jpg]

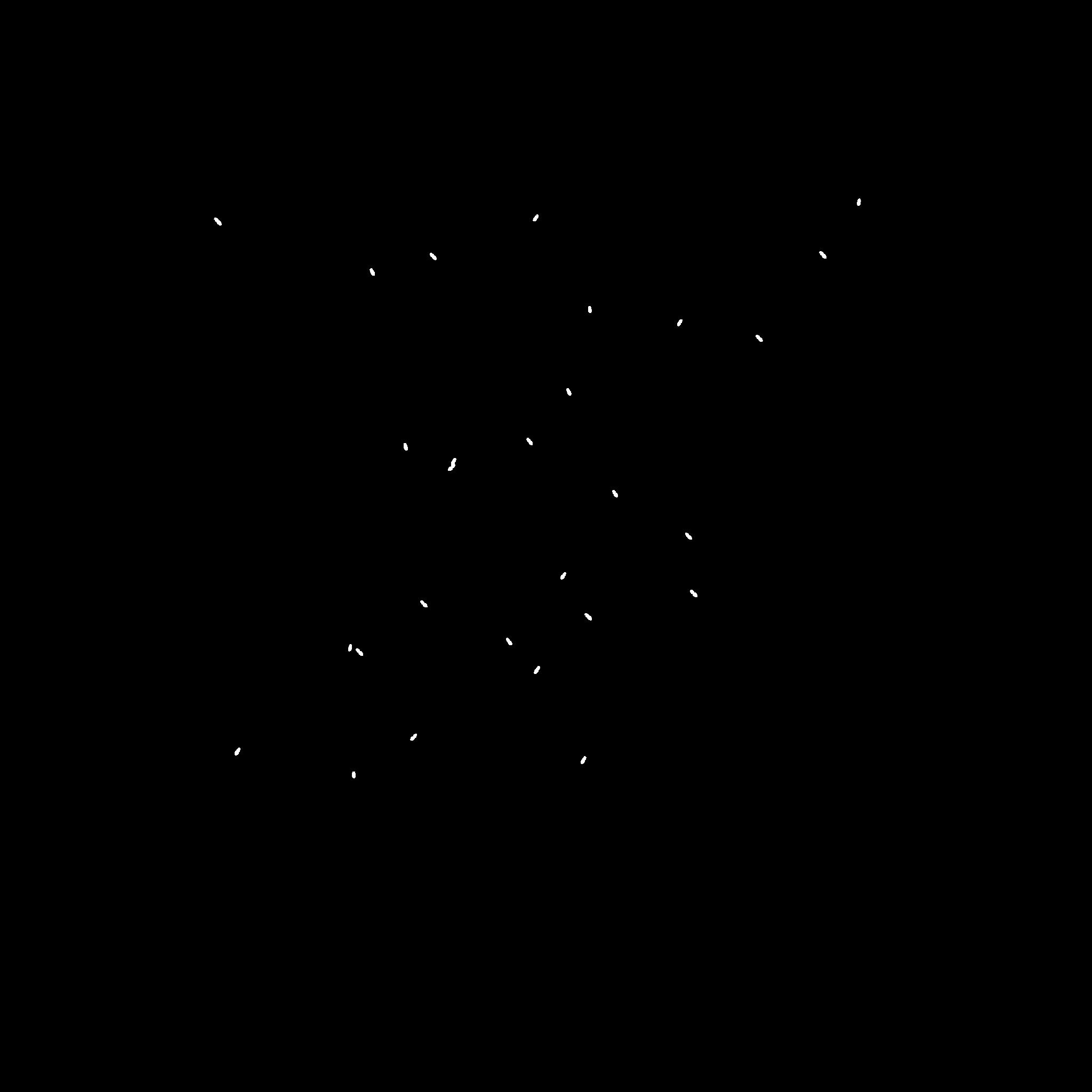

Supplement: S1 File — (ZIP) [file pone.0132101.s003.zip › ORsrc/nonortho/simu028/camx/imx118.jpg]

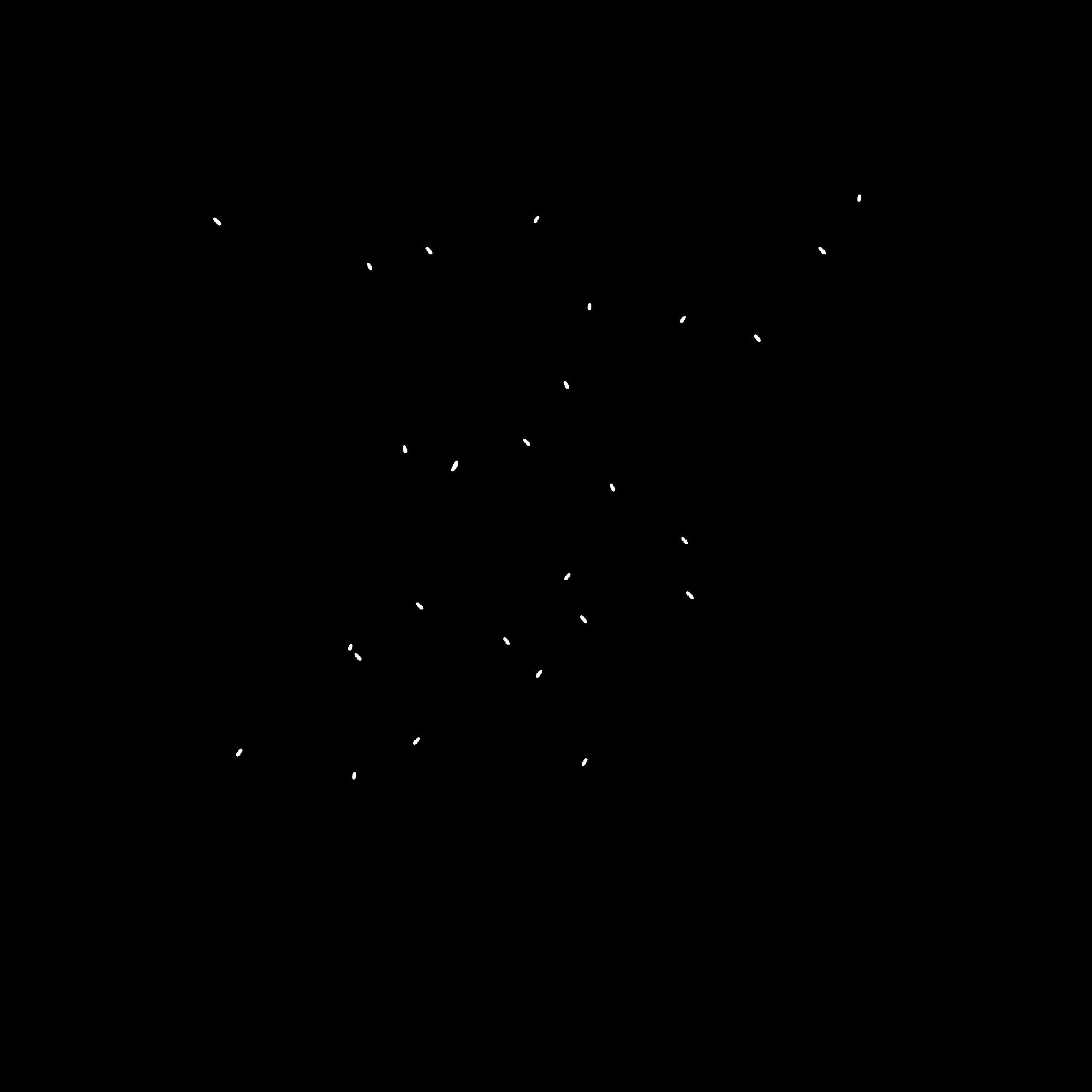

Supplement: S1 File — (ZIP) [file pone.0132101.s003.zip › ORsrc/nonortho/simu028/camx/imx119.jpg]

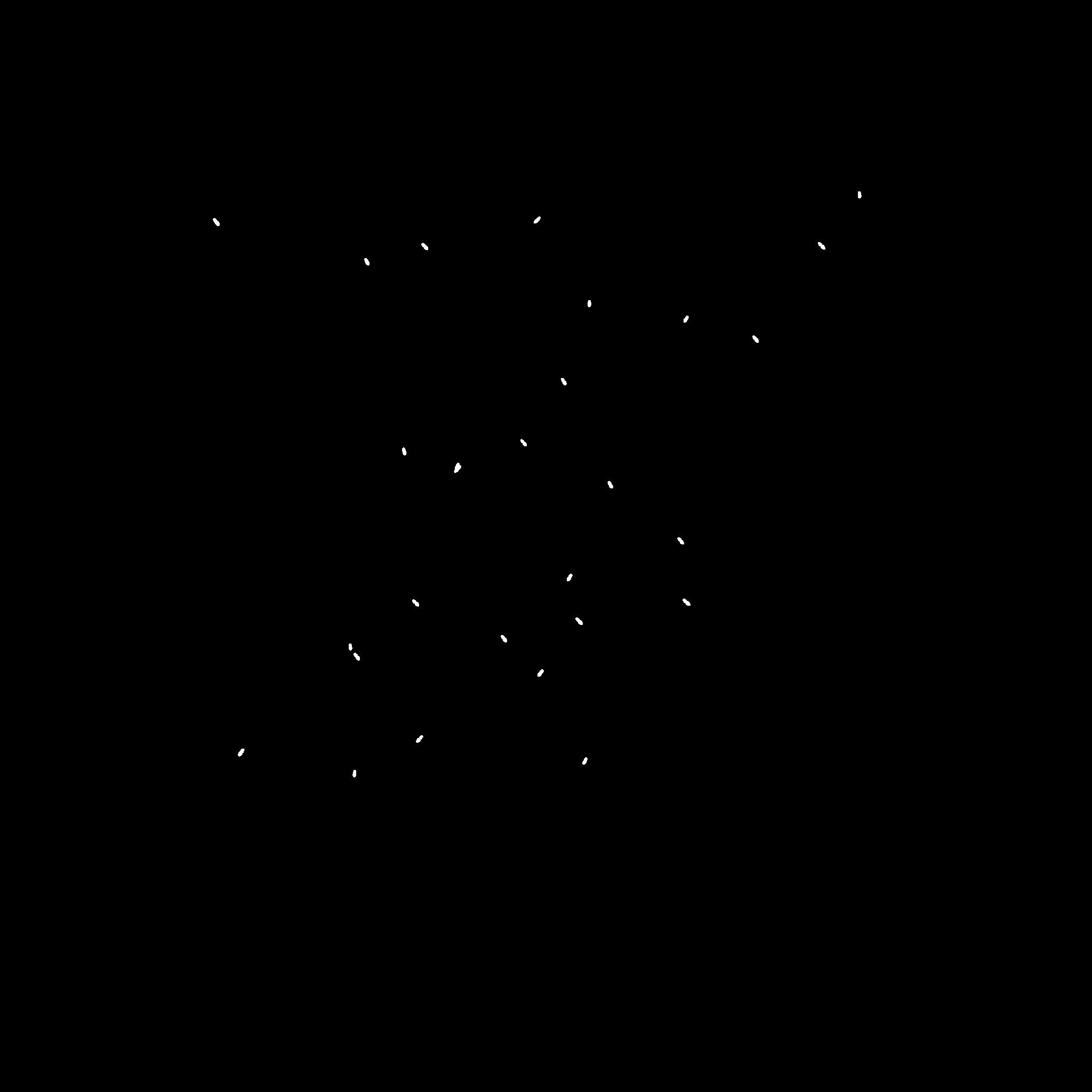

Supplement: S1 File — (ZIP) [file pone.0132101.s003.zip › ORsrc/nonortho/simu028/camx/imx120.jpg]

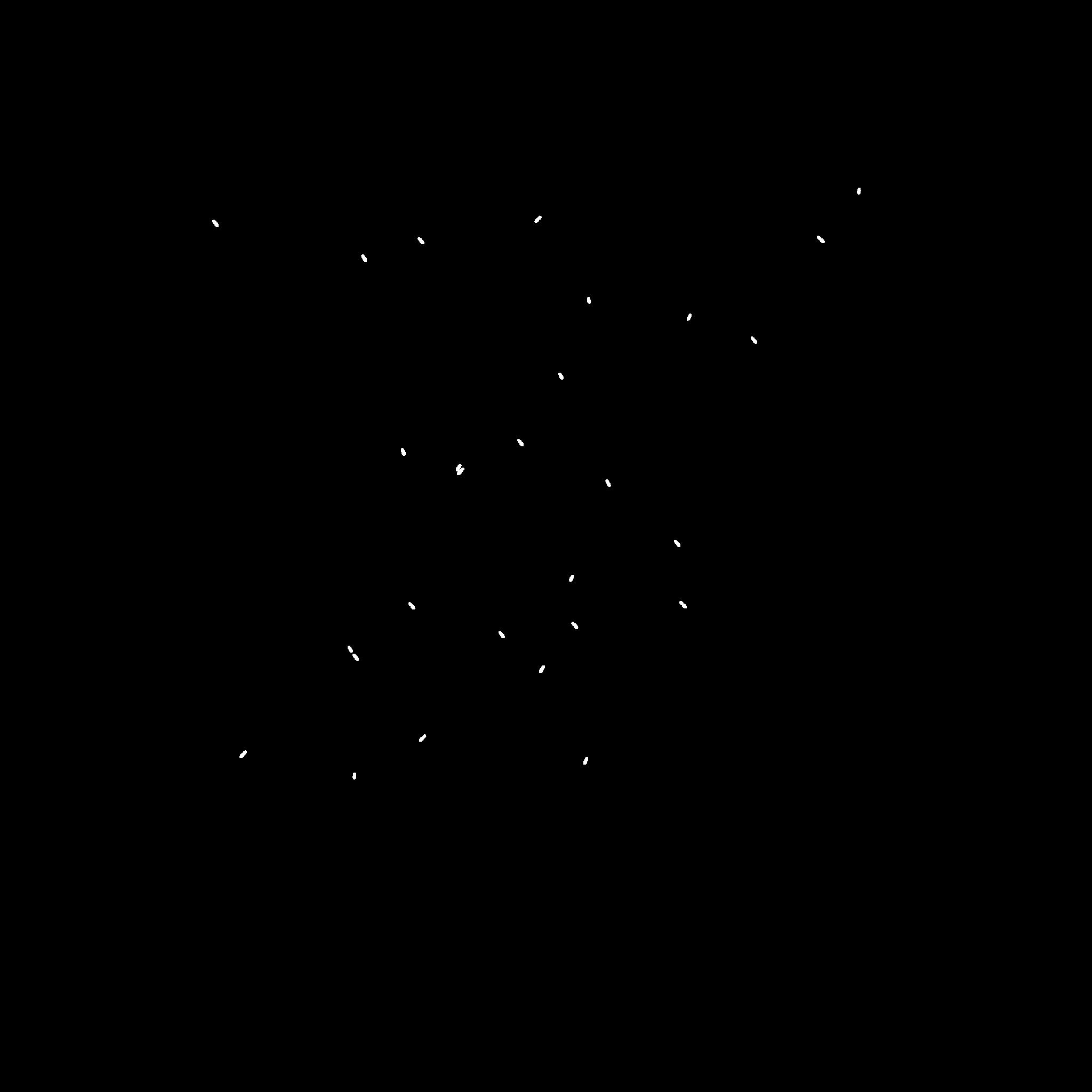

Supplement: S1 File — (ZIP) [file pone.0132101.s003.zip › ORsrc/nonortho/simu028/camx/imx121.jpg]

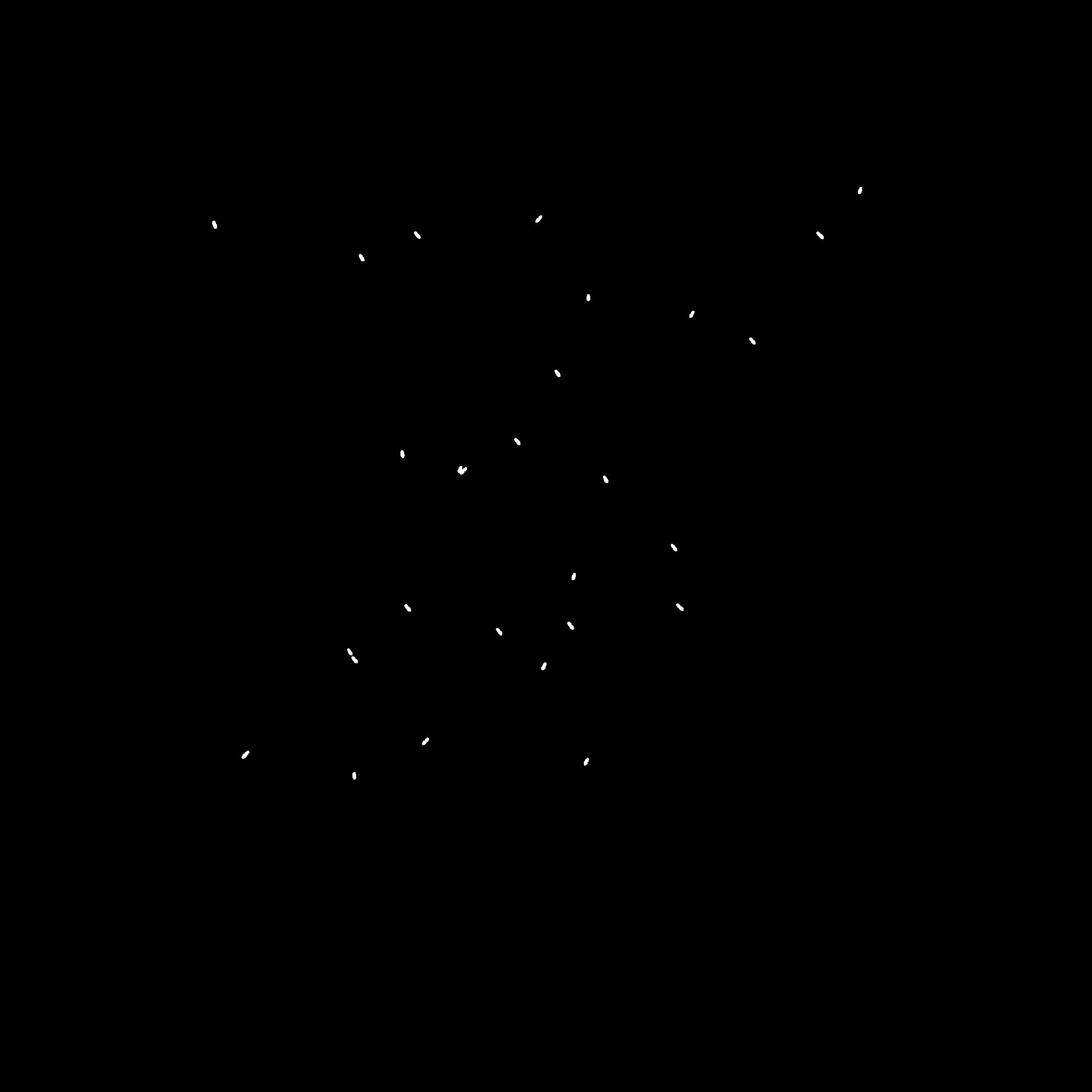

Supplement: S1 File — (ZIP) [file pone.0132101.s003.zip › ORsrc/nonortho/simu028/camx/imx122.jpg]

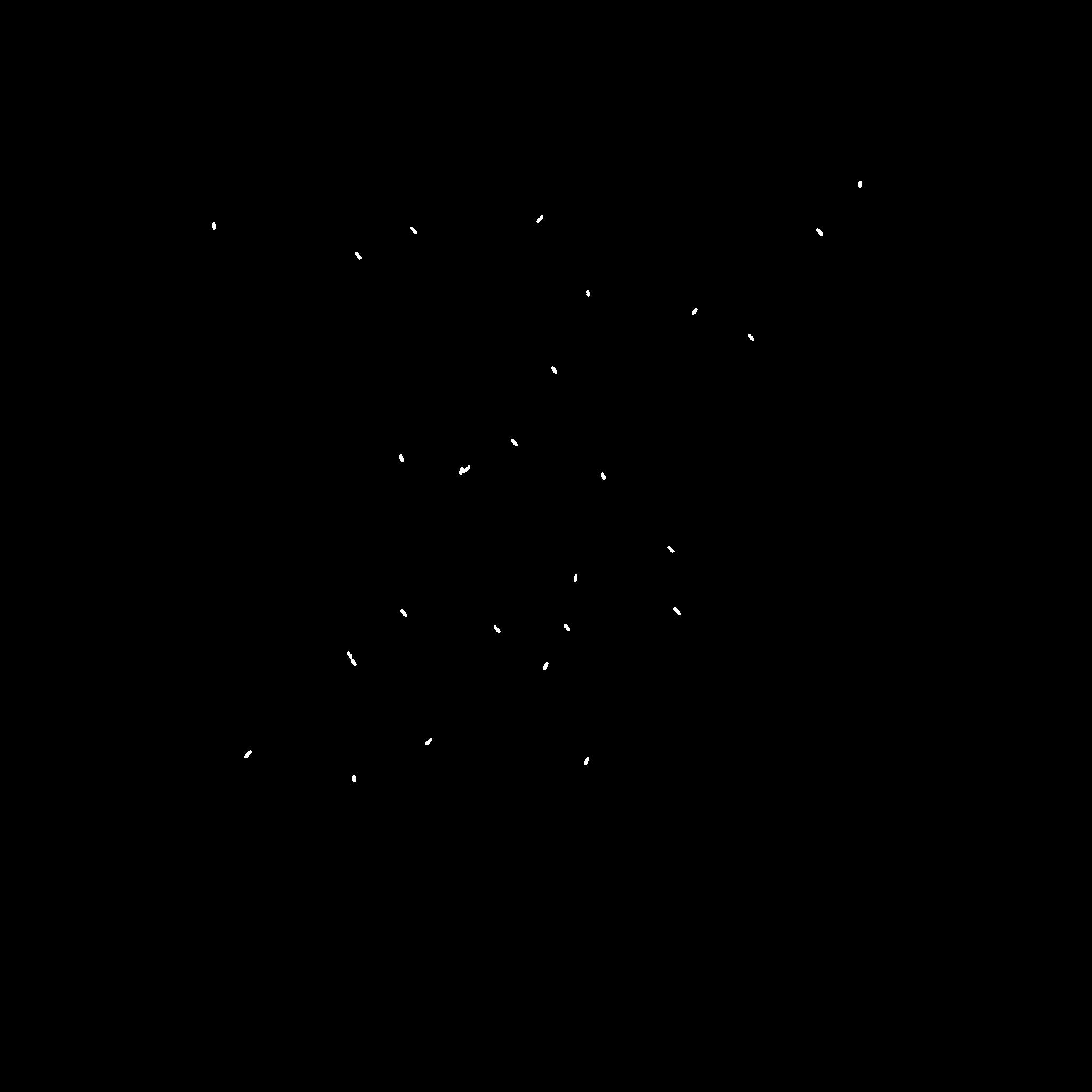

Supplement: S1 File — (ZIP) [file pone.0132101.s003.zip › ORsrc/nonortho/simu028/camx/imx123.jpg]

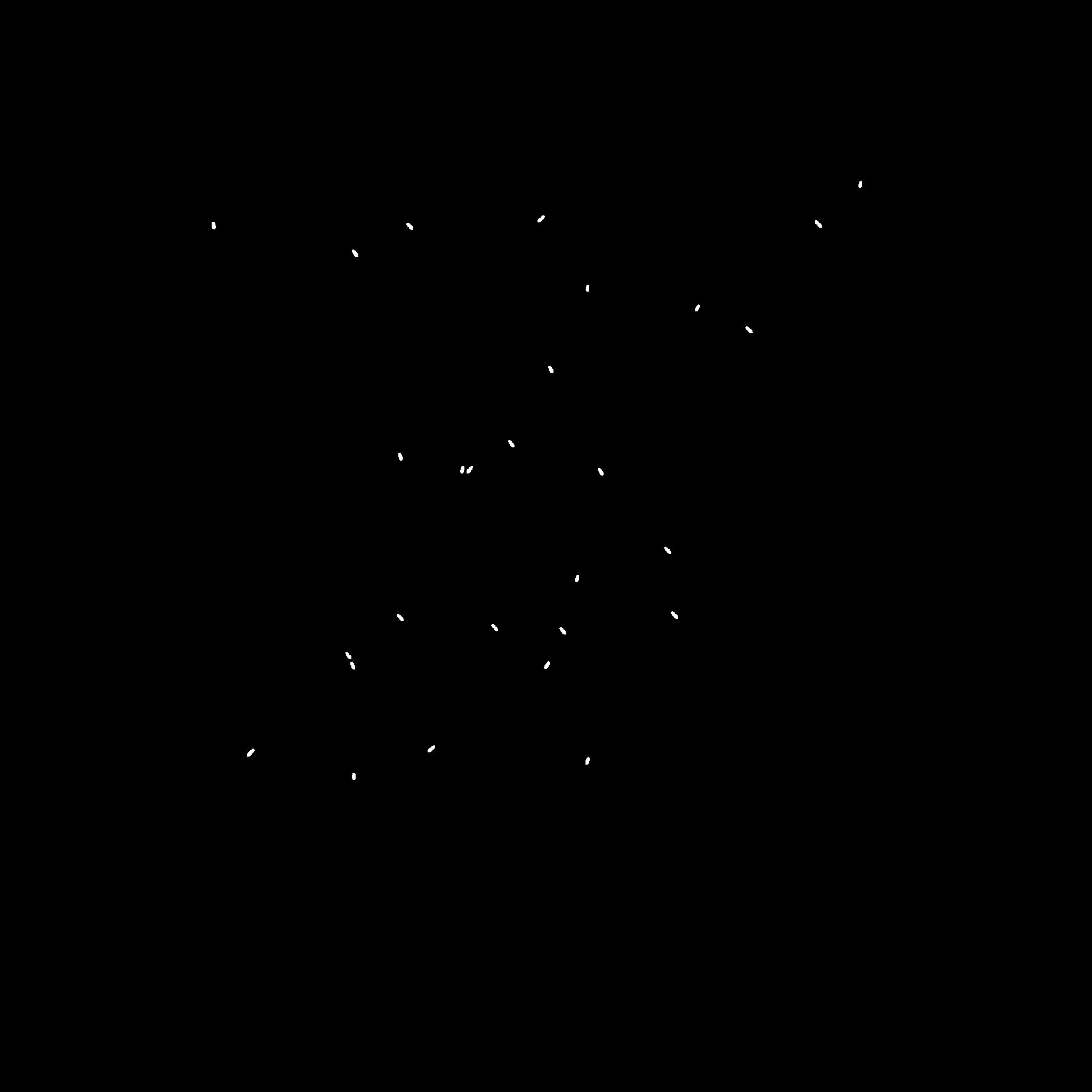

Supplement: S1 File — (ZIP) [file pone.0132101.s003.zip › ORsrc/nonortho/simu028/camx/imx124.jpg]

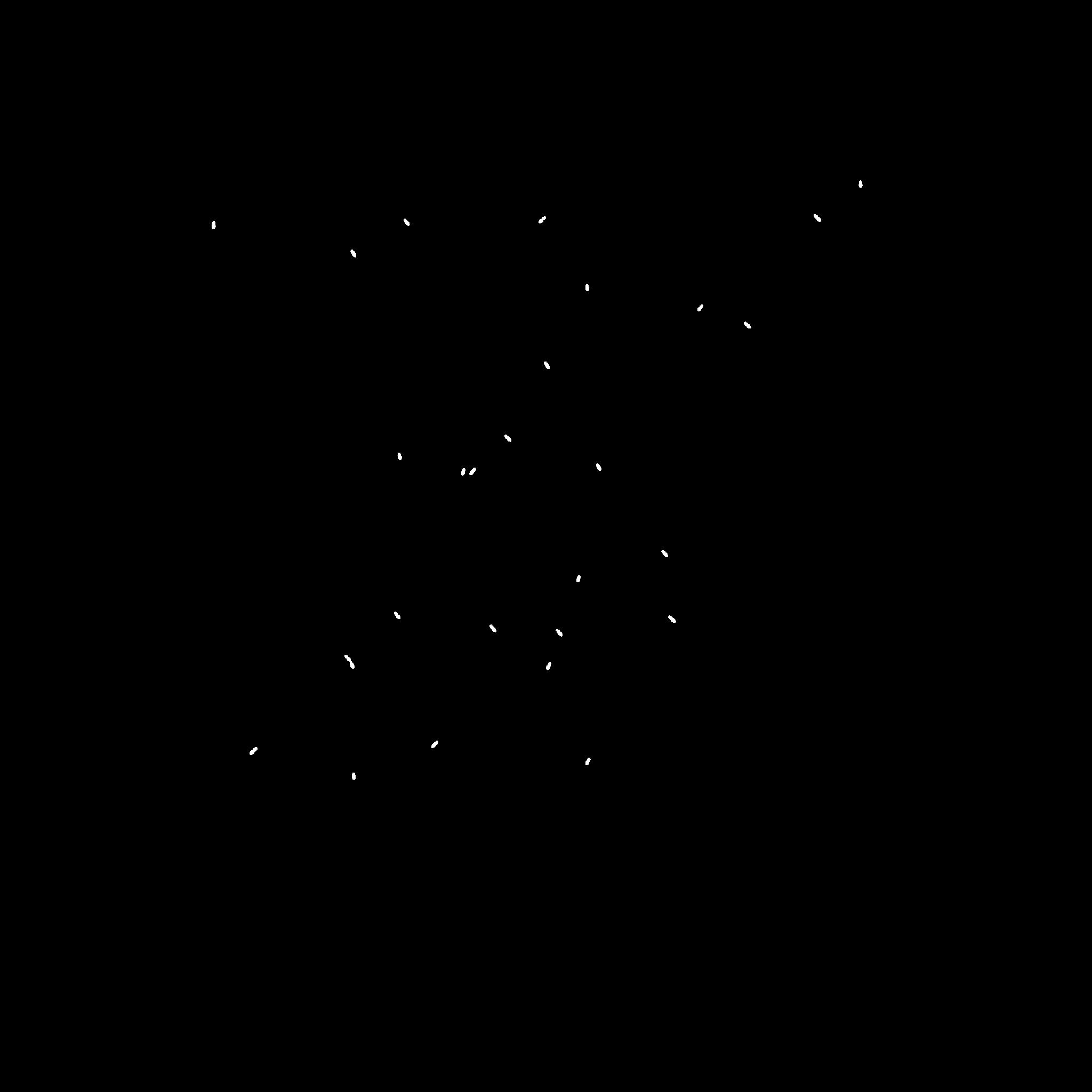

Supplement: S1 File — (ZIP) [file pone.0132101.s003.zip › ORsrc/nonortho/simu028/camx/imx125.jpg]

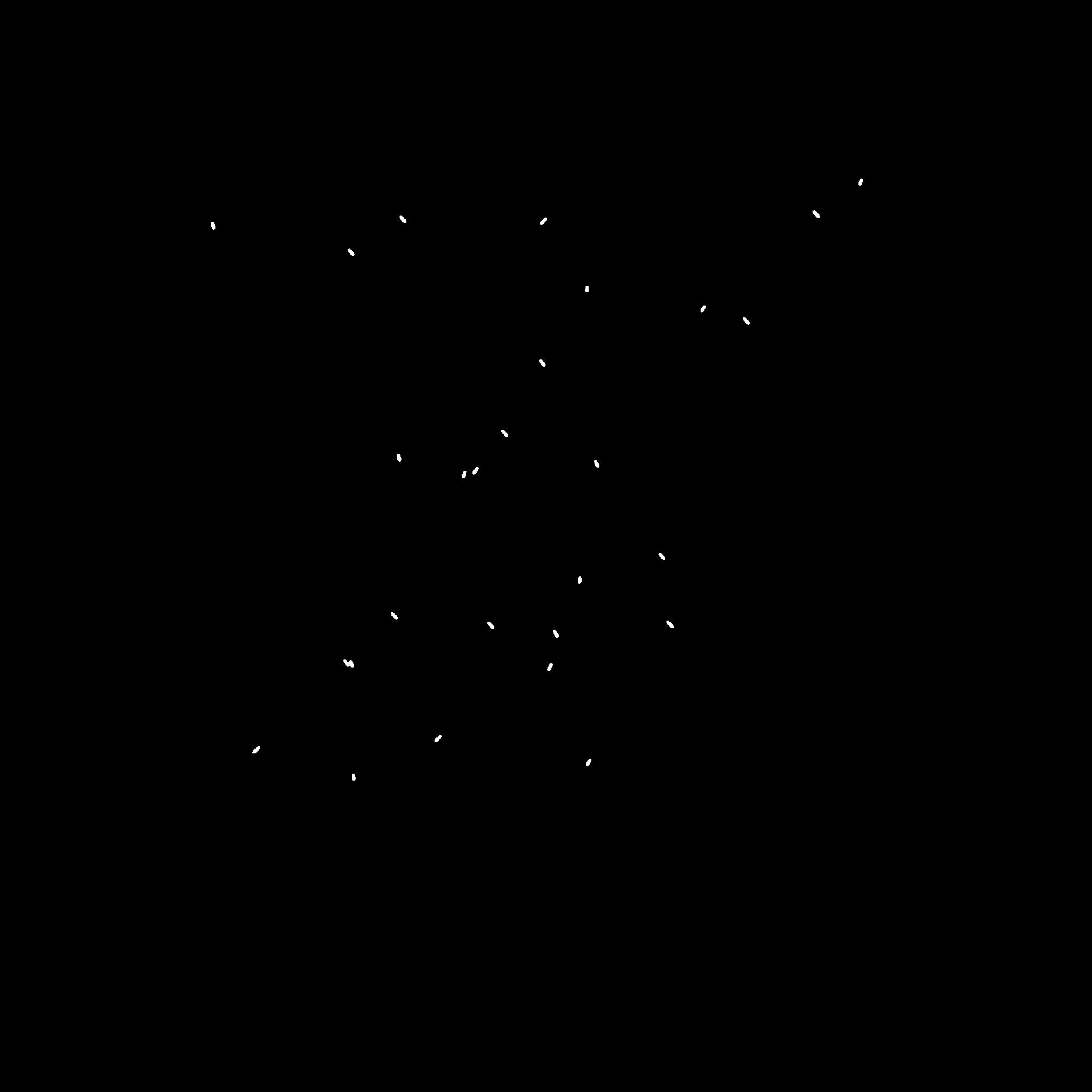

Supplement: S1 File — (ZIP) [file pone.0132101.s003.zip › ORsrc/nonortho/simu028/camx/imx126.jpg]

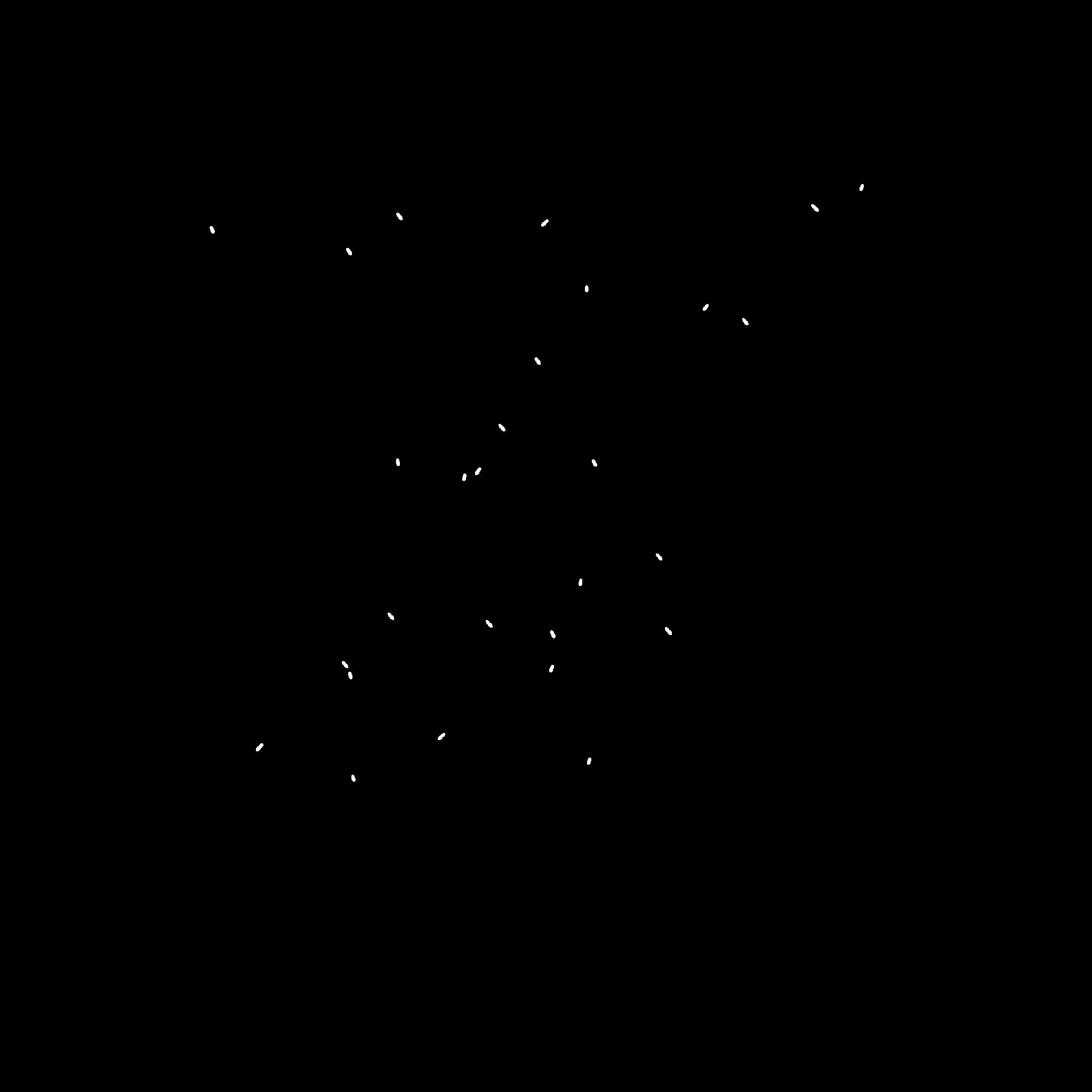

Supplement: S1 File — (ZIP) [file pone.0132101.s003.zip › ORsrc/nonortho/simu028/camx/imx127.jpg]

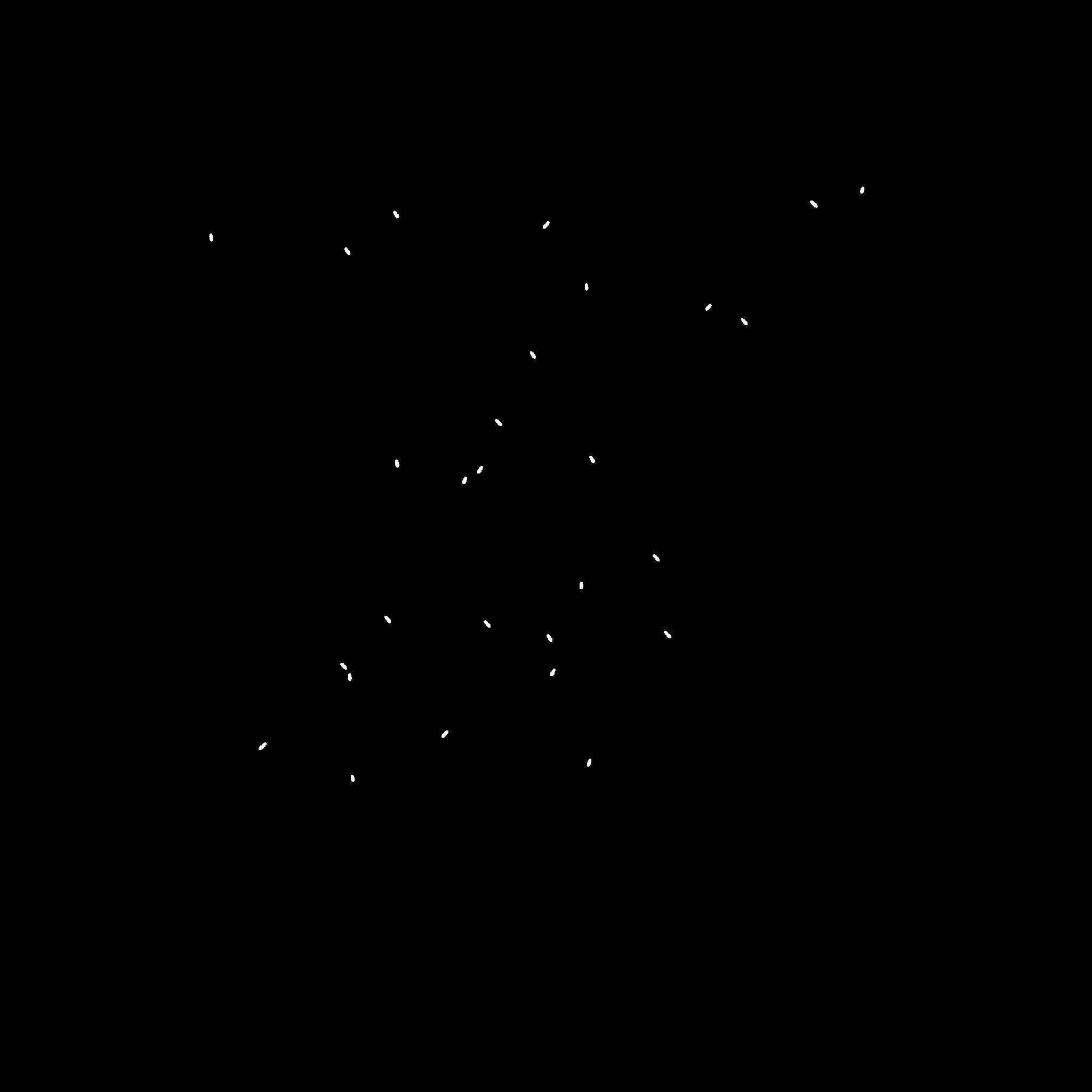

Supplement: S1 File — (ZIP) [file pone.0132101.s003.zip › ORsrc/nonortho/simu028/camx/imx128.jpg]

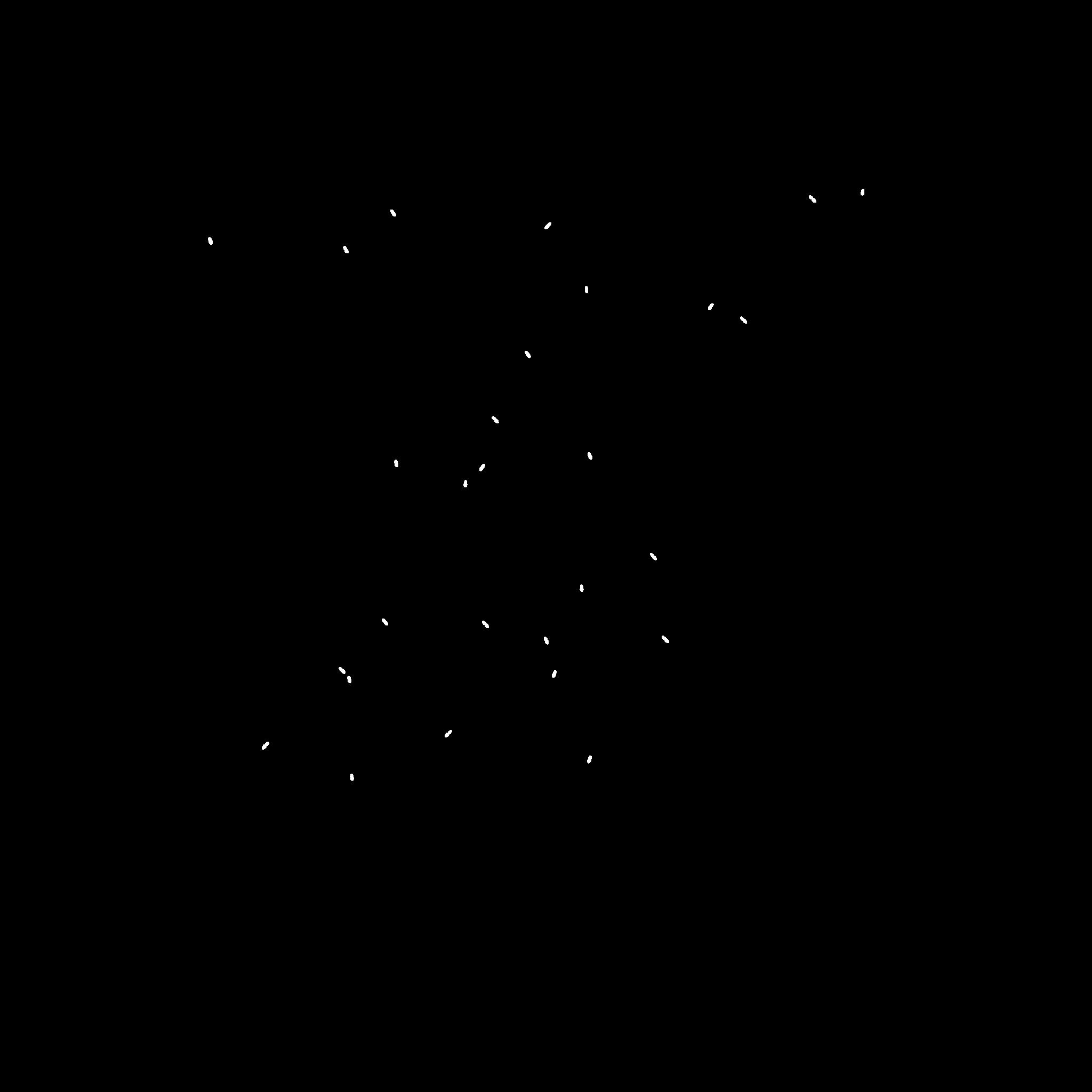

Supplement: S1 File — (ZIP) [file pone.0132101.s003.zip › ORsrc/nonortho/simu028/camx/imx129.jpg]

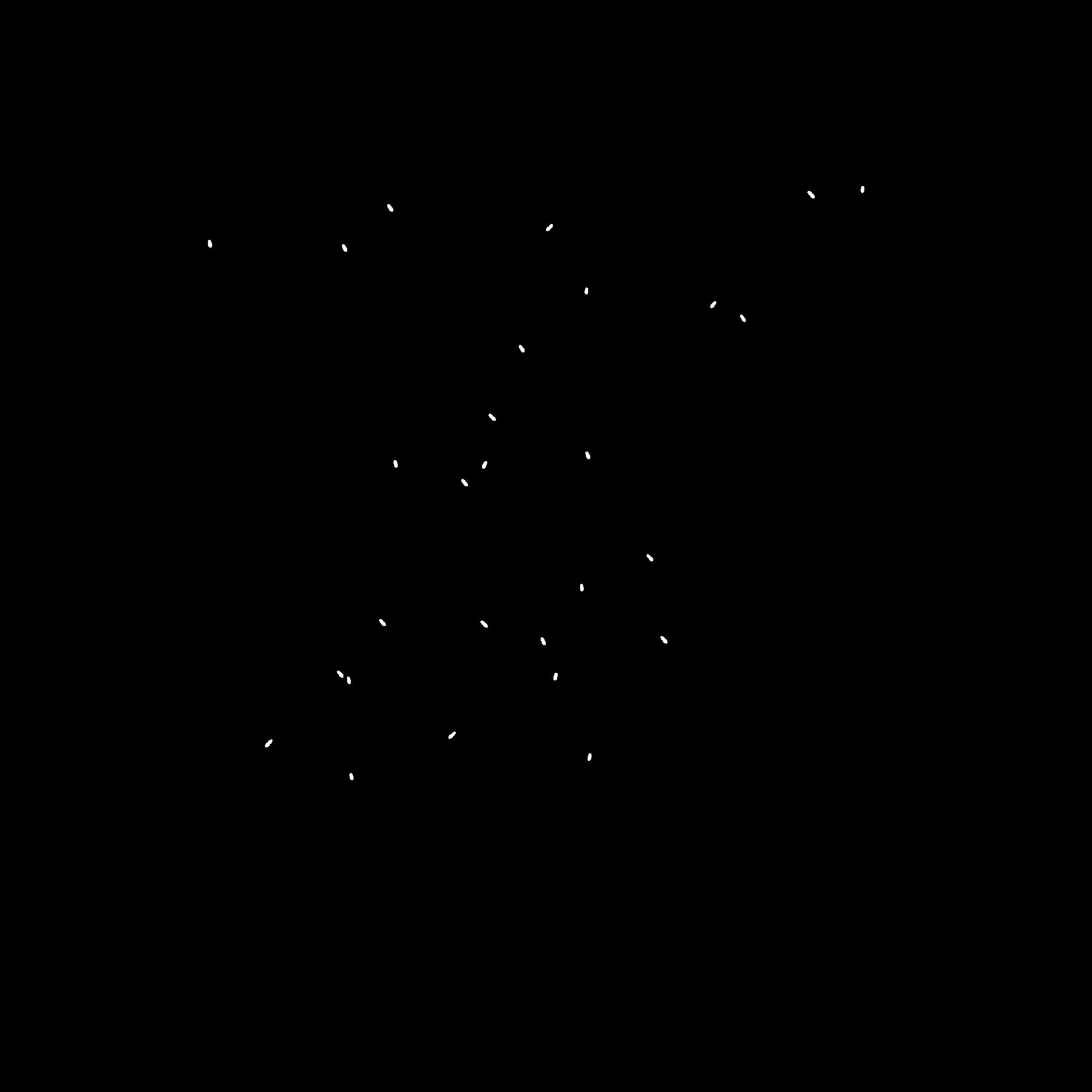

Supplement: S1 File — (ZIP) [file pone.0132101.s003.zip › ORsrc/nonortho/simu028/camx/imx130.jpg]

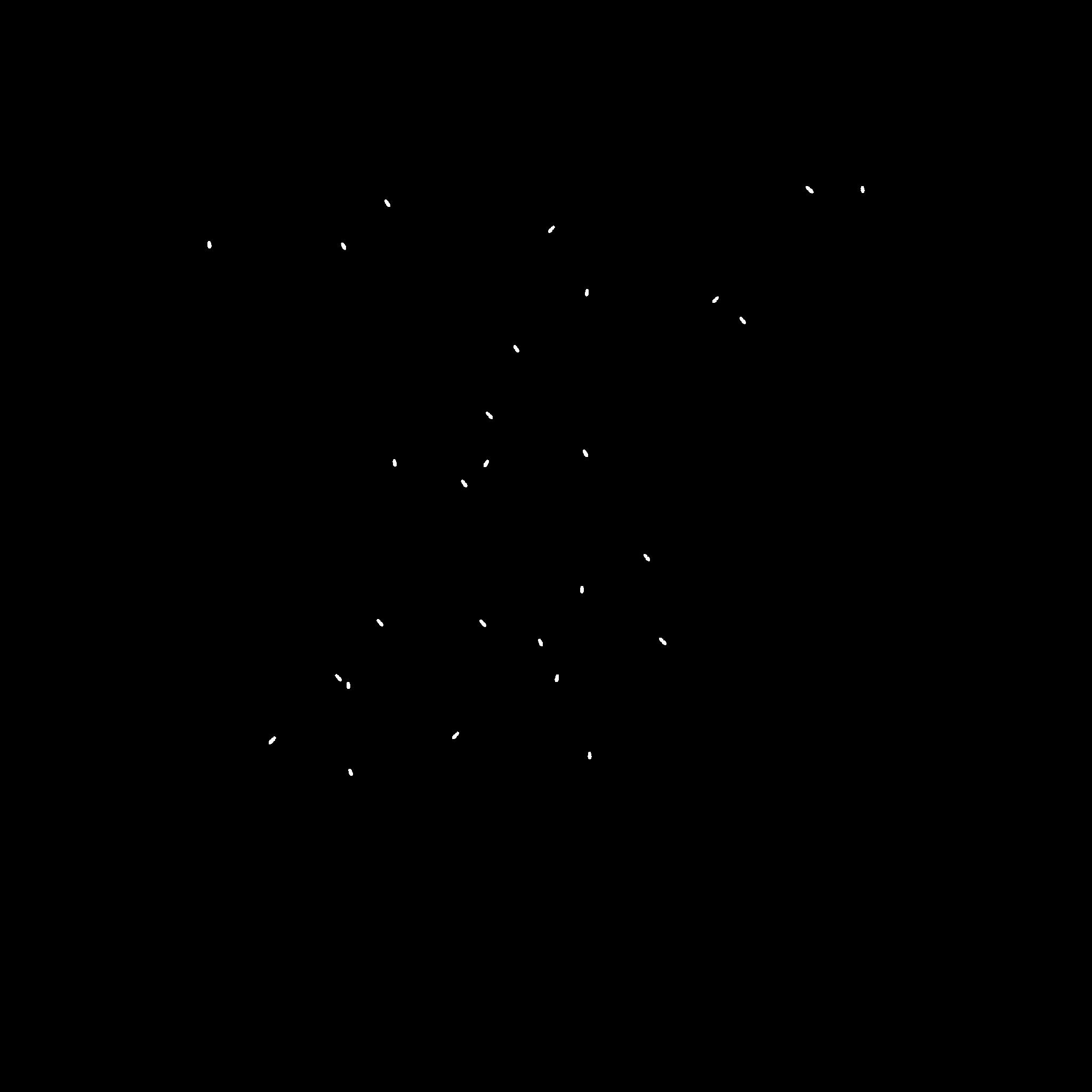

Supplement: S1 File — (ZIP) [file pone.0132101.s003.zip › ORsrc/nonortho/simu028/camx/imx131.jpg]

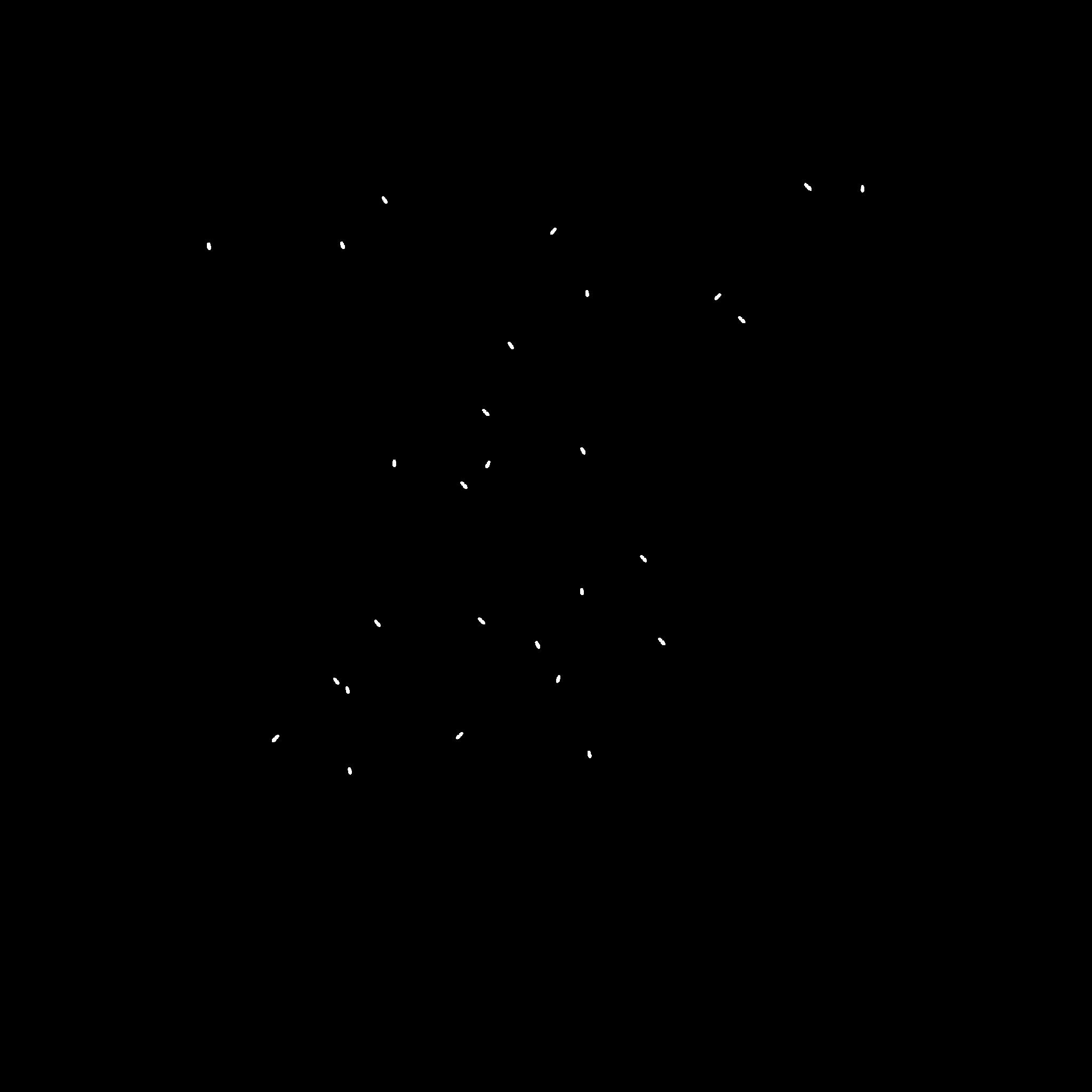

Supplement: S1 File — (ZIP) [file pone.0132101.s003.zip › ORsrc/nonortho/simu028/camx/imx132.jpg]

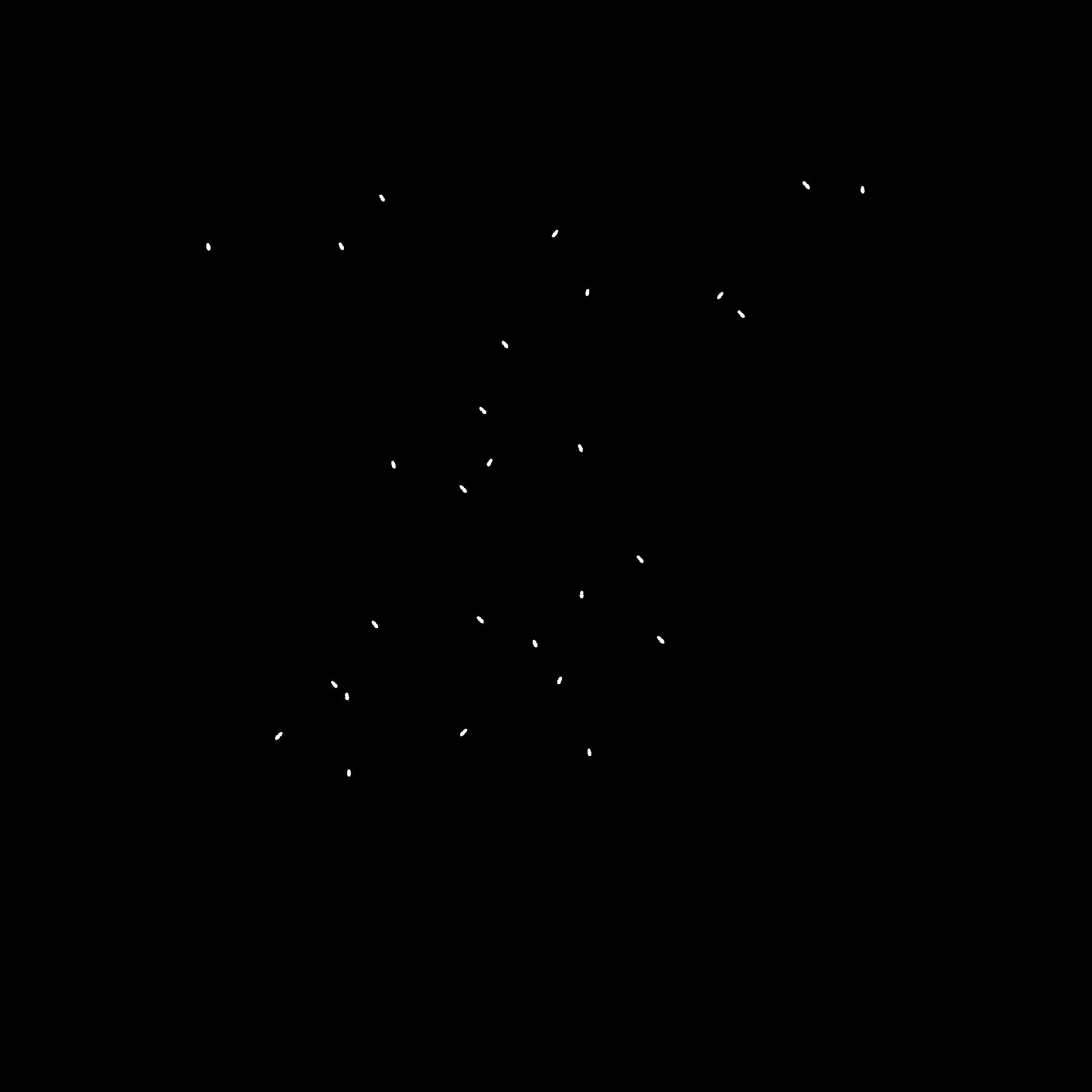

Supplement: S1 File — (ZIP) [file pone.0132101.s003.zip › ORsrc/nonortho/simu028/camx/imx133.jpg]

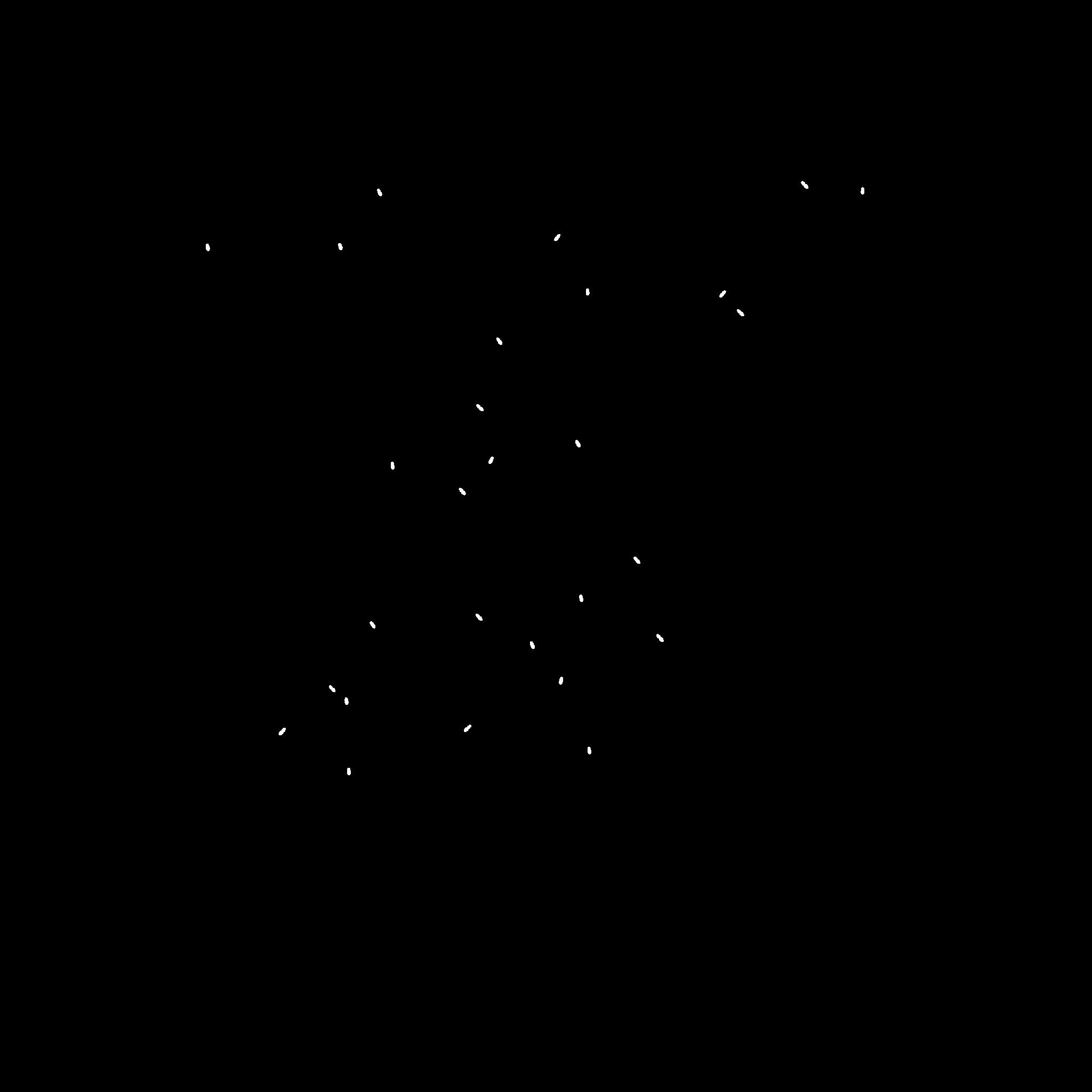

Supplement: S1 File — (ZIP) [file pone.0132101.s003.zip › ORsrc/nonortho/simu028/camx/imx134.jpg]

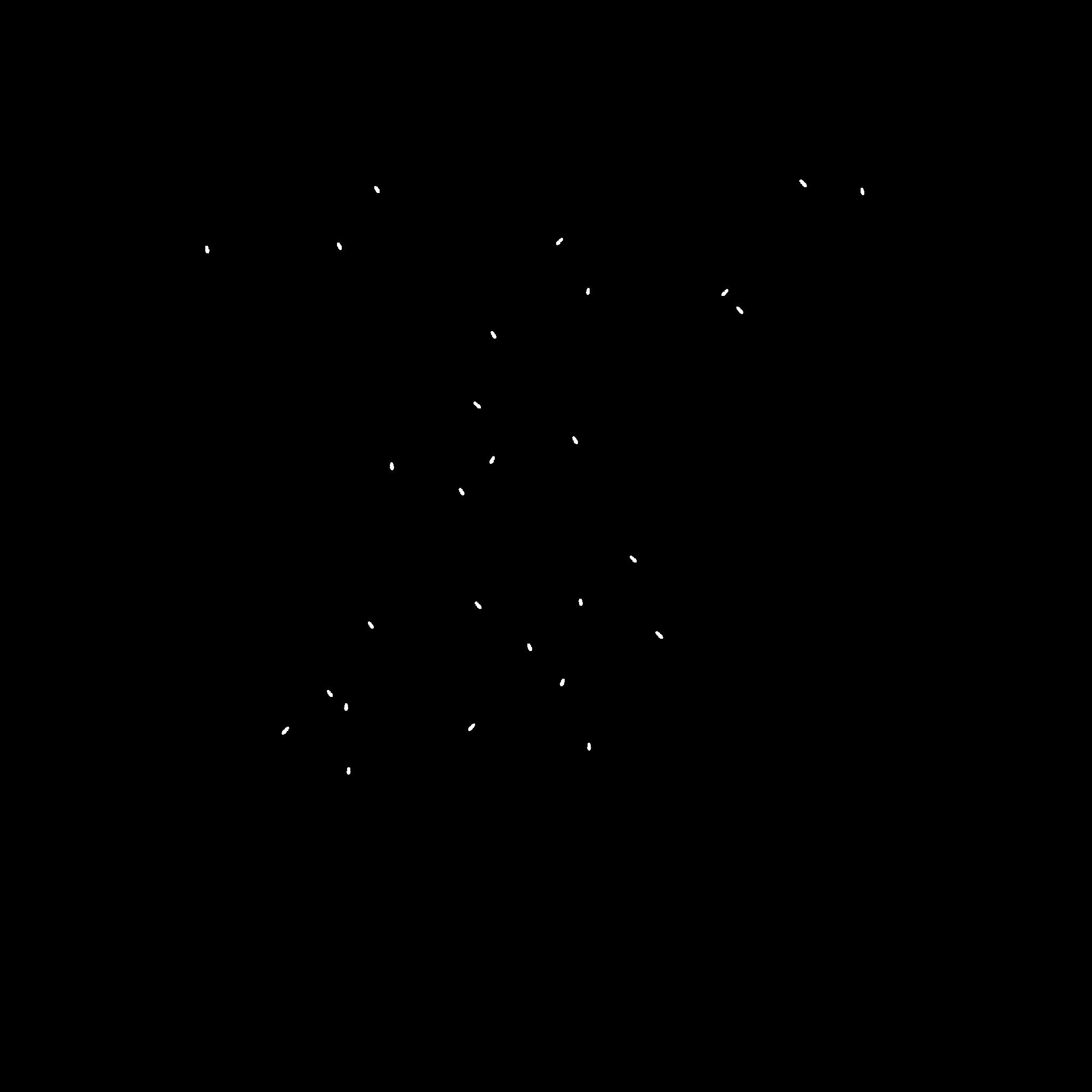

Supplement: S1 File — (ZIP) [file pone.0132101.s003.zip › ORsrc/nonortho/simu028/camx/imx135.jpg]

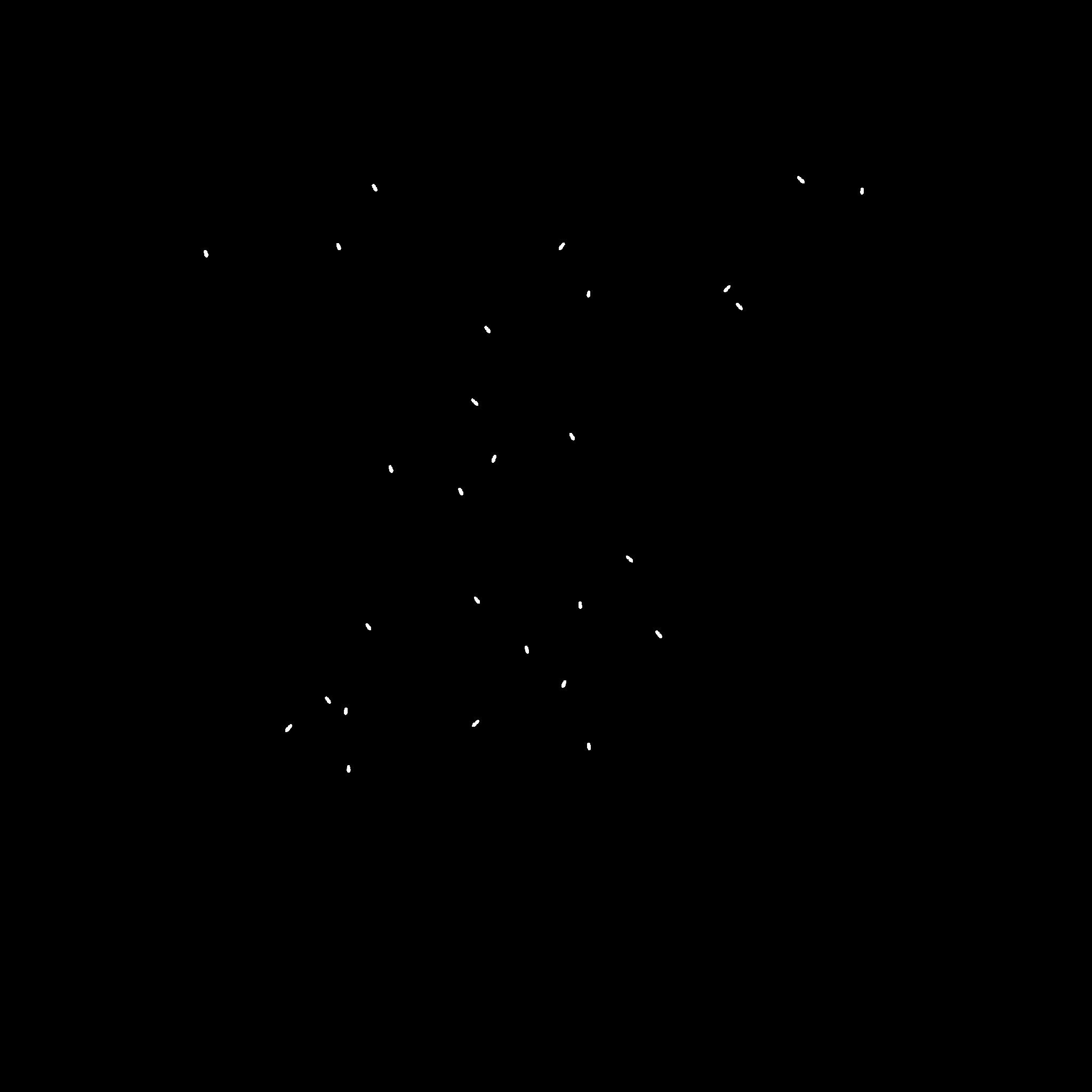

Supplement: S1 File — (ZIP) [file pone.0132101.s003.zip › ORsrc/nonortho/simu028/camx/imx136.jpg]

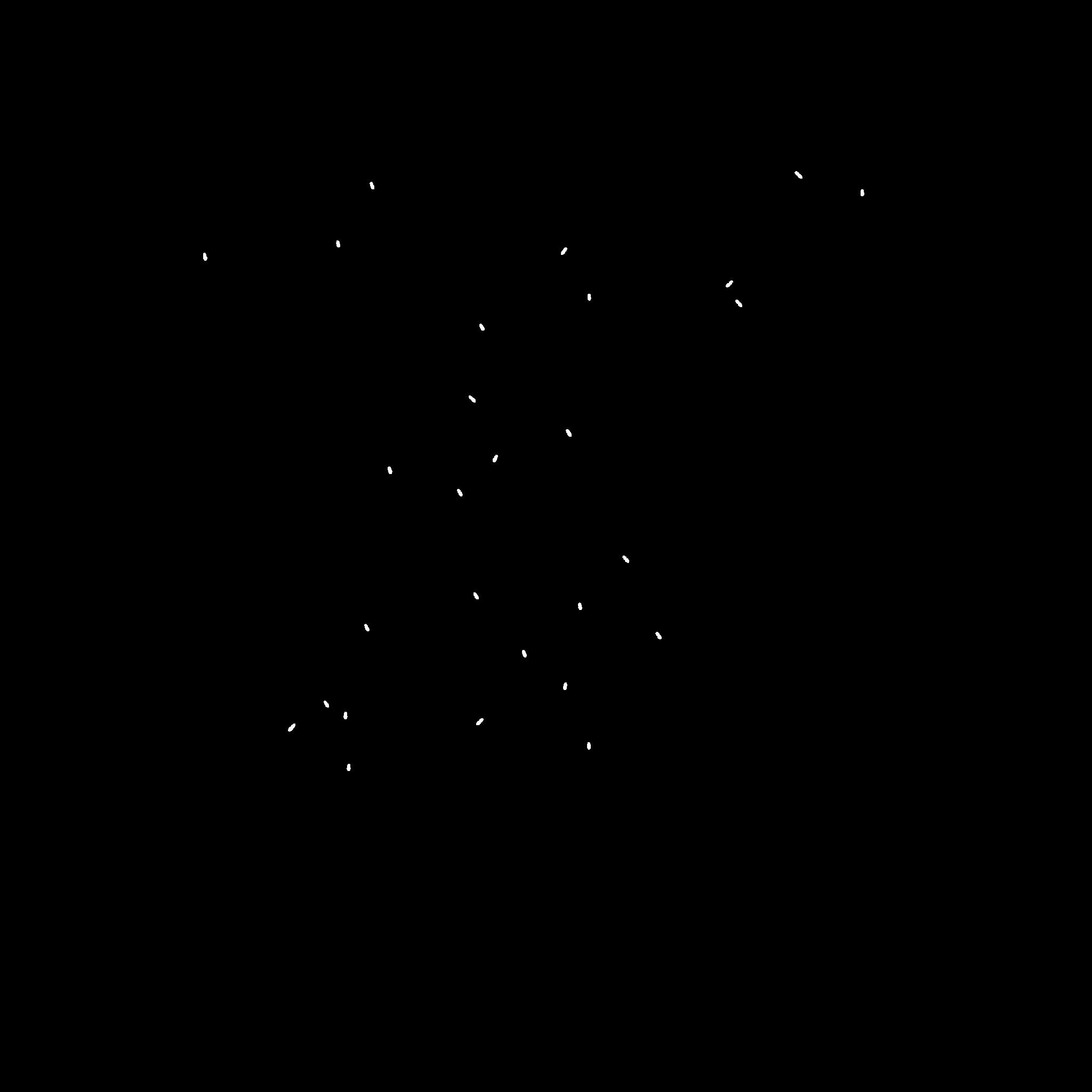

Supplement: S1 File — (ZIP) [file pone.0132101.s003.zip › ORsrc/nonortho/simu028/camx/imx137.jpg]

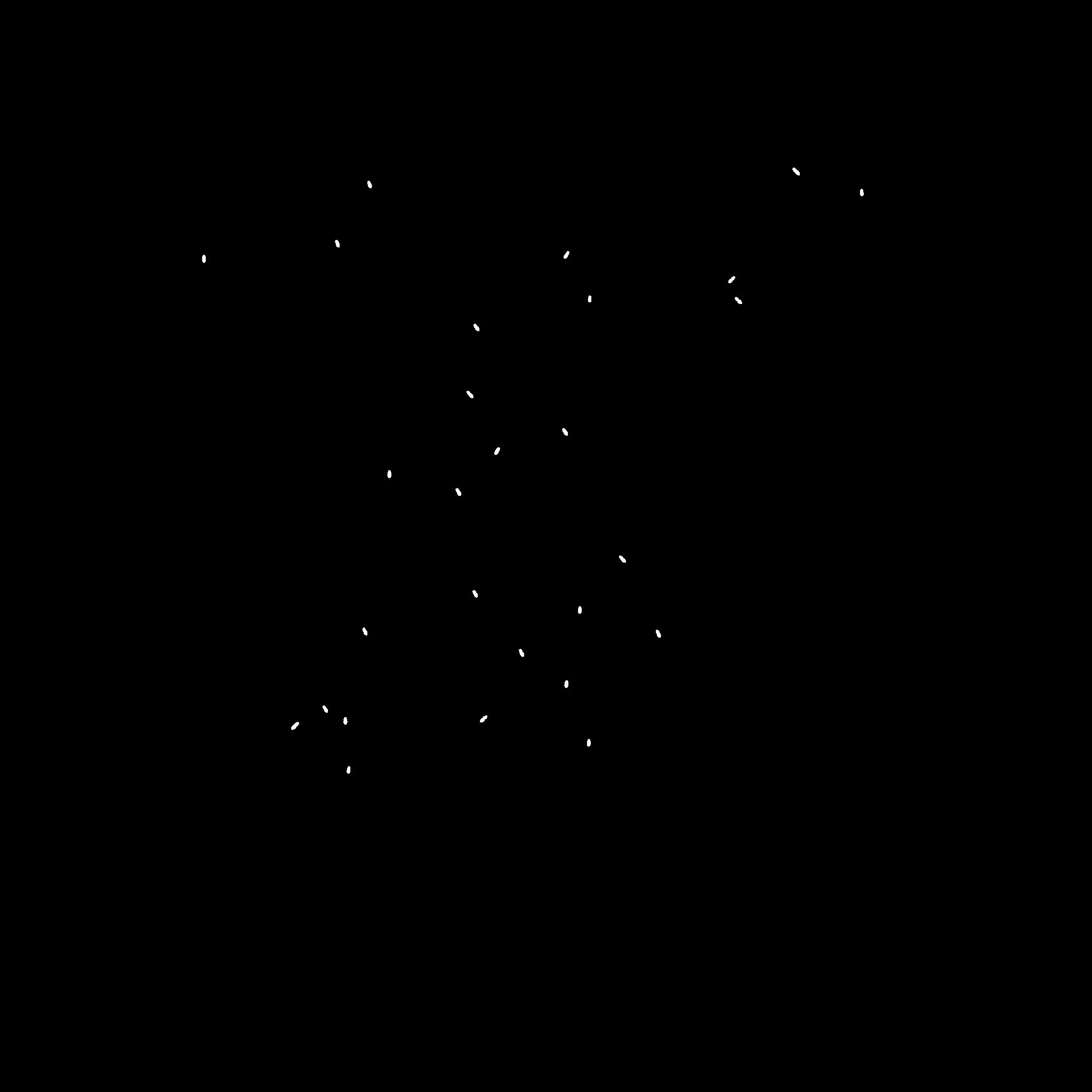

Supplement: S1 File — (ZIP) [file pone.0132101.s003.zip › ORsrc/nonortho/simu028/camx/imx138.jpg]

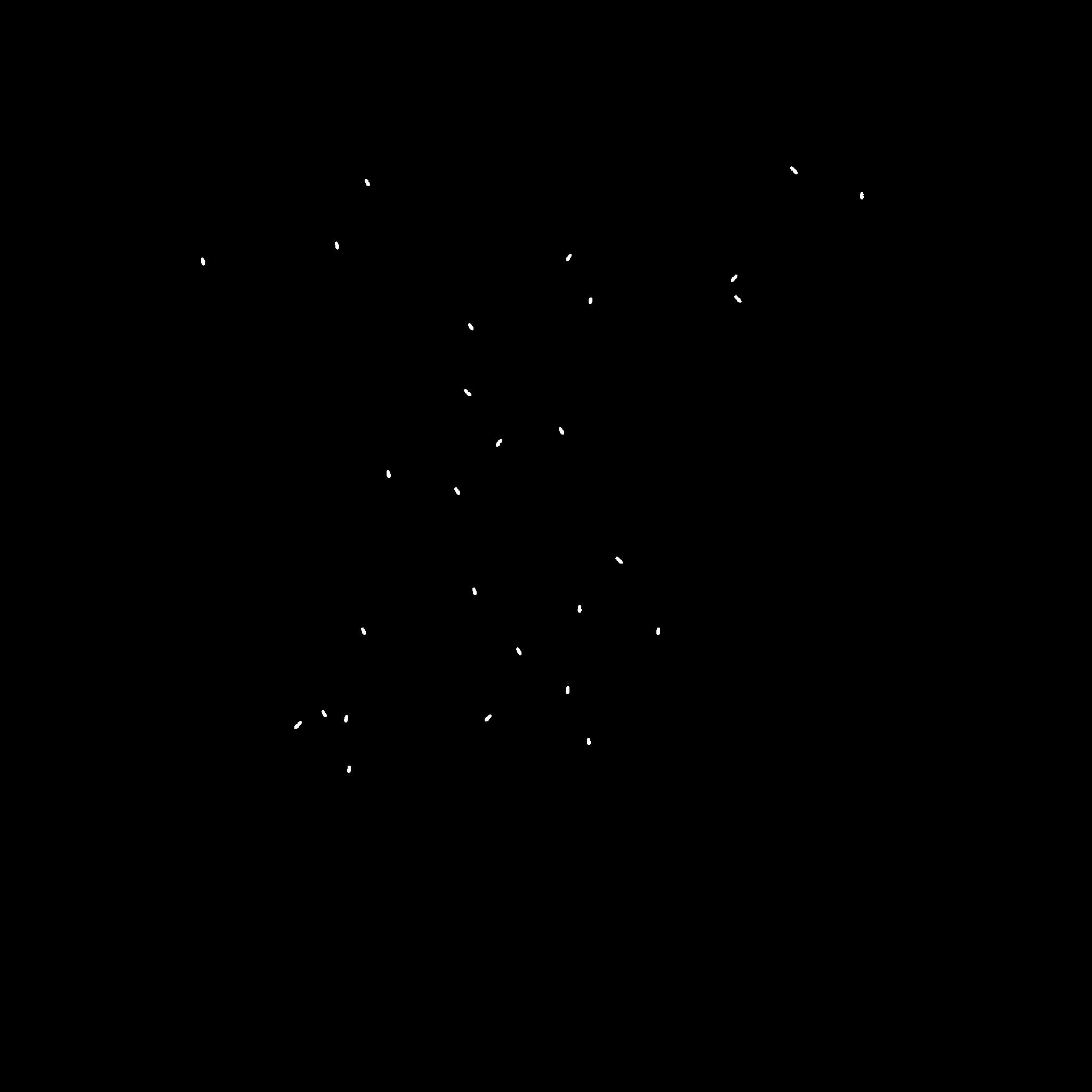

Supplement: S1 File — (ZIP) [file pone.0132101.s003.zip › ORsrc/nonortho/simu028/camx/imx139.jpg]

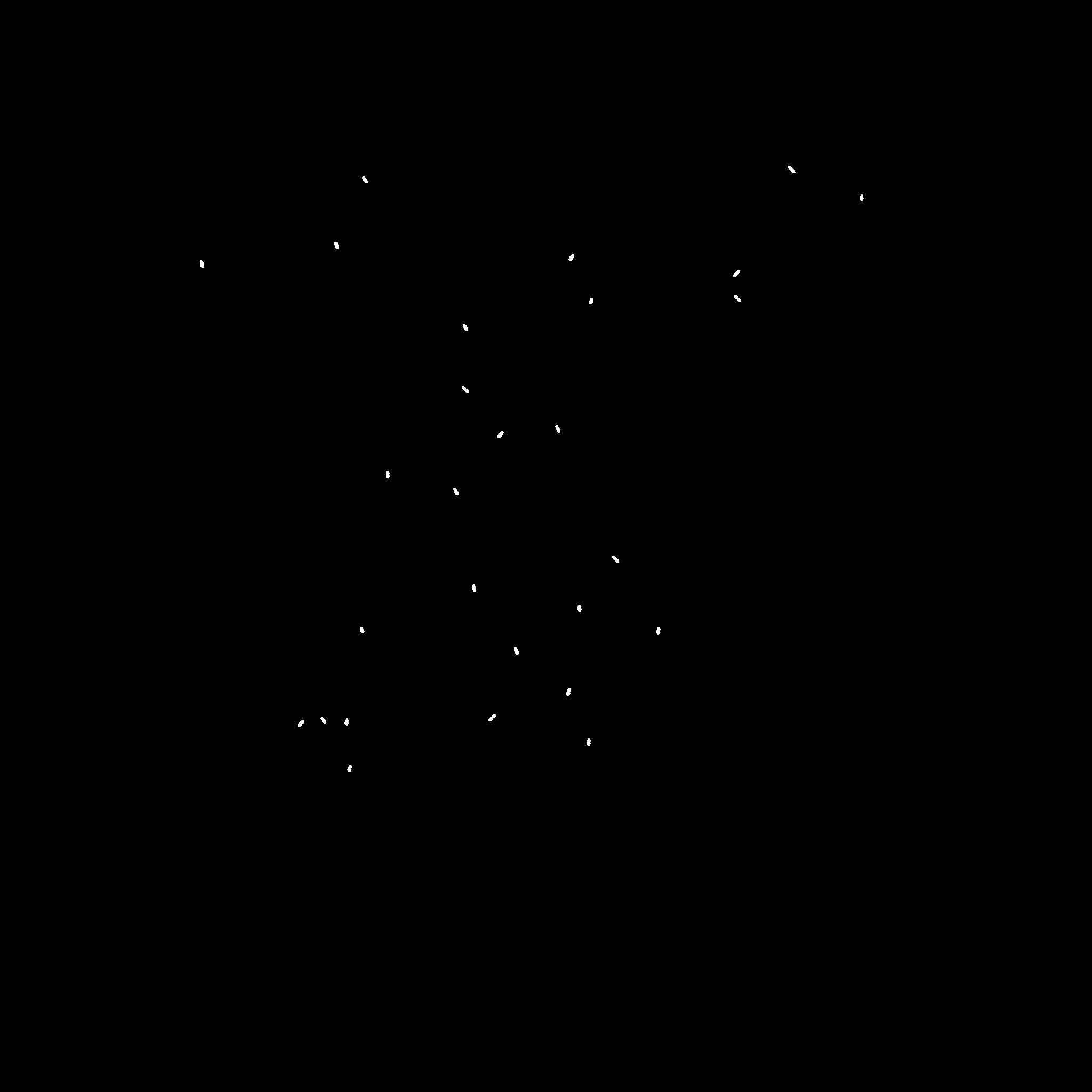

Supplement: S1 File — (ZIP) [file pone.0132101.s003.zip › ORsrc/nonortho/simu028/camx/imx140.jpg]

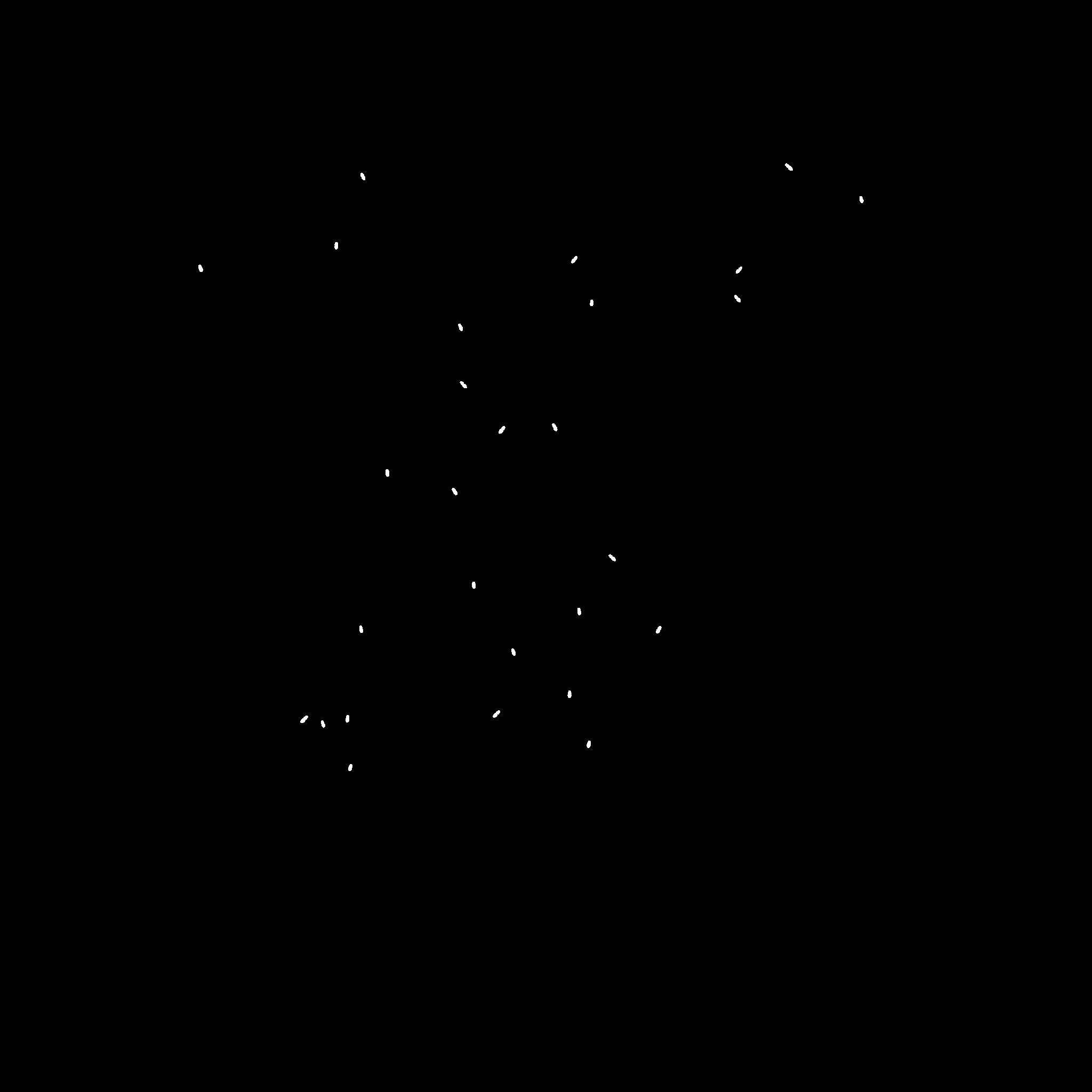

Supplement: S1 File — (ZIP) [file pone.0132101.s003.zip › ORsrc/nonortho/simu028/camx/imx141.jpg]

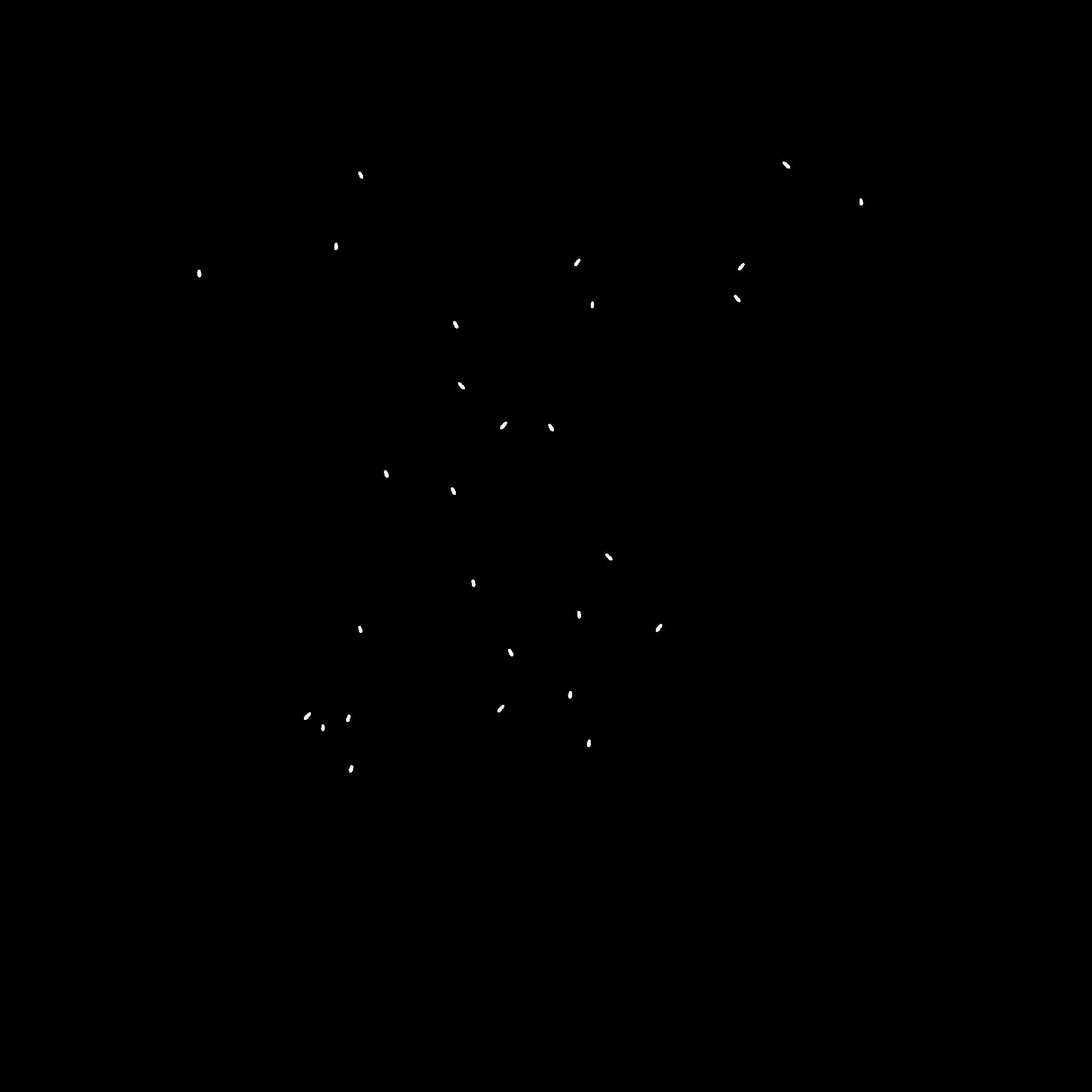

Supplement: S1 File — (ZIP) [file pone.0132101.s003.zip › ORsrc/nonortho/simu028/camx/imx142.jpg]

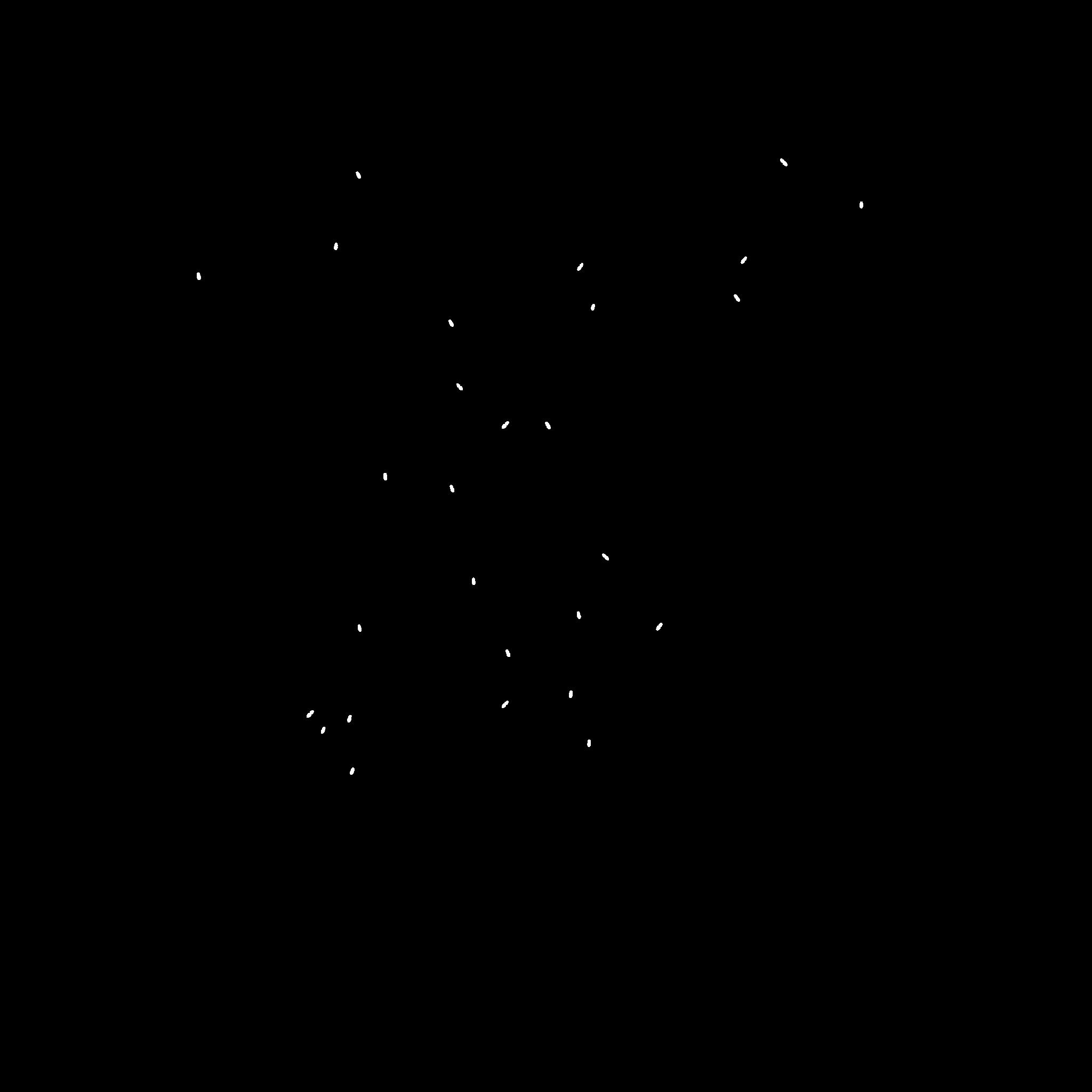

Supplement: S1 File — (ZIP) [file pone.0132101.s003.zip › ORsrc/nonortho/simu028/camx/imx143.jpg]

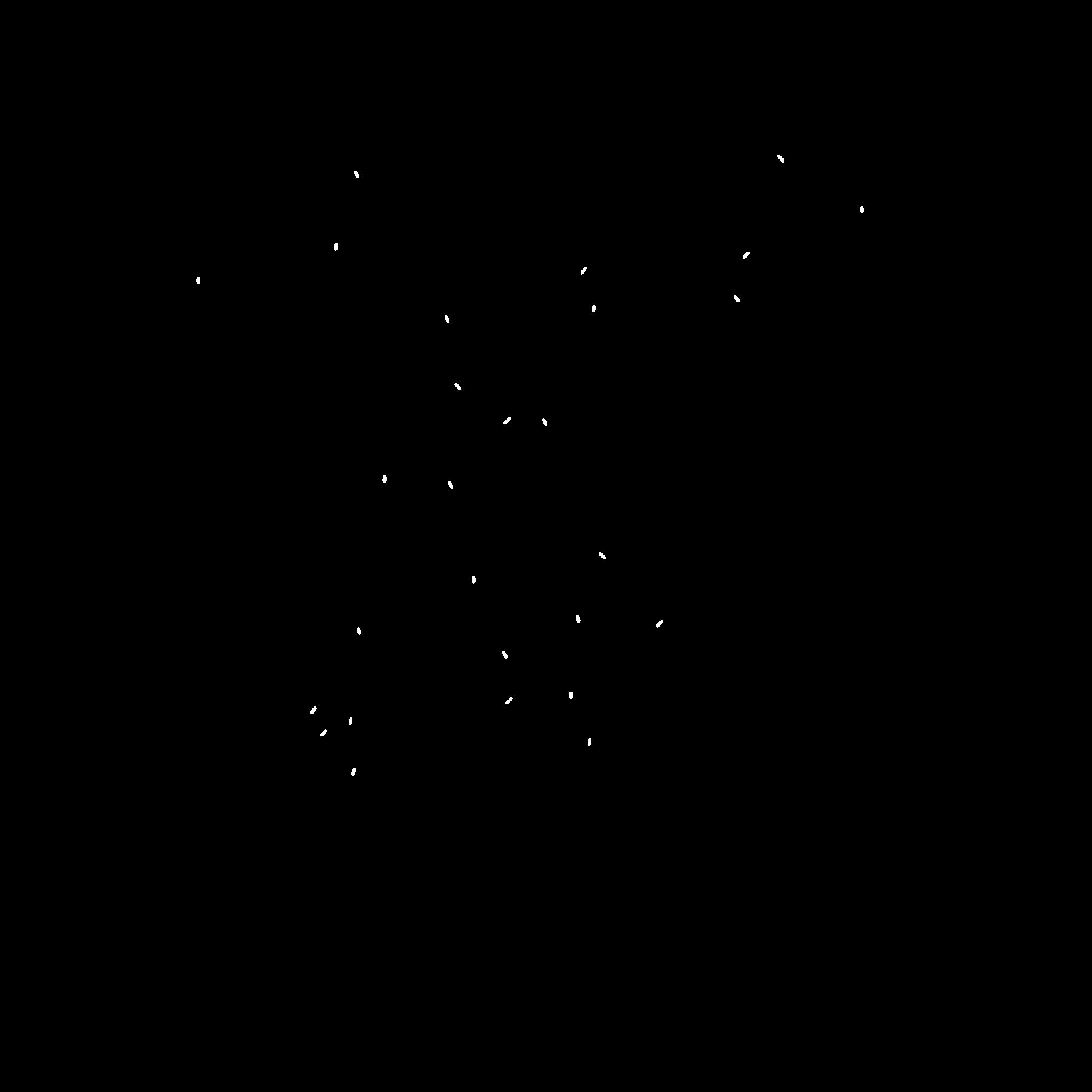

Supplement: S1 File — (ZIP) [file pone.0132101.s003.zip › ORsrc/nonortho/simu028/camx/imx144.jpg]

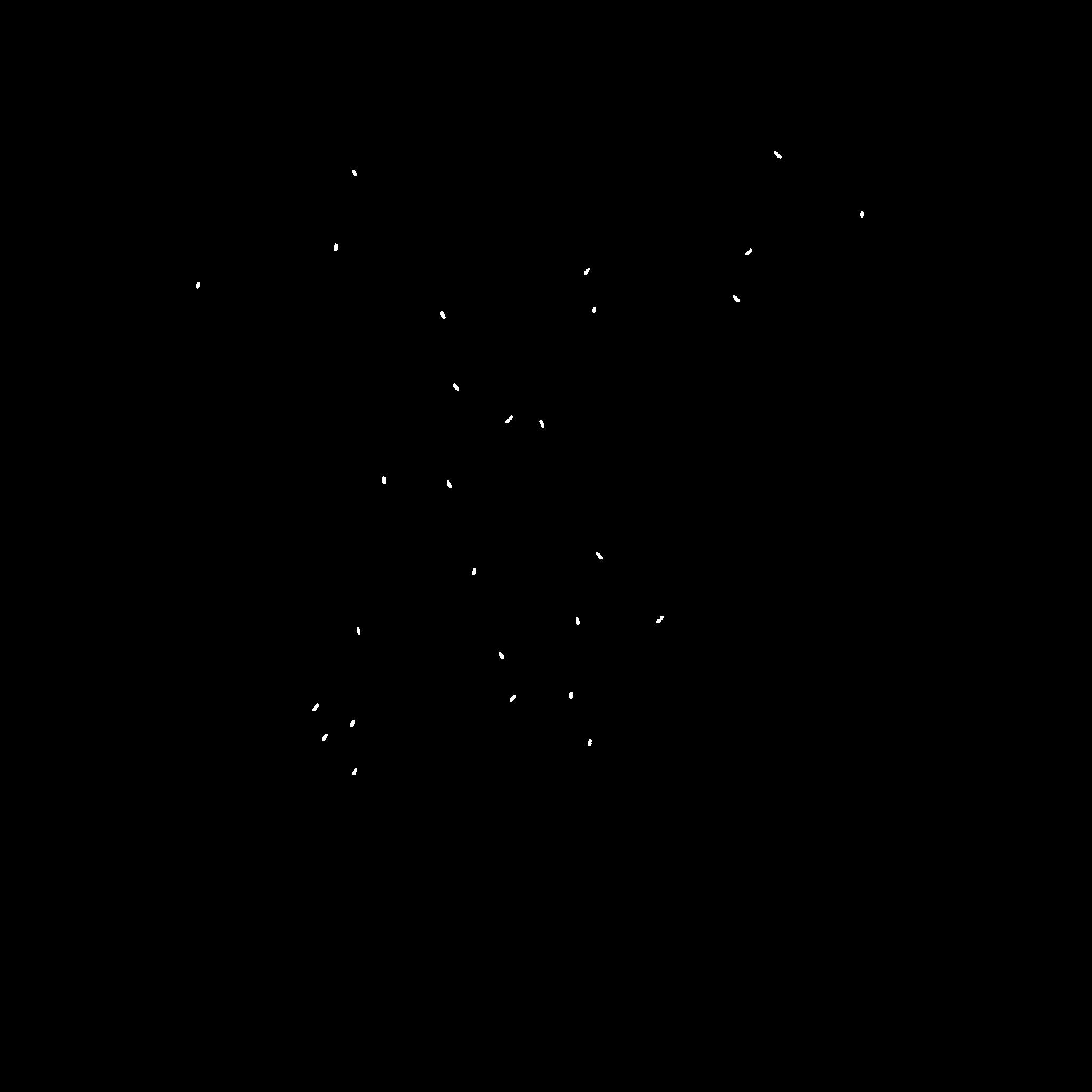

Supplement: S1 File — (ZIP) [file pone.0132101.s003.zip › ORsrc/nonortho/simu028/camx/imx145.jpg]

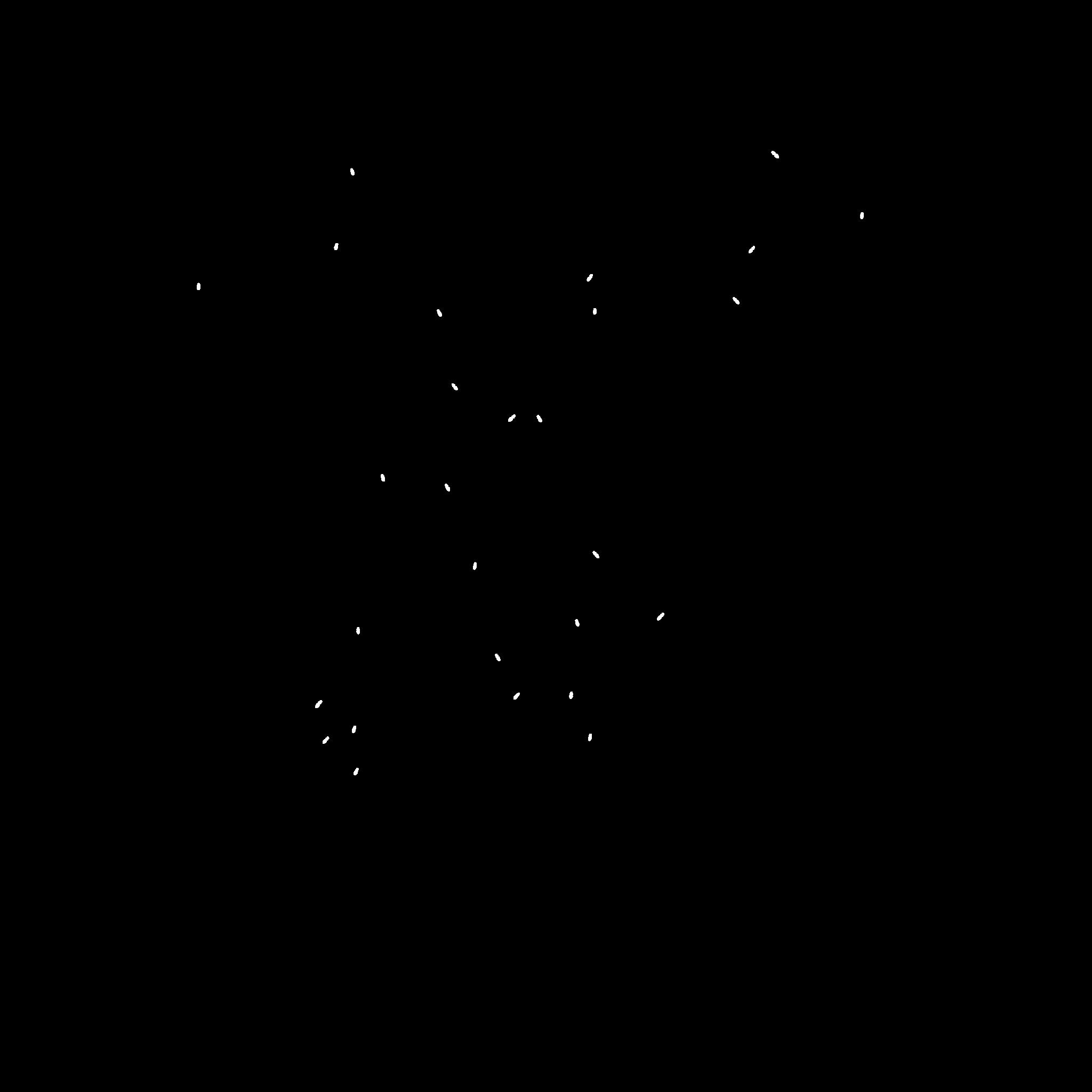

Supplement: S1 File — (ZIP) [file pone.0132101.s003.zip › ORsrc/nonortho/simu028/camx/imx146.jpg]

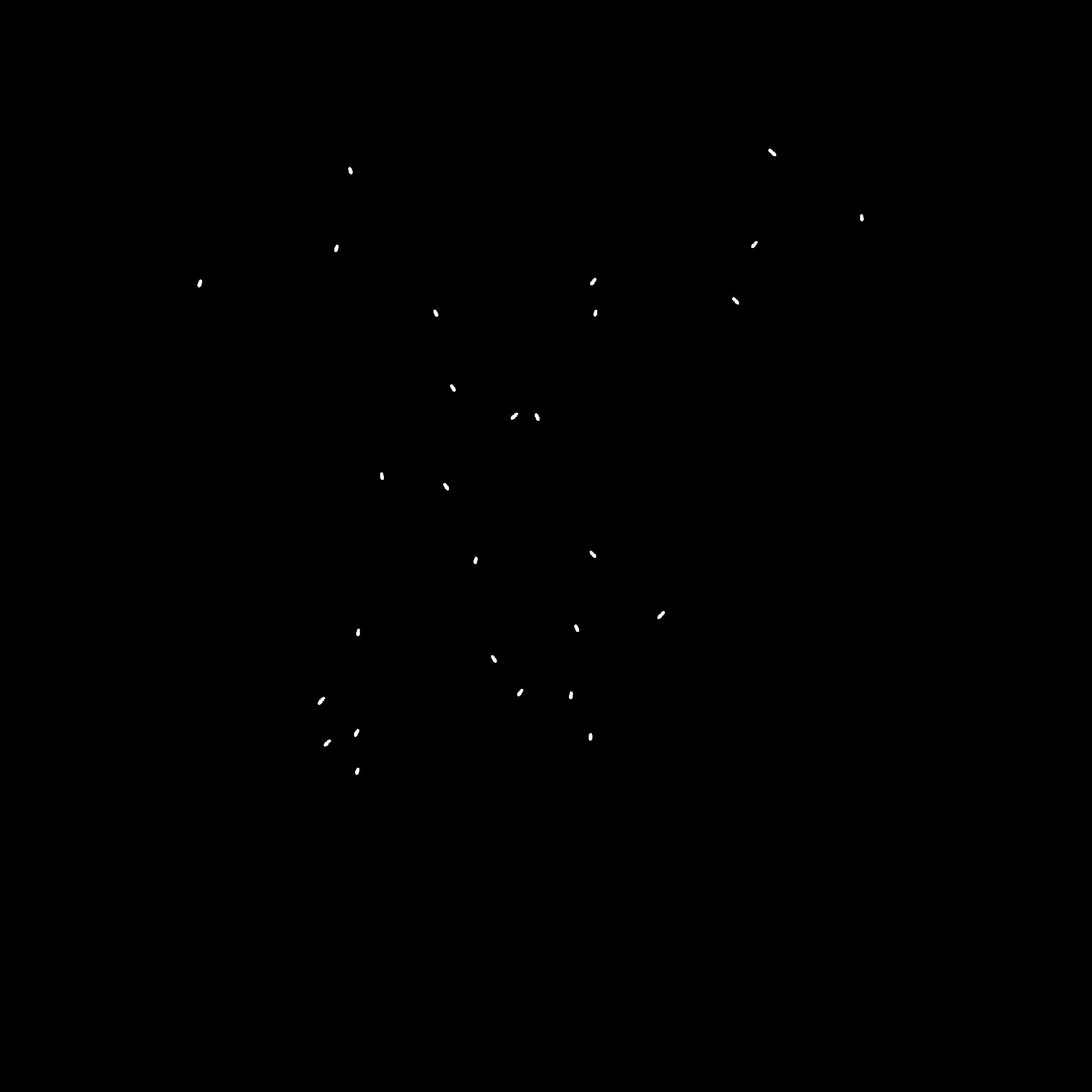

Supplement: S1 File — (ZIP) [file pone.0132101.s003.zip › ORsrc/nonortho/simu028/camx/imx147.jpg]

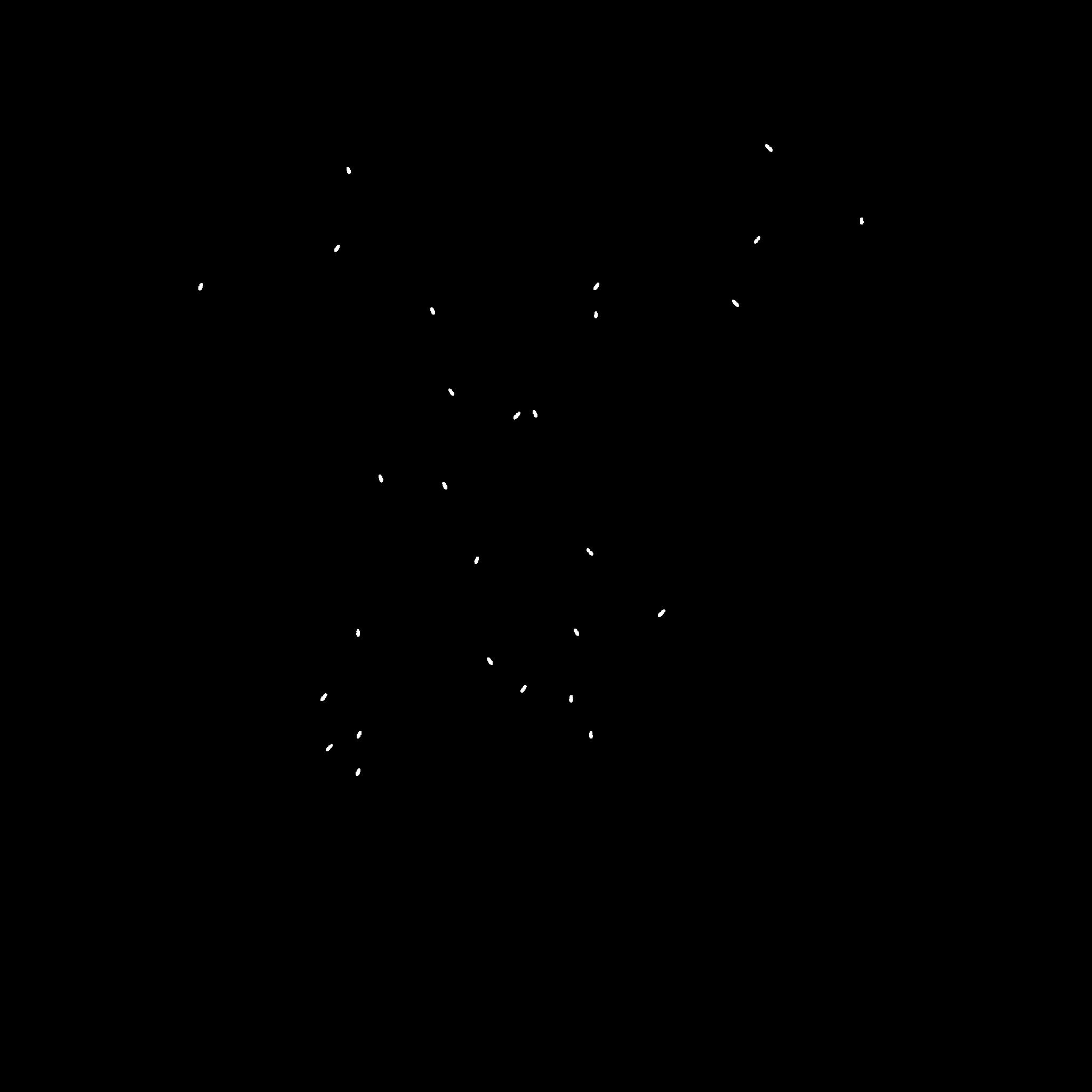

Supplement: S1 File — (ZIP) [file pone.0132101.s003.zip › ORsrc/nonortho/simu028/camx/imx148.jpg]

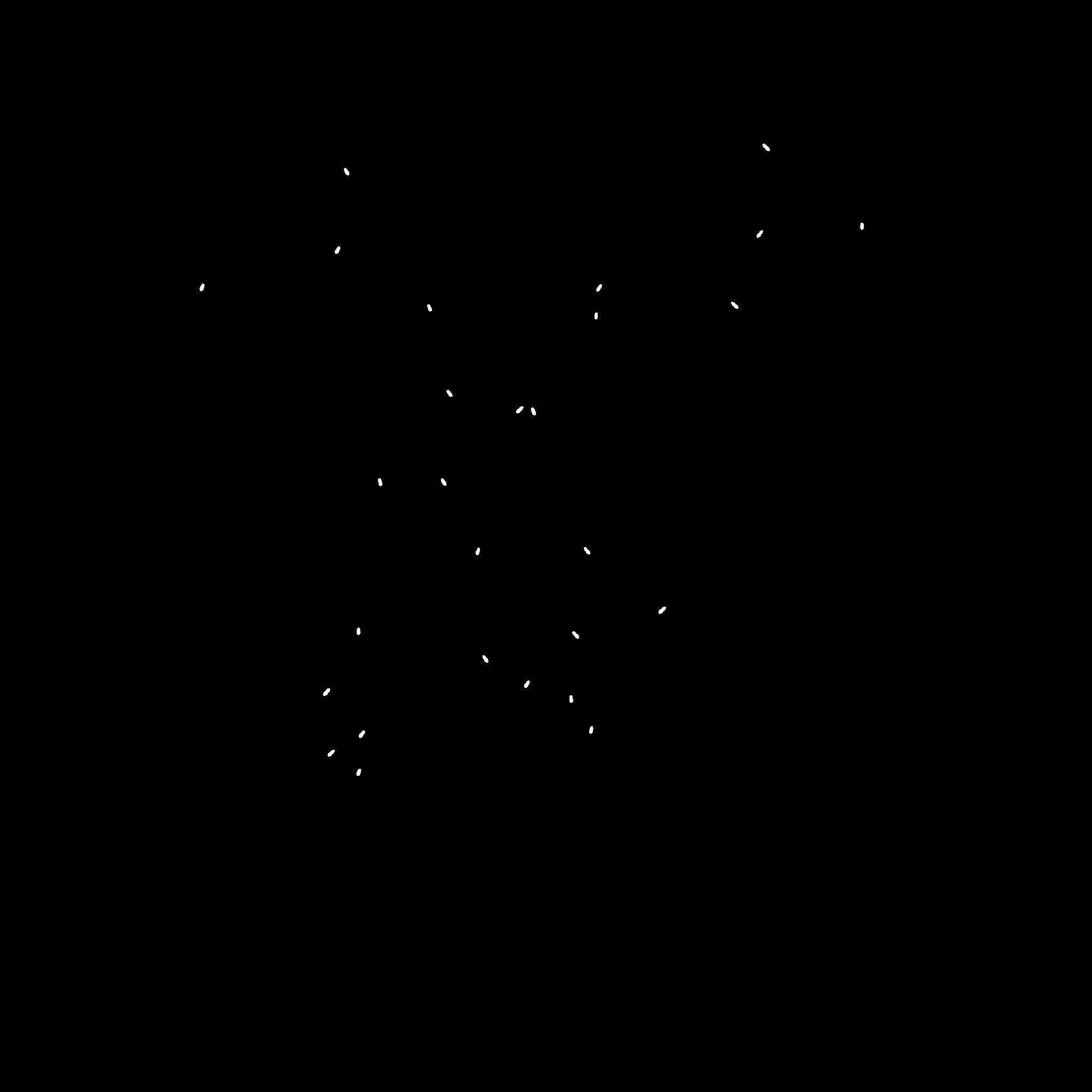

Supplement: S1 File — (ZIP) [file pone.0132101.s003.zip › ORsrc/nonortho/simu028/camx/imx149.jpg]

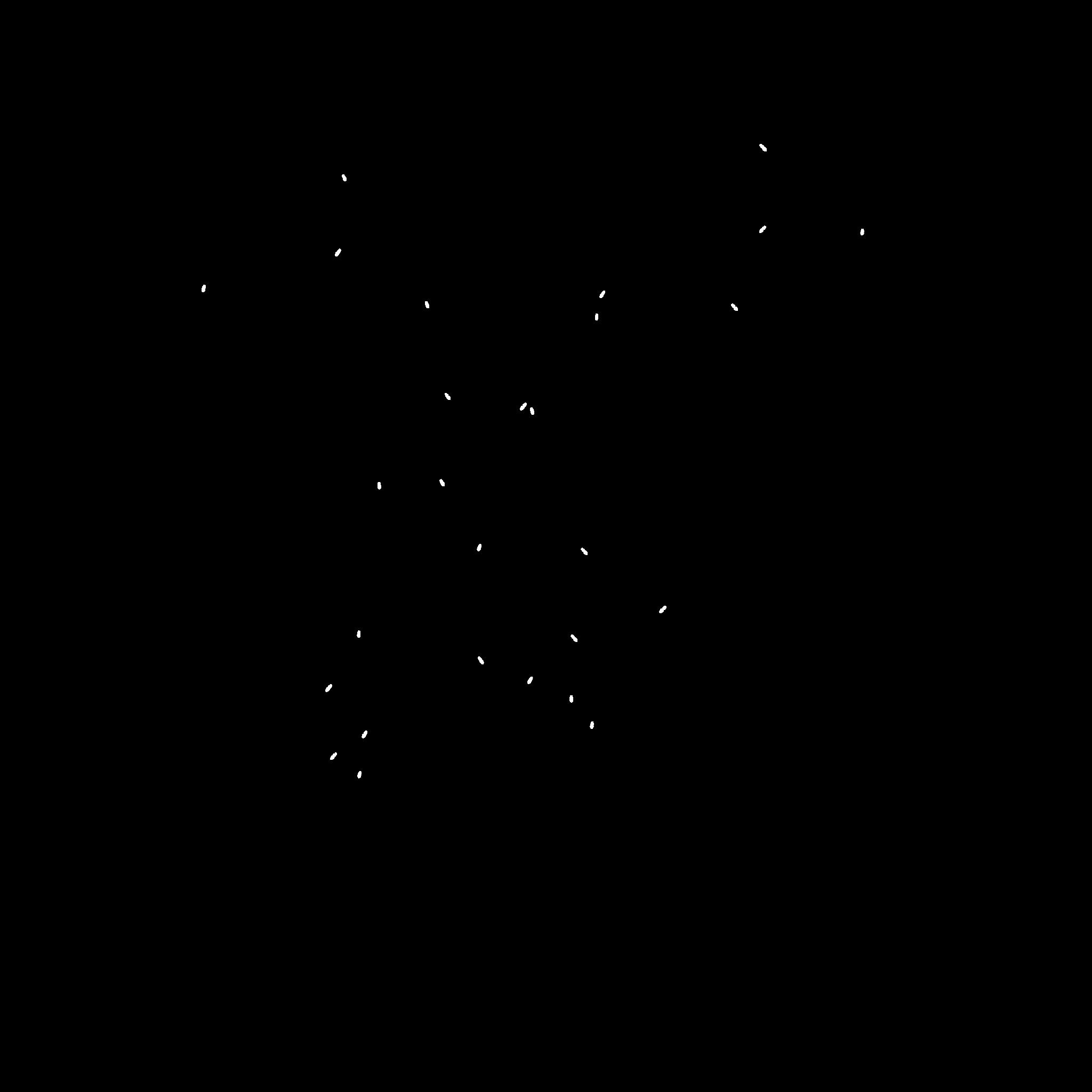

Supplement: S1 File — (ZIP) [file pone.0132101.s003.zip › ORsrc/nonortho/simu028/camx/imx150.jpg]

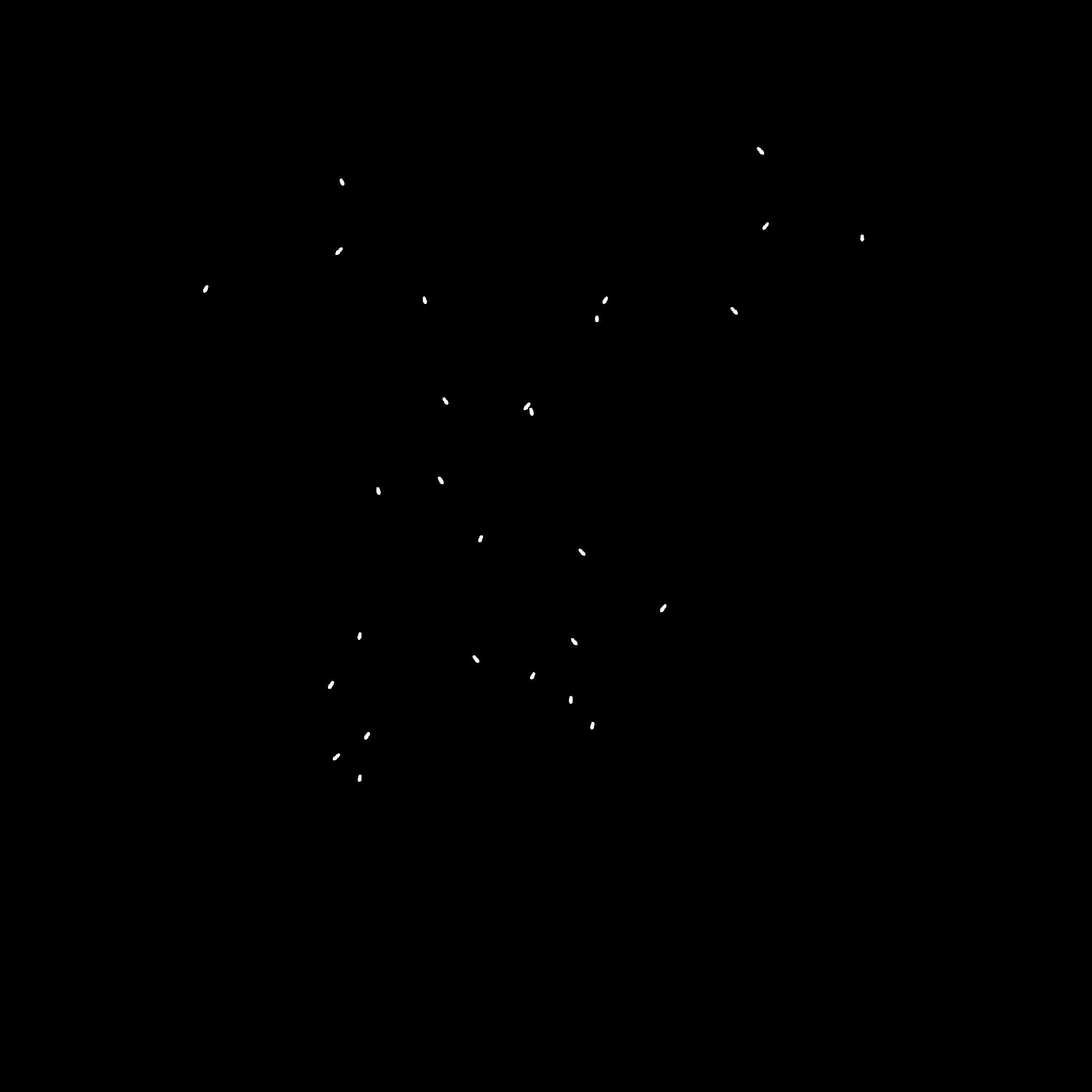

Supplement: S1 File — (ZIP) [file pone.0132101.s003.zip › ORsrc/nonortho/simu028/camx/imx151.jpg]

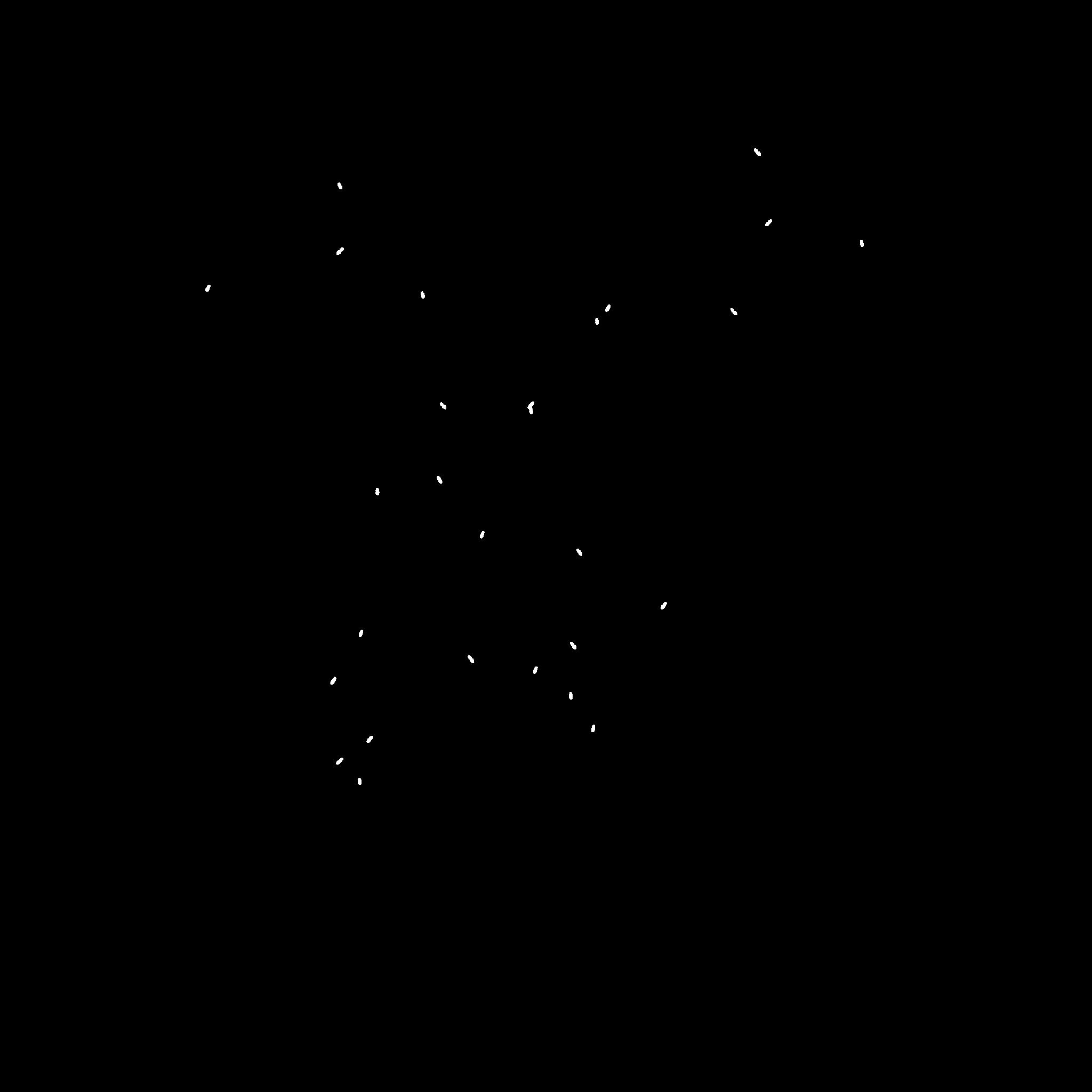

Supplement: S1 File — (ZIP) [file pone.0132101.s003.zip › ORsrc/nonortho/simu028/camx/imx152.jpg]

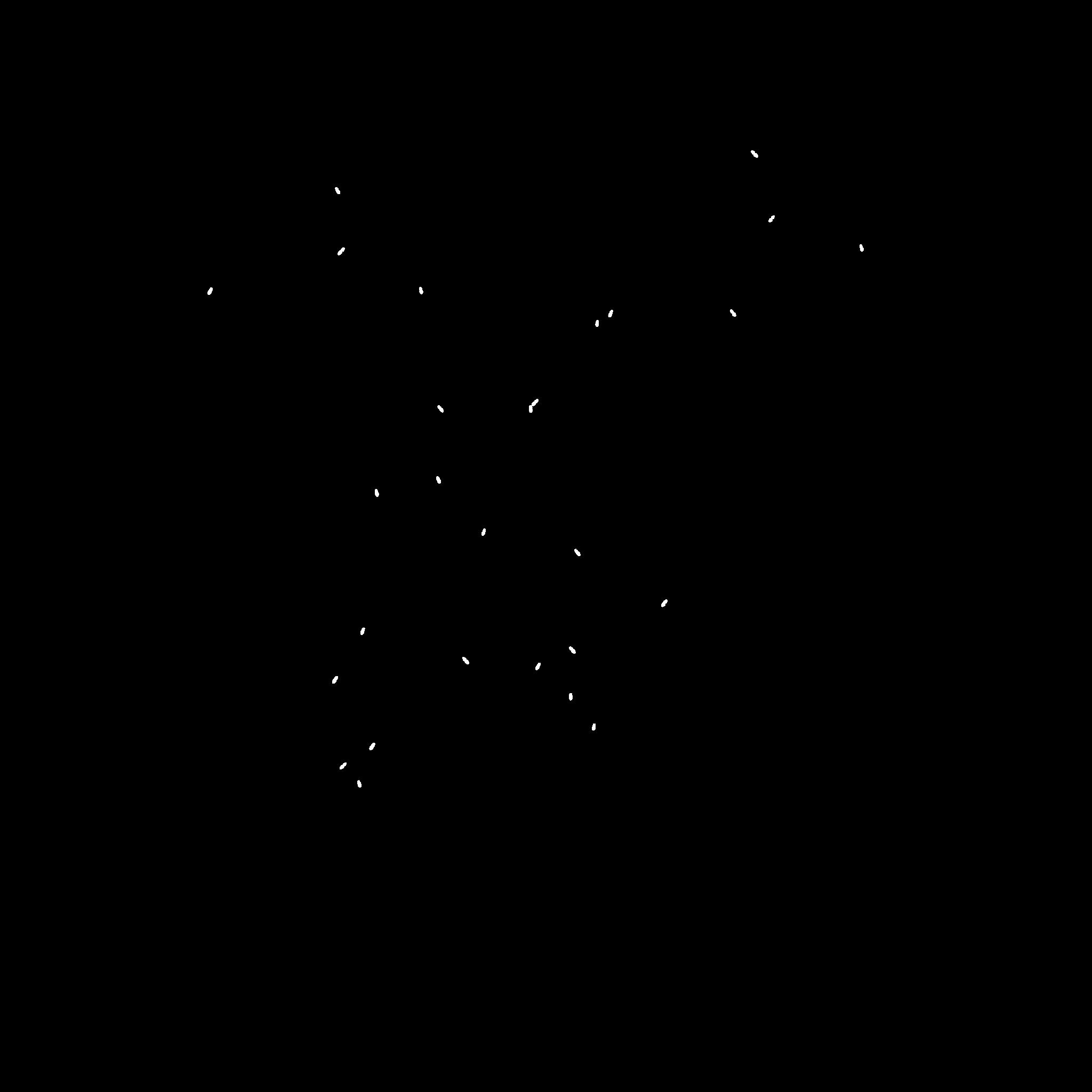

Supplement: S1 File — (ZIP) [file pone.0132101.s003.zip › ORsrc/nonortho/simu028/camx/imx153.jpg]

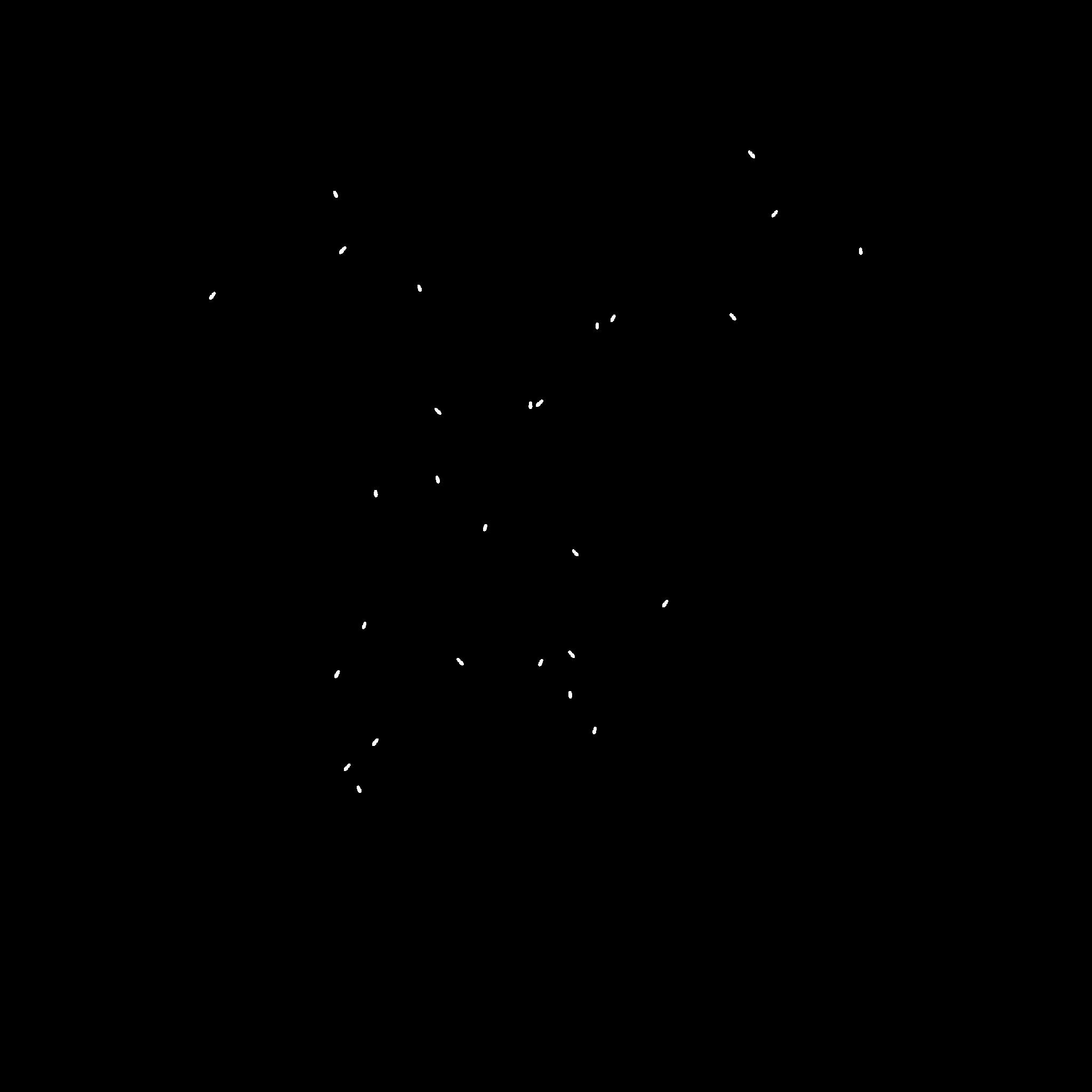

Supplement: S1 File — (ZIP) [file pone.0132101.s003.zip › ORsrc/nonortho/simu028/camx/imx154.jpg]

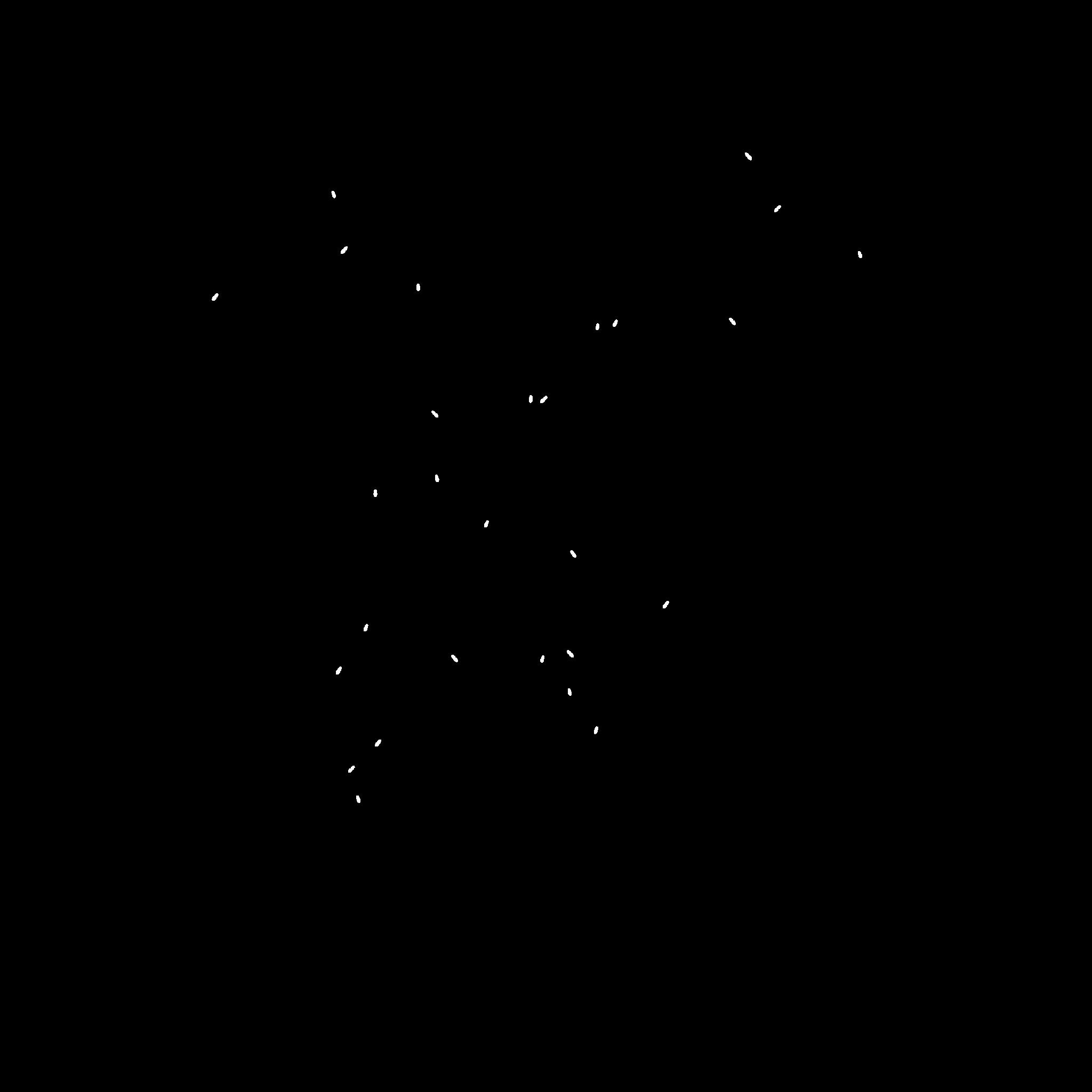

Supplement: S1 File — (ZIP) [file pone.0132101.s003.zip › ORsrc/nonortho/simu028/camx/imx155.jpg]

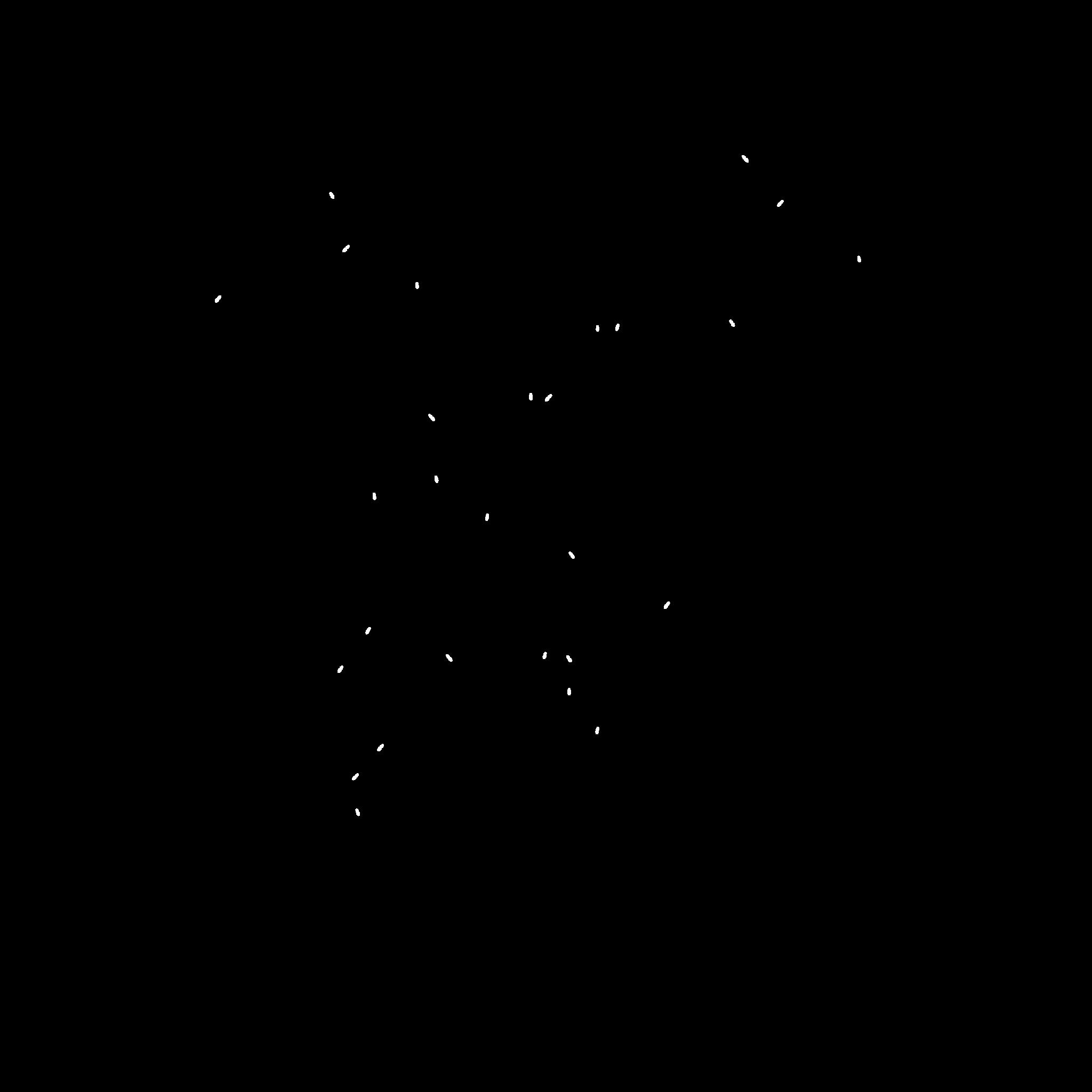

Supplement: S1 File — (ZIP) [file pone.0132101.s003.zip › ORsrc/nonortho/simu028/camx/imx156.jpg]

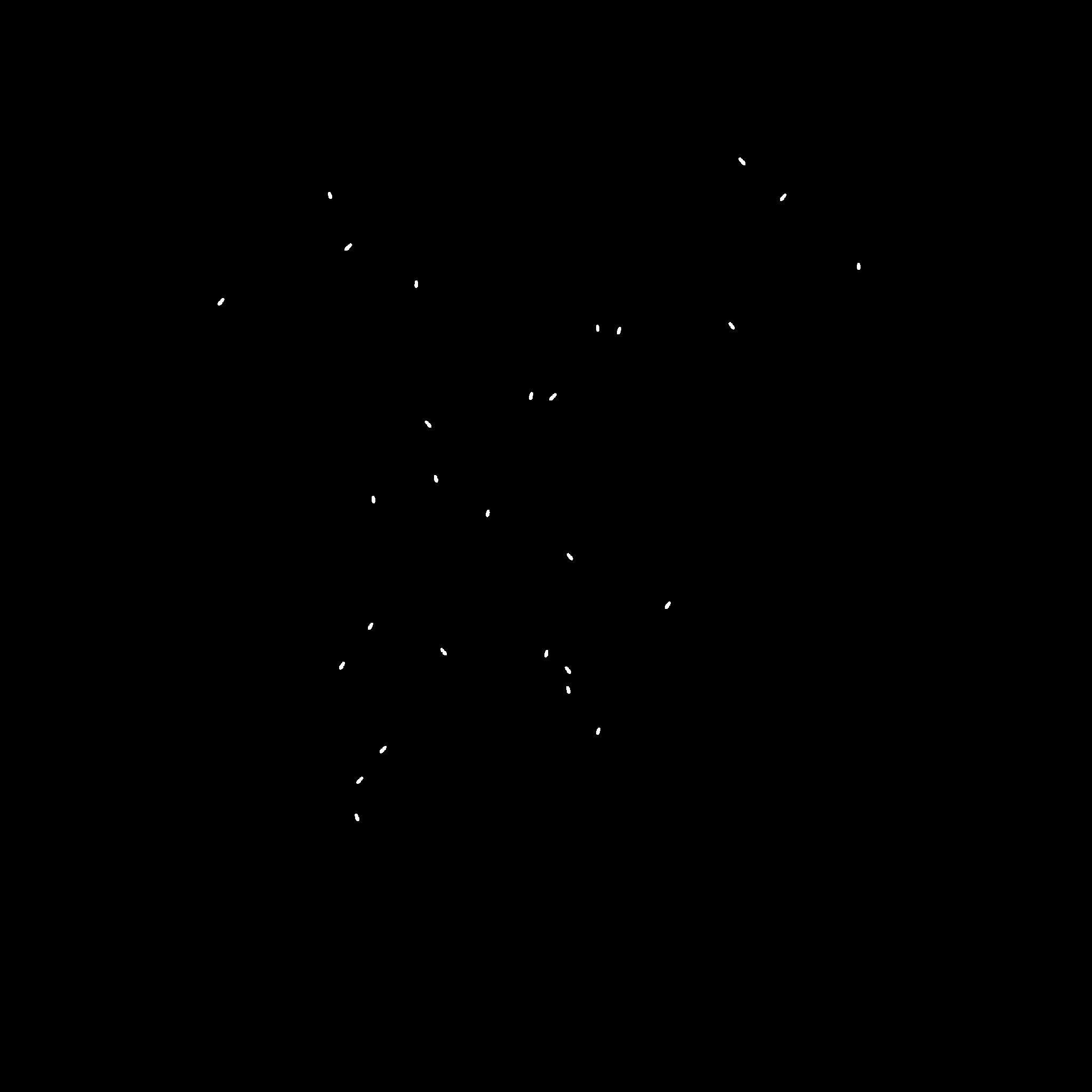

Supplement: S1 File — (ZIP) [file pone.0132101.s003.zip › ORsrc/nonortho/simu028/camx/imx157.jpg]

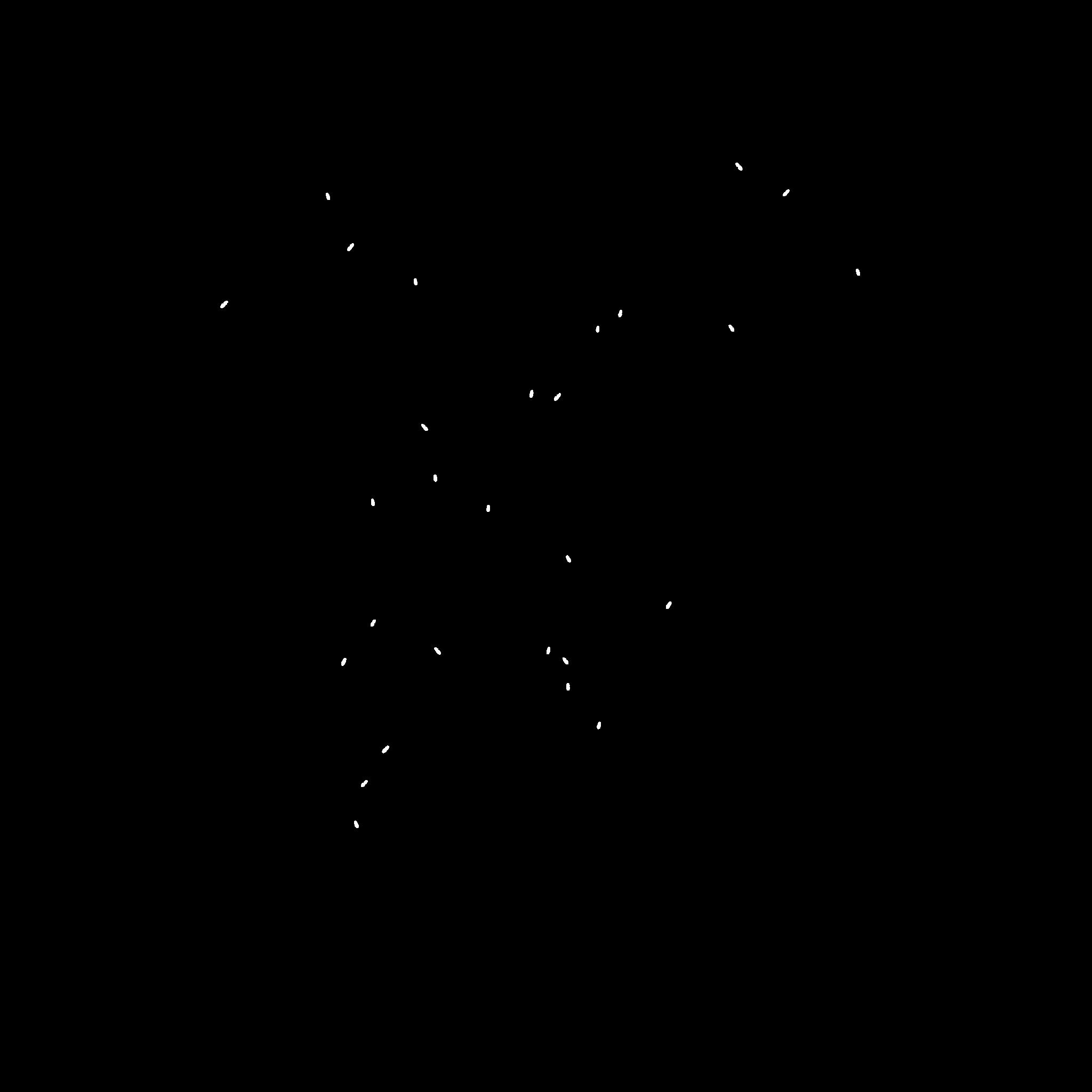

Supplement: S1 File — (ZIP) [file pone.0132101.s003.zip › ORsrc/nonortho/simu028/camx/imx158.jpg]

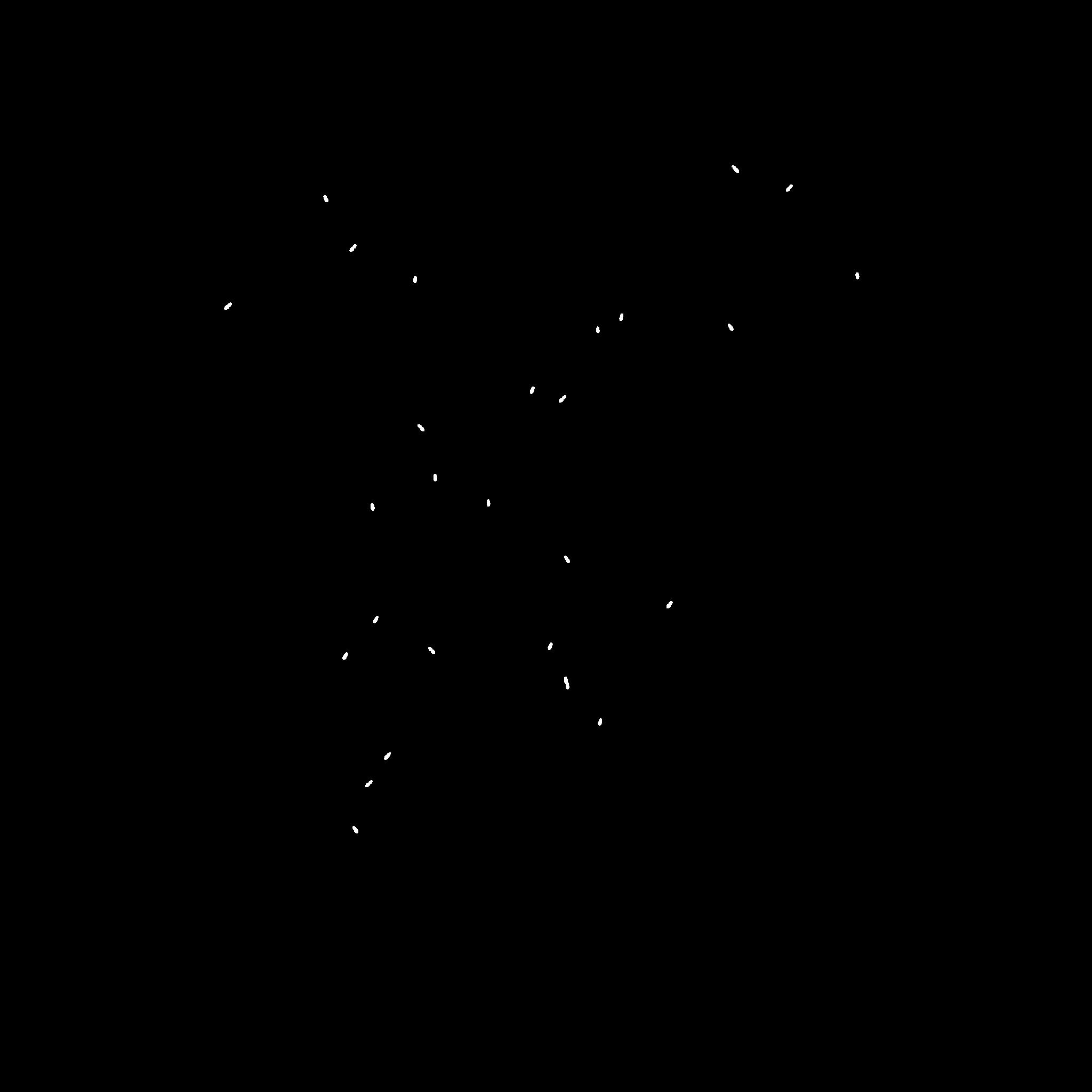

Supplement: S1 File — (ZIP) [file pone.0132101.s003.zip › ORsrc/nonortho/simu028/camx/imx159.jpg]

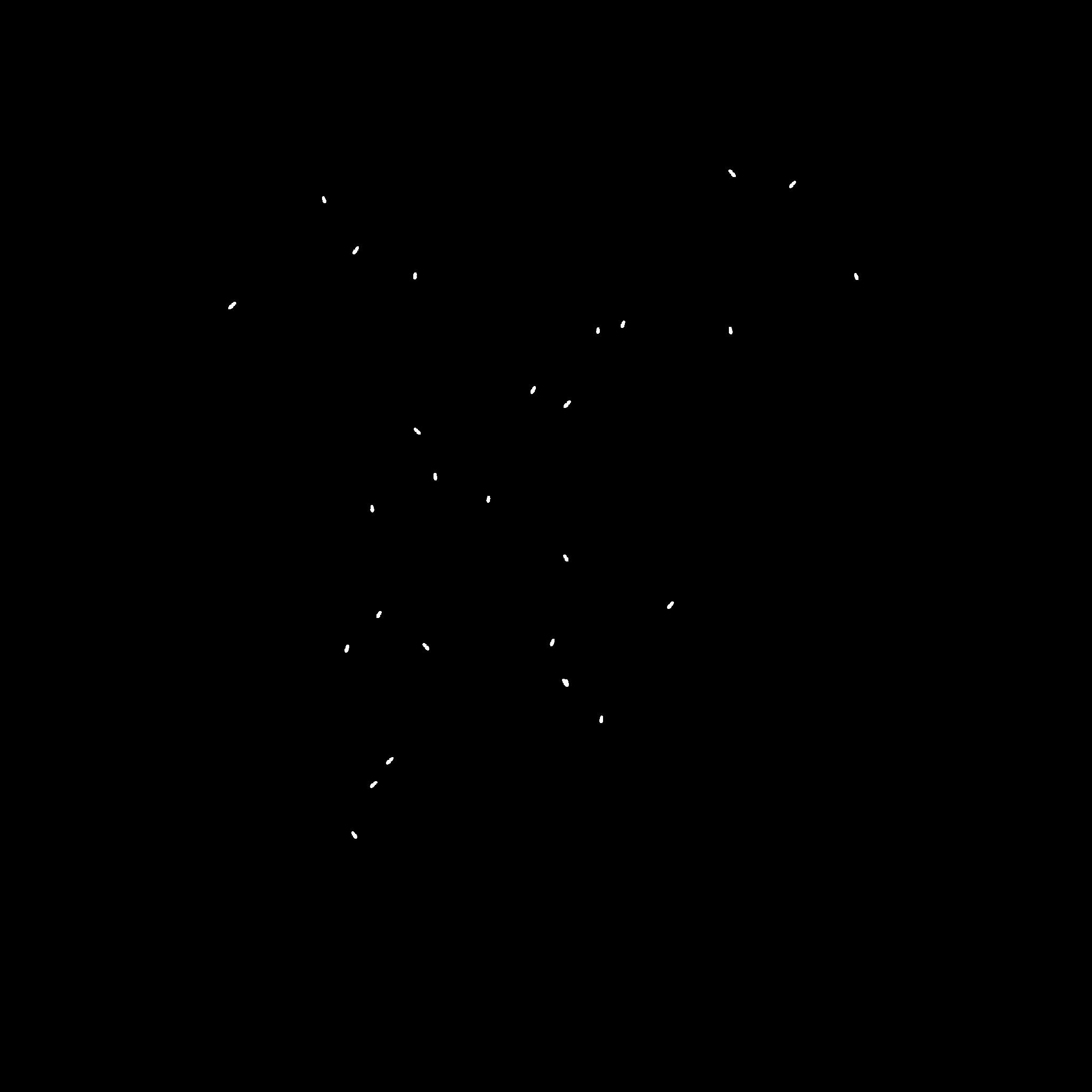

Supplement: S1 File — (ZIP) [file pone.0132101.s003.zip › ORsrc/nonortho/simu028/camx/imx160.jpg]

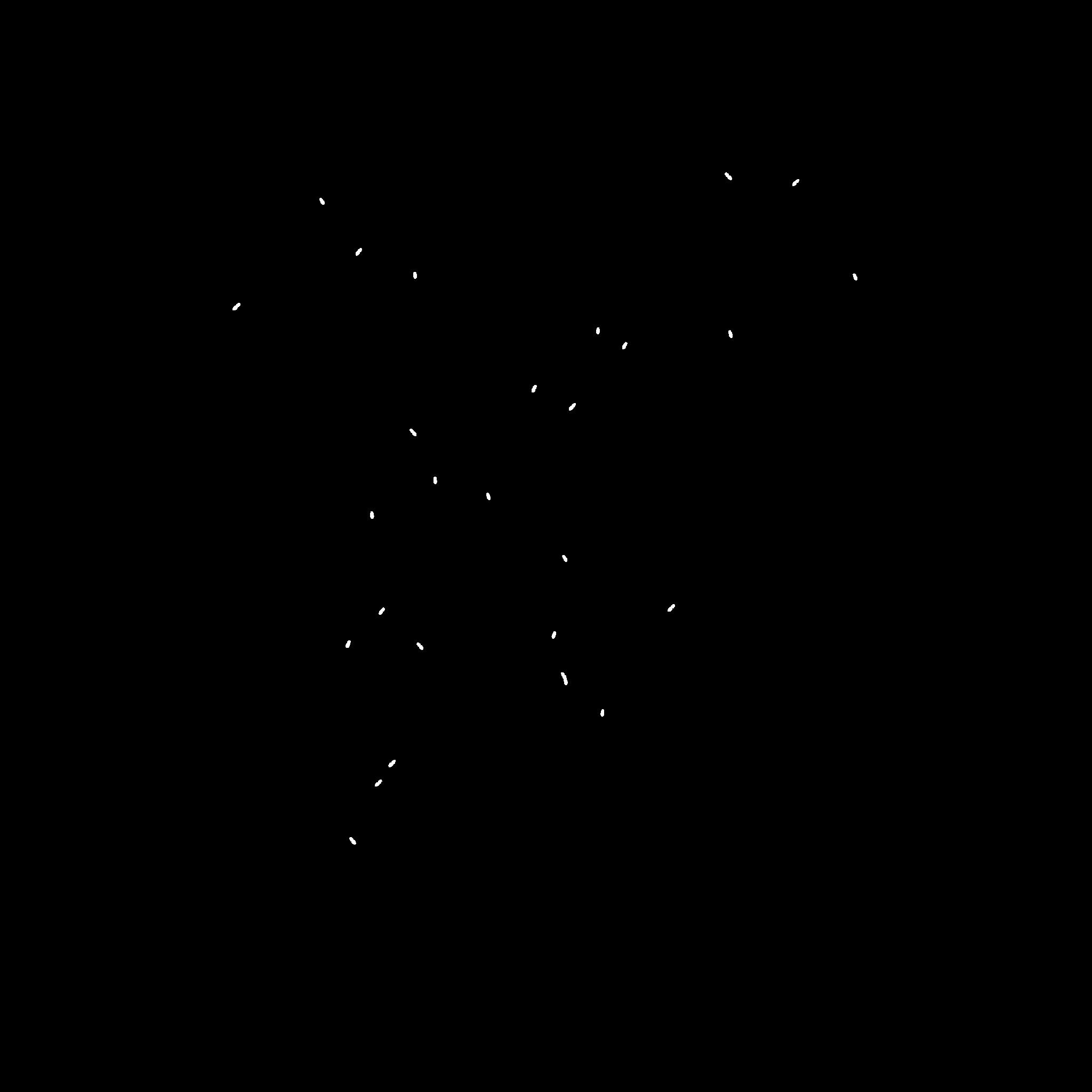

Supplement: S1 File — (ZIP) [file pone.0132101.s003.zip › ORsrc/nonortho/simu028/camx/imx161.jpg]

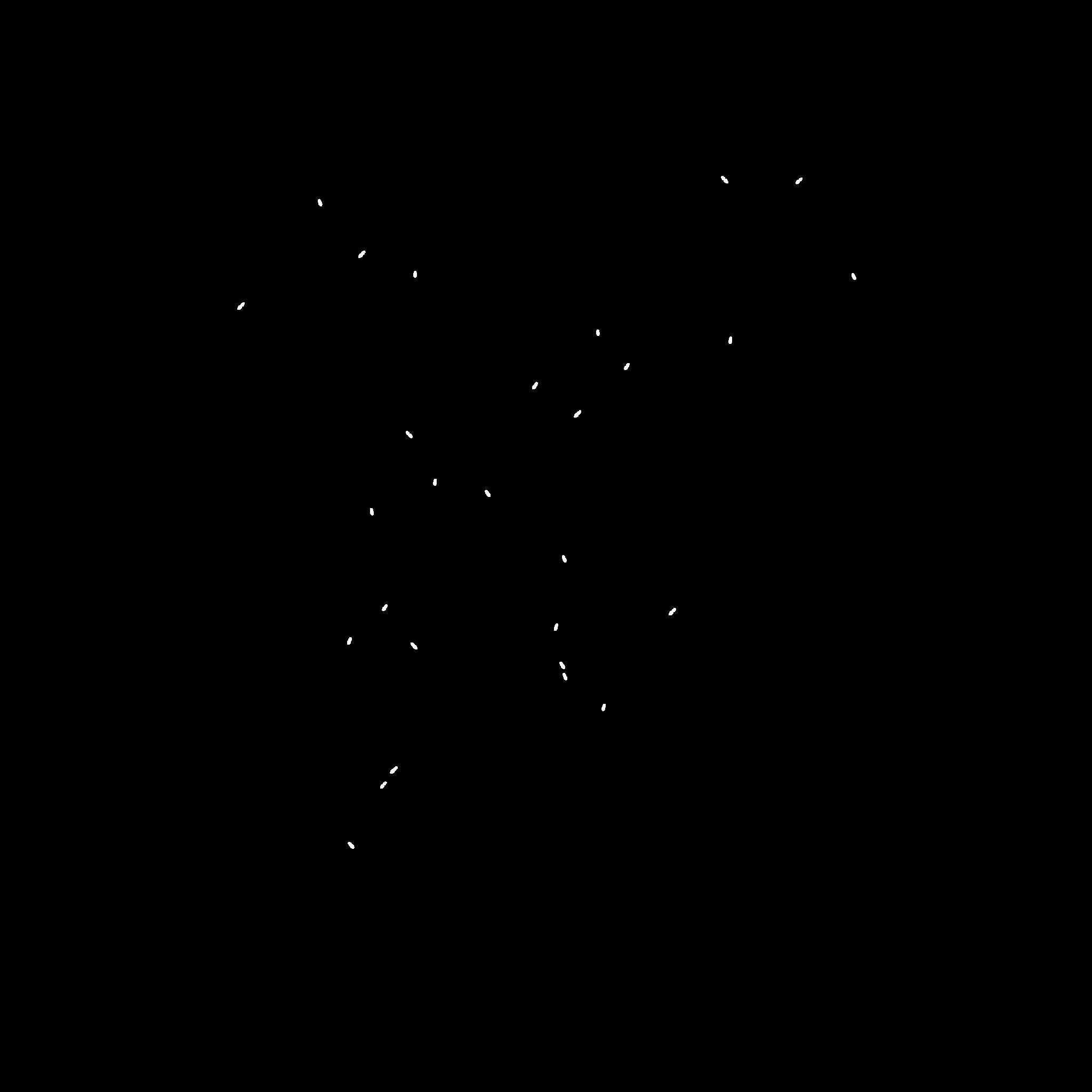

Supplement: S1 File — (ZIP) [file pone.0132101.s003.zip › ORsrc/nonortho/simu028/camx/imx162.jpg]

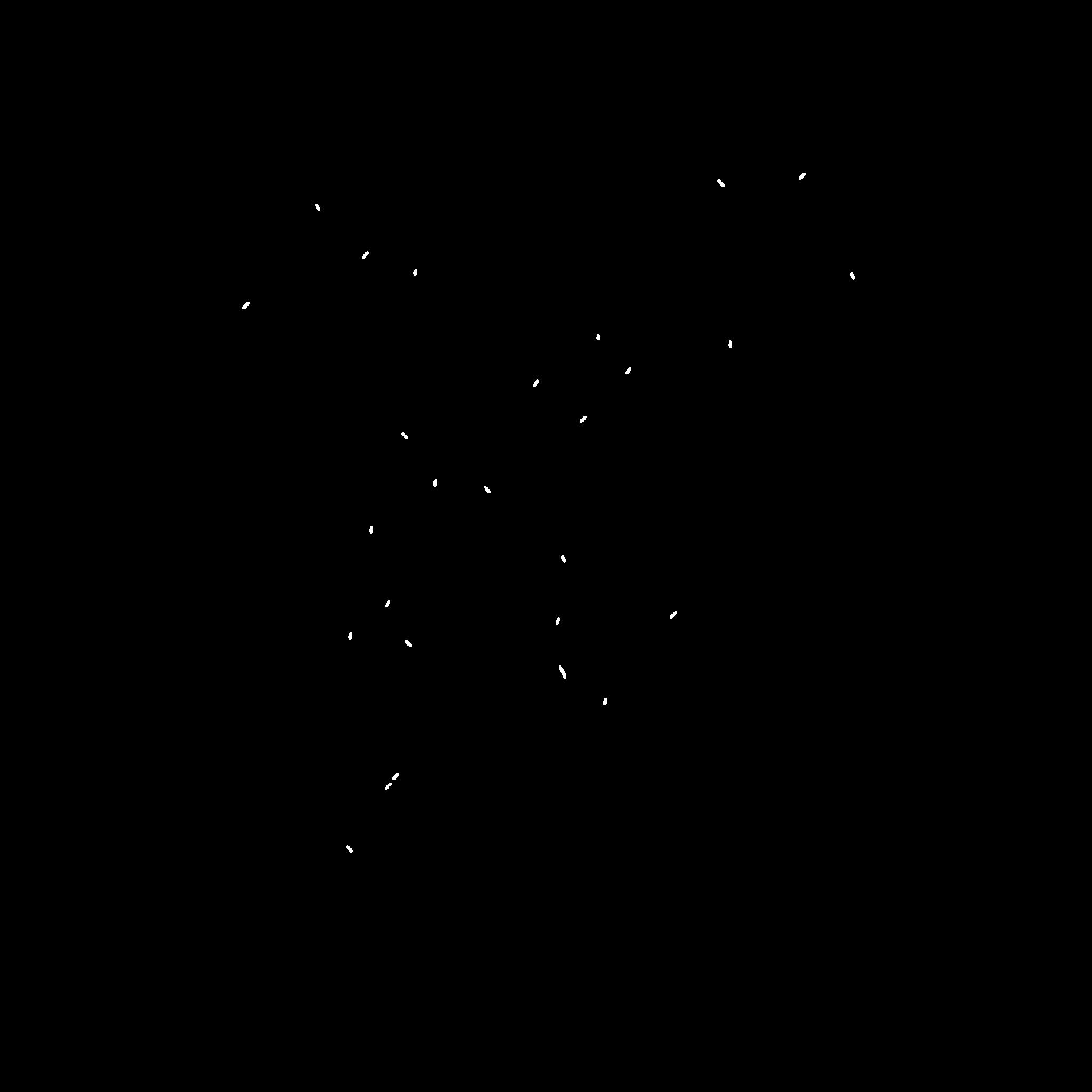

Supplement: S1 File — (ZIP) [file pone.0132101.s003.zip › ORsrc/nonortho/simu028/camx/imx163.jpg]

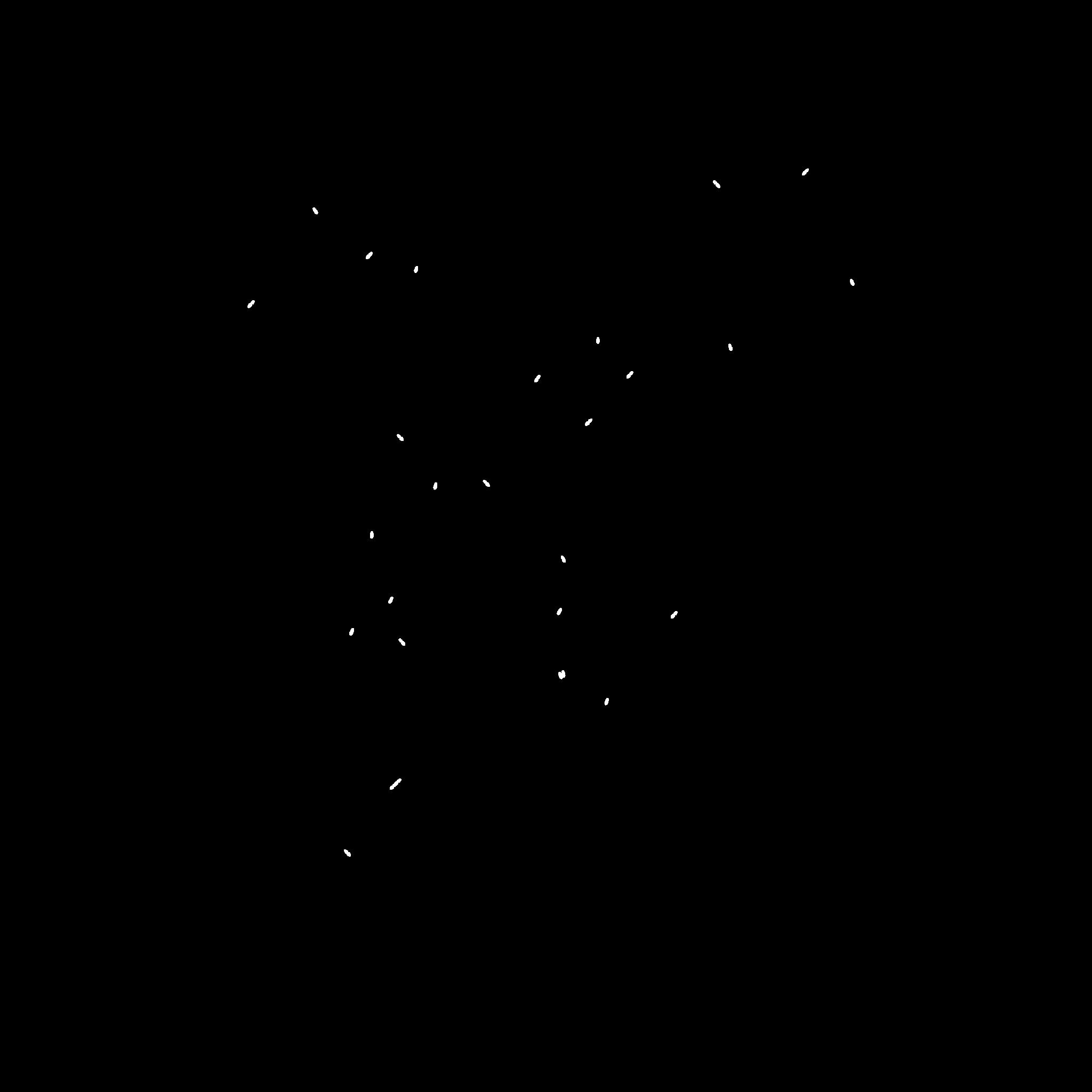

Supplement: S1 File — (ZIP) [file pone.0132101.s003.zip › ORsrc/nonortho/simu028/camx/imx164.jpg]

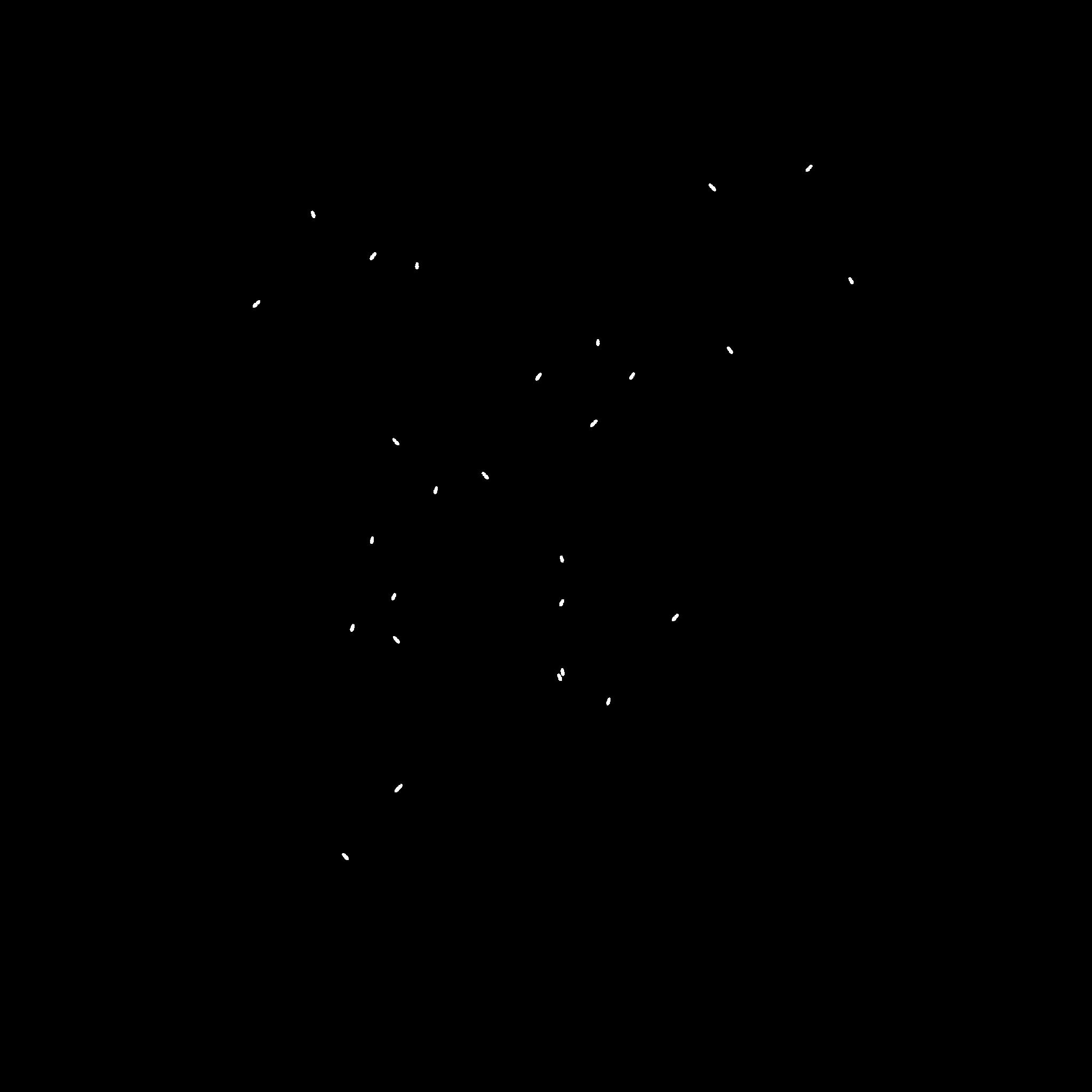

Supplement: S1 File — (ZIP) [file pone.0132101.s003.zip › ORsrc/nonortho/simu028/camx/imx165.jpg]

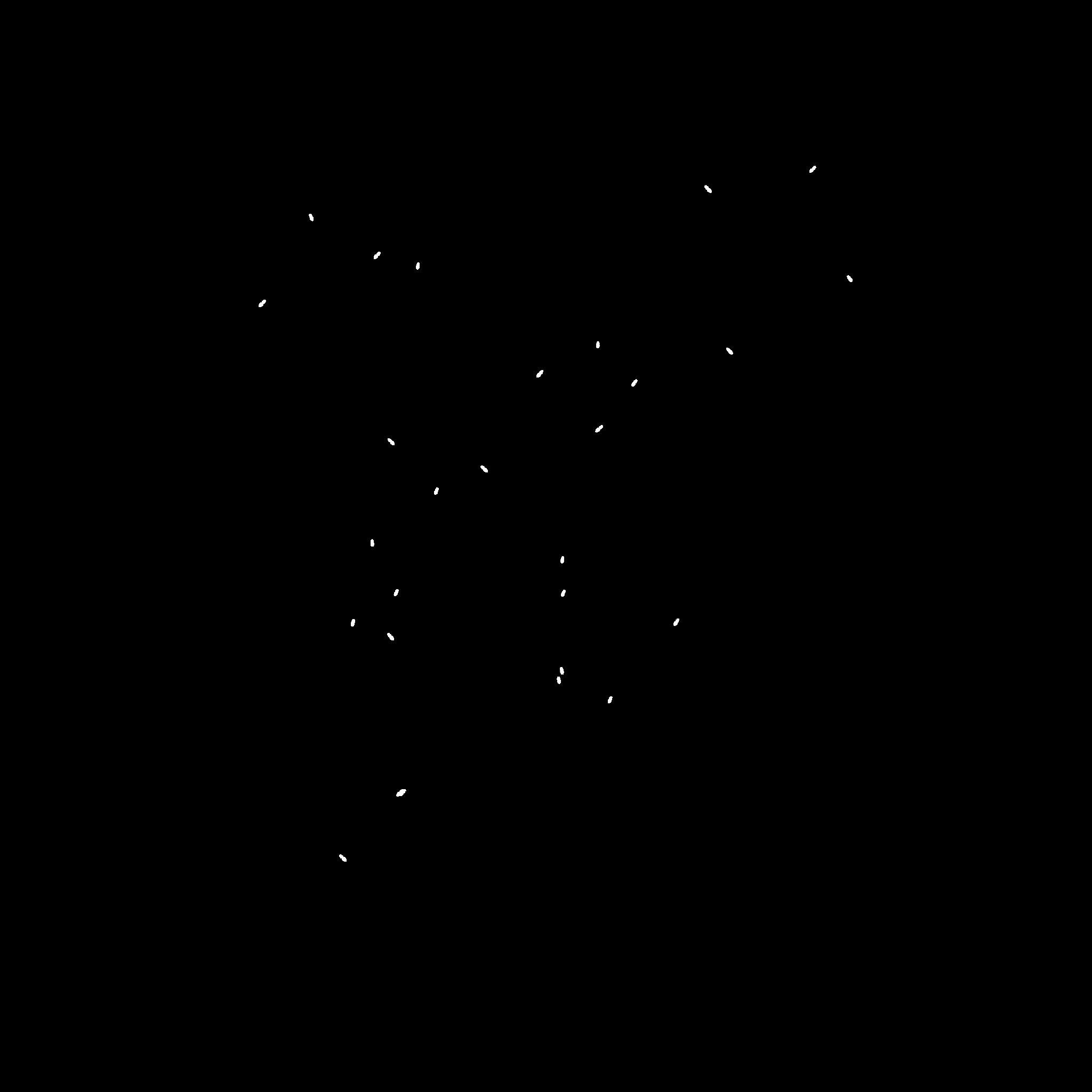

Supplement: S1 File — (ZIP) [file pone.0132101.s003.zip › ORsrc/nonortho/simu028/camx/imx166.jpg]

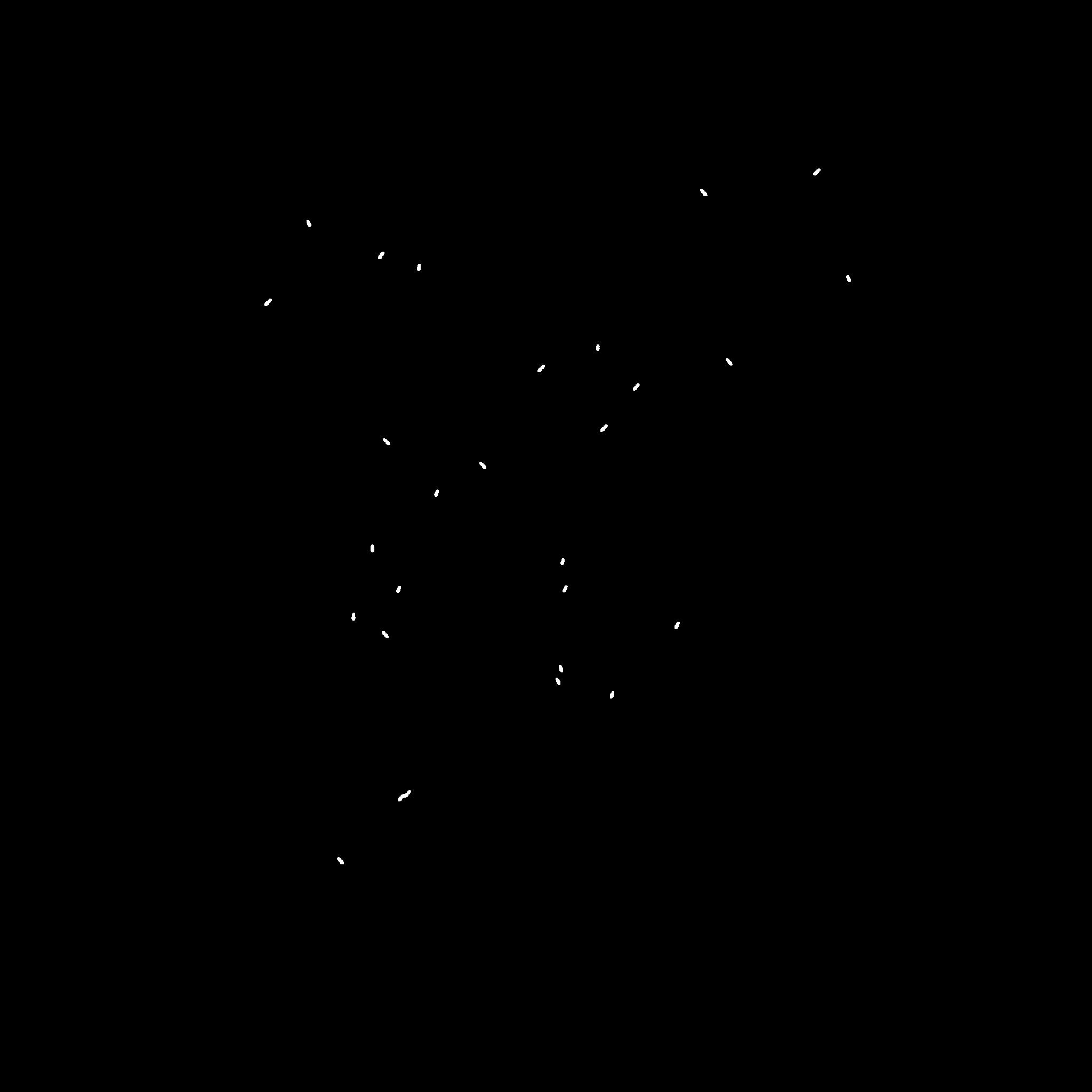

Supplement: S1 File — (ZIP) [file pone.0132101.s003.zip › ORsrc/nonortho/simu028/camx/imx167.jpg]

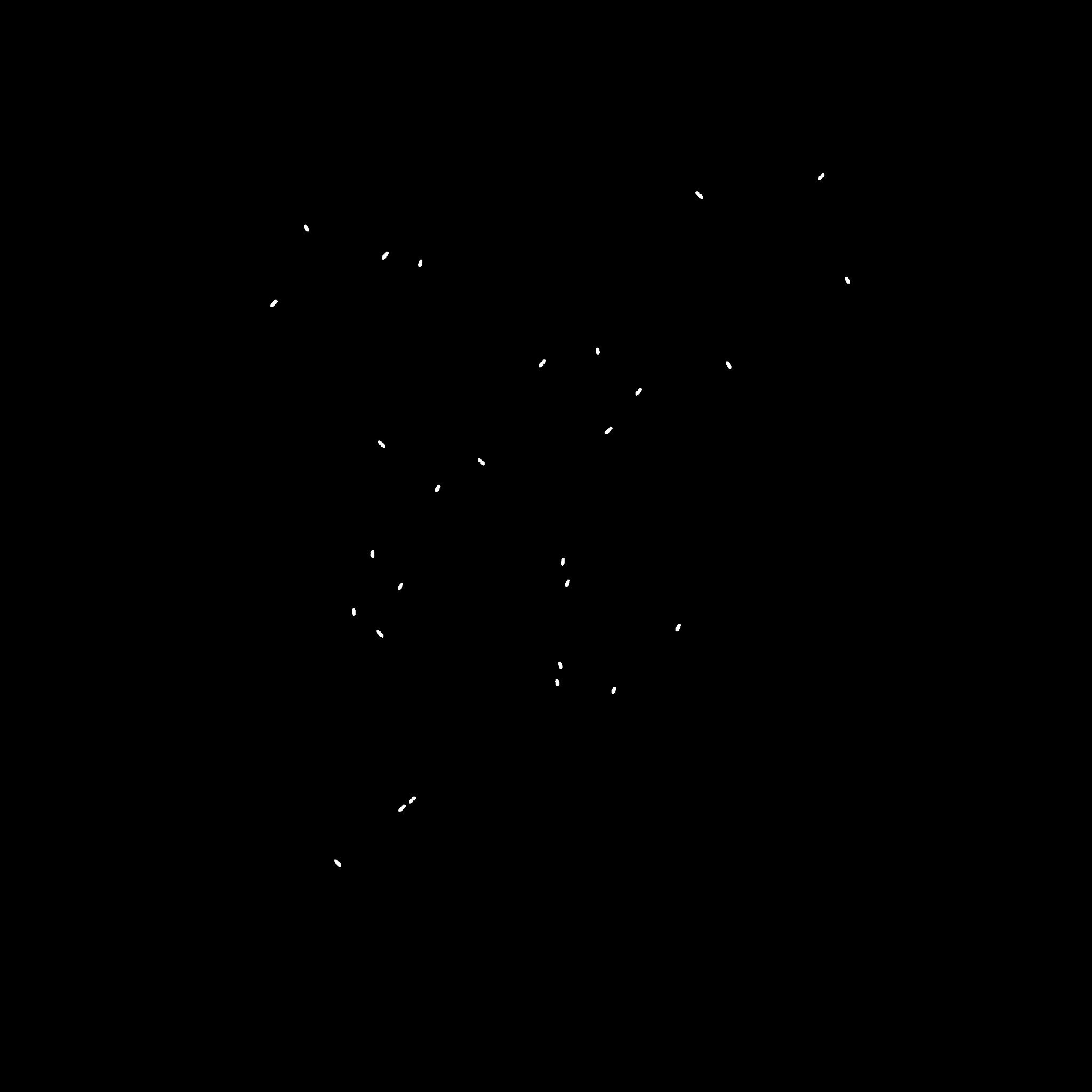

Supplement: S1 File — (ZIP) [file pone.0132101.s003.zip › ORsrc/nonortho/simu028/camx/imx168.jpg]

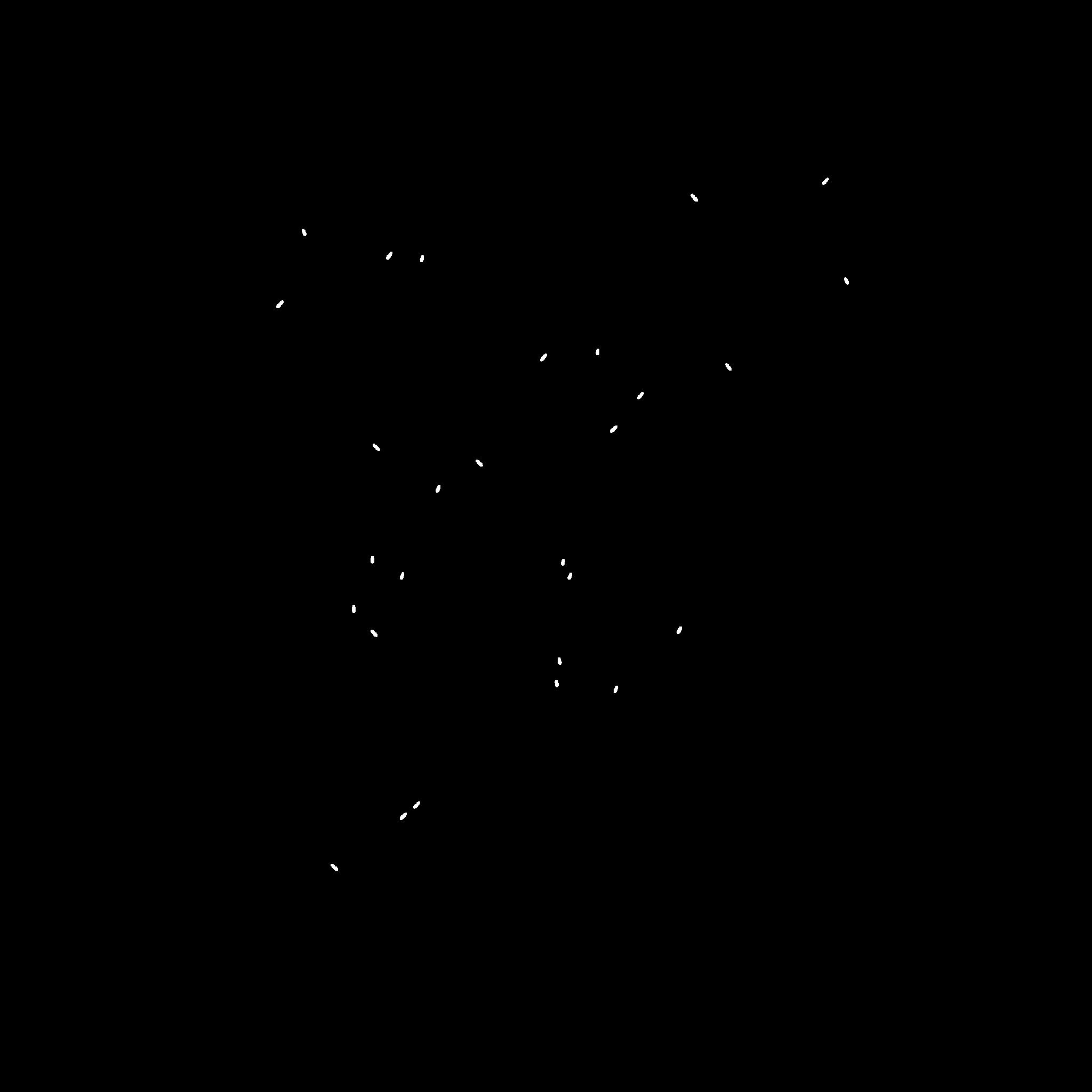

Supplement: S1 File — (ZIP) [file pone.0132101.s003.zip › ORsrc/nonortho/simu028/camx/imx169.jpg]

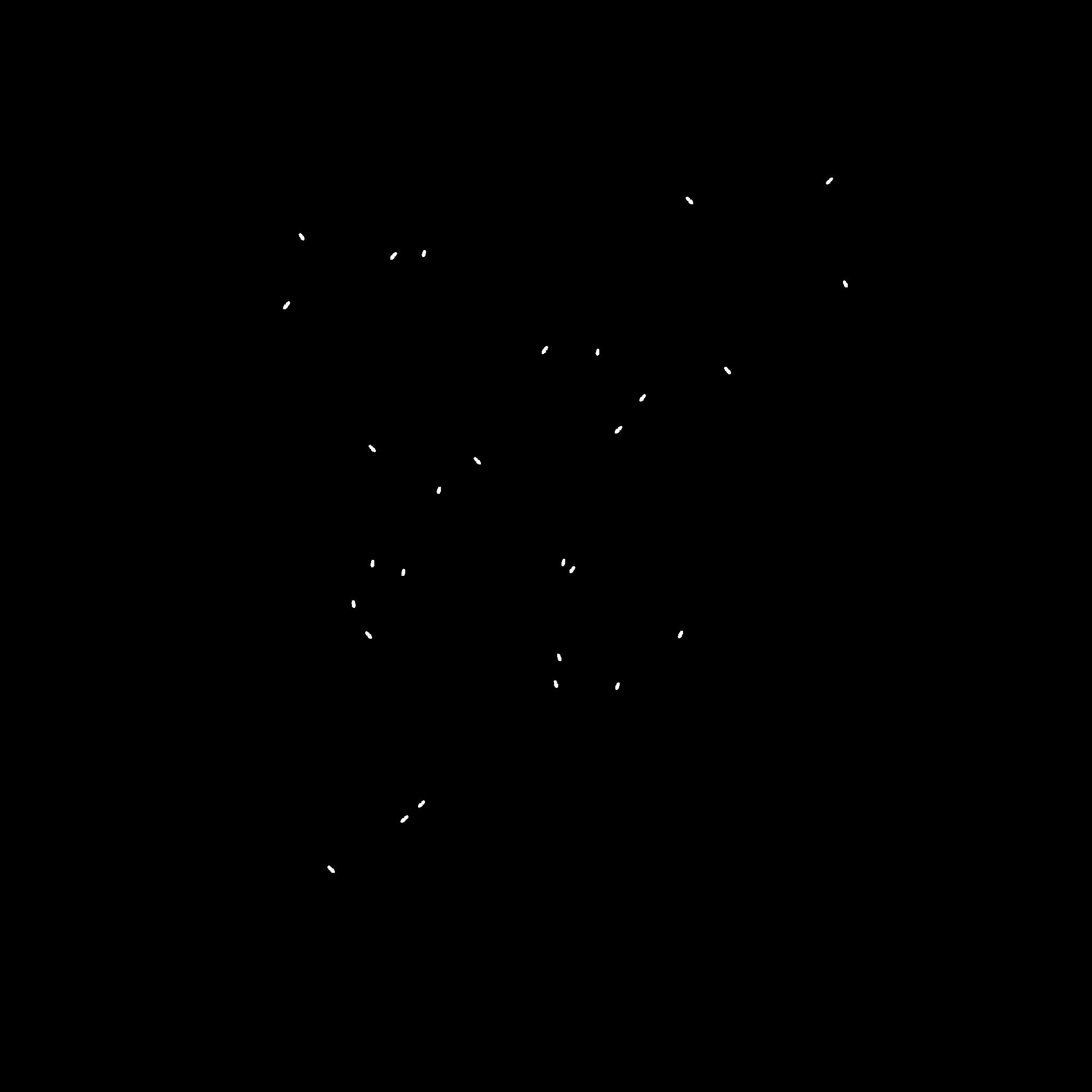

Supplement: S1 File — (ZIP) [file pone.0132101.s003.zip › ORsrc/nonortho/simu028/camx/imx170.jpg]

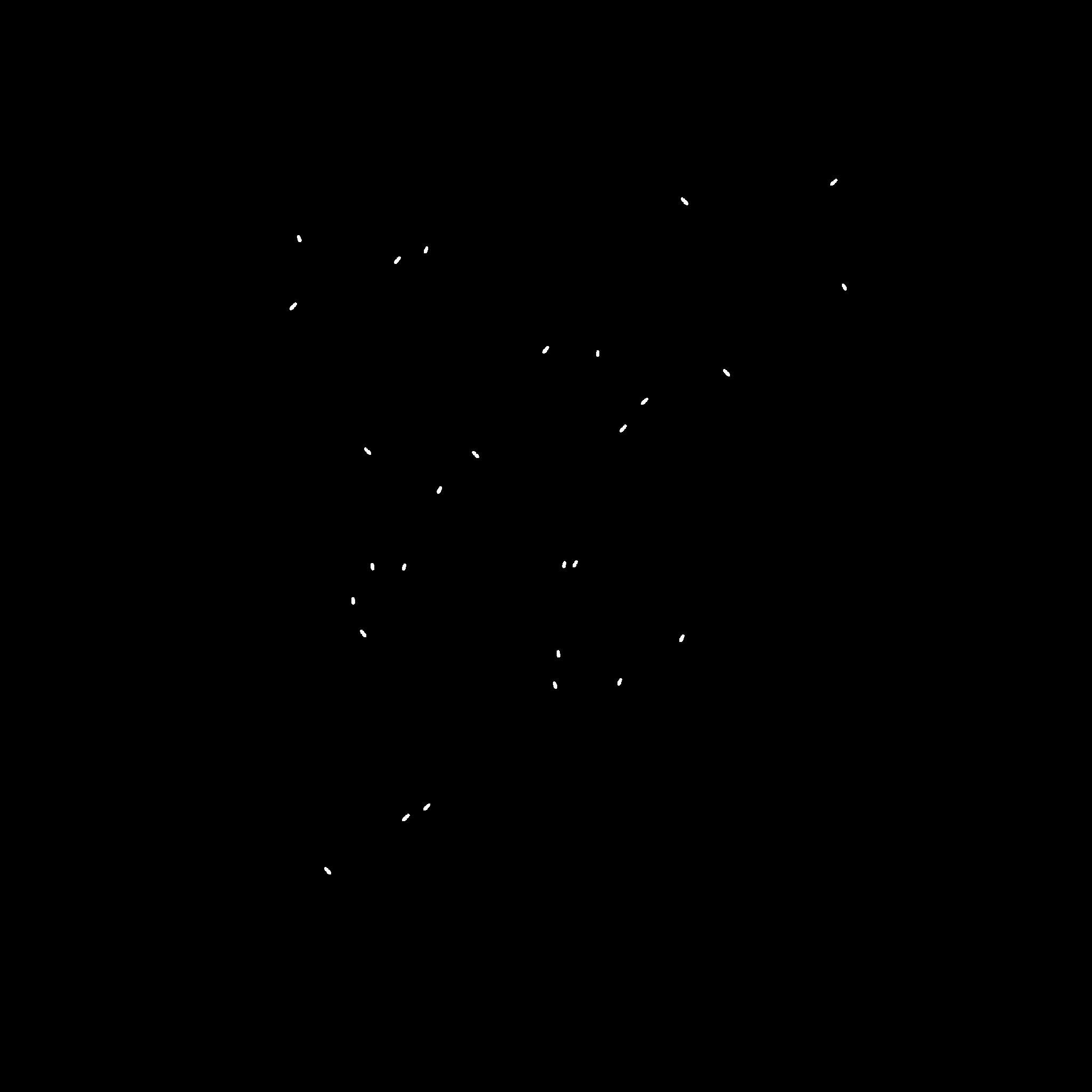

Supplement: S1 File — (ZIP) [file pone.0132101.s003.zip › ORsrc/nonortho/simu028/camx/imx171.jpg]

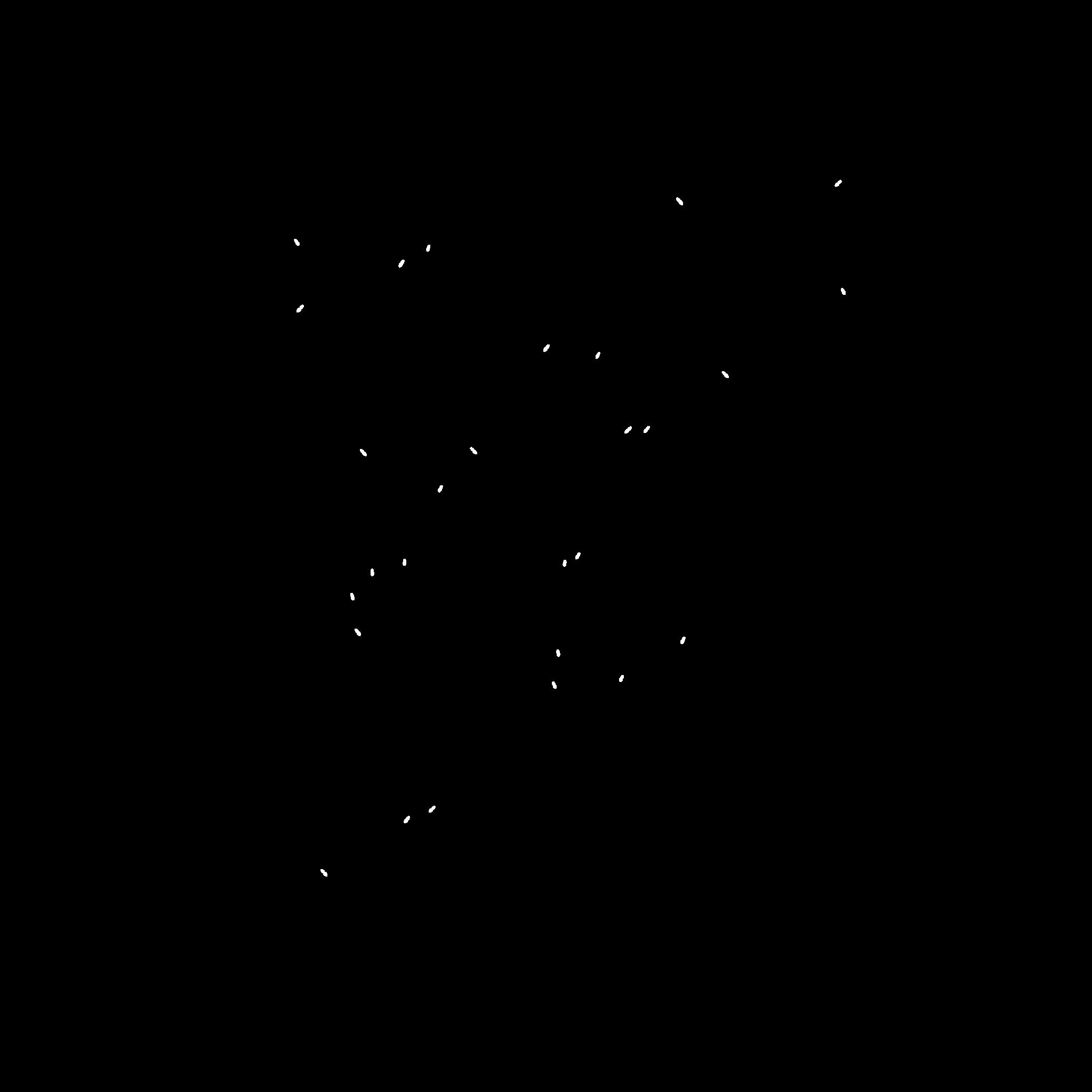

Supplement: S1 File — (ZIP) [file pone.0132101.s003.zip › ORsrc/nonortho/simu028/camx/imx172.jpg]

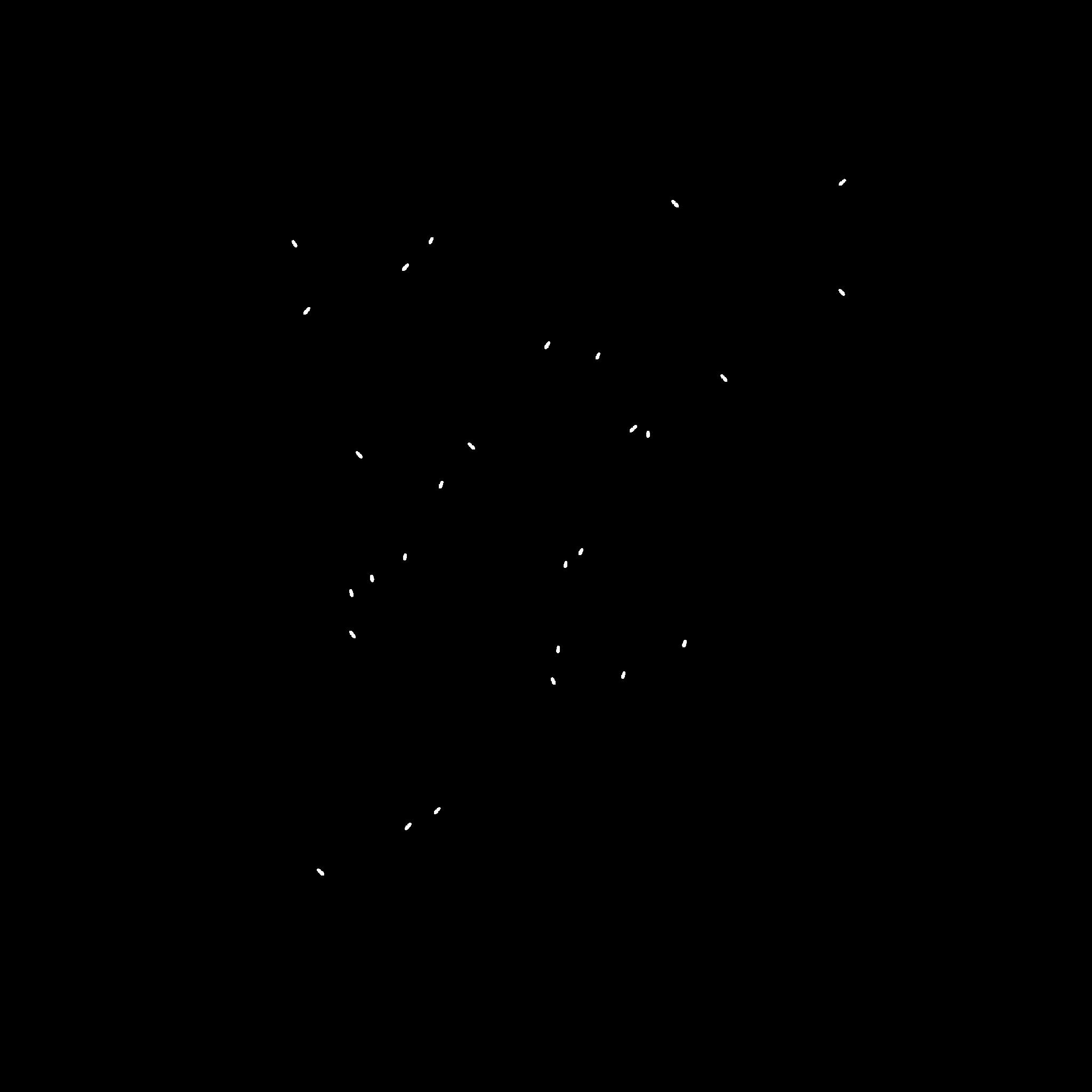

Supplement: S1 File — (ZIP) [file pone.0132101.s003.zip › ORsrc/nonortho/simu028/camx/imx173.jpg]

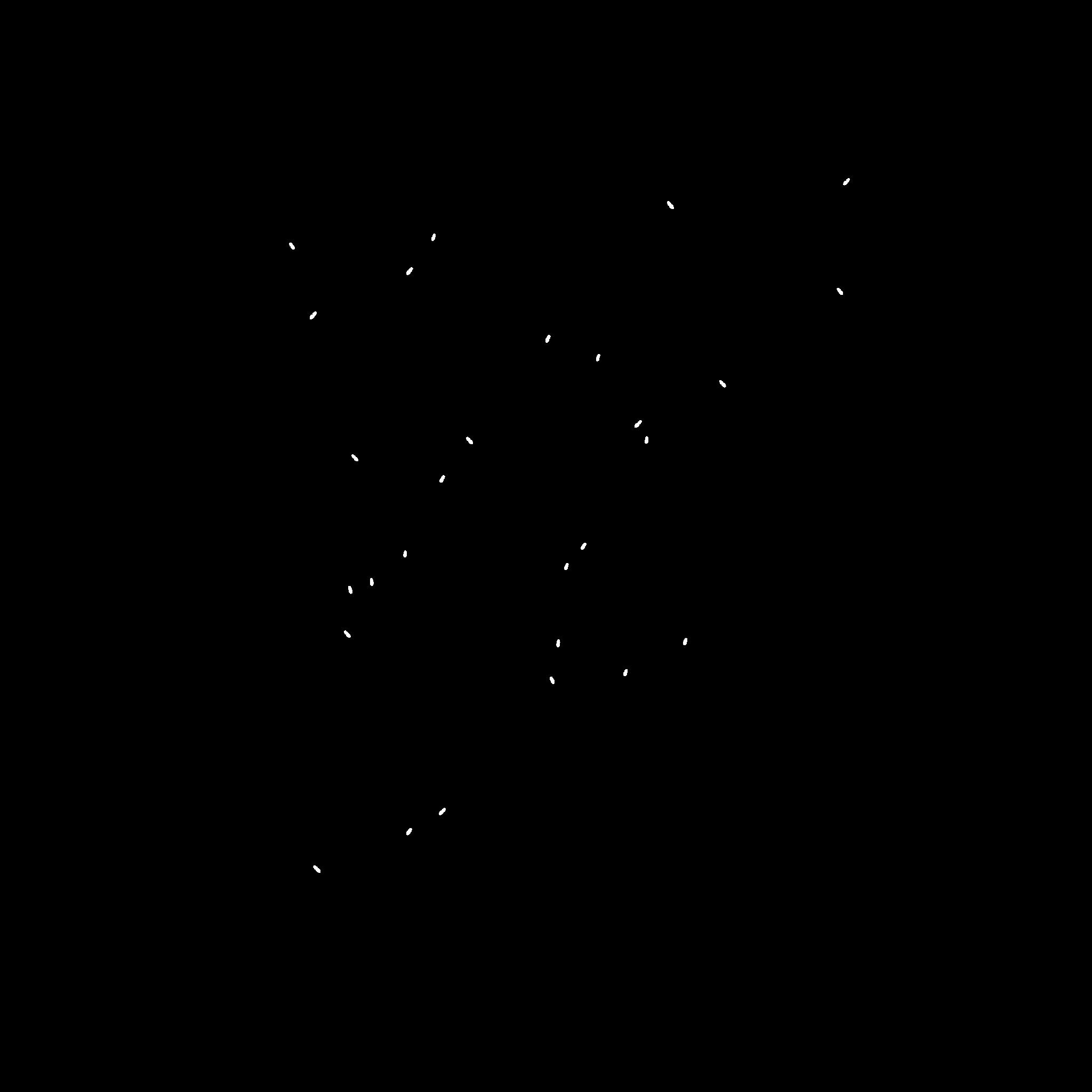

Supplement: S1 File — (ZIP) [file pone.0132101.s003.zip › ORsrc/nonortho/simu028/camx/imx174.jpg]

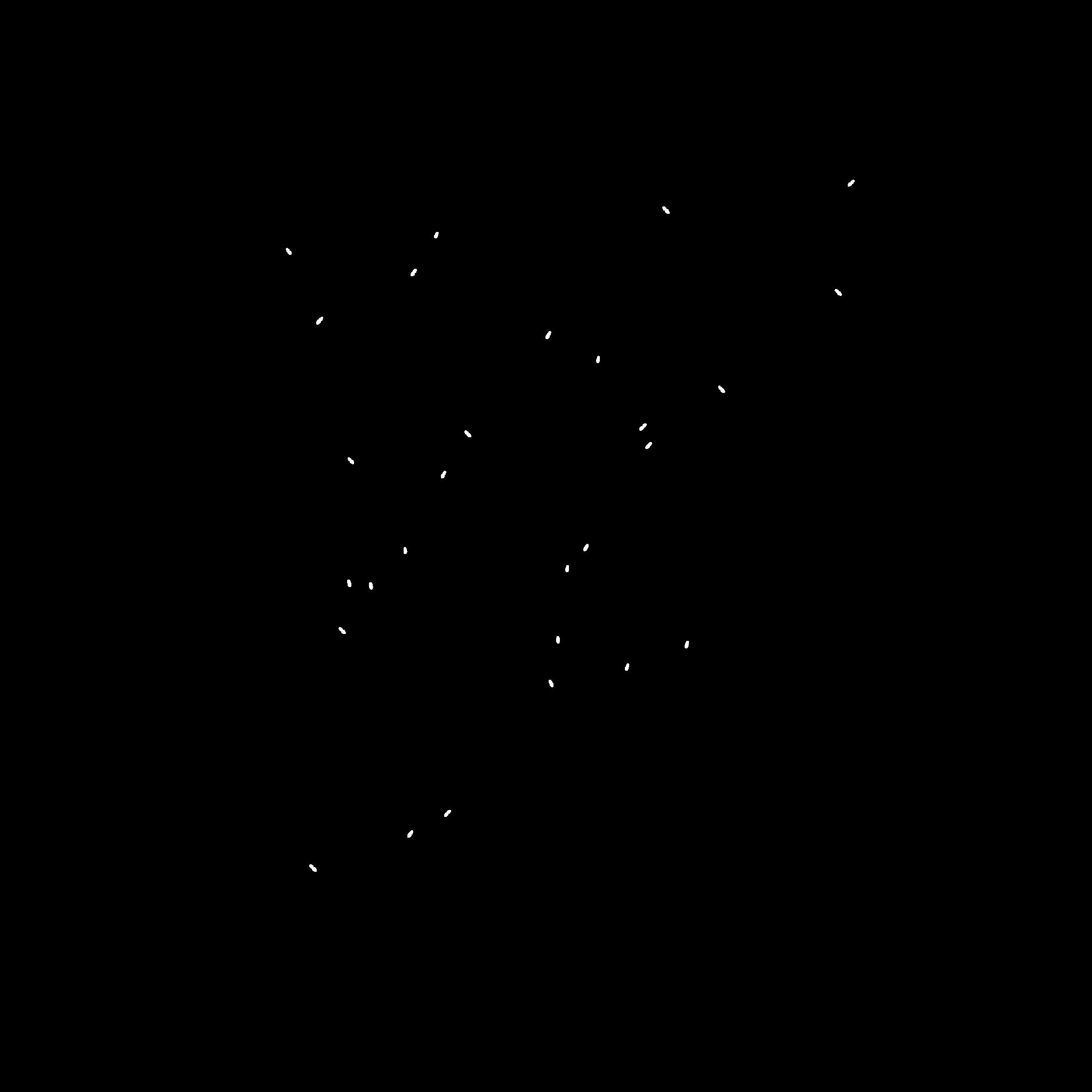

Supplement: S1 File — (ZIP) [file pone.0132101.s003.zip › ORsrc/nonortho/simu028/camx/imx175.jpg]

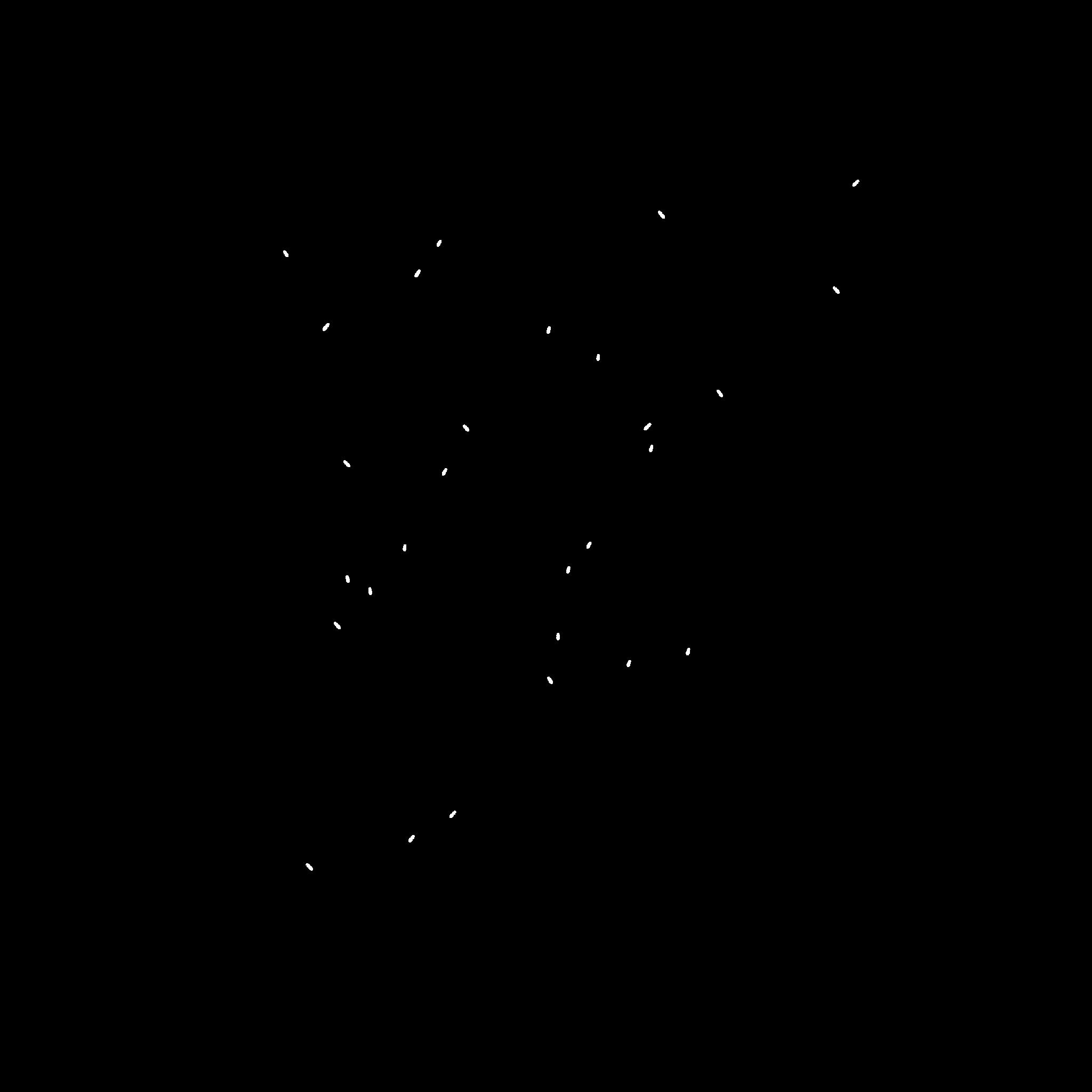

Supplement: S1 File — (ZIP) [file pone.0132101.s003.zip › ORsrc/nonortho/simu028/camx/imx176.jpg]

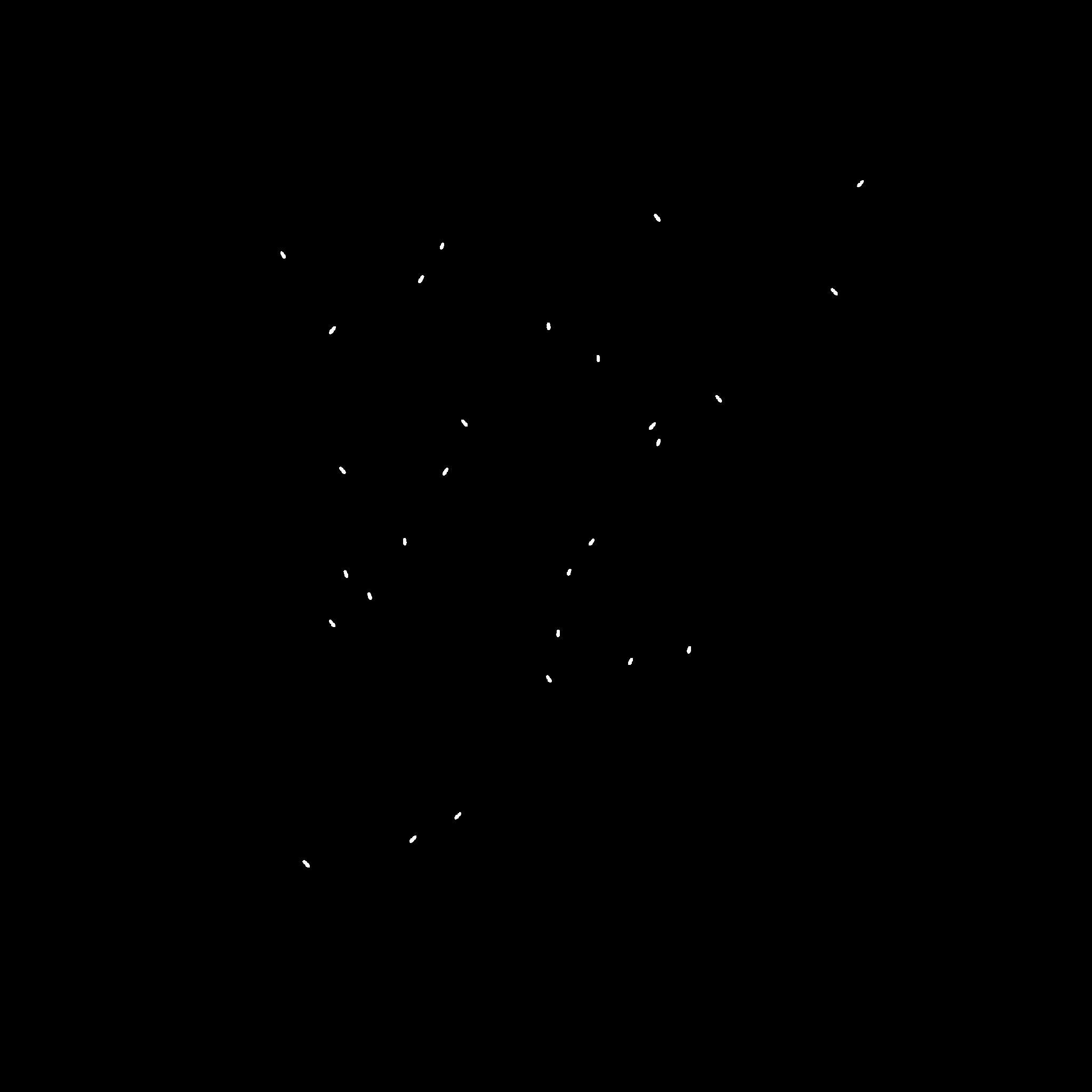

Supplement: S1 File — (ZIP) [file pone.0132101.s003.zip › ORsrc/nonortho/simu028/camx/imx177.jpg]

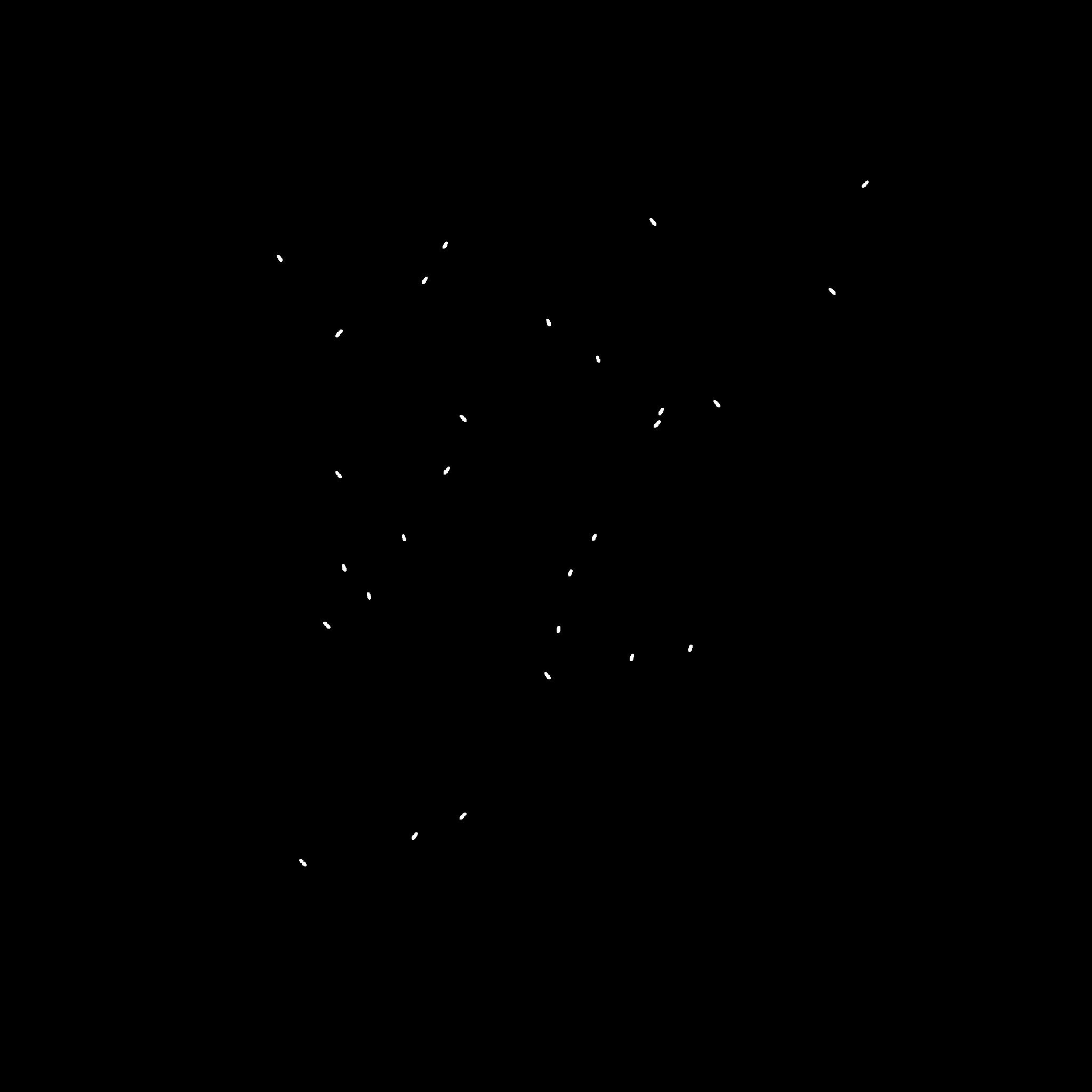

Supplement: S1 File — (ZIP) [file pone.0132101.s003.zip › ORsrc/nonortho/simu028/camx/imx178.jpg]

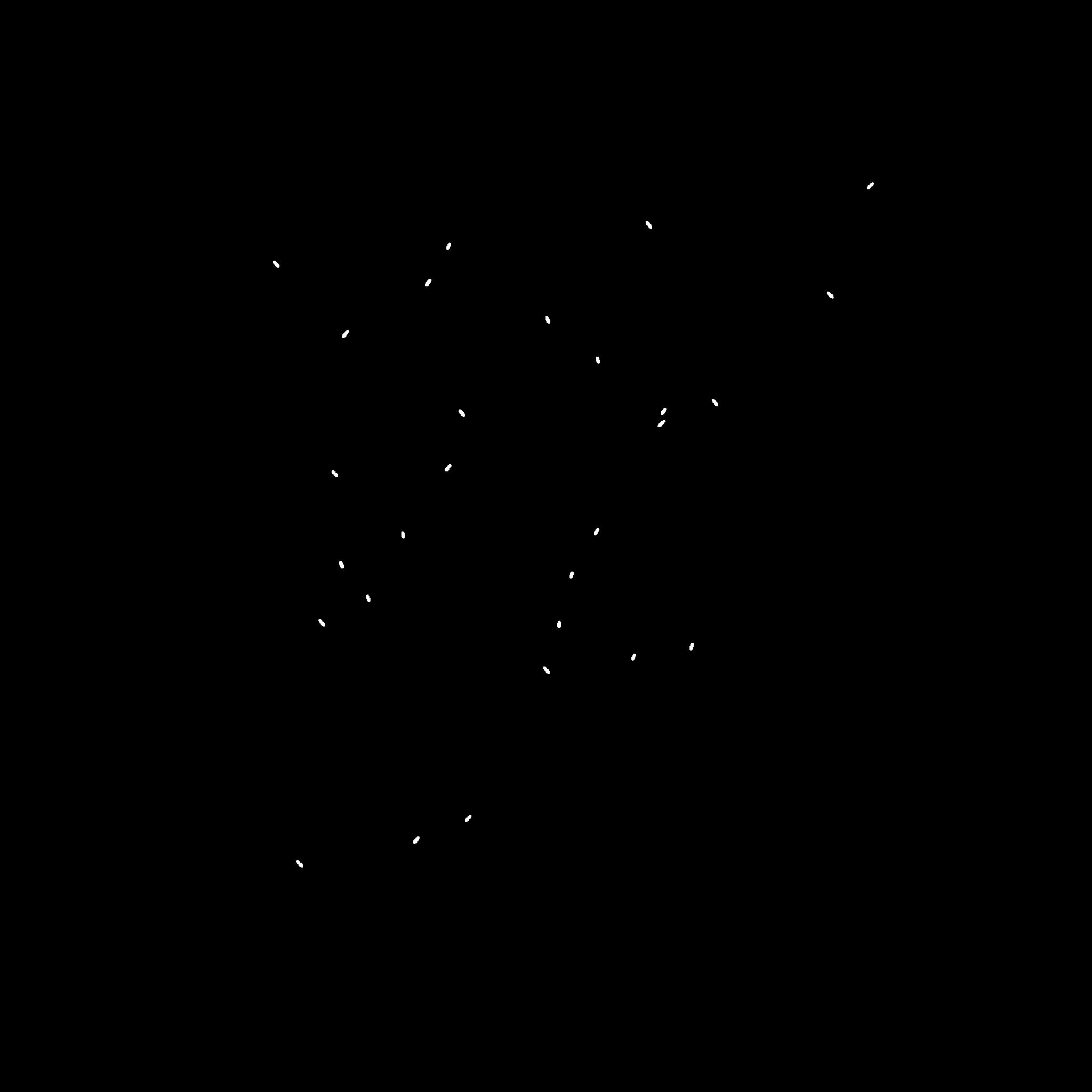

Supplement: S1 File — (ZIP) [file pone.0132101.s003.zip › ORsrc/nonortho/simu028/camx/imx179.jpg]

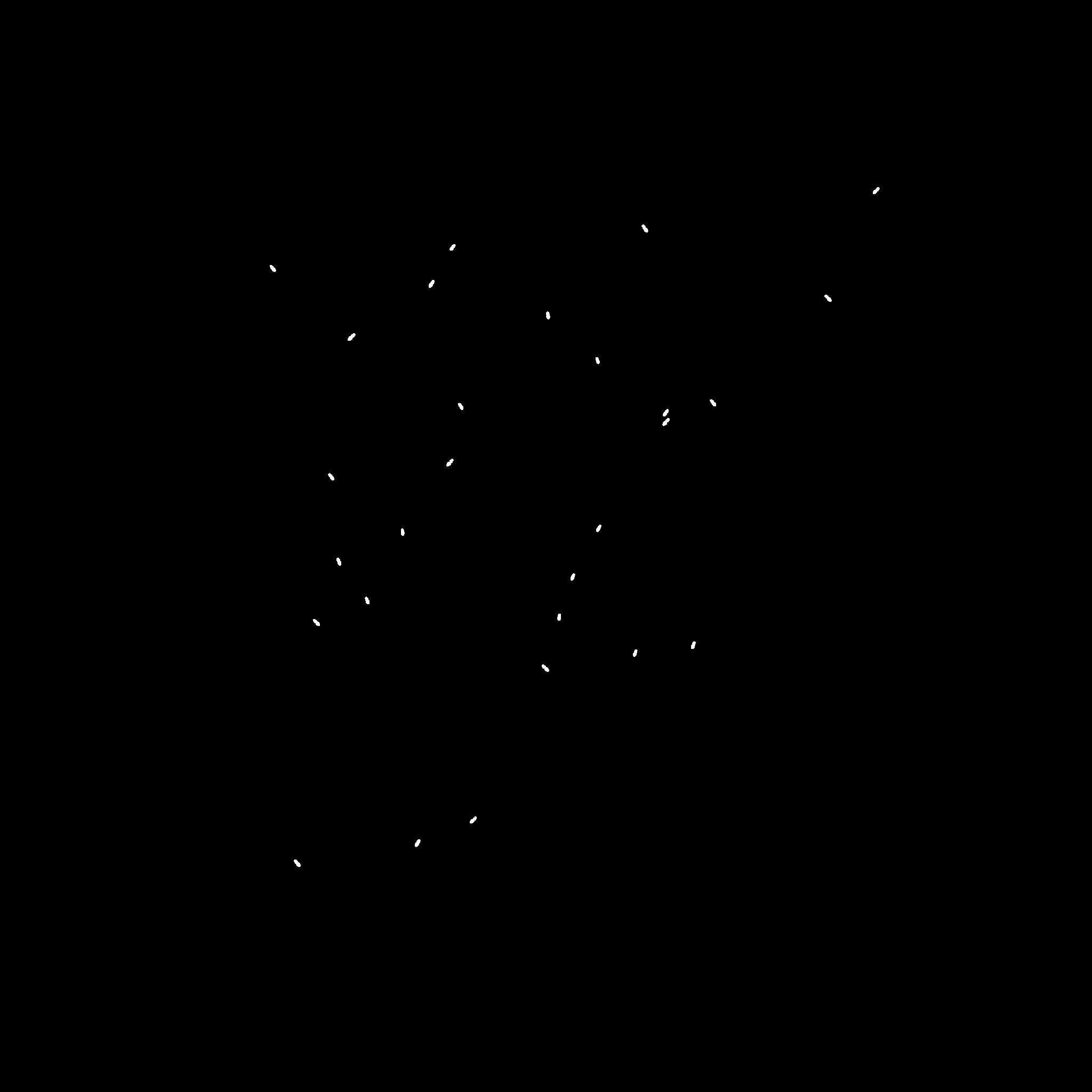

Supplement: S1 File — (ZIP) [file pone.0132101.s003.zip › ORsrc/nonortho/simu028/camx/imx180.jpg]

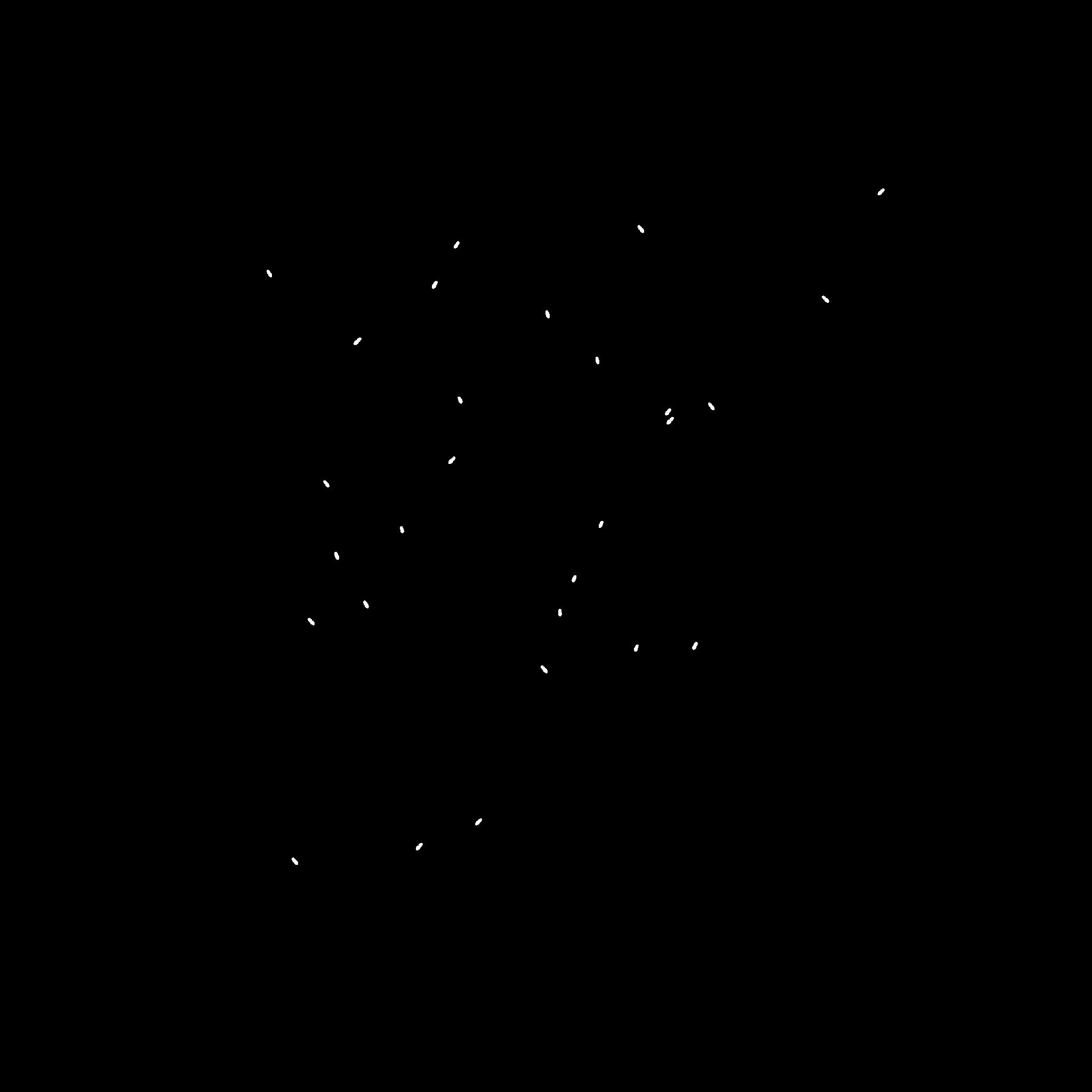

Supplement: S1 File — (ZIP) [file pone.0132101.s003.zip › ORsrc/nonortho/simu028/camx/imx181.jpg]

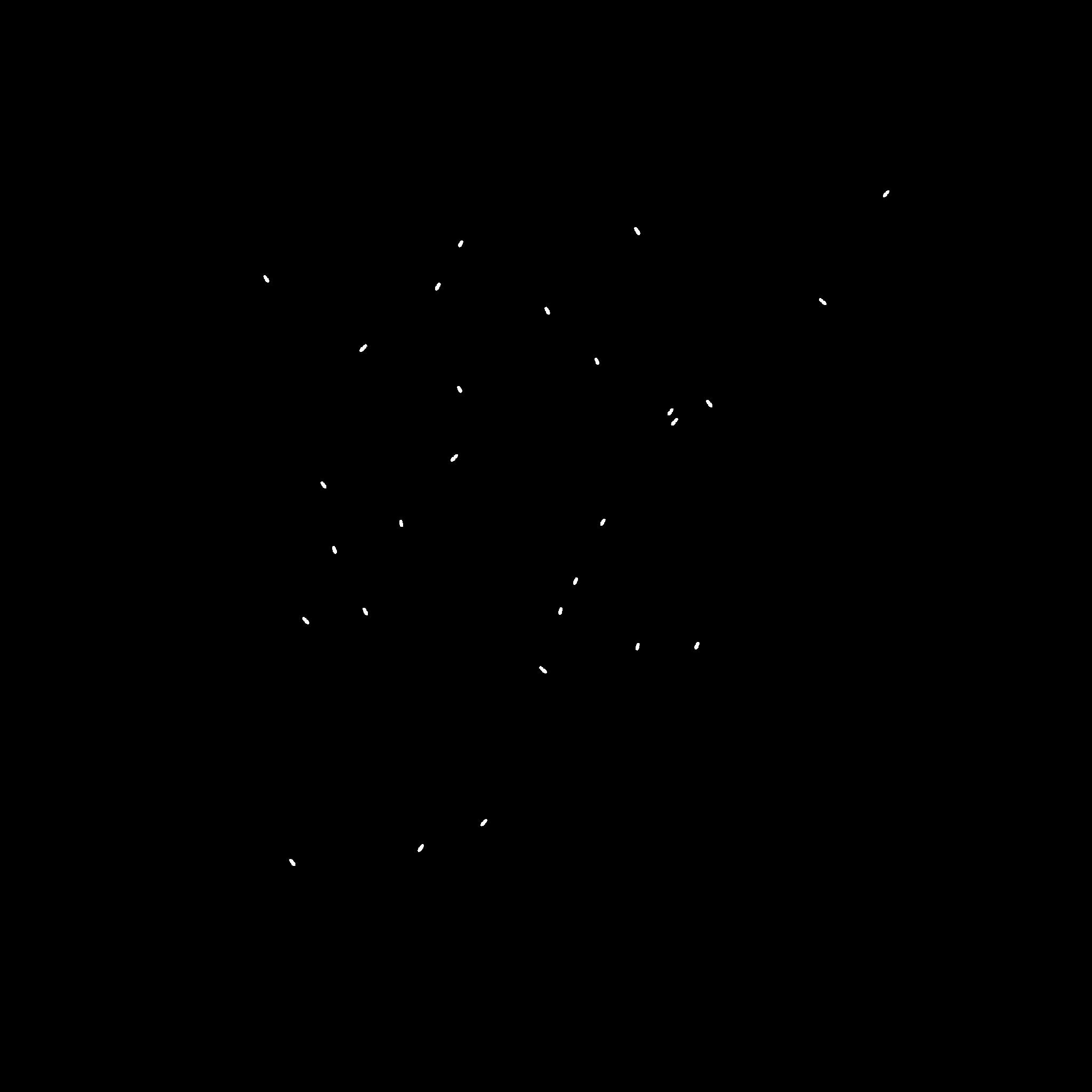

Supplement: S1 File — (ZIP) [file pone.0132101.s003.zip › ORsrc/nonortho/simu028/camx/imx182.jpg]

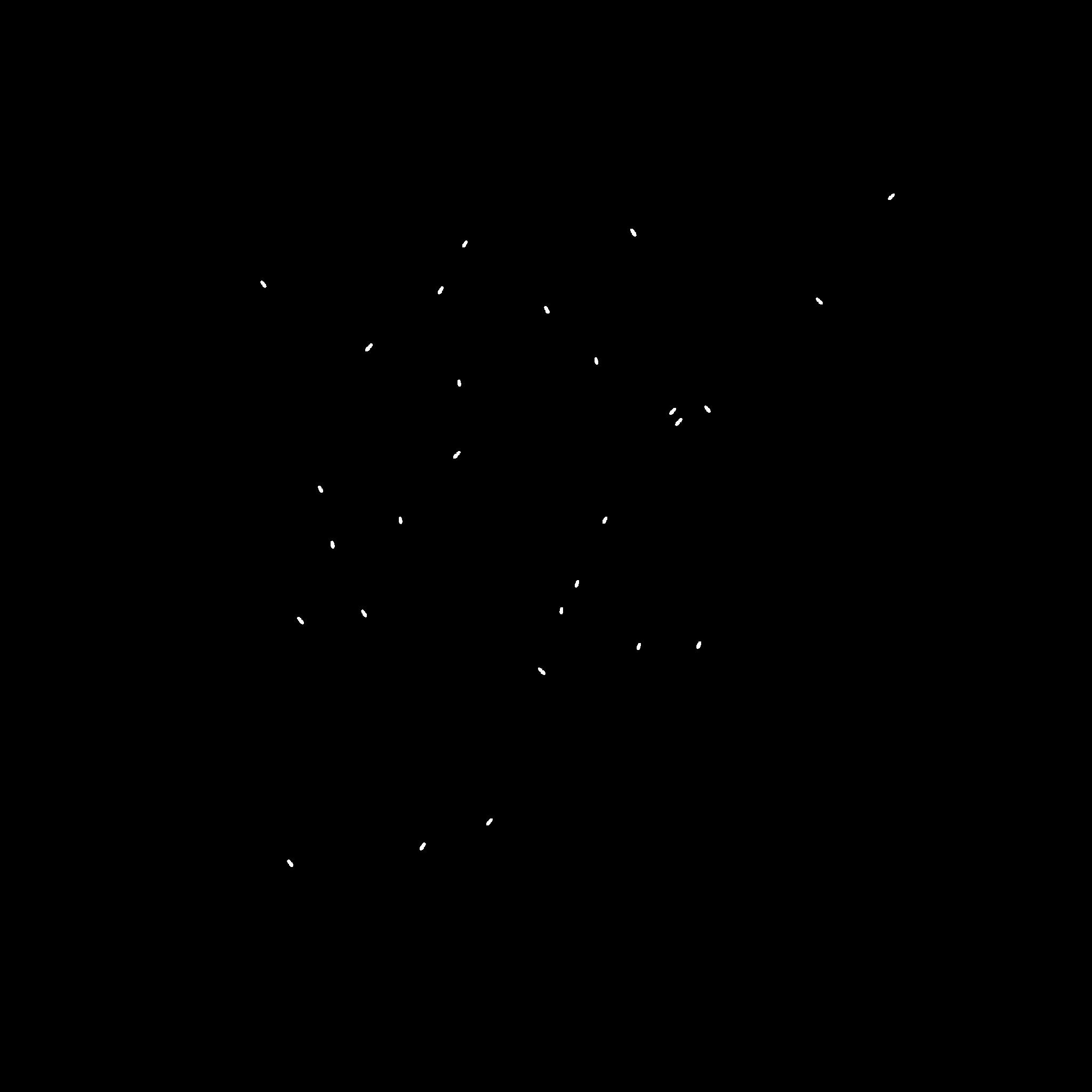

Supplement: S1 File — (ZIP) [file pone.0132101.s003.zip › ORsrc/nonortho/simu028/camx/imx183.jpg]

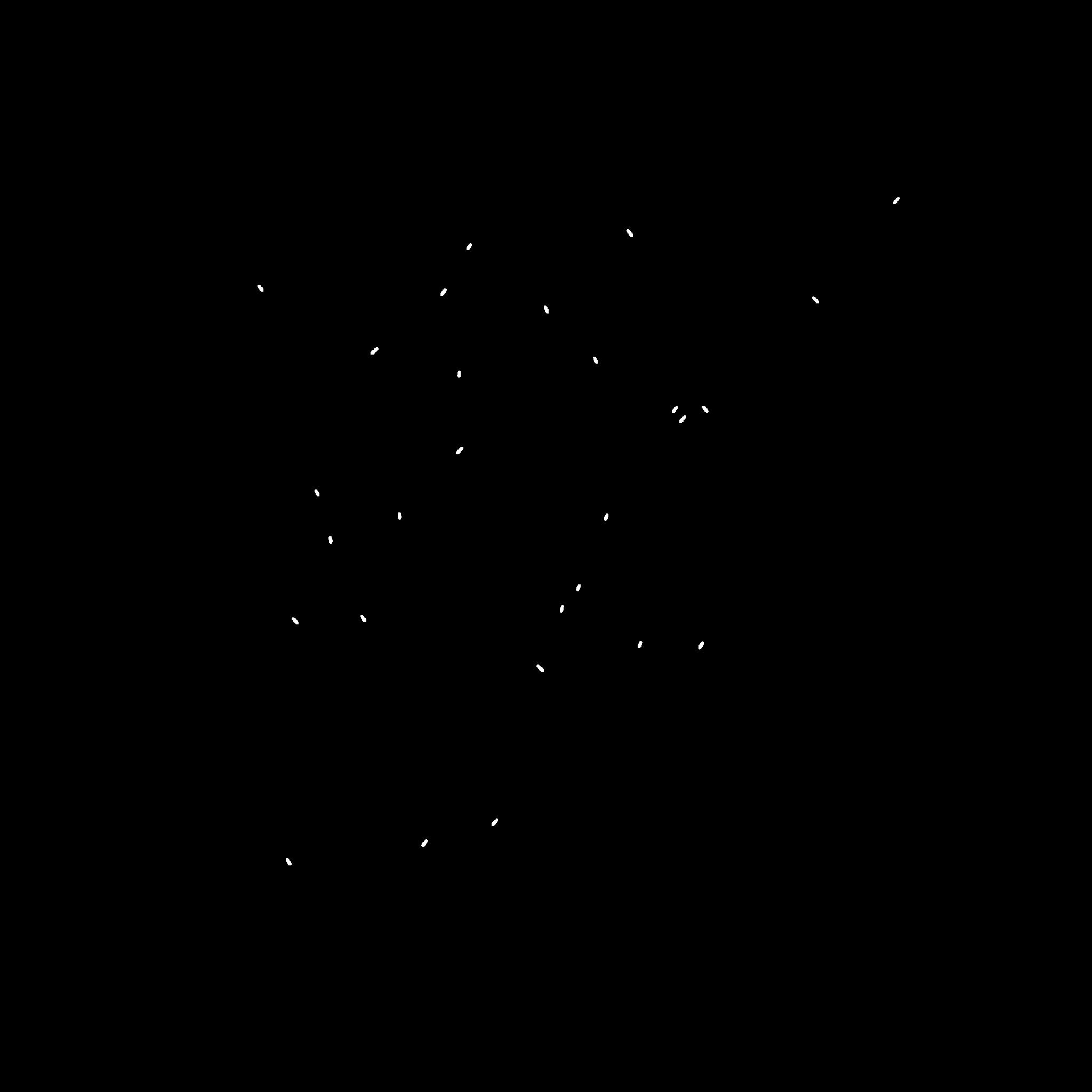

Supplement: S1 File — (ZIP) [file pone.0132101.s003.zip › ORsrc/nonortho/simu028/camx/imx184.jpg]

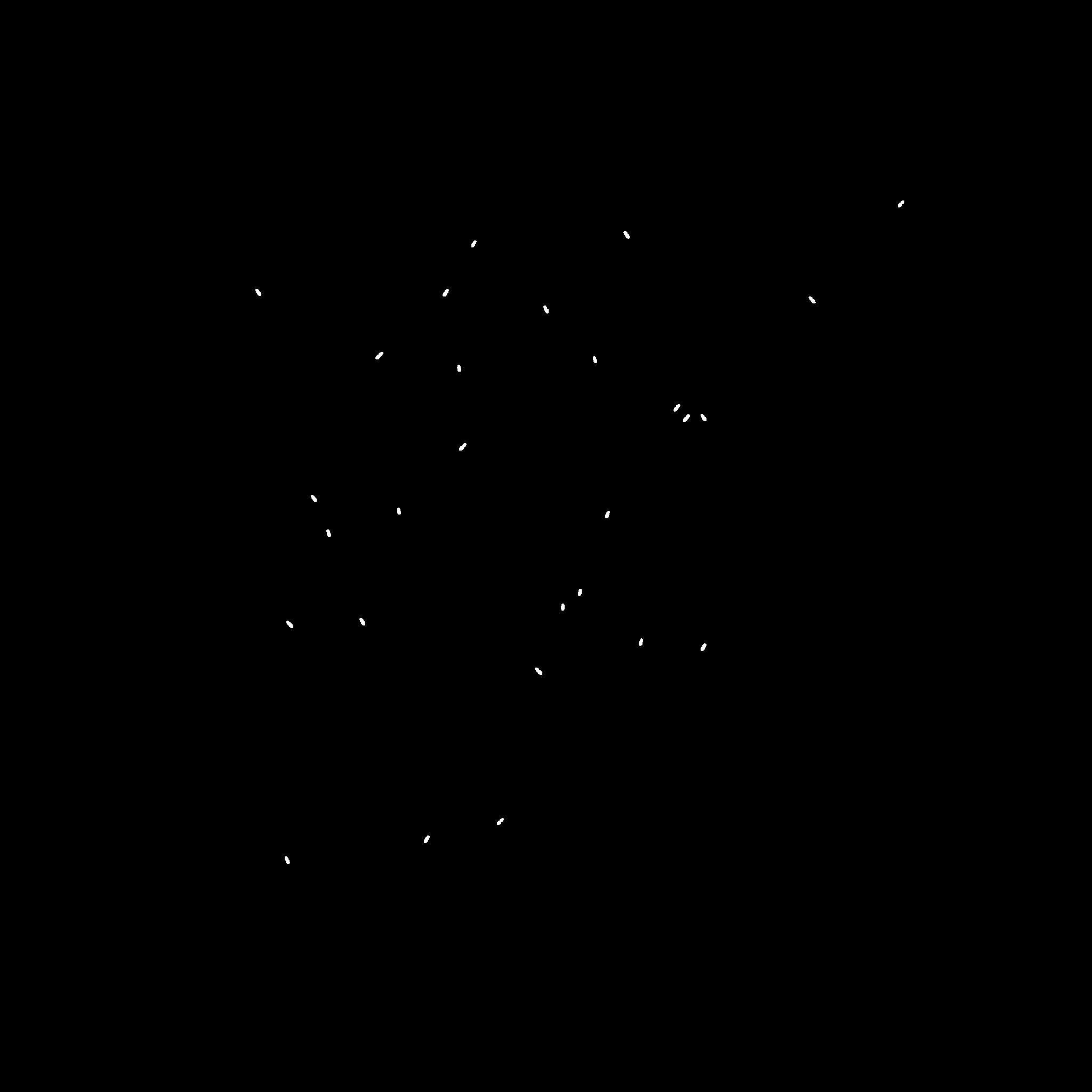

Supplement: S1 File — (ZIP) [file pone.0132101.s003.zip › ORsrc/nonortho/simu028/camx/imx185.jpg]

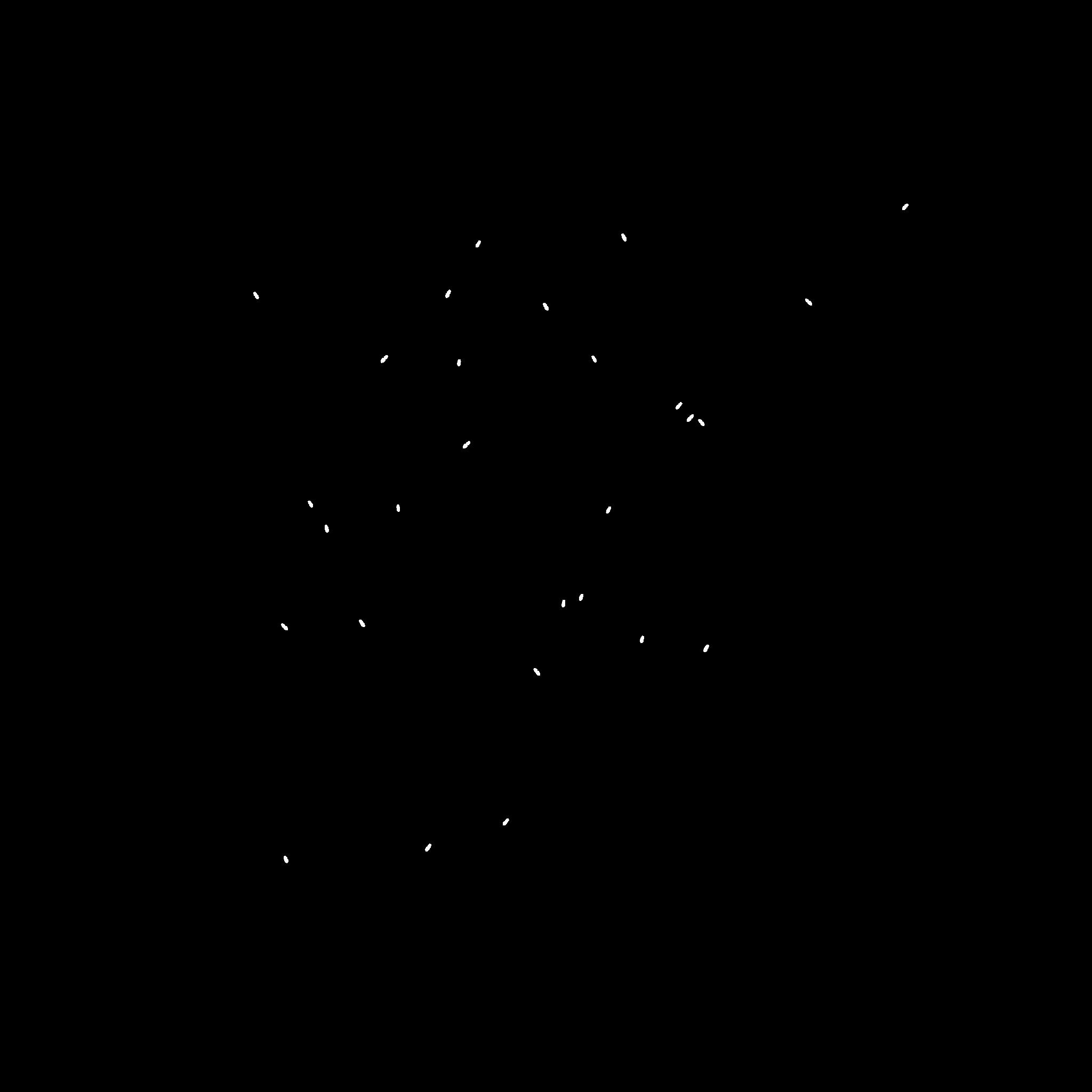

Supplement: S1 File — (ZIP) [file pone.0132101.s003.zip › ORsrc/nonortho/simu028/camx/imx186.jpg]

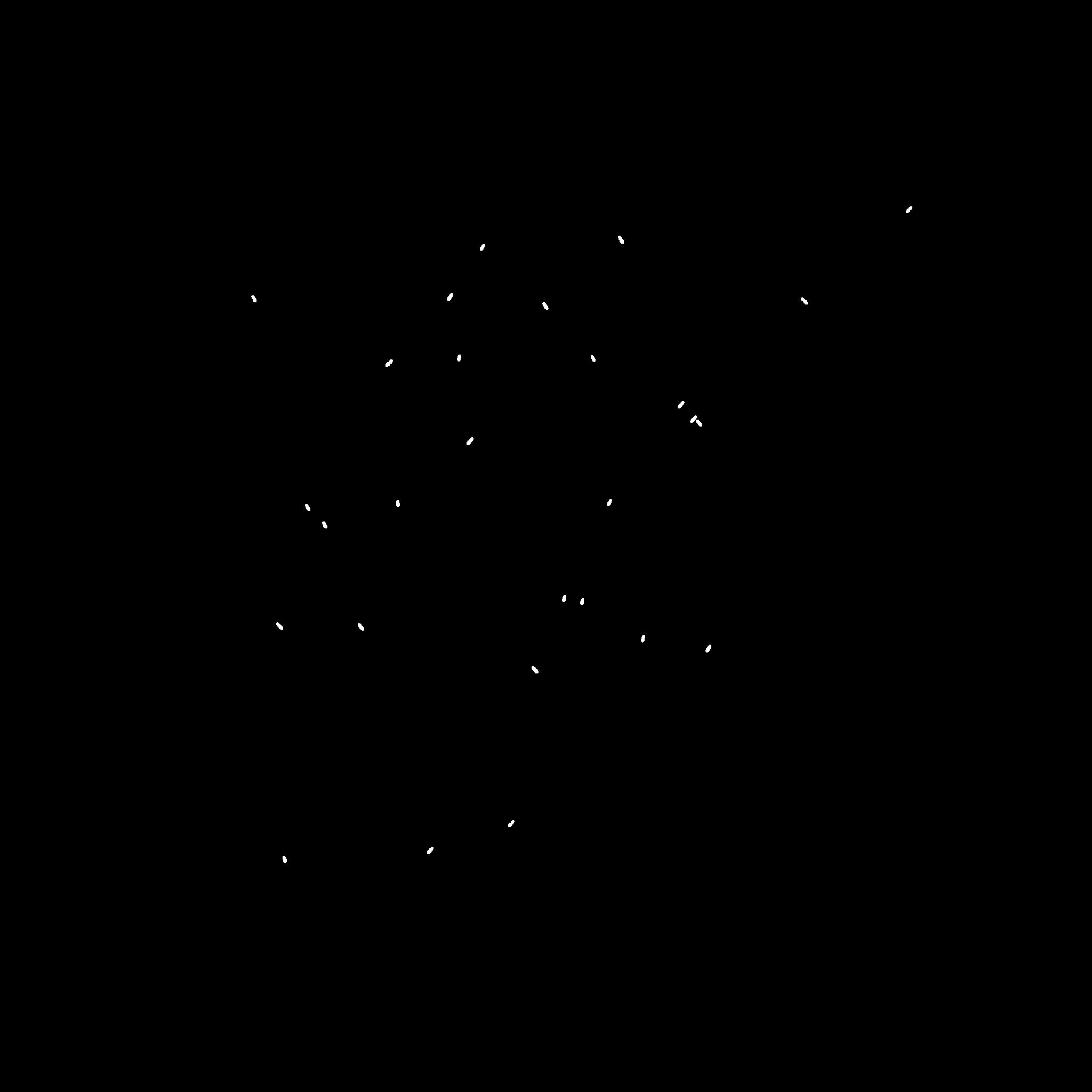

Supplement: S1 File — (ZIP) [file pone.0132101.s003.zip › ORsrc/nonortho/simu028/camx/imx187.jpg]

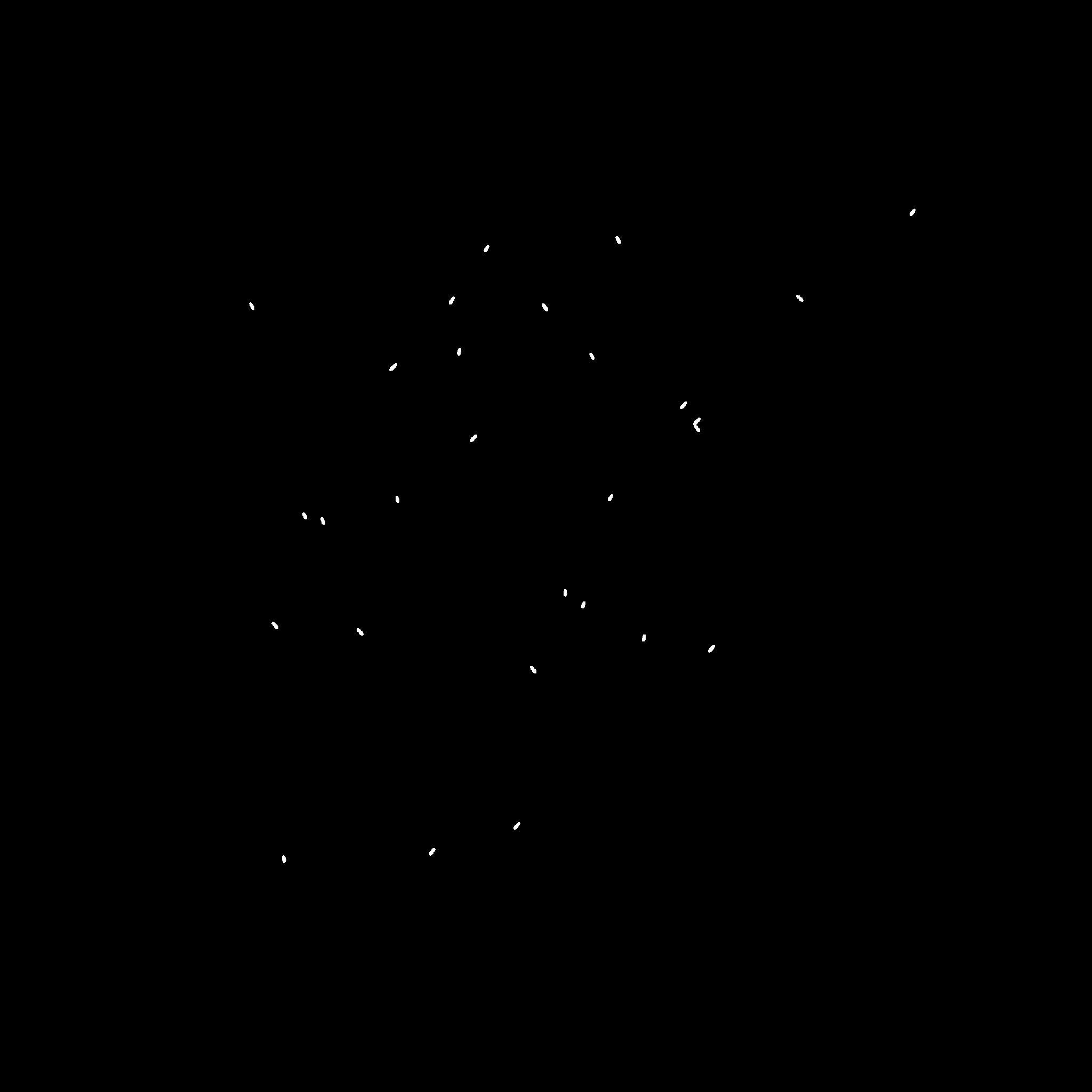

Supplement: S1 File — (ZIP) [file pone.0132101.s003.zip › ORsrc/nonortho/simu028/camx/imx188.jpg]

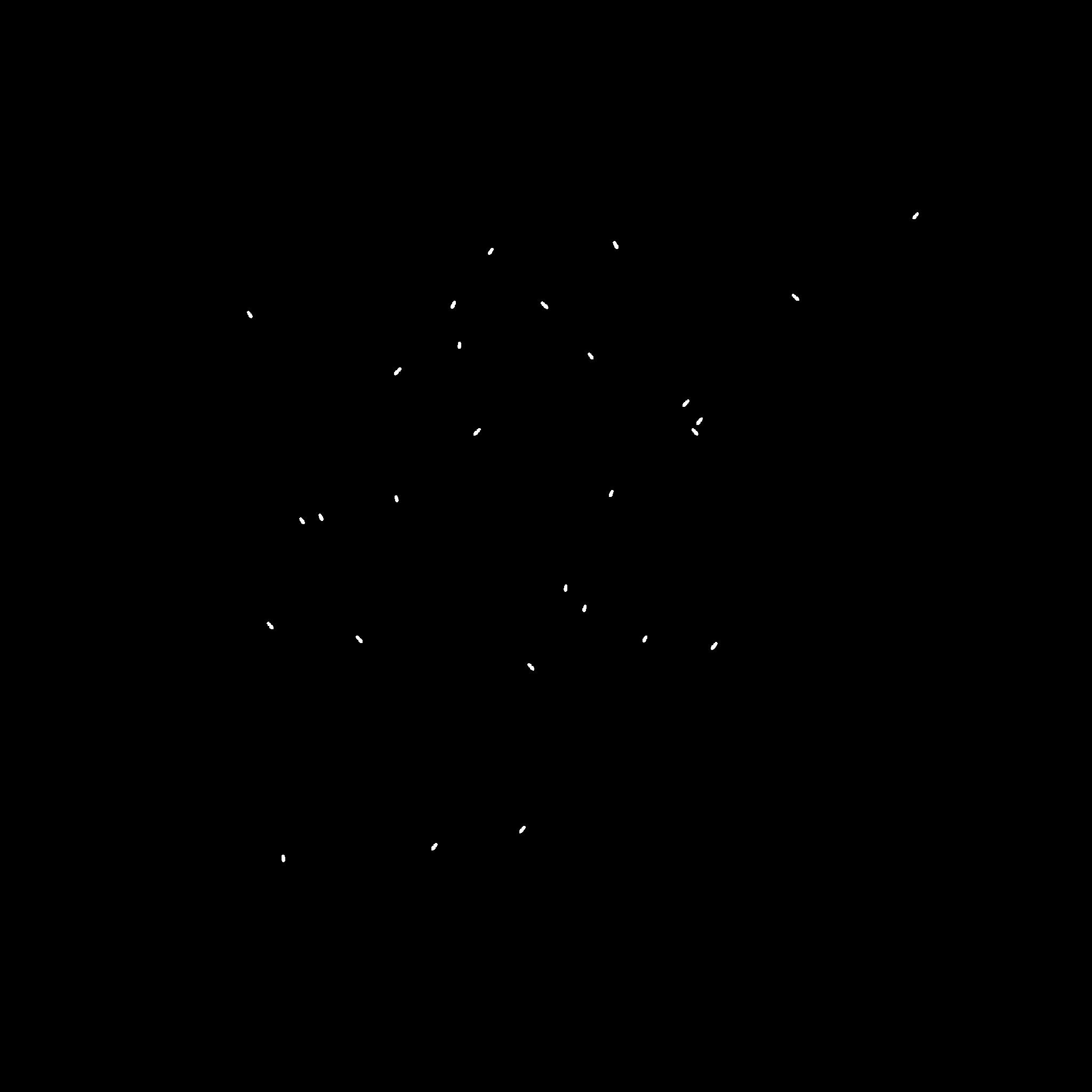

Supplement: S1 File — (ZIP) [file pone.0132101.s003.zip › ORsrc/nonortho/simu028/camx/imx189.jpg]

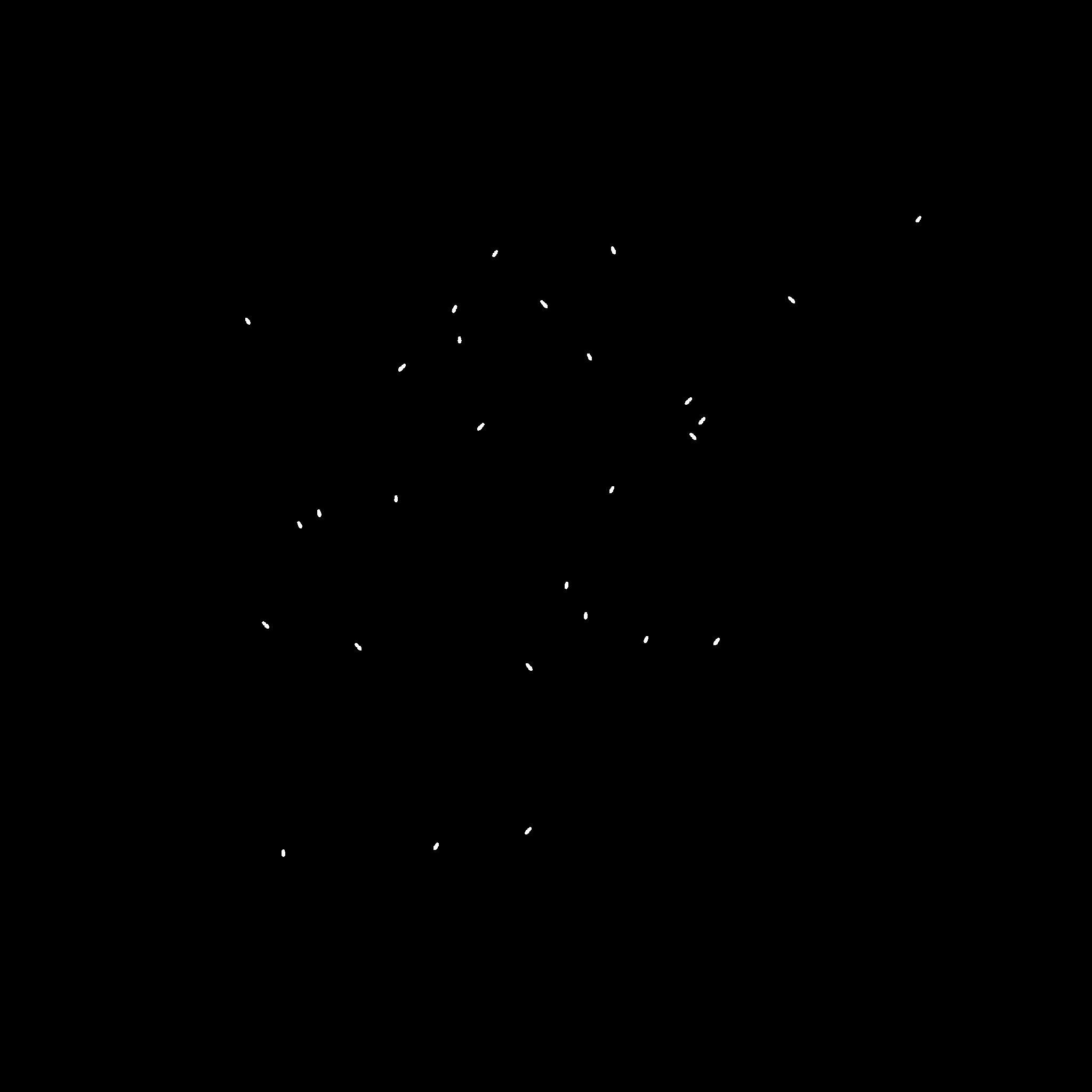

Supplement: S1 File — (ZIP) [file pone.0132101.s003.zip › ORsrc/nonortho/simu028/camx/imx190.jpg]

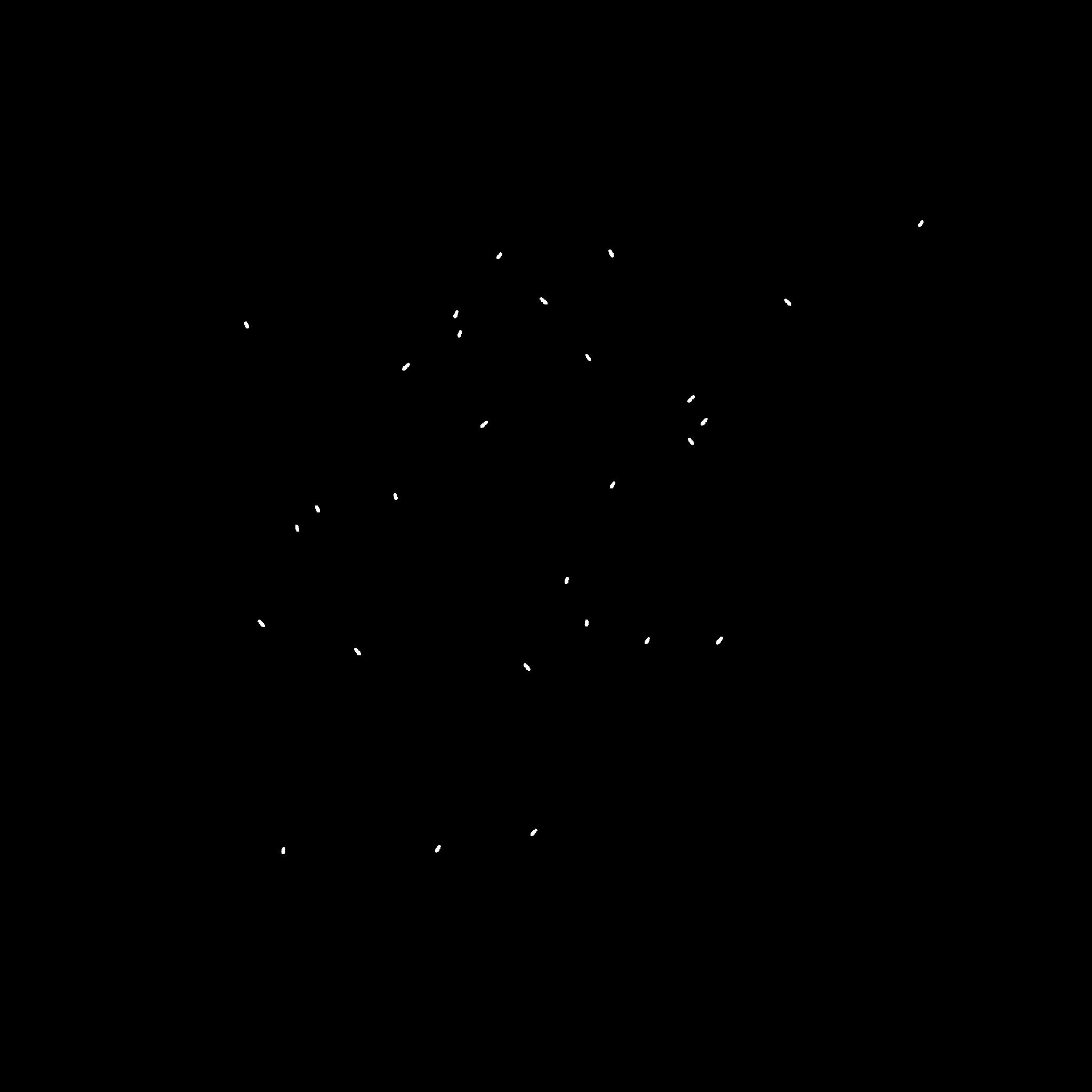

Supplement: S1 File — (ZIP) [file pone.0132101.s003.zip › ORsrc/nonortho/simu028/camx/imx191.jpg]

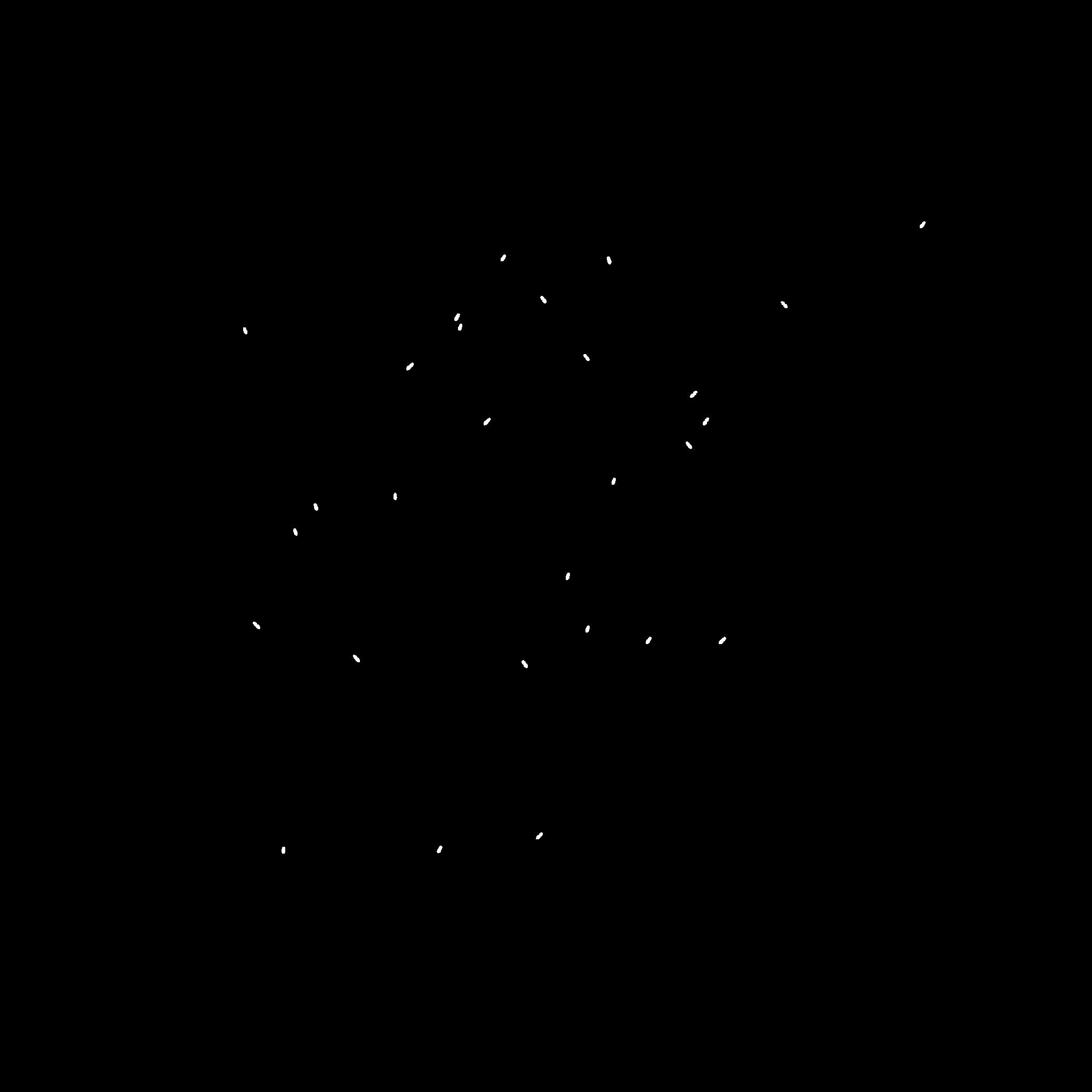

Supplement: S1 File — (ZIP) [file pone.0132101.s003.zip › ORsrc/nonortho/simu028/camx/imx192.jpg]

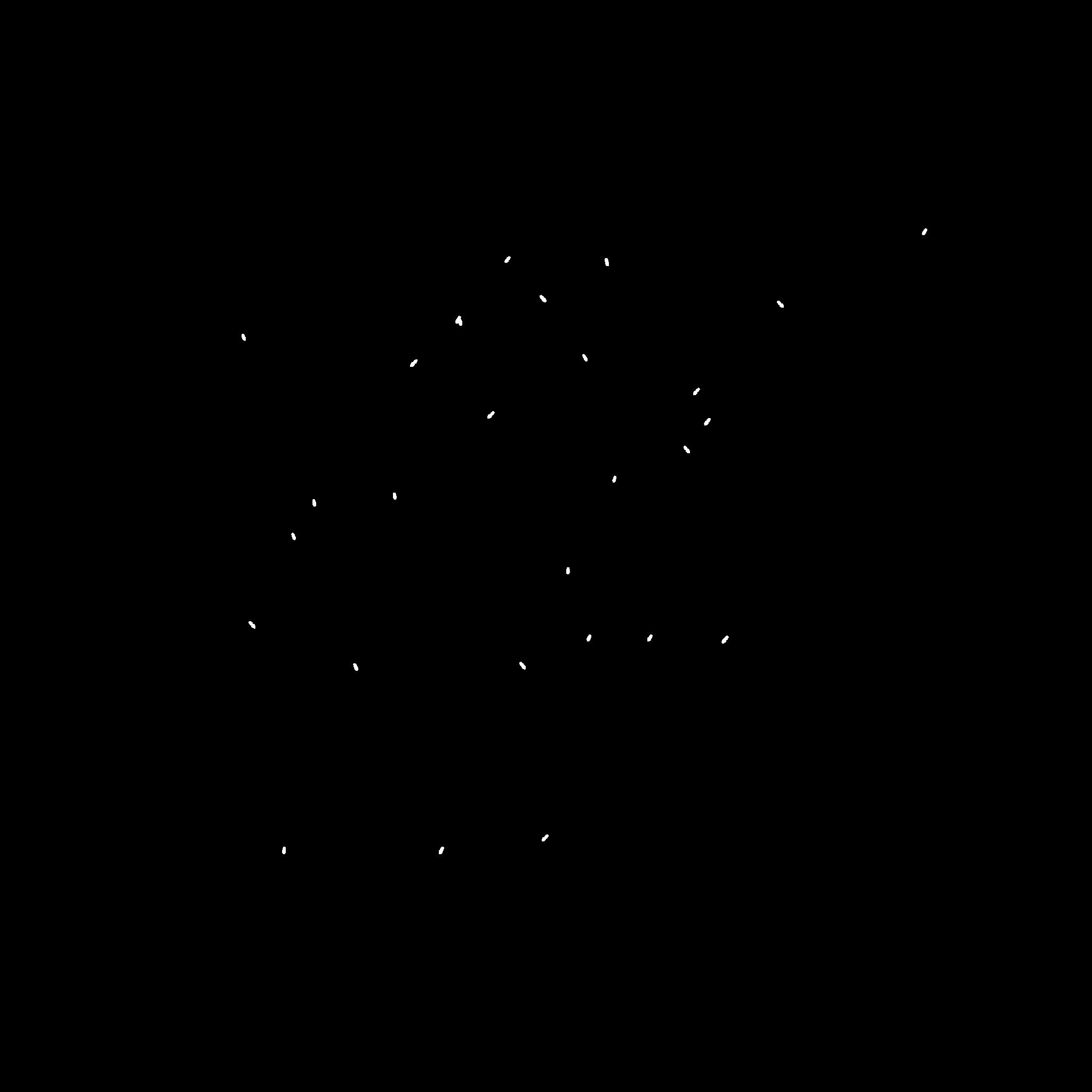

Supplement: S1 File — (ZIP) [file pone.0132101.s003.zip › ORsrc/nonortho/simu028/camx/imx193.jpg]

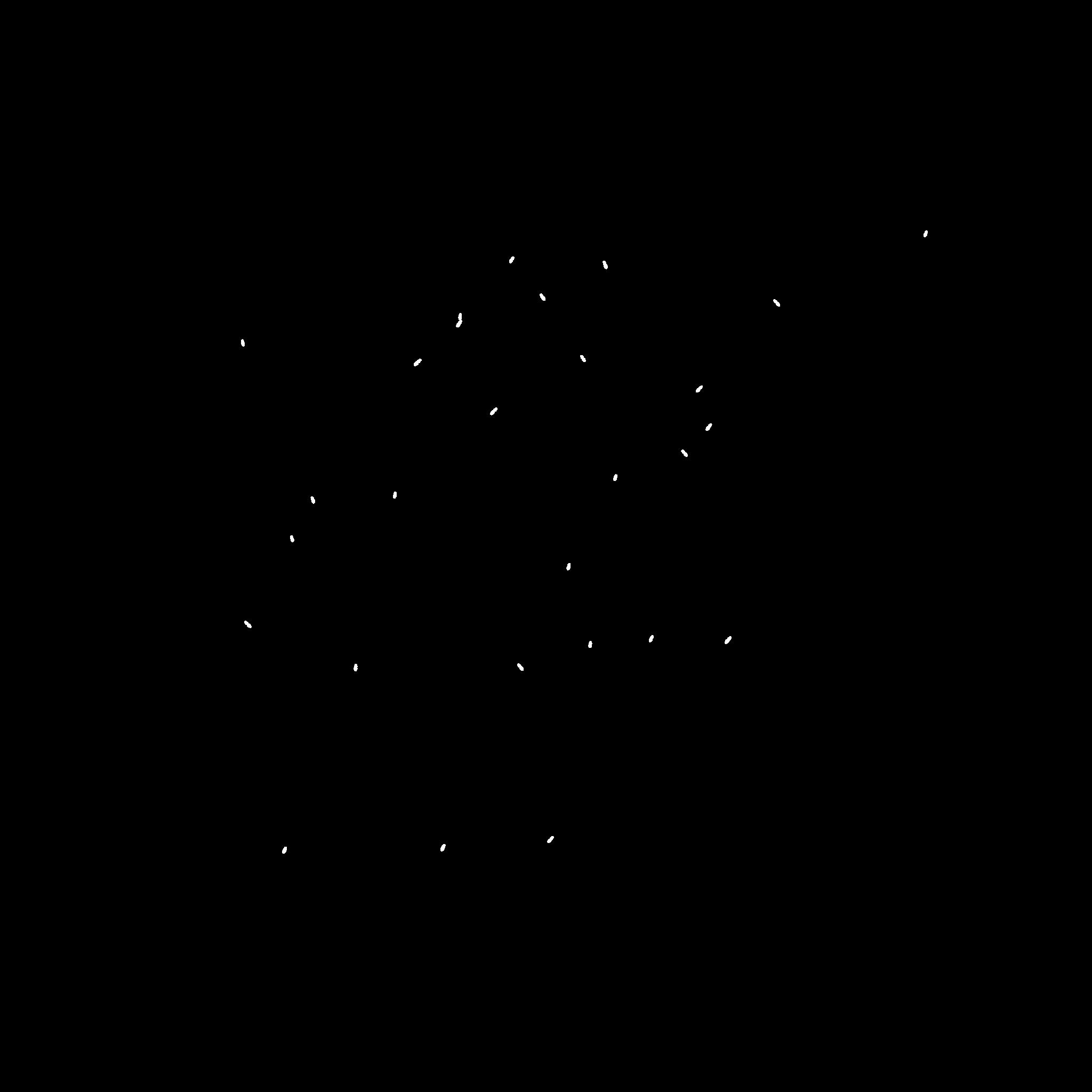

Supplement: S1 File — (ZIP) [file pone.0132101.s003.zip › ORsrc/nonortho/simu028/camx/imx194.jpg]

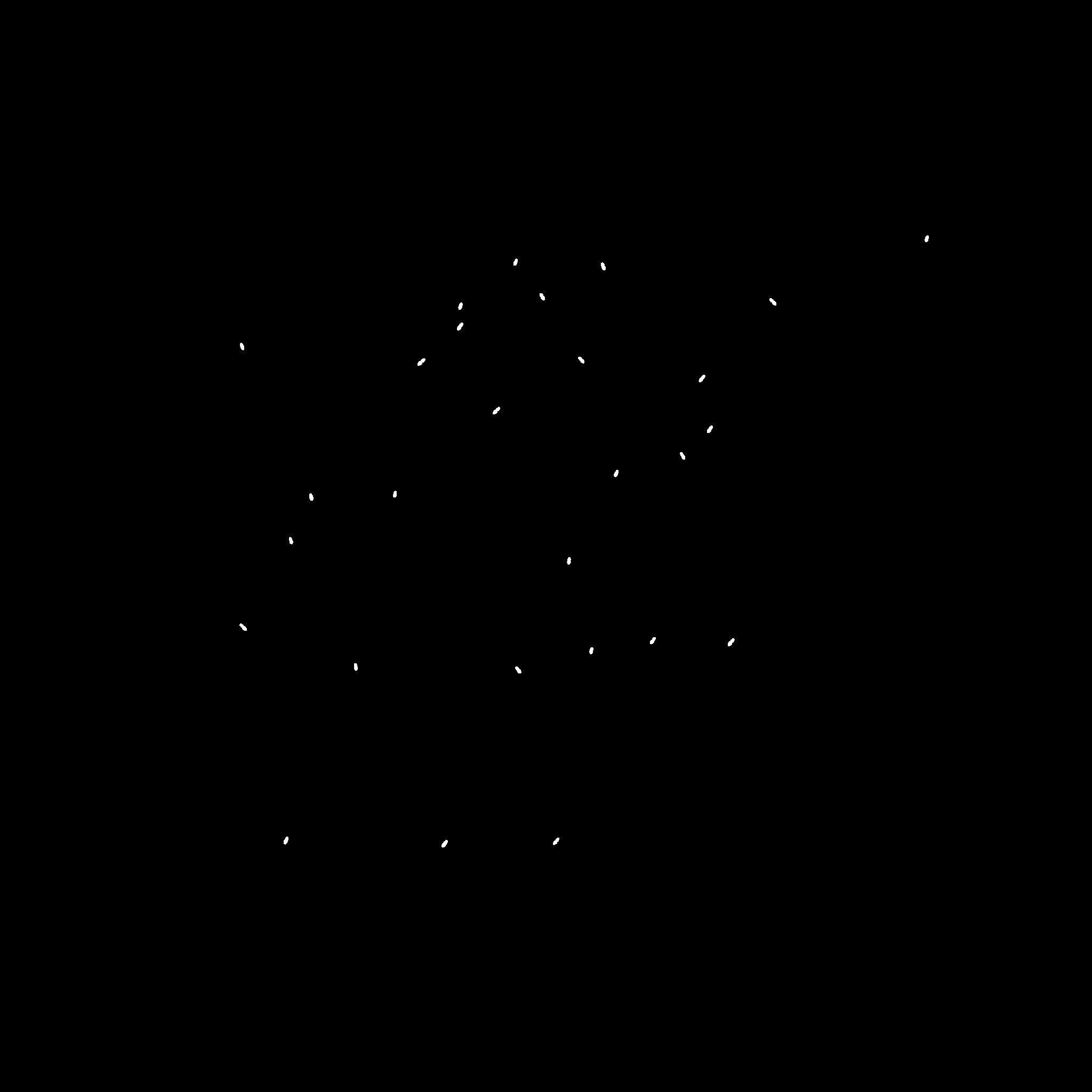

Supplement: S1 File — (ZIP) [file pone.0132101.s003.zip › ORsrc/nonortho/simu028/camx/imx195.jpg]
