# Supplementary figures and images for: Estimating Orientation of Flying Fruit Flies (part 1 of 2)
Source: PLoS One. 2015 Jul 14;10(7):e0132101. doi: 10.1371/journal.pone.0132101 (PMC4501570; doi:10.1371/journal.pone.0132101)

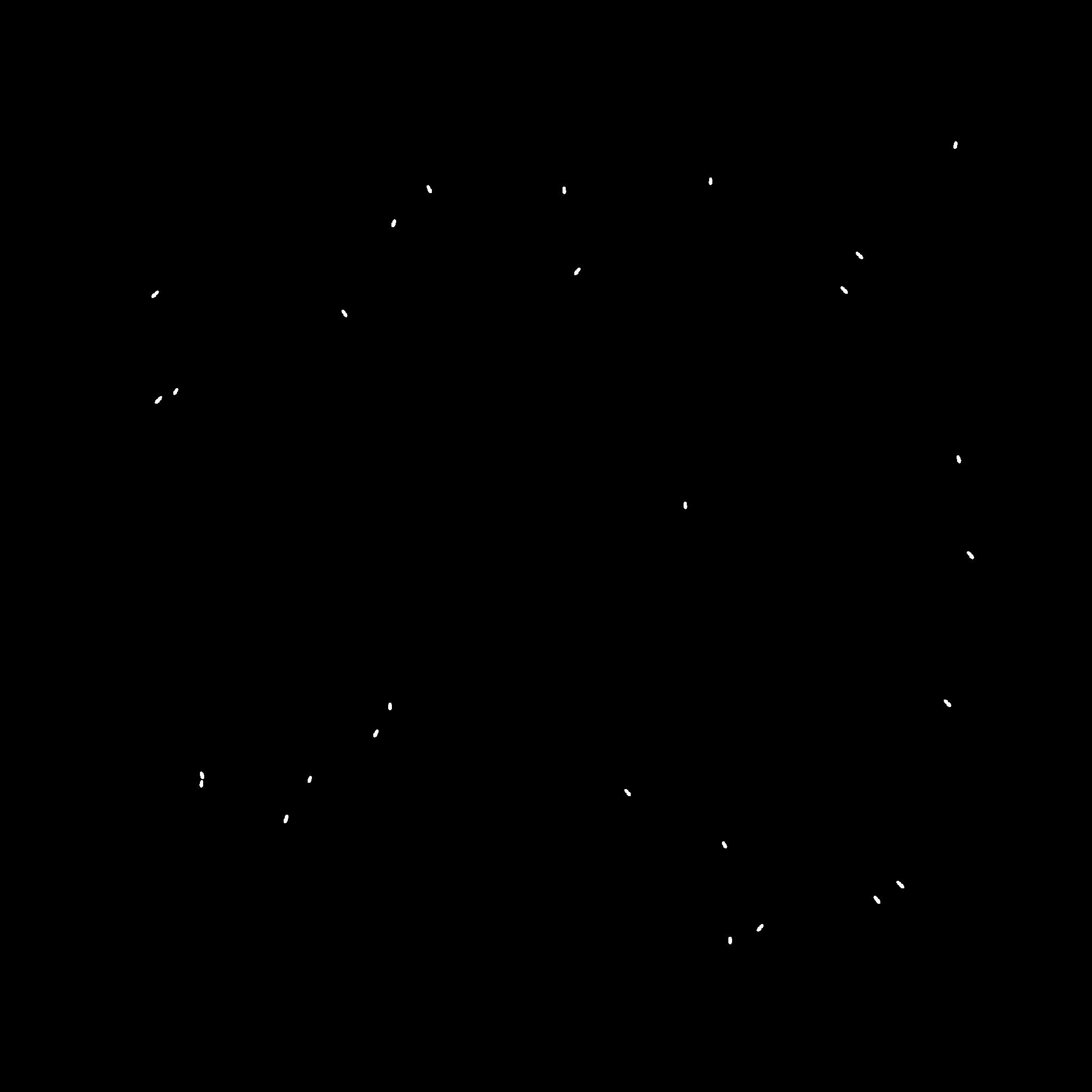

Supplement: S1 File — (ZIP) [file pone.0132101.s003.zip › ORsrc/nonortho/simu028/camx/imx003.jpg]

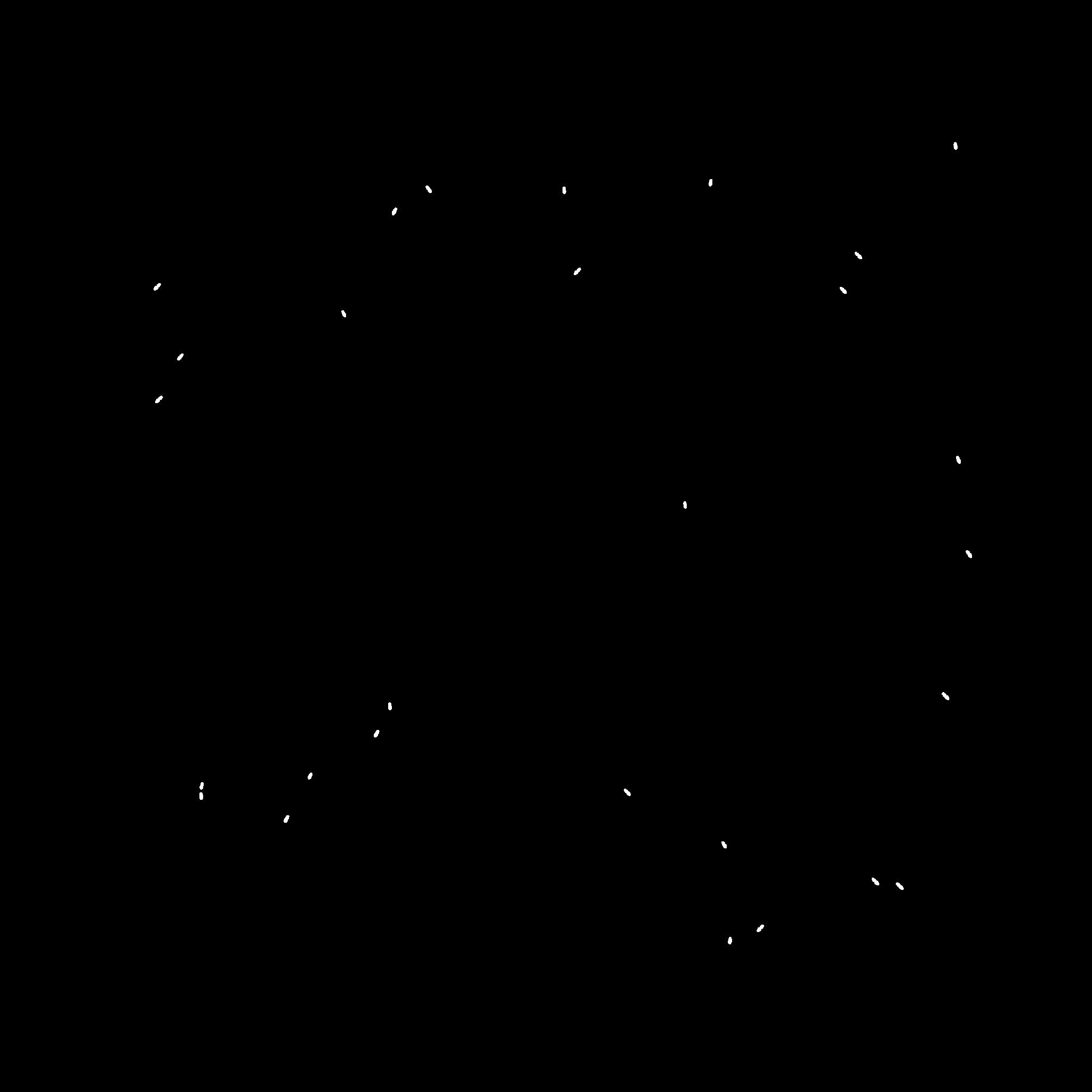

Supplement: S1 File — (ZIP) [file pone.0132101.s003.zip › ORsrc/nonortho/simu028/camx/imx004.jpg]

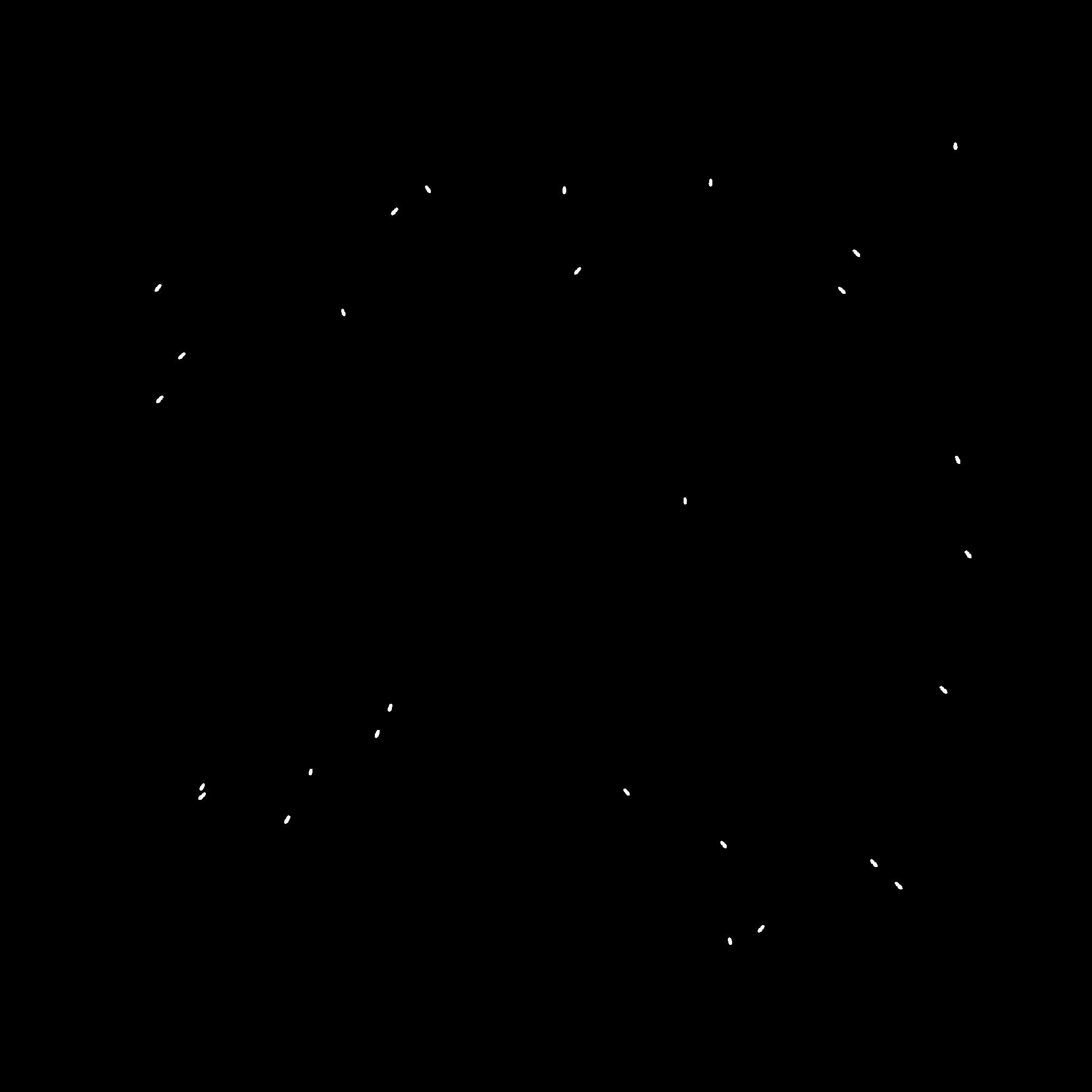

Supplement: S1 File — (ZIP) [file pone.0132101.s003.zip › ORsrc/nonortho/simu028/camx/imx005.jpg]

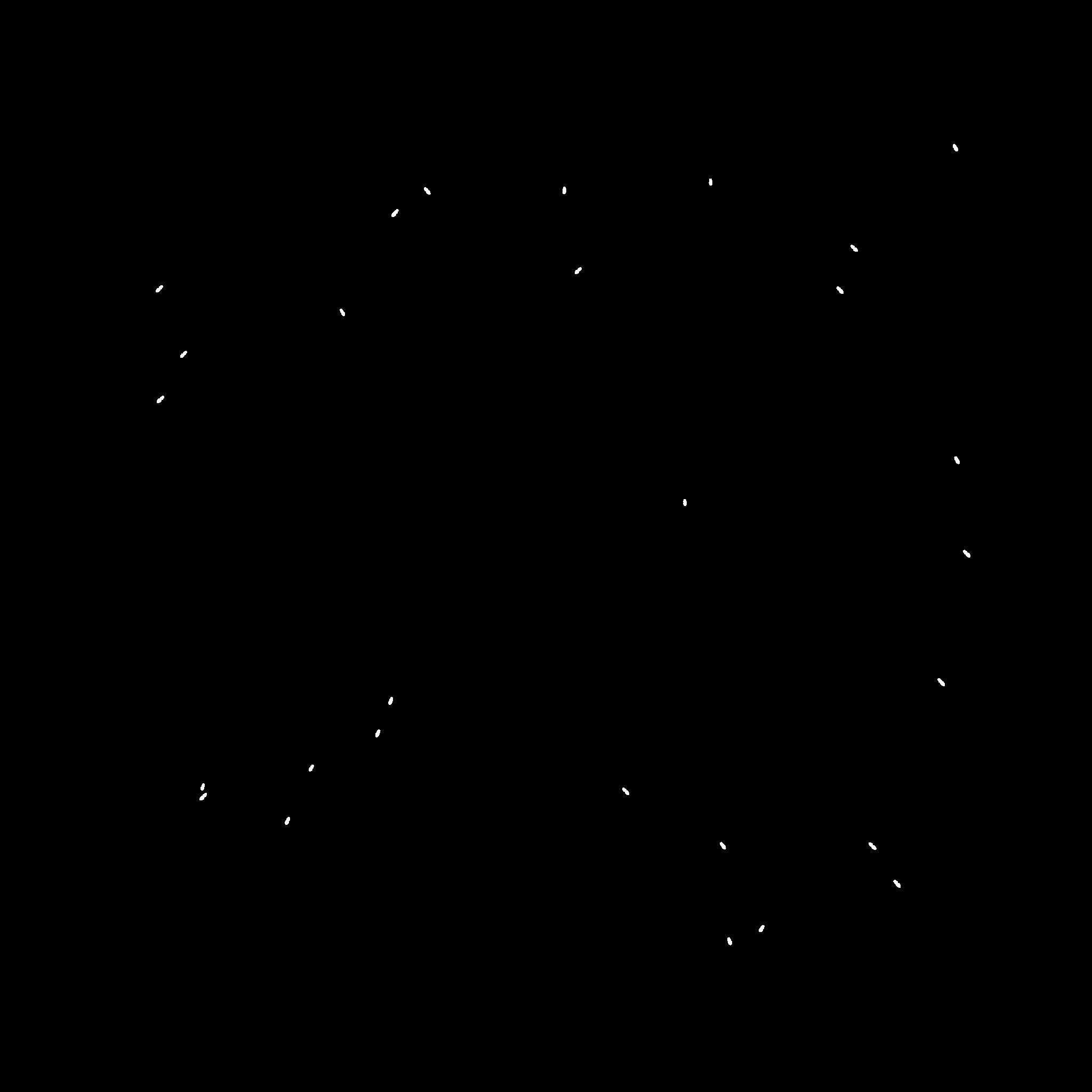

Supplement: S1 File — (ZIP) [file pone.0132101.s003.zip › ORsrc/nonortho/simu028/camx/imx006.jpg]

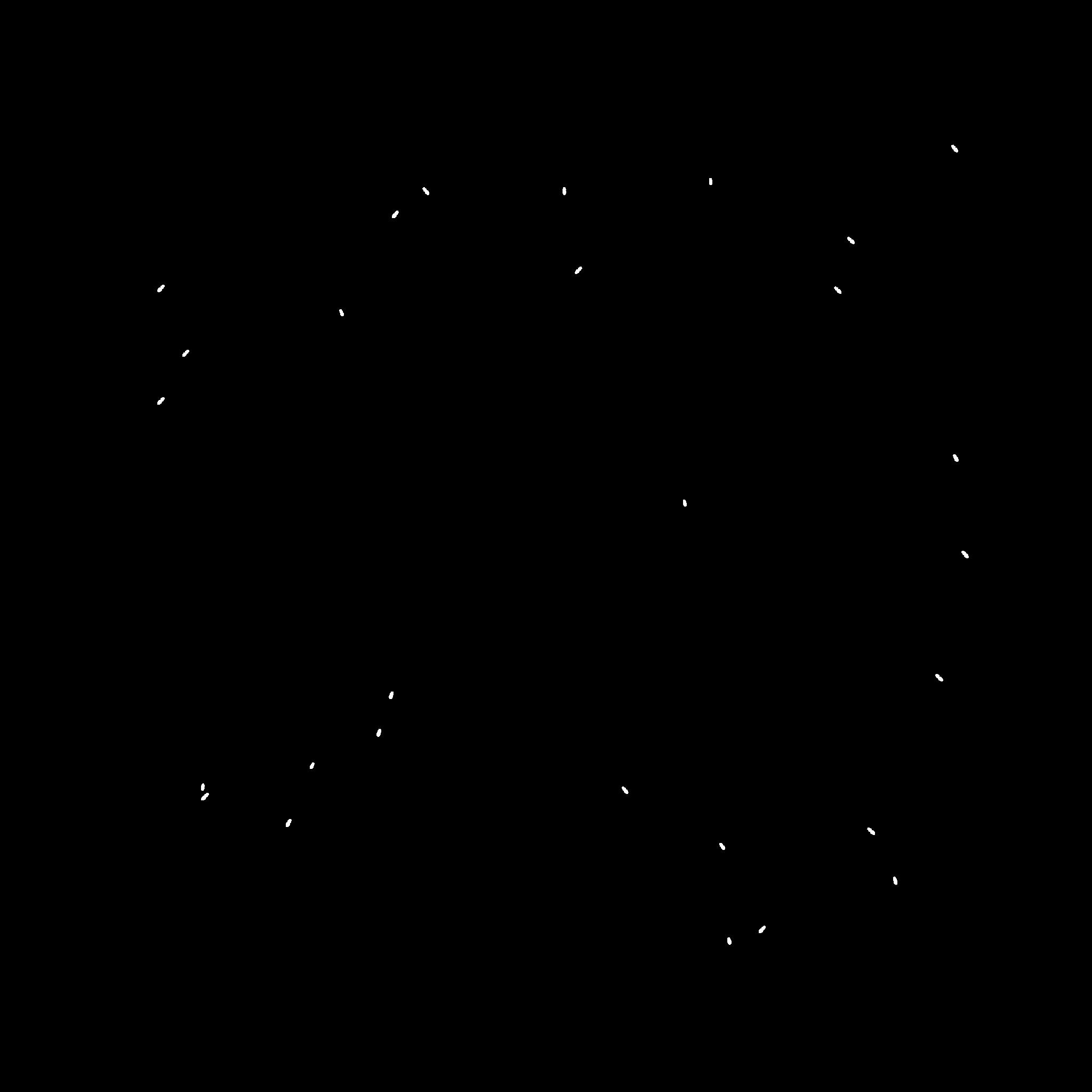

Supplement: S1 File — (ZIP) [file pone.0132101.s003.zip › ORsrc/nonortho/simu028/camx/imx007.jpg]

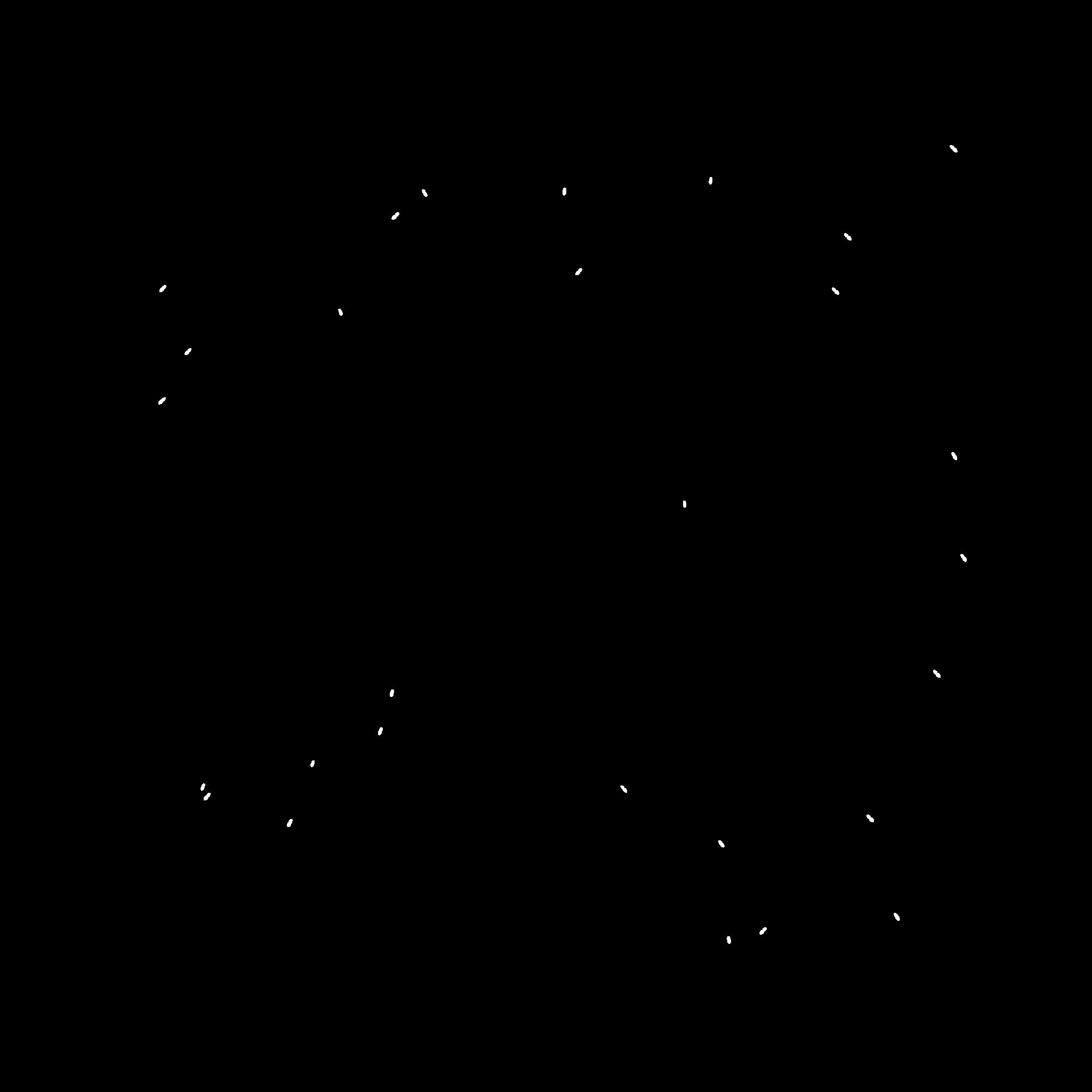

Supplement: S1 File — (ZIP) [file pone.0132101.s003.zip › ORsrc/nonortho/simu028/camx/imx008.jpg]

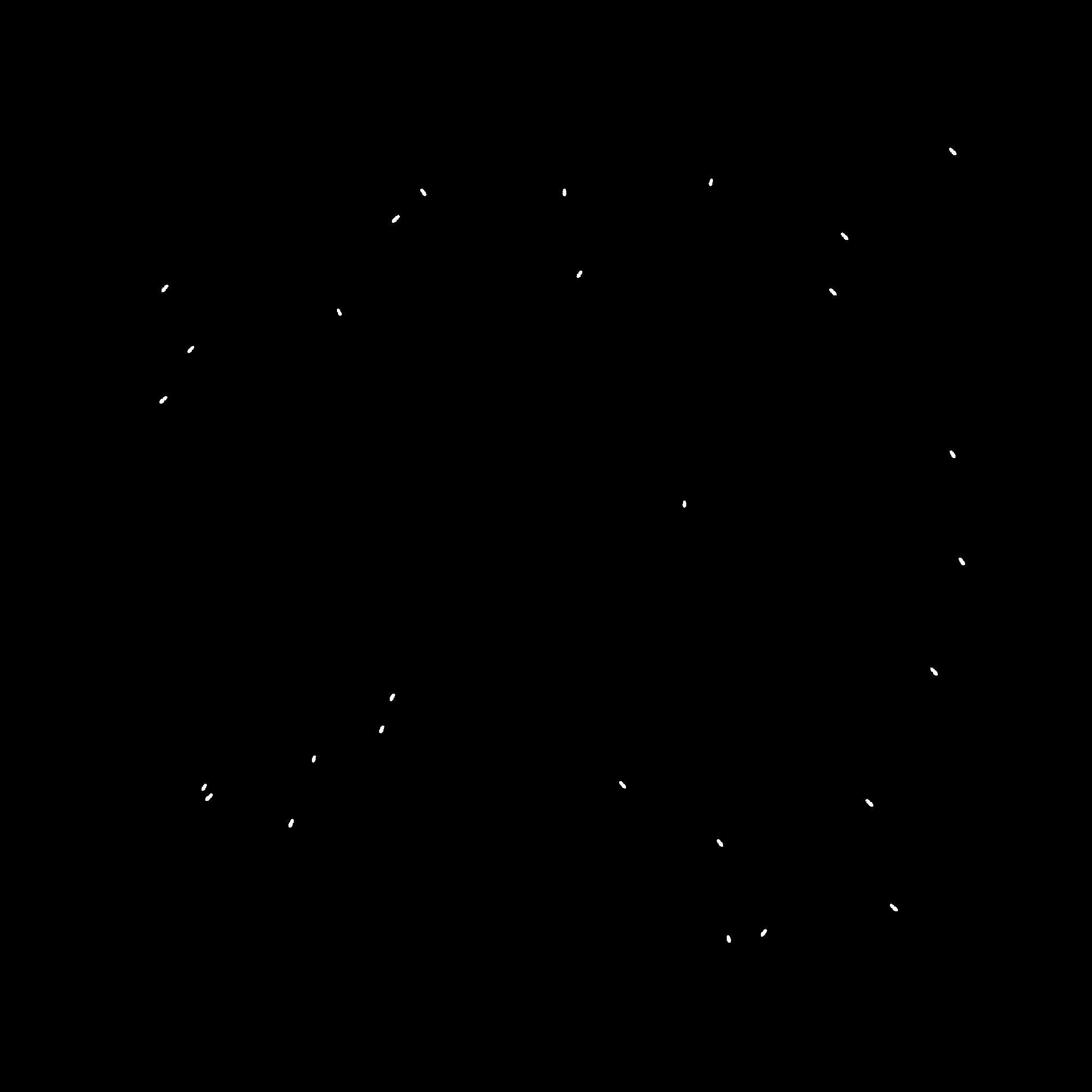

Supplement: S1 File — (ZIP) [file pone.0132101.s003.zip › ORsrc/nonortho/simu028/camx/imx009.jpg]

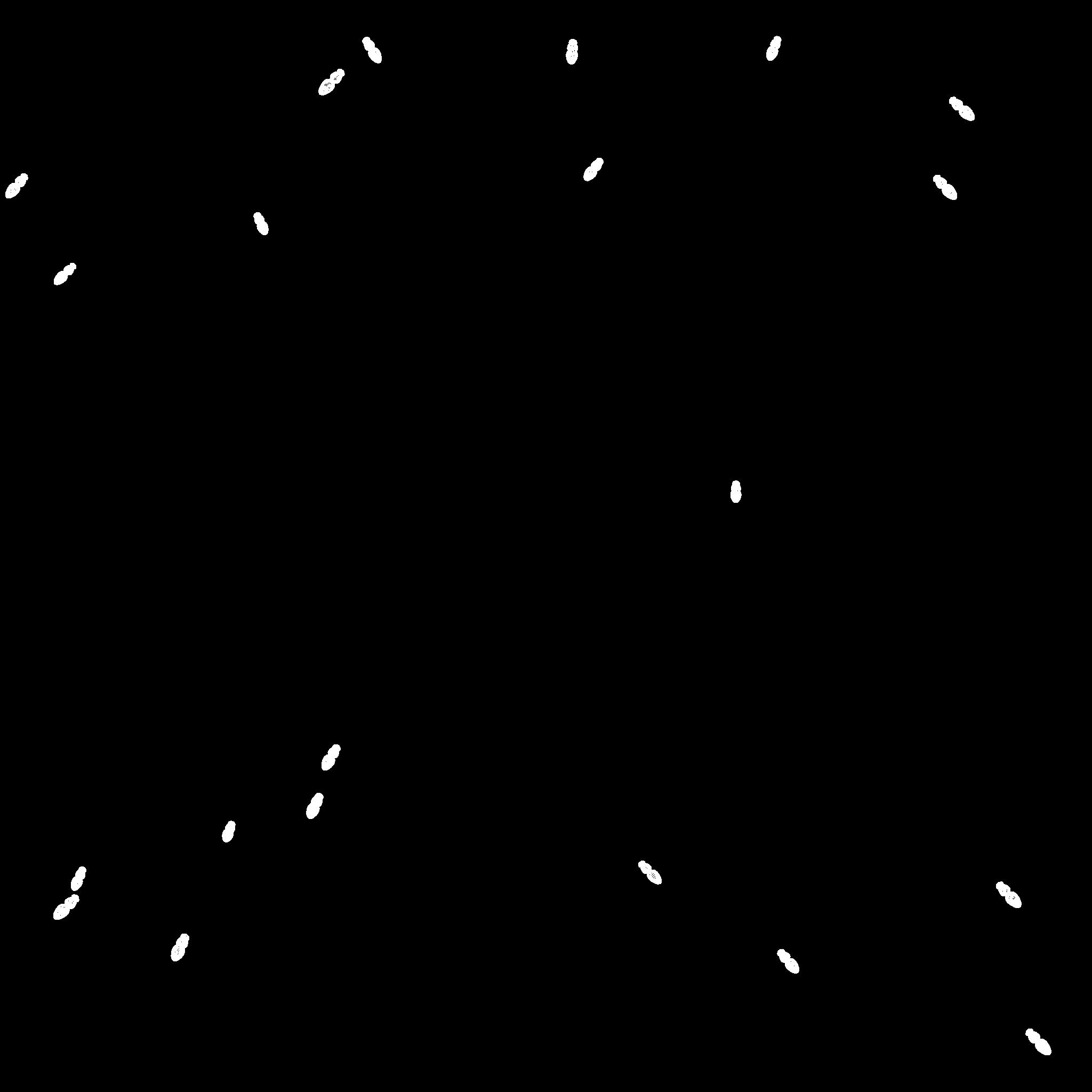

Supplement: S1 File — (ZIP) [file pone.0132101.s003.zip › ORsrc/nonortho/simu028/camx/imx010.jpg]

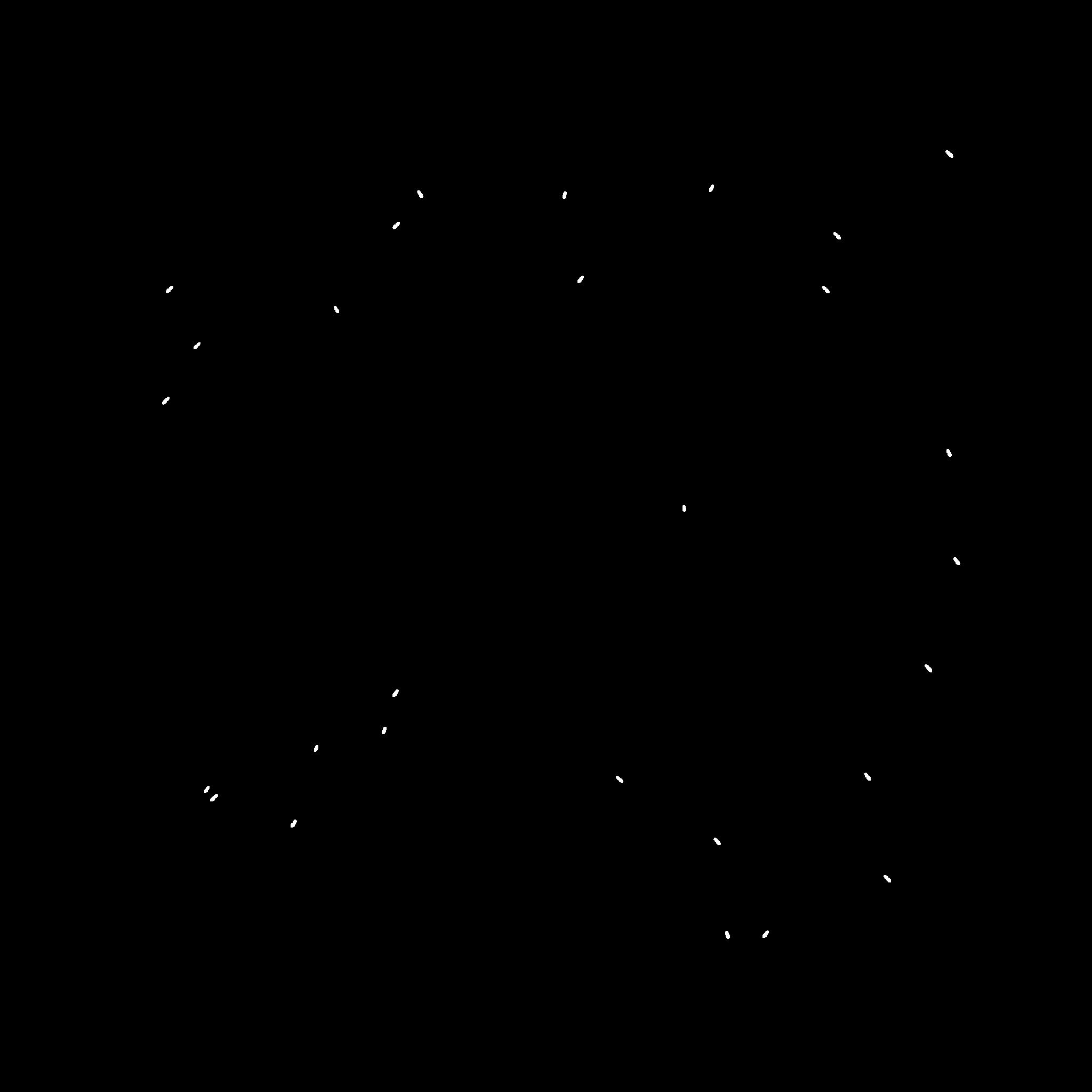

Supplement: S1 File — (ZIP) [file pone.0132101.s003.zip › ORsrc/nonortho/simu028/camx/imx011.jpg]

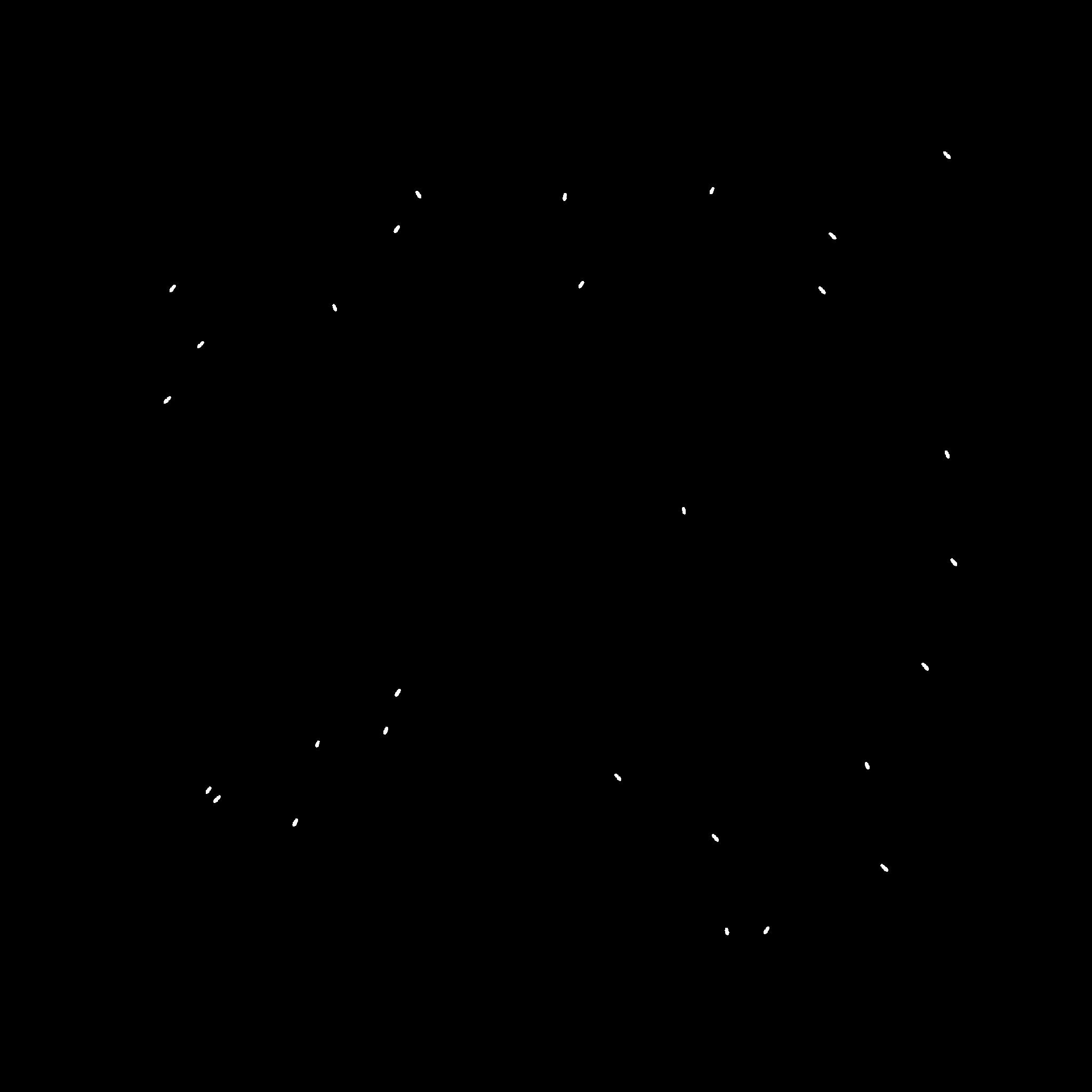

Supplement: S1 File — (ZIP) [file pone.0132101.s003.zip › ORsrc/nonortho/simu028/camx/imx012.jpg]

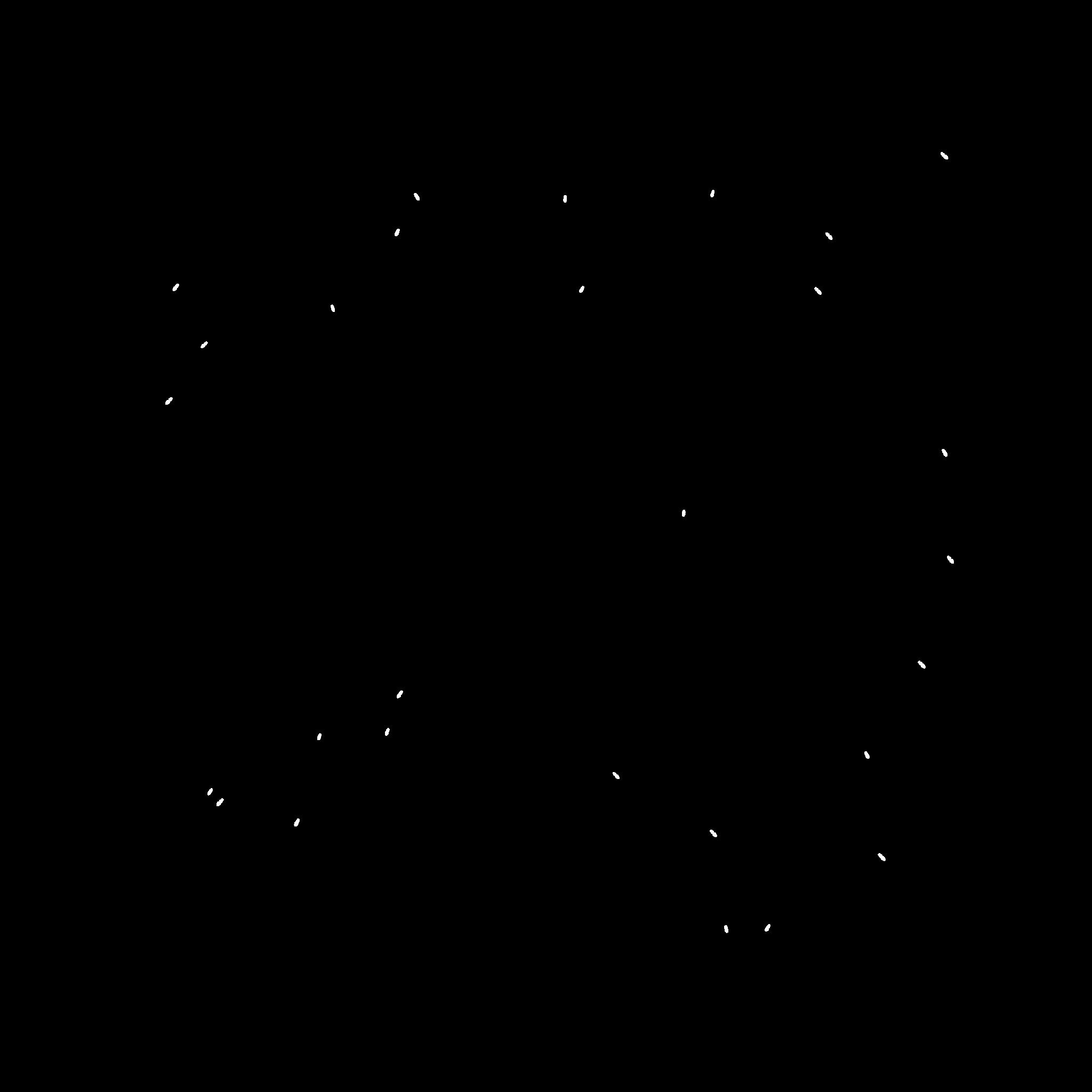

Supplement: S1 File — (ZIP) [file pone.0132101.s003.zip › ORsrc/nonortho/simu028/camx/imx013.jpg]

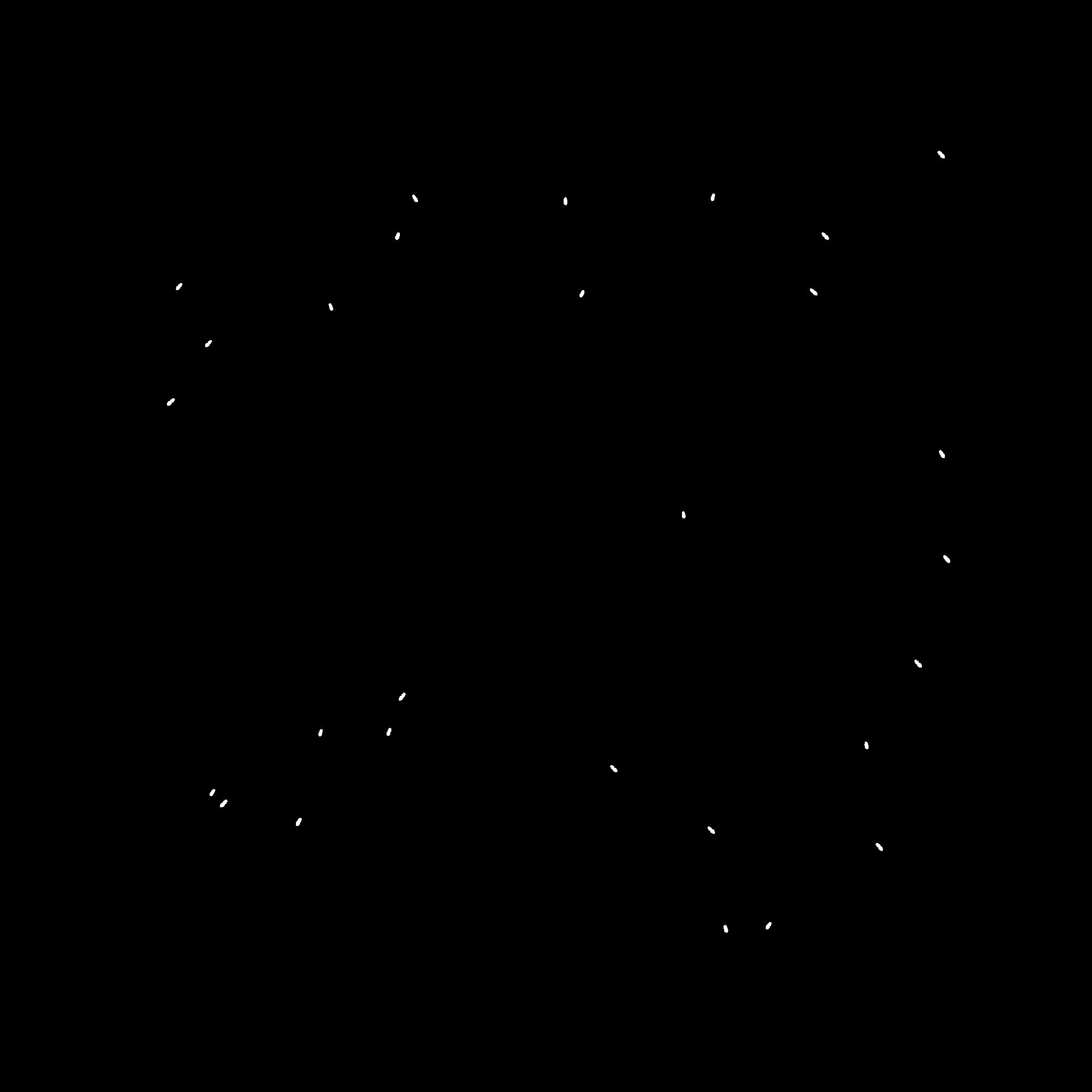

Supplement: S1 File — (ZIP) [file pone.0132101.s003.zip › ORsrc/nonortho/simu028/camx/imx014.jpg]

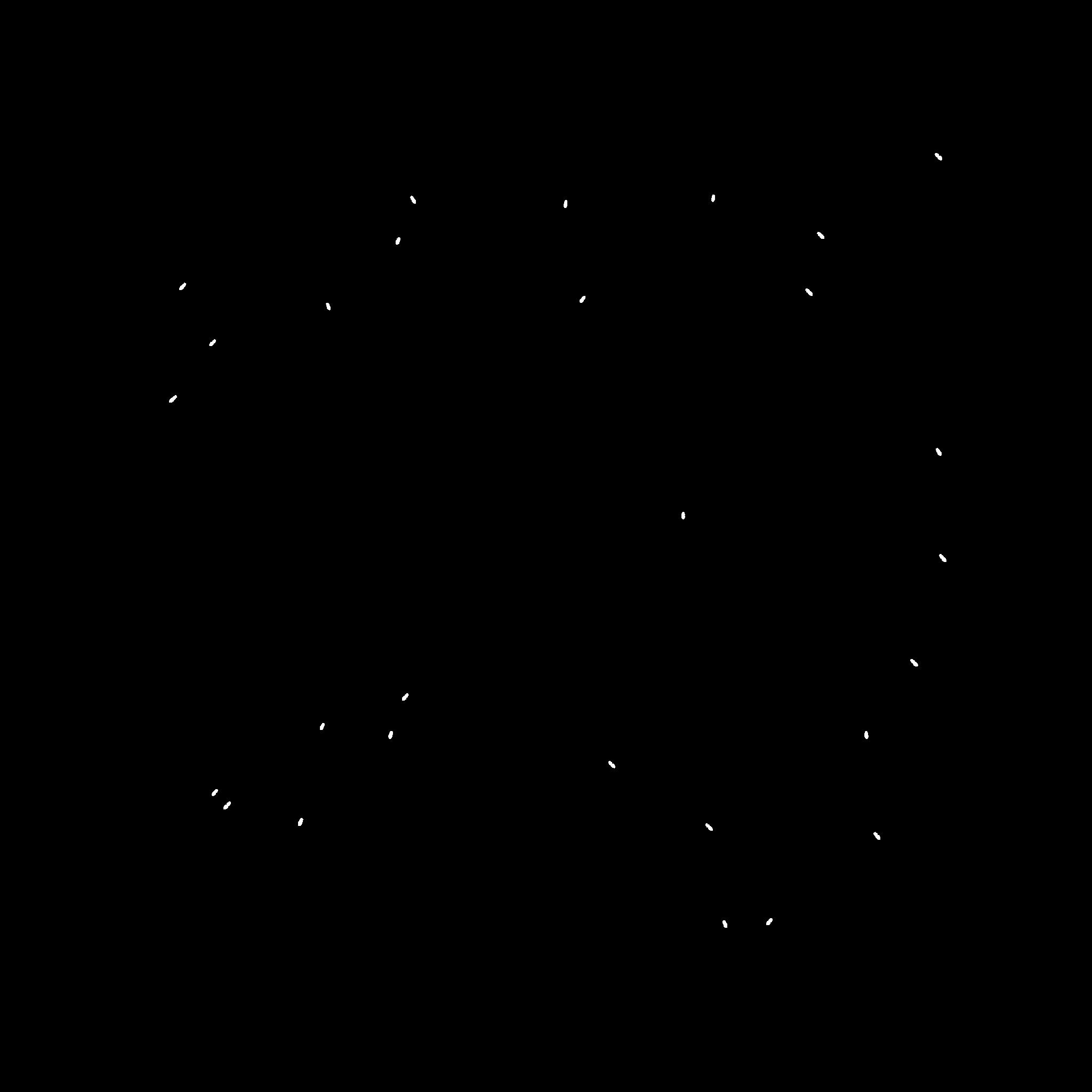

Supplement: S1 File — (ZIP) [file pone.0132101.s003.zip › ORsrc/nonortho/simu028/camx/imx015.jpg]

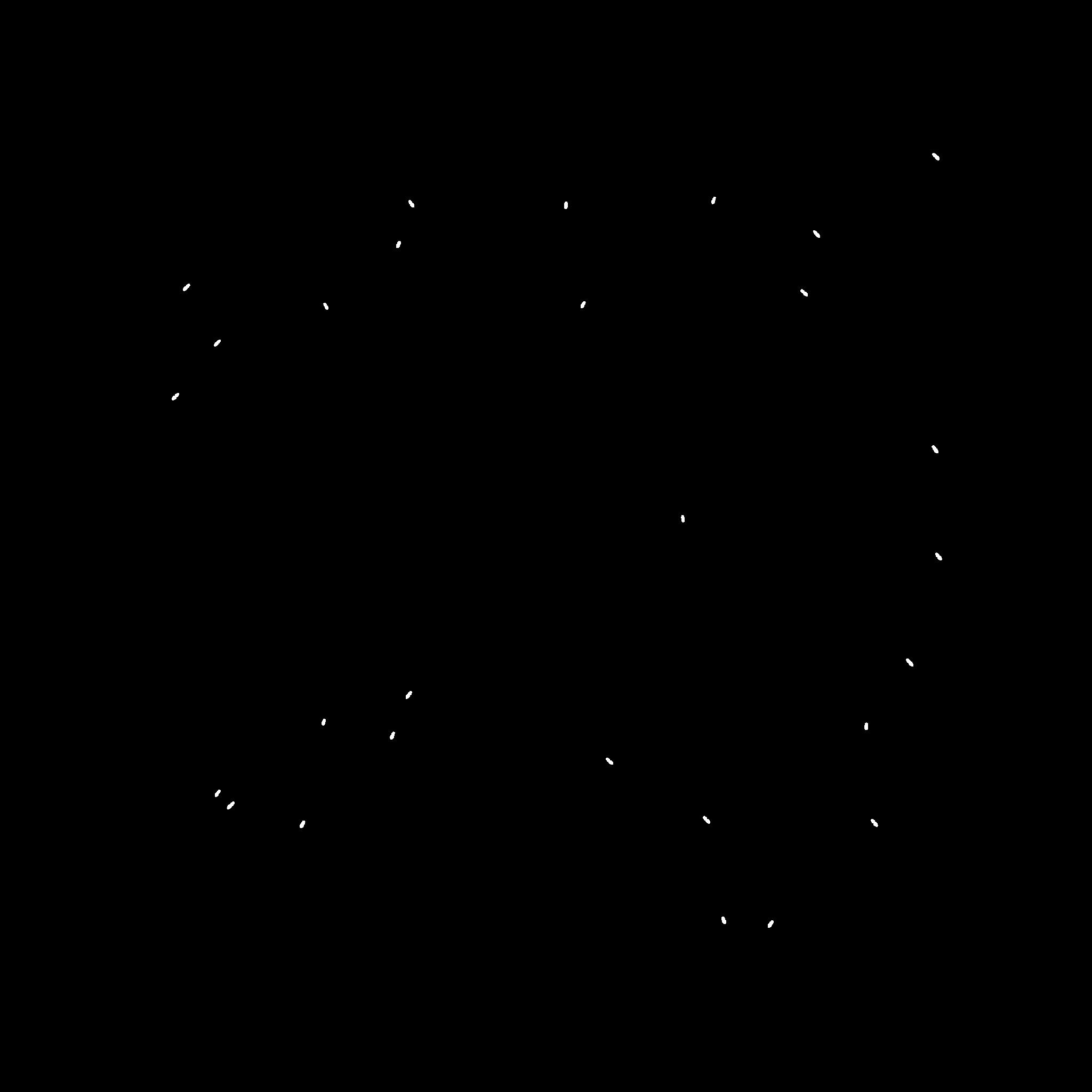

Supplement: S1 File — (ZIP) [file pone.0132101.s003.zip › ORsrc/nonortho/simu028/camx/imx016.jpg]

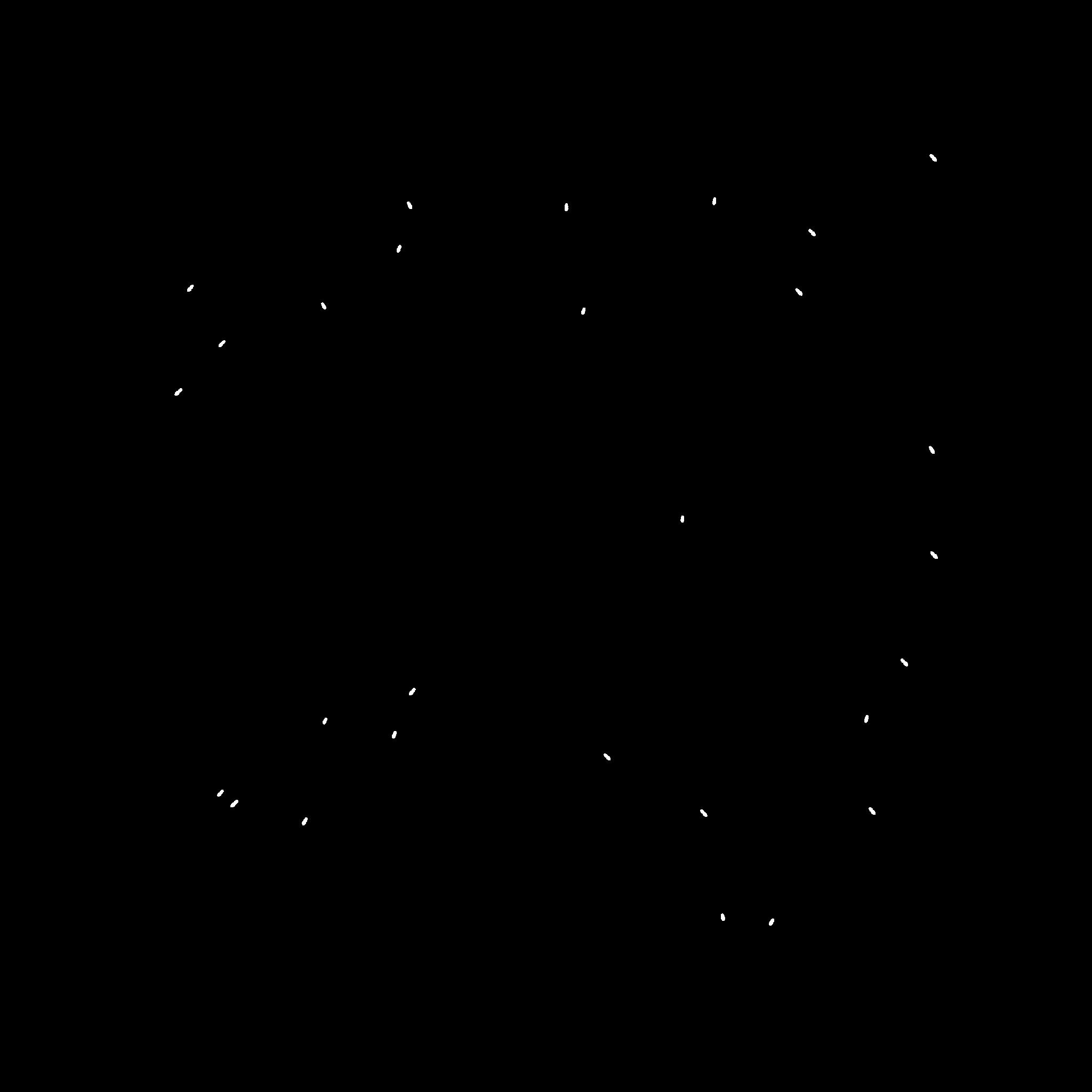

Supplement: S1 File — (ZIP) [file pone.0132101.s003.zip › ORsrc/nonortho/simu028/camx/imx017.jpg]

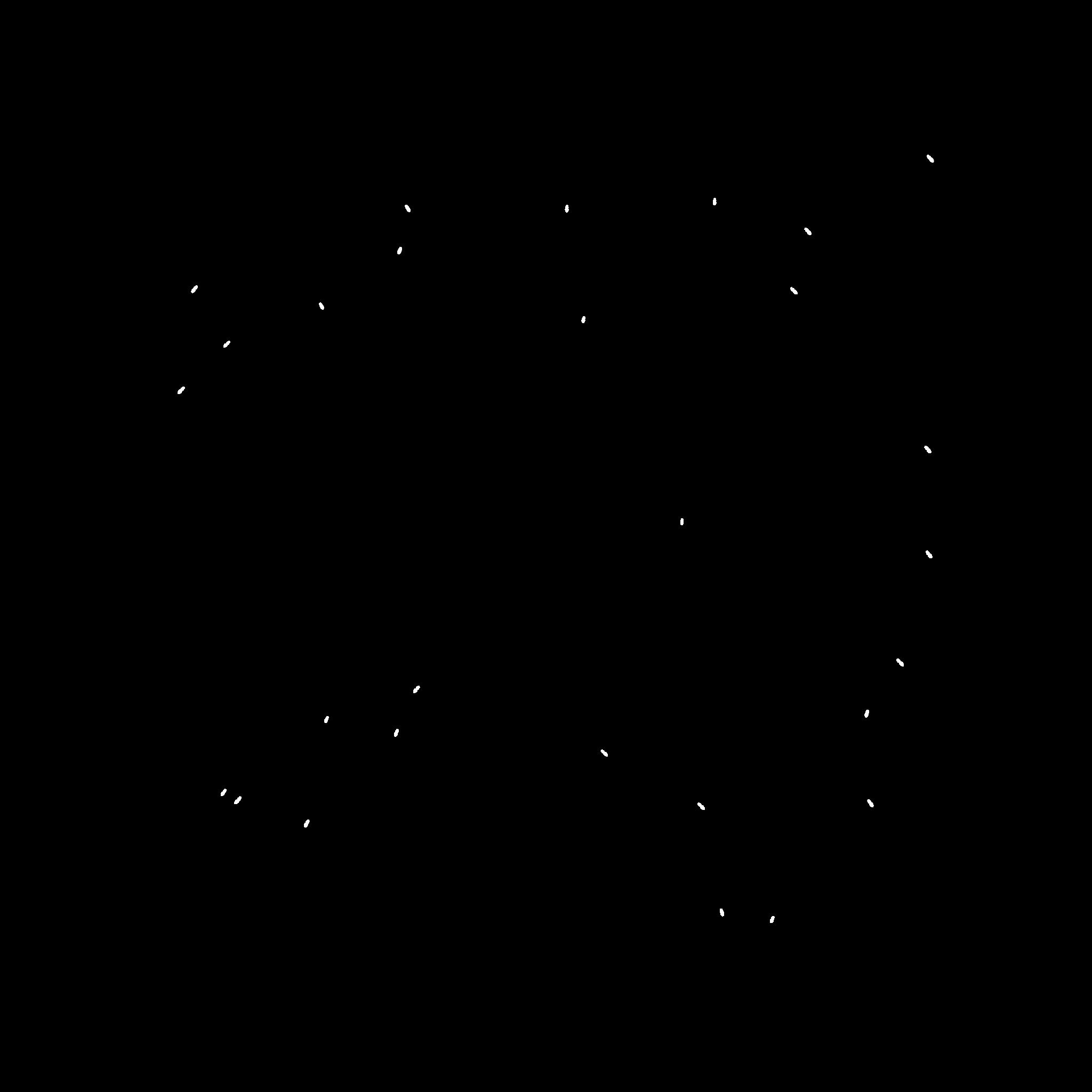

Supplement: S1 File — (ZIP) [file pone.0132101.s003.zip › ORsrc/nonortho/simu028/camx/imx018.jpg]

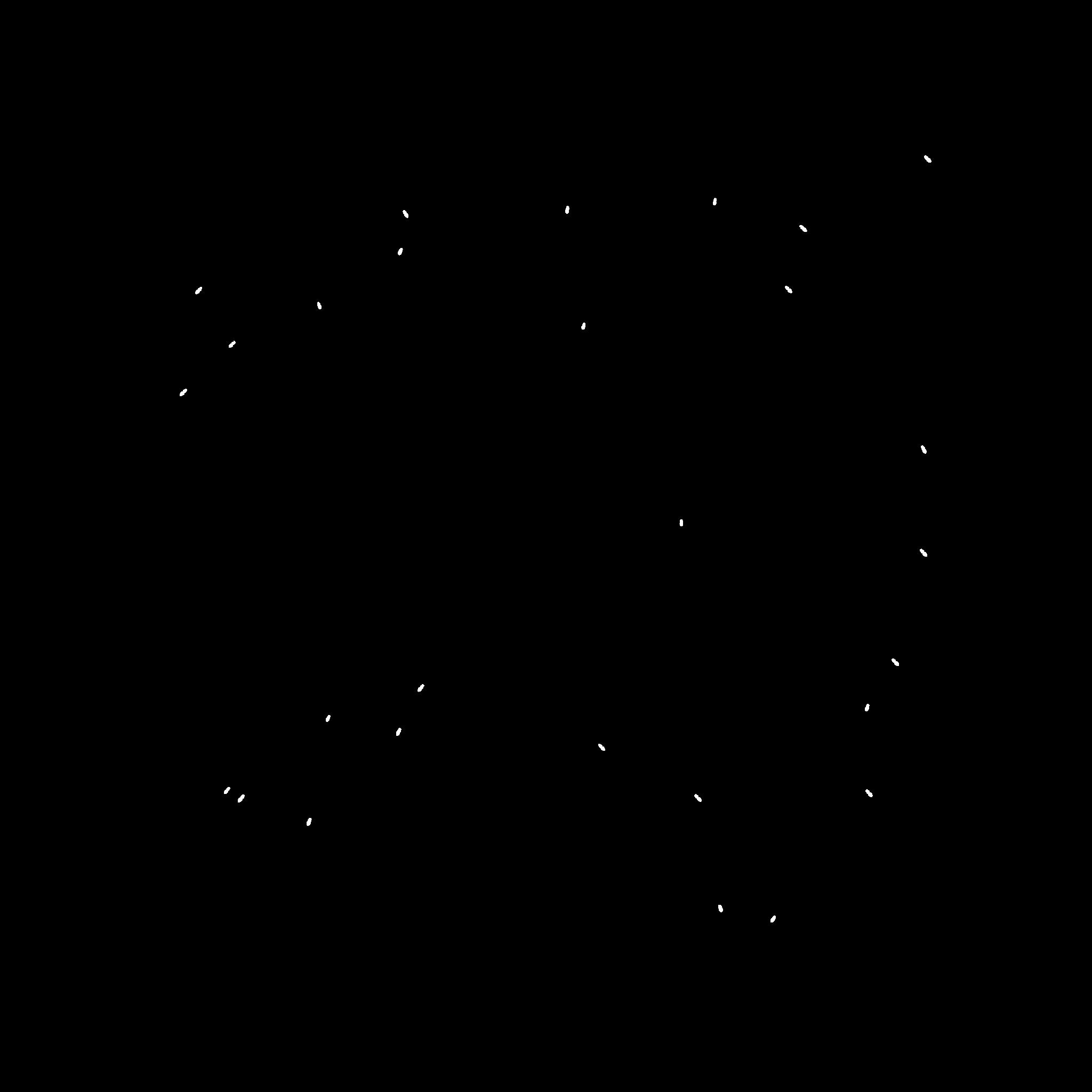

Supplement: S1 File — (ZIP) [file pone.0132101.s003.zip › ORsrc/nonortho/simu028/camx/imx019.jpg]

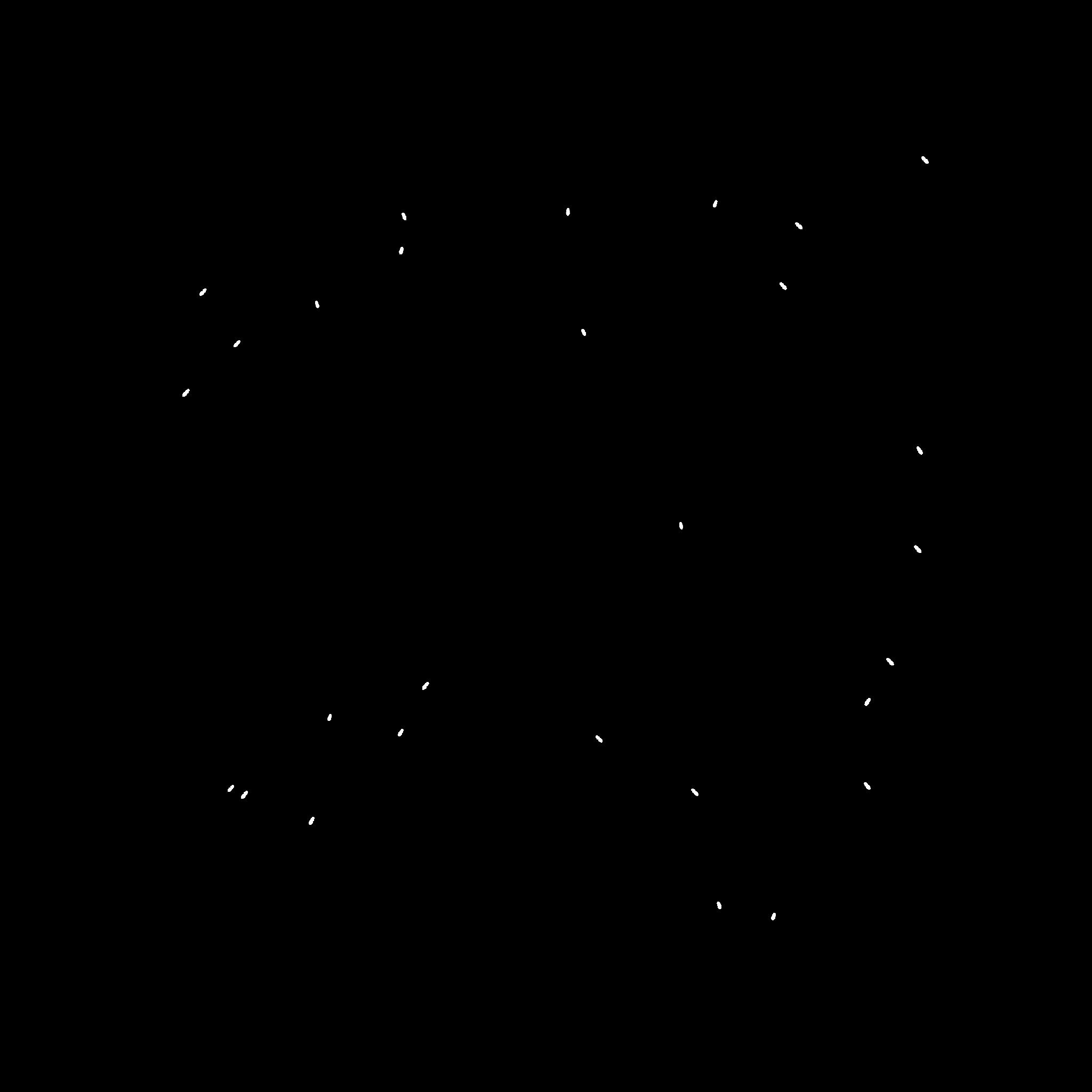

Supplement: S1 File — (ZIP) [file pone.0132101.s003.zip › ORsrc/nonortho/simu028/camx/imx020.jpg]

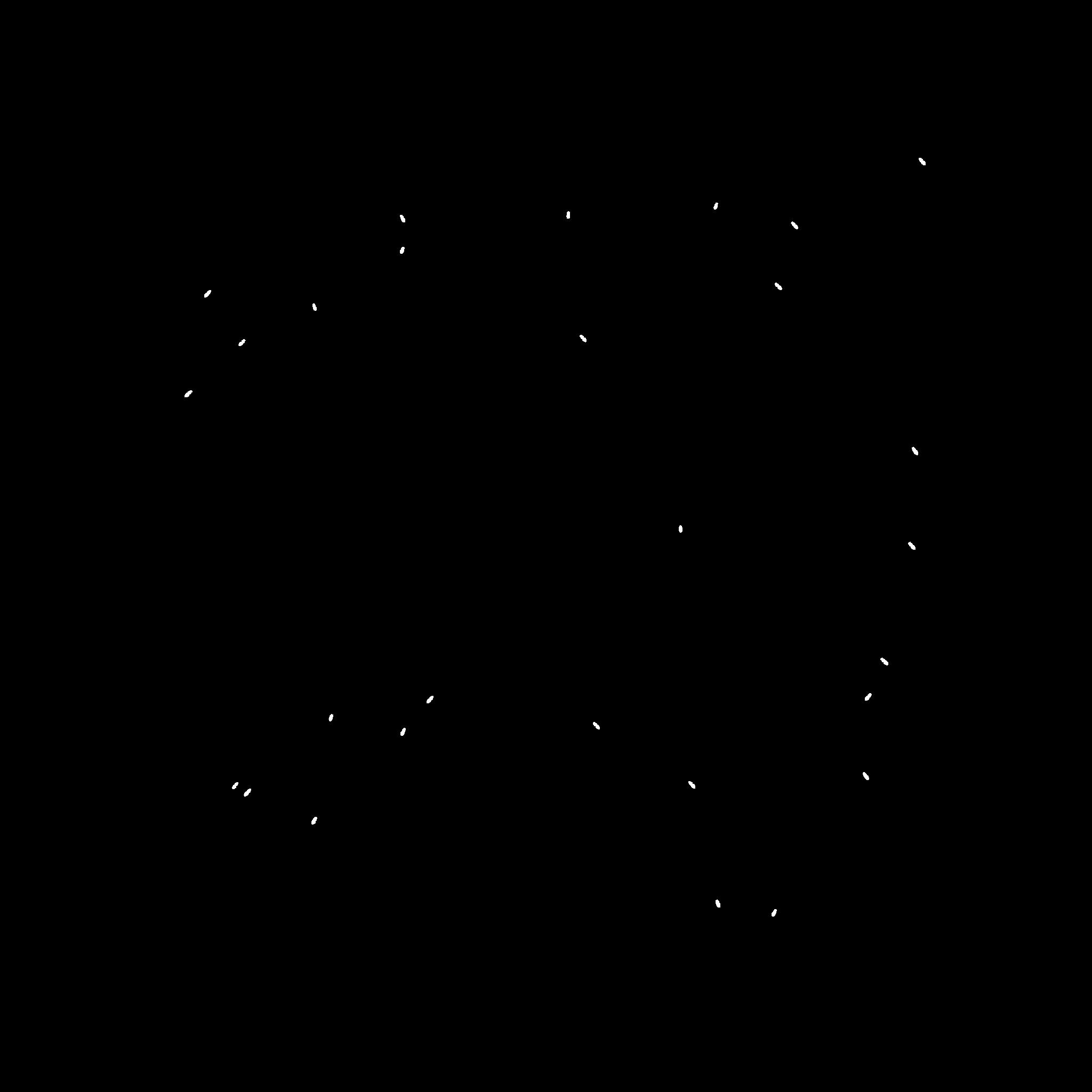

Supplement: S1 File — (ZIP) [file pone.0132101.s003.zip › ORsrc/nonortho/simu028/camx/imx021.jpg]

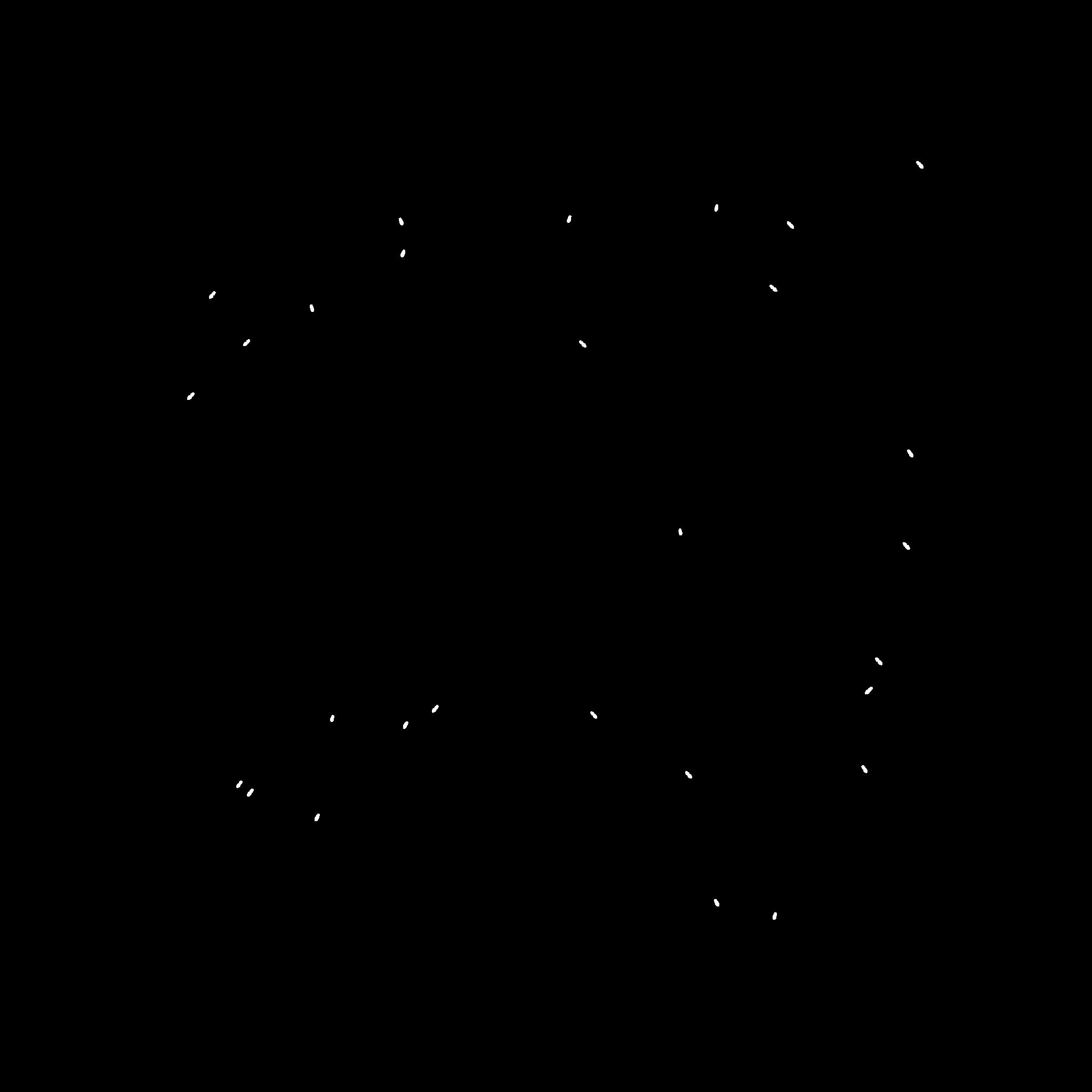

Supplement: S1 File — (ZIP) [file pone.0132101.s003.zip › ORsrc/nonortho/simu028/camx/imx022.jpg]

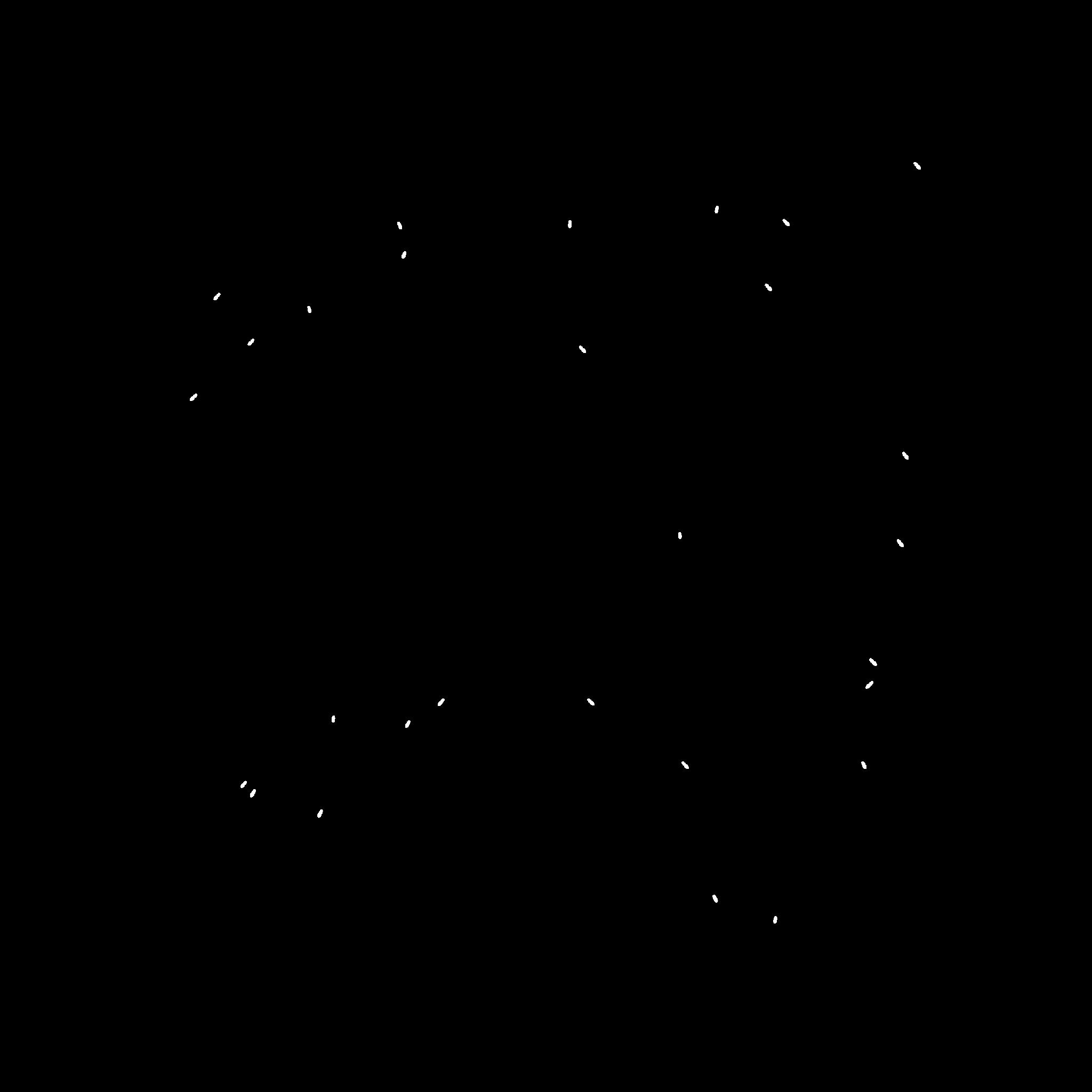

Supplement: S1 File — (ZIP) [file pone.0132101.s003.zip › ORsrc/nonortho/simu028/camx/imx023.jpg]

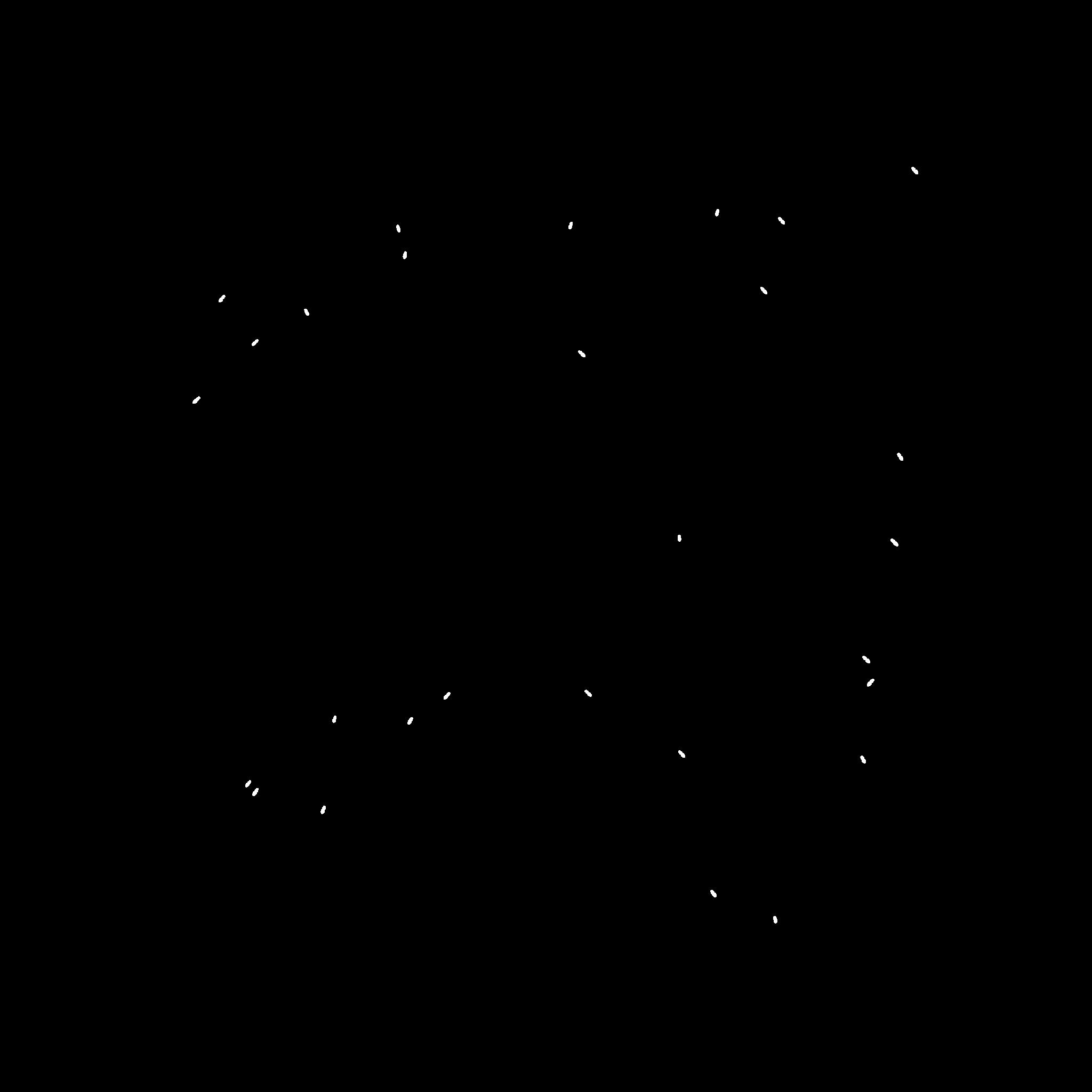

Supplement: S1 File — (ZIP) [file pone.0132101.s003.zip › ORsrc/nonortho/simu028/camx/imx024.jpg]

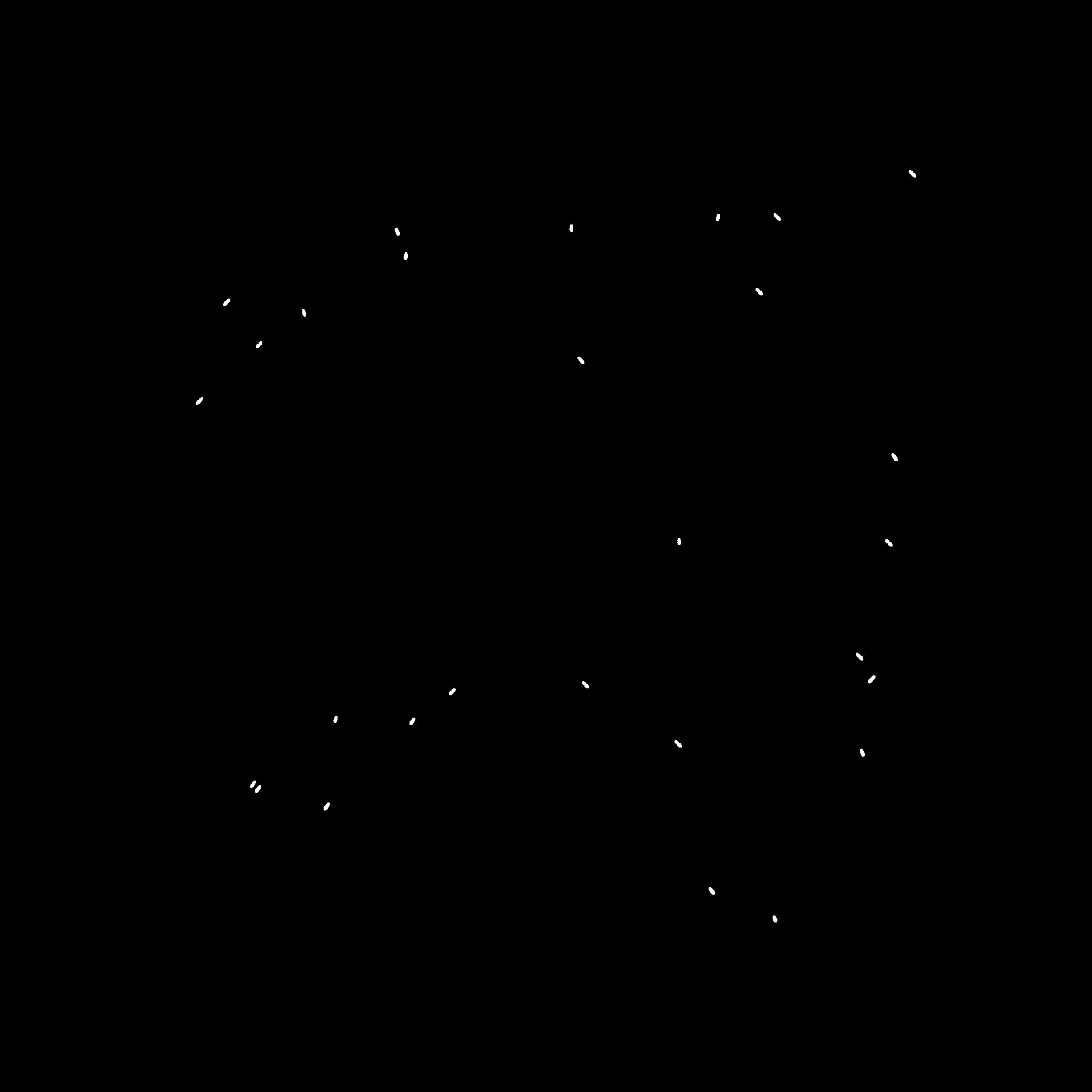

Supplement: S1 File — (ZIP) [file pone.0132101.s003.zip › ORsrc/nonortho/simu028/camx/imx025.jpg]

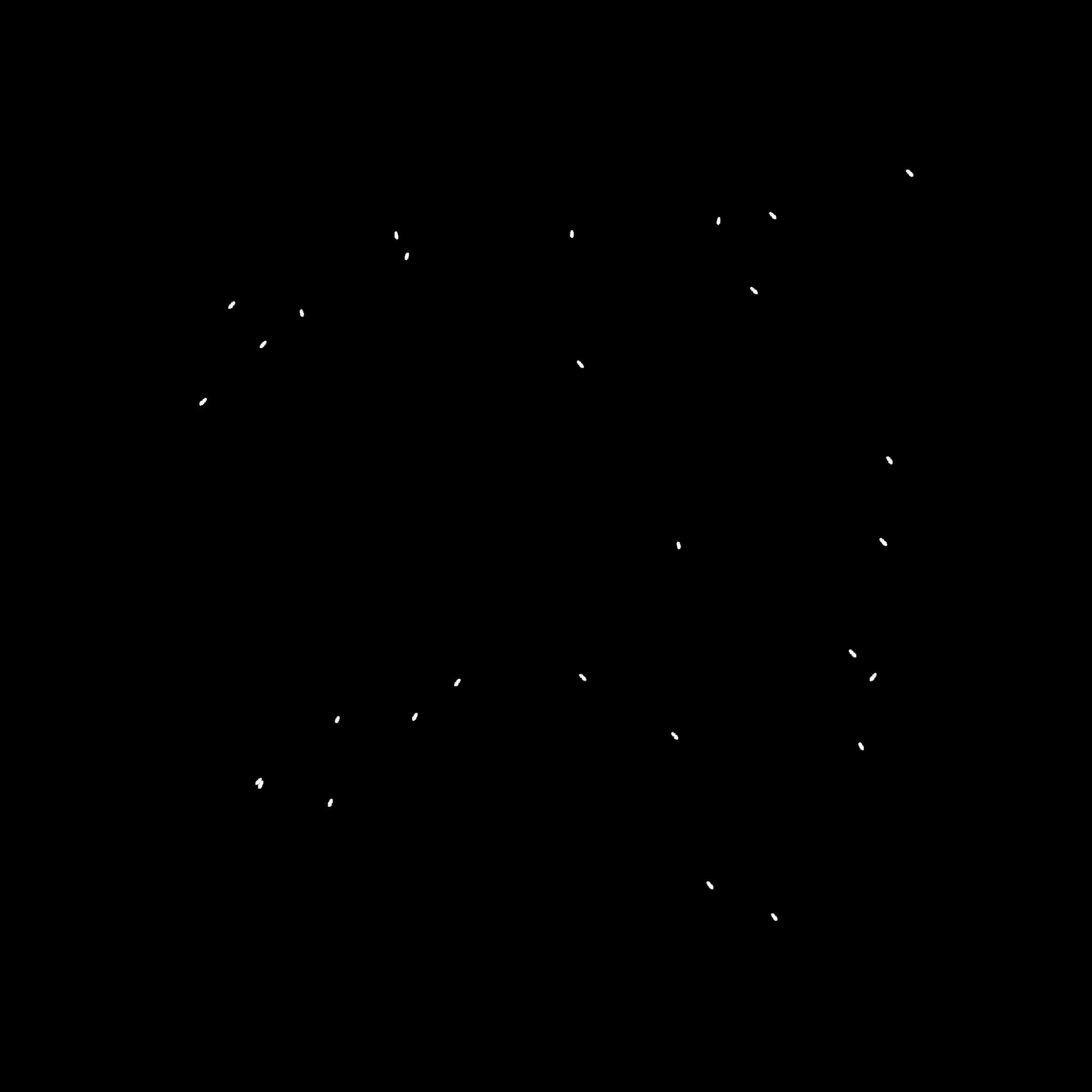

Supplement: S1 File — (ZIP) [file pone.0132101.s003.zip › ORsrc/nonortho/simu028/camx/imx026.jpg]

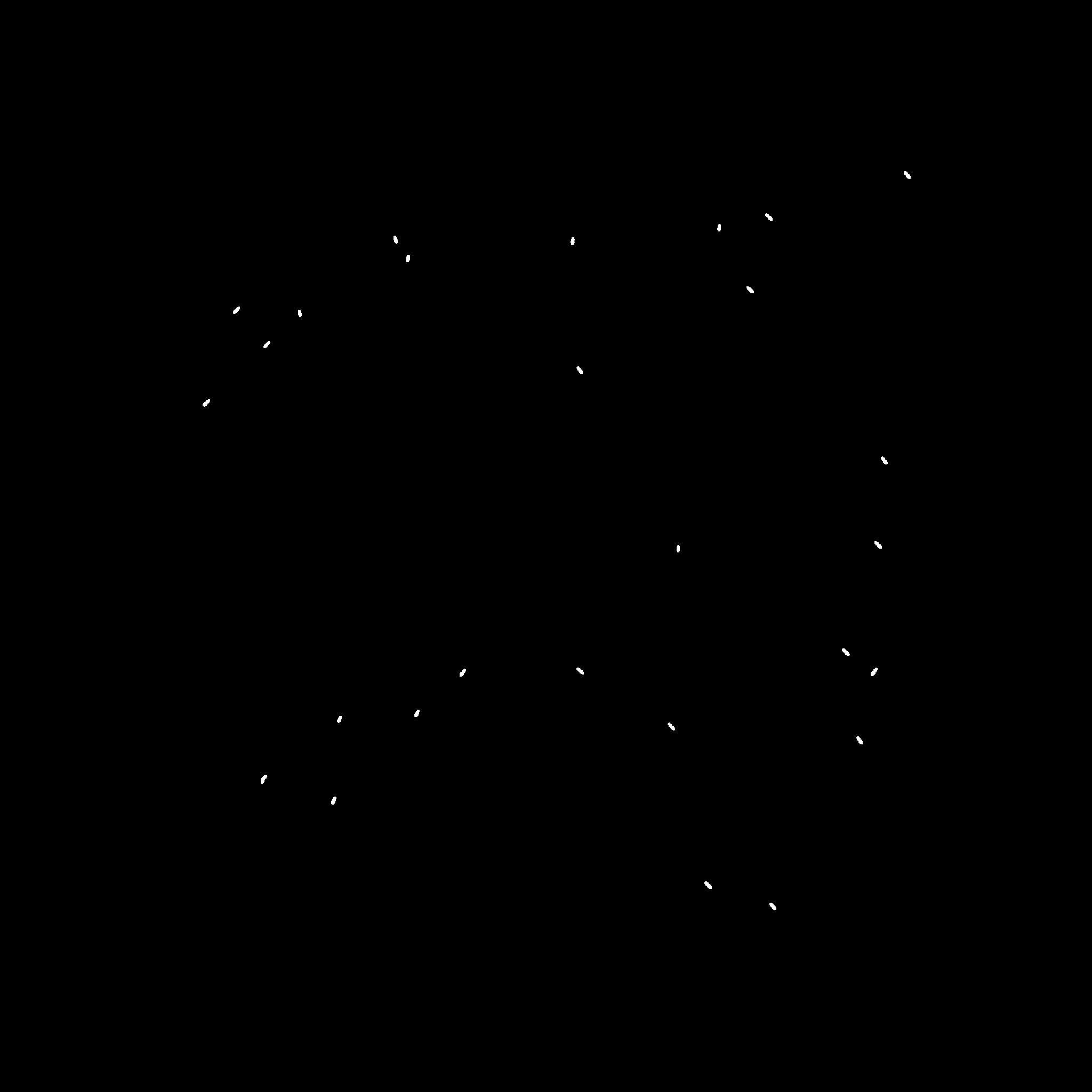

Supplement: S1 File — (ZIP) [file pone.0132101.s003.zip › ORsrc/nonortho/simu028/camx/imx027.jpg]

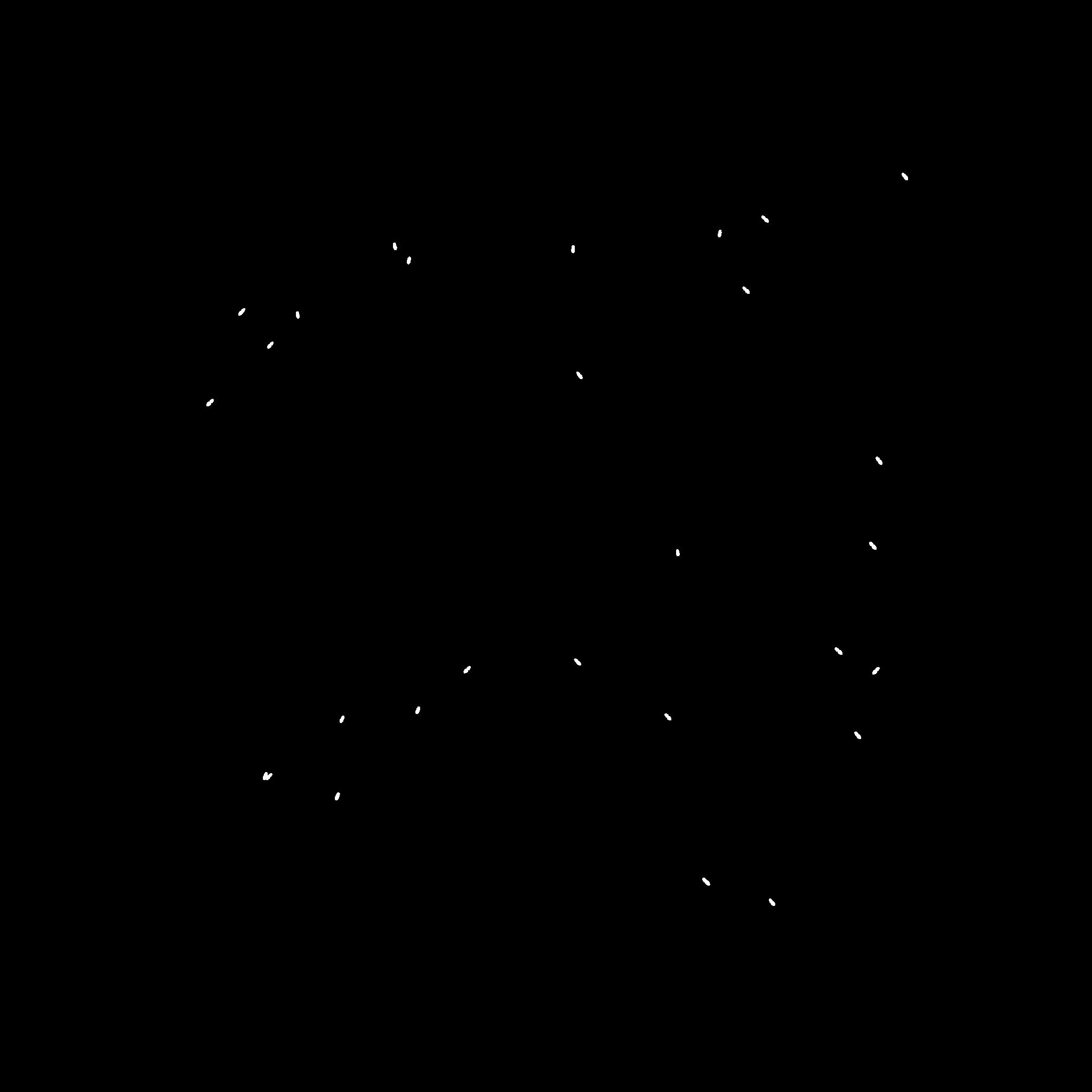

Supplement: S1 File — (ZIP) [file pone.0132101.s003.zip › ORsrc/nonortho/simu028/camx/imx028.jpg]

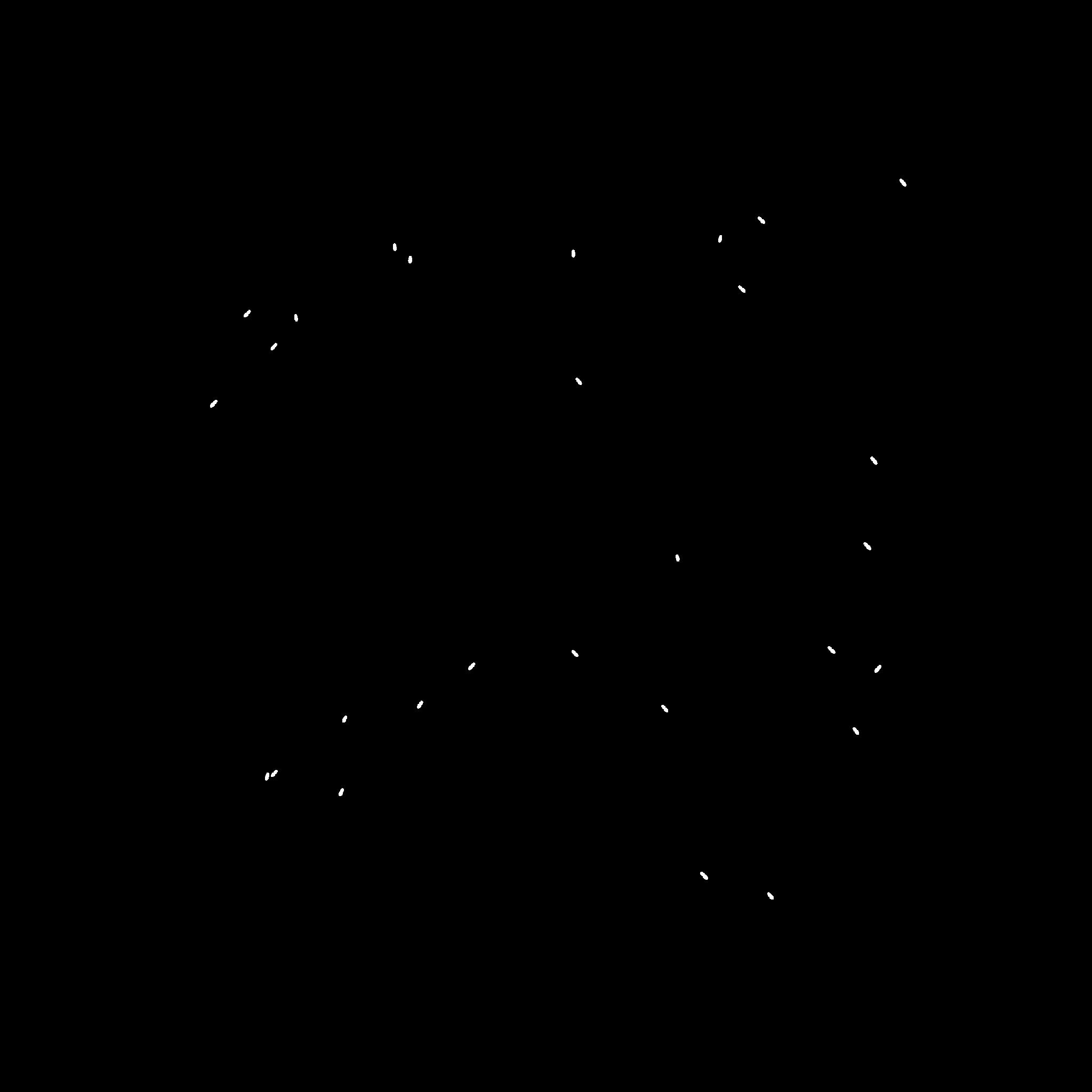

Supplement: S1 File — (ZIP) [file pone.0132101.s003.zip › ORsrc/nonortho/simu028/camx/imx029.jpg]

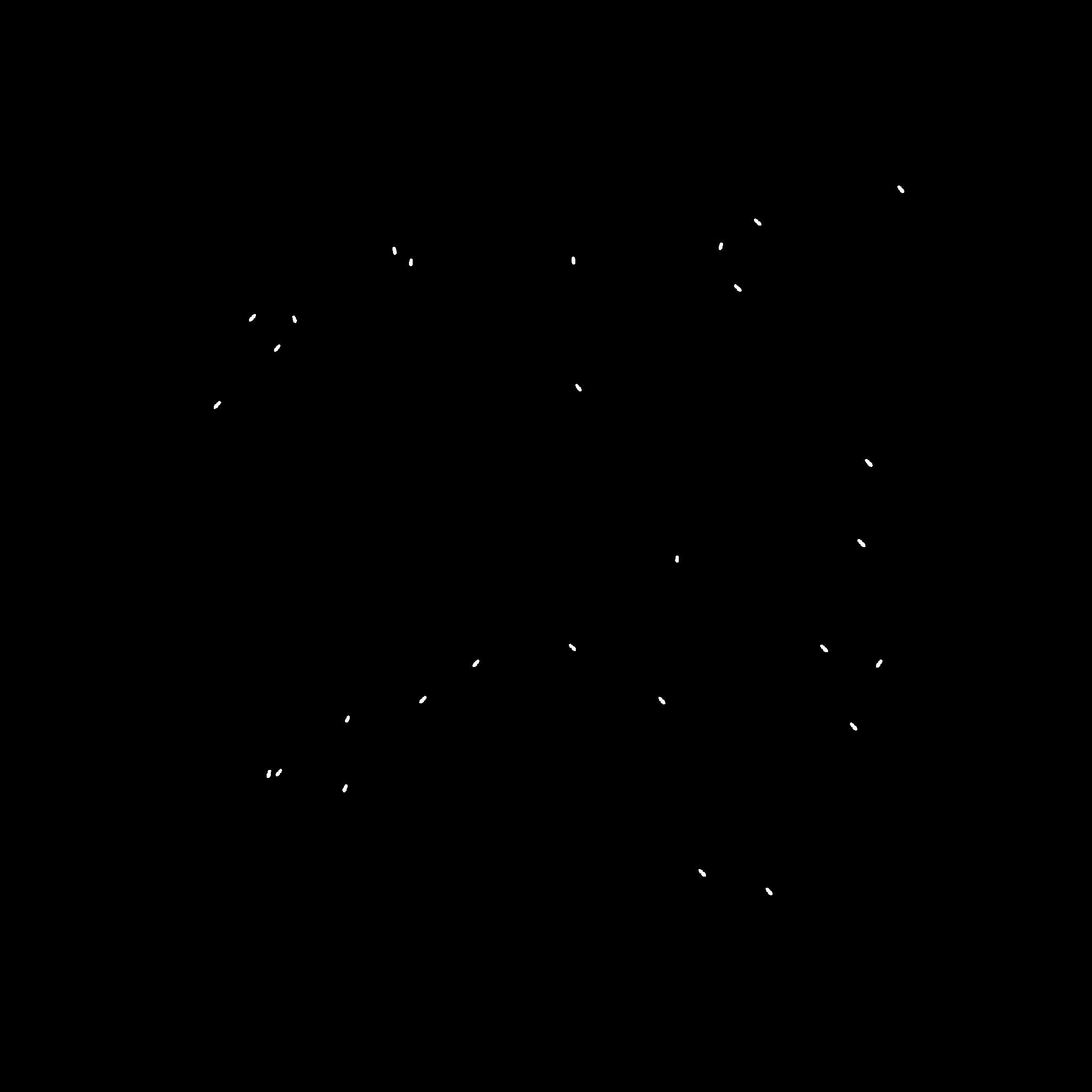

Supplement: S1 File — (ZIP) [file pone.0132101.s003.zip › ORsrc/nonortho/simu028/camx/imx030.jpg]

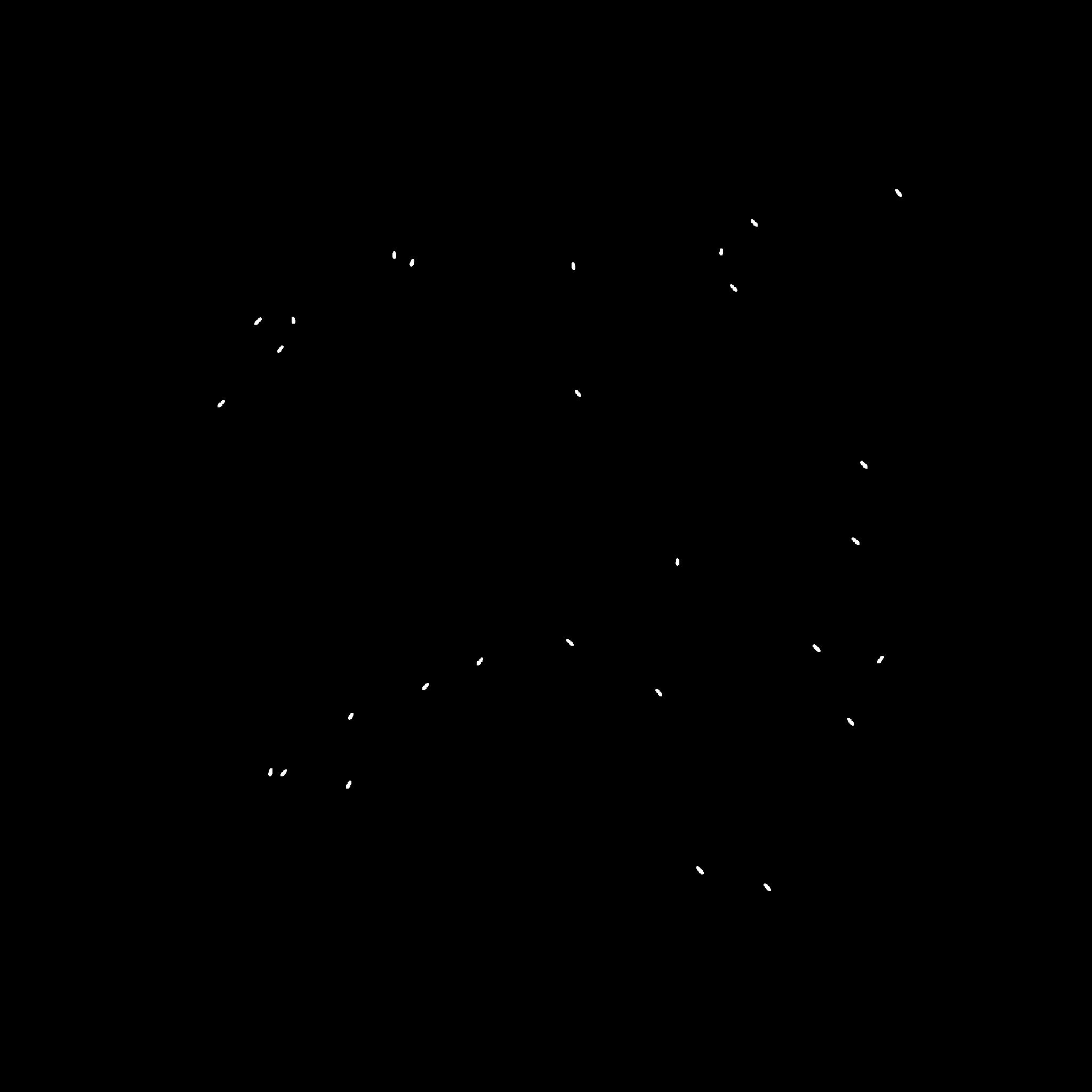

Supplement: S1 File — (ZIP) [file pone.0132101.s003.zip › ORsrc/nonortho/simu028/camx/imx031.jpg]

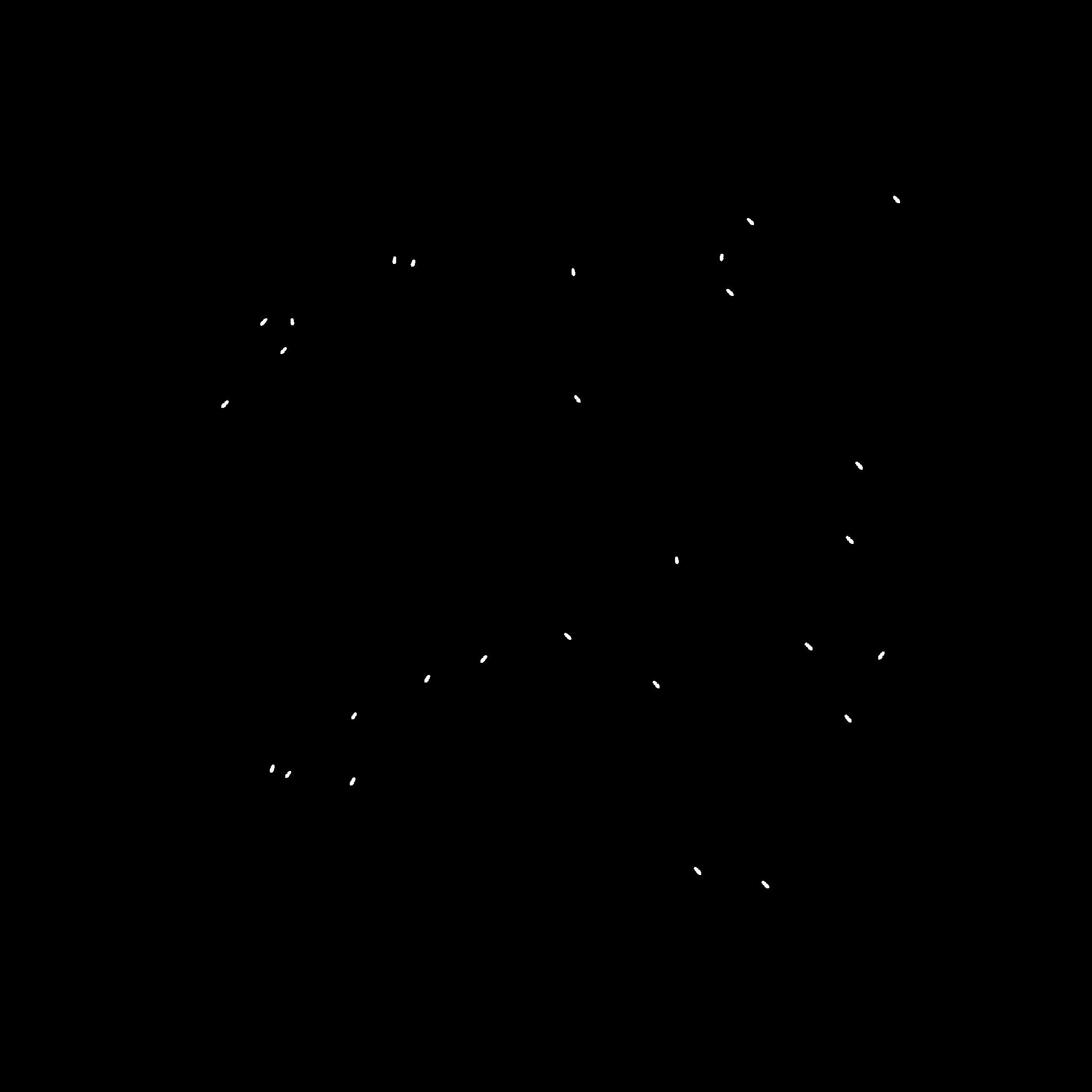

Supplement: S1 File — (ZIP) [file pone.0132101.s003.zip › ORsrc/nonortho/simu028/camx/imx032.jpg]

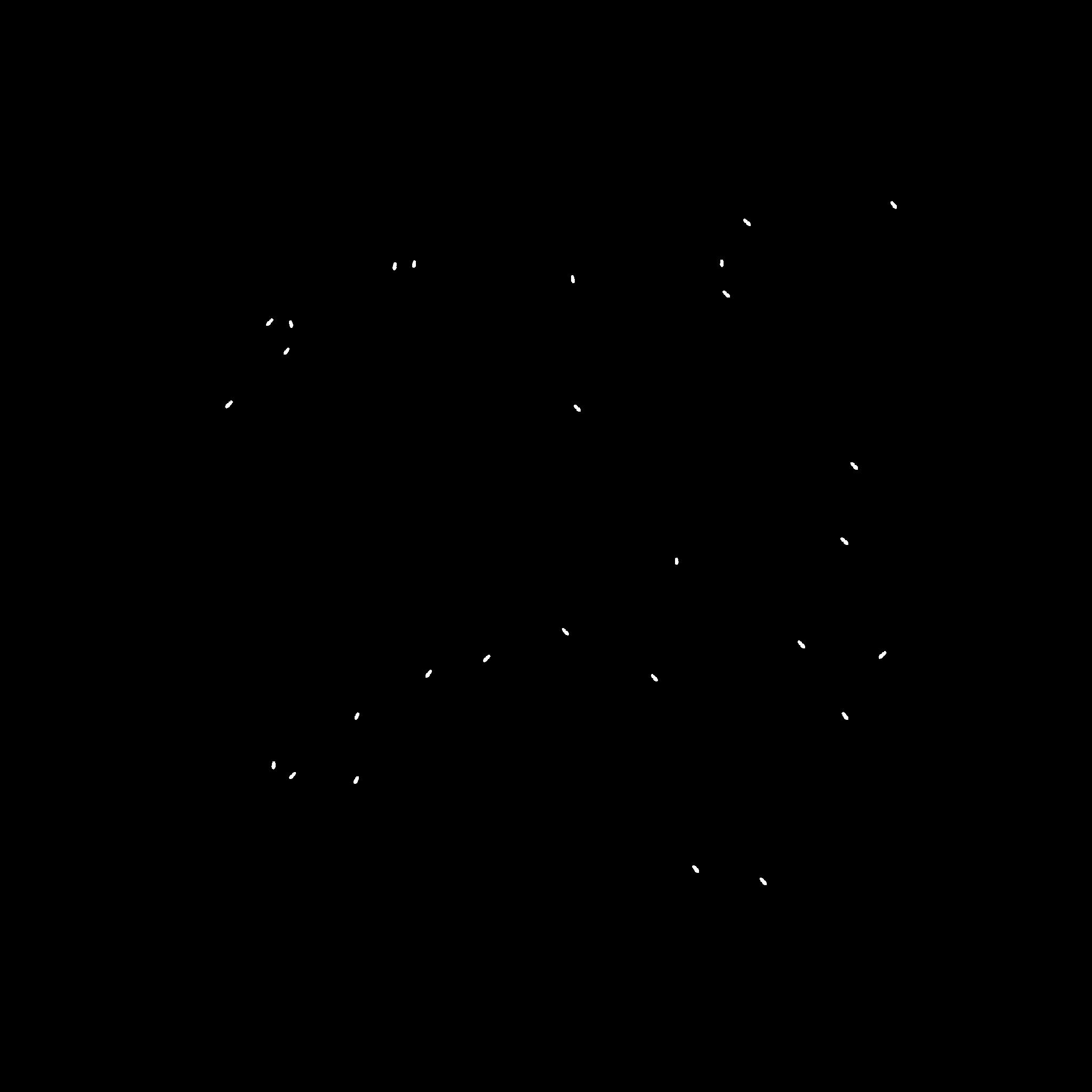

Supplement: S1 File — (ZIP) [file pone.0132101.s003.zip › ORsrc/nonortho/simu028/camx/imx033.jpg]

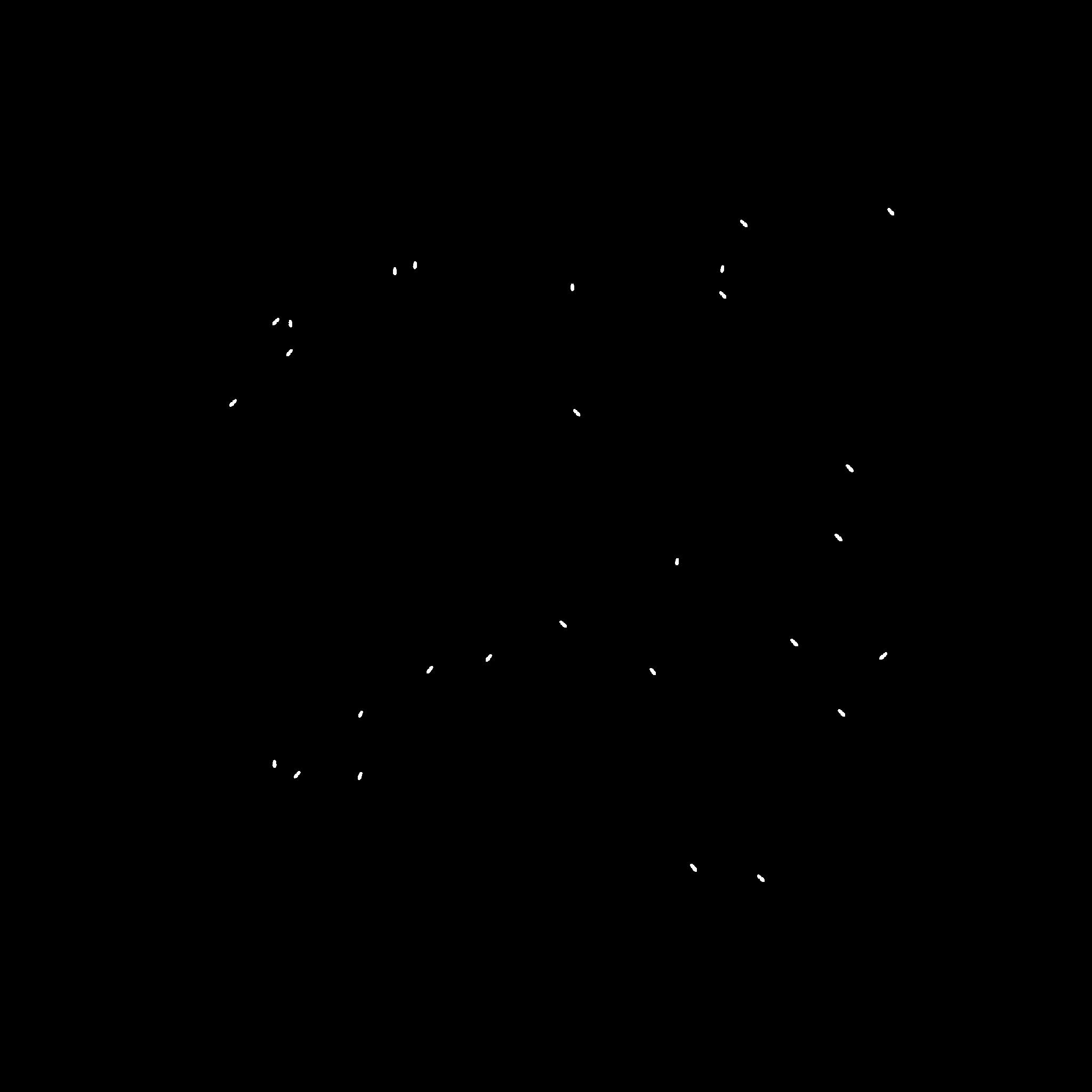

Supplement: S1 File — (ZIP) [file pone.0132101.s003.zip › ORsrc/nonortho/simu028/camx/imx034.jpg]

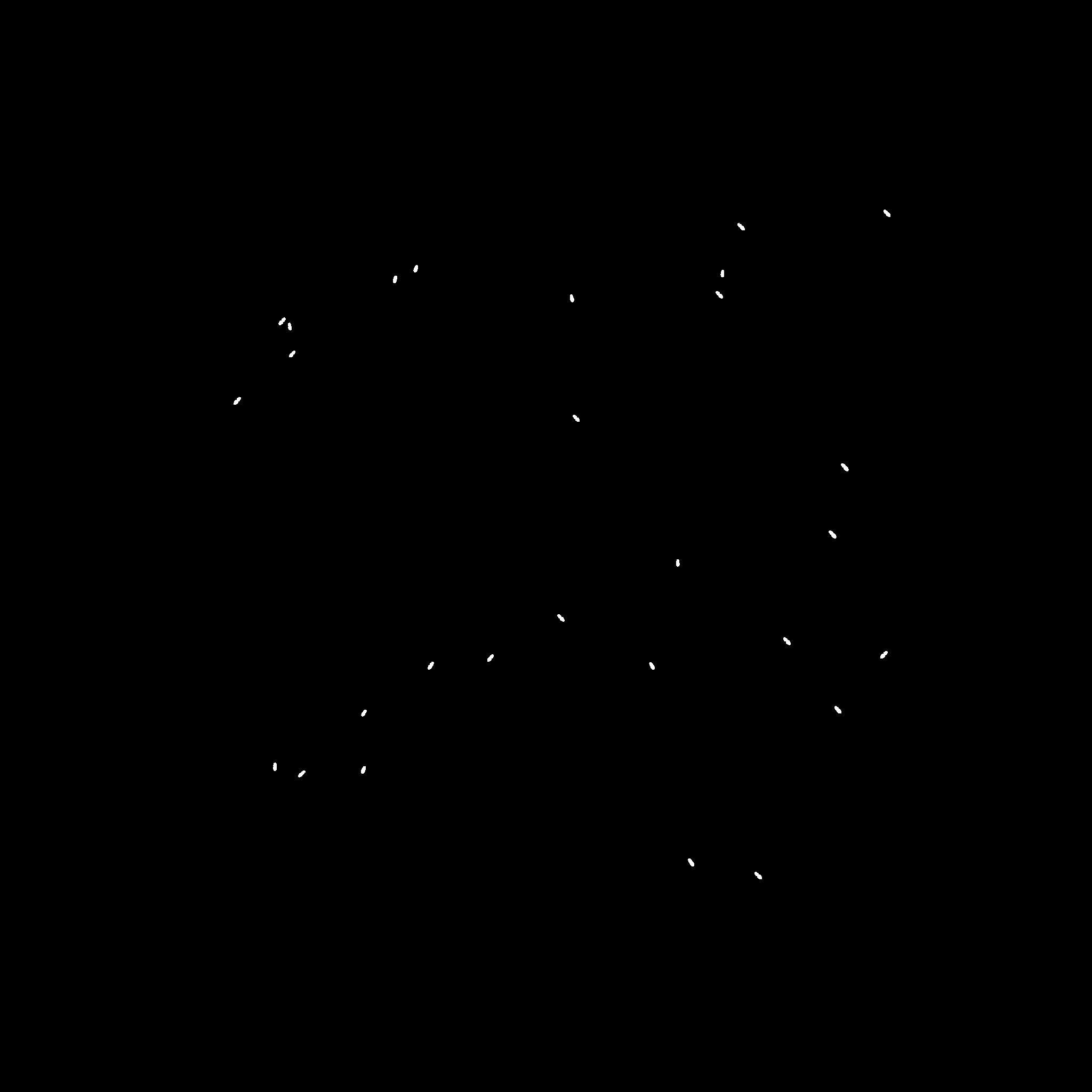

Supplement: S1 File — (ZIP) [file pone.0132101.s003.zip › ORsrc/nonortho/simu028/camx/imx035.jpg]

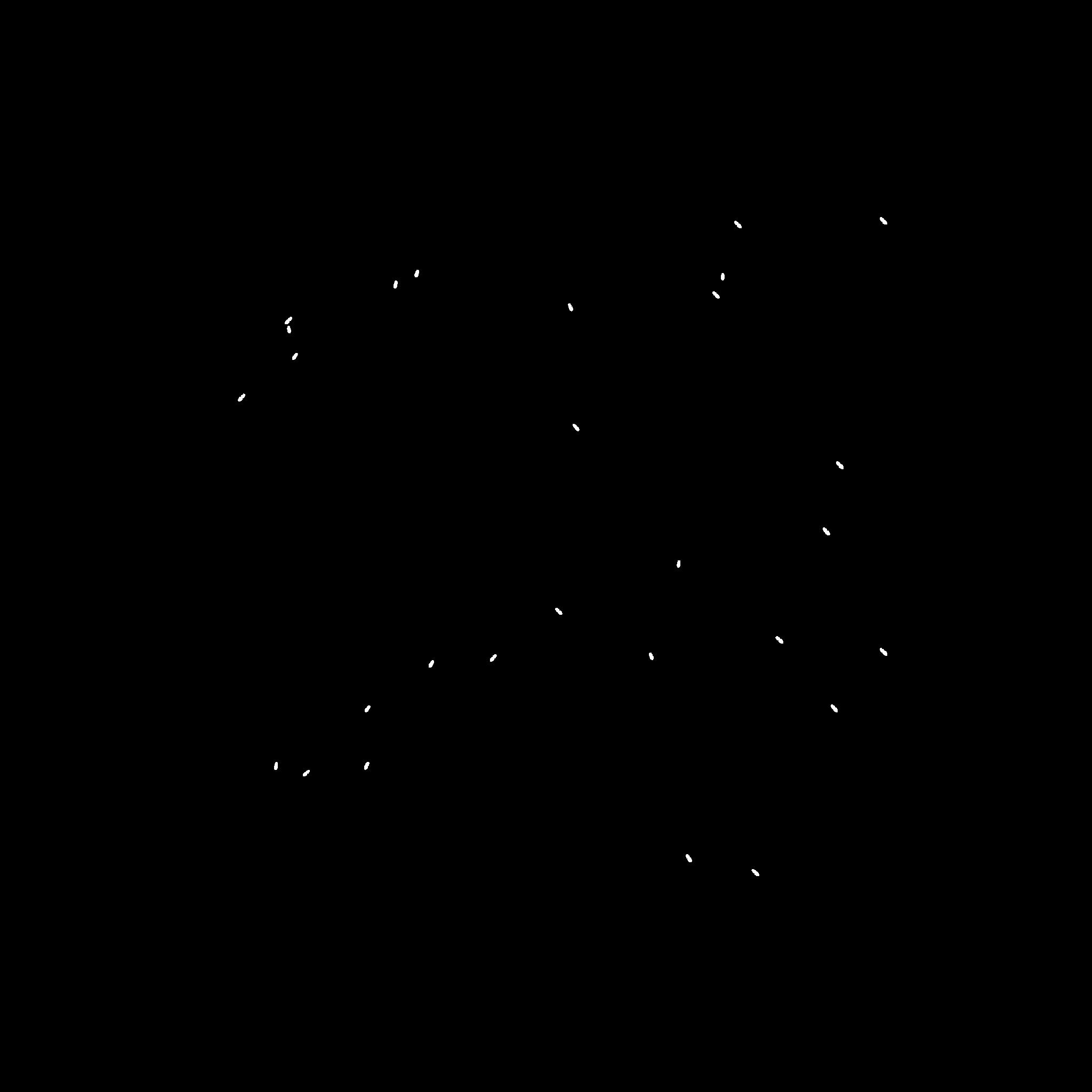

Supplement: S1 File — (ZIP) [file pone.0132101.s003.zip › ORsrc/nonortho/simu028/camx/imx036.jpg]

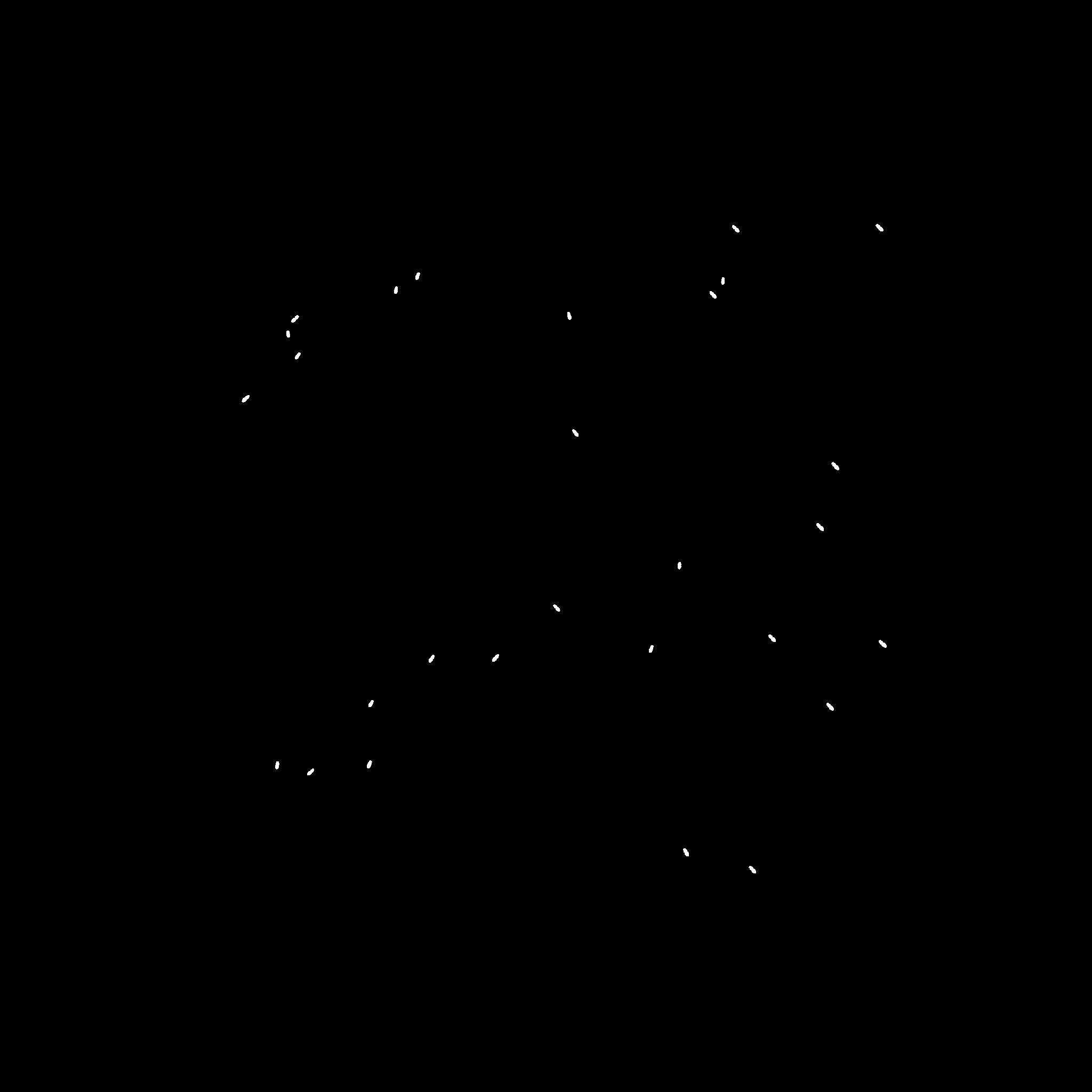

Supplement: S1 File — (ZIP) [file pone.0132101.s003.zip › ORsrc/nonortho/simu028/camx/imx037.jpg]

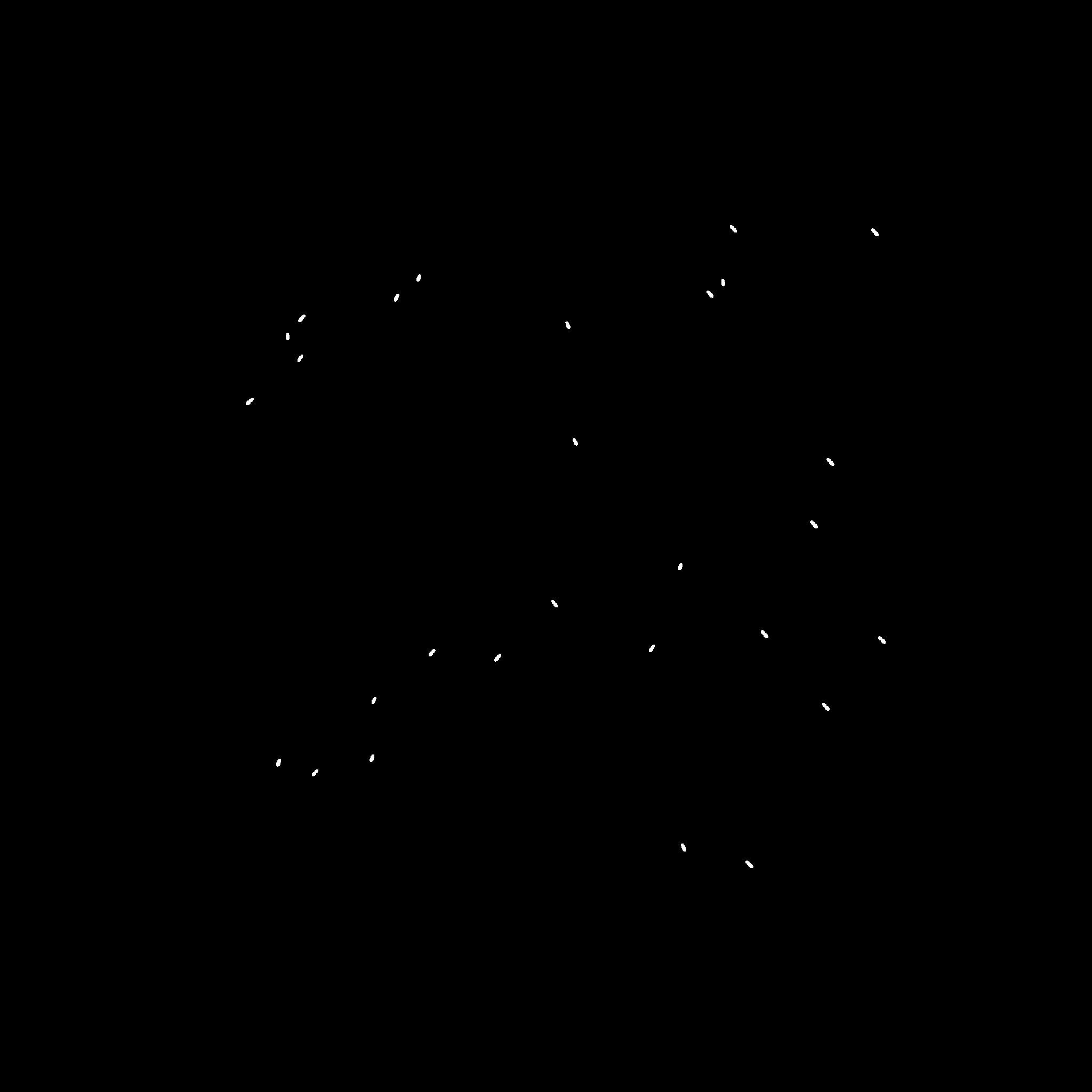

Supplement: S1 File — (ZIP) [file pone.0132101.s003.zip › ORsrc/nonortho/simu028/camx/imx038.jpg]

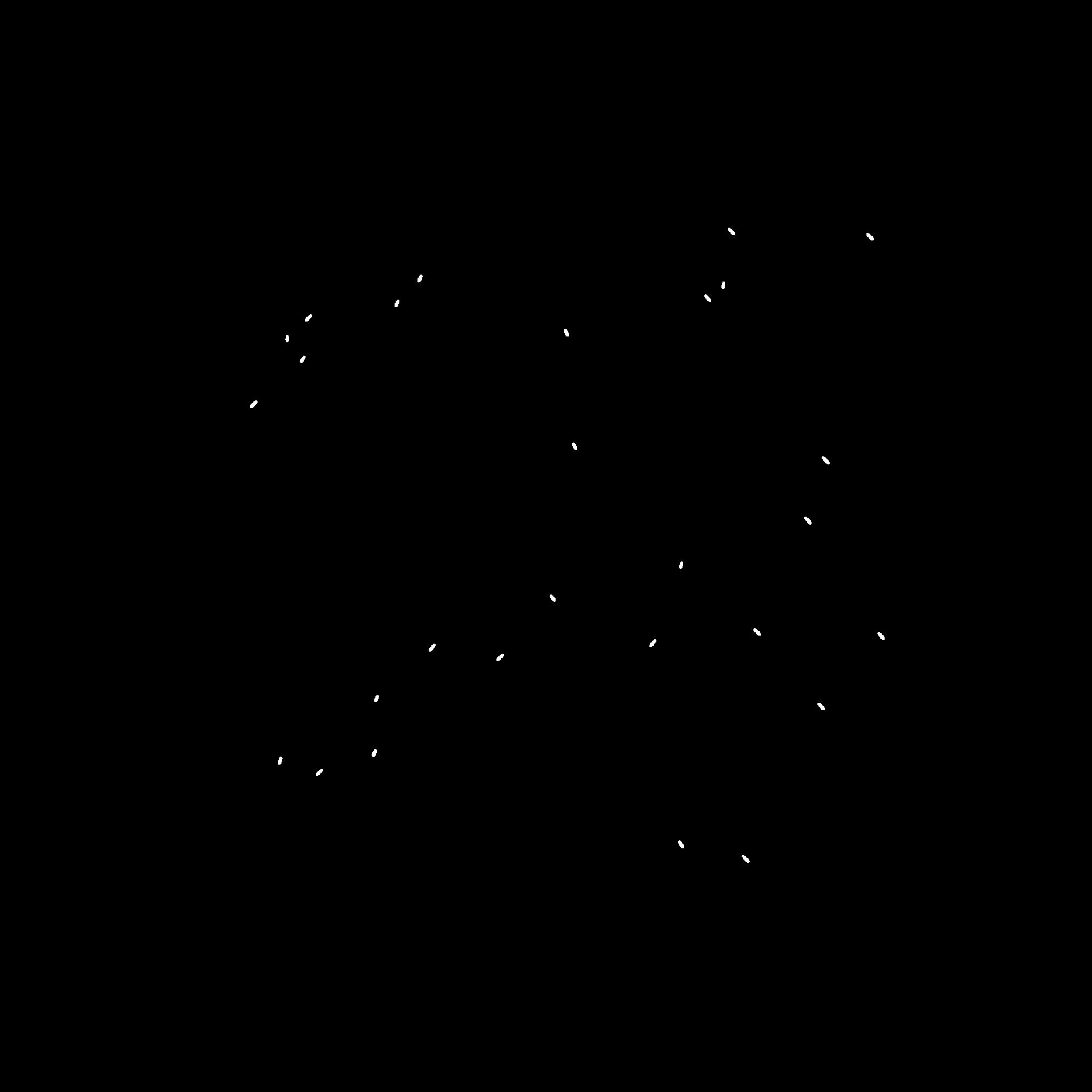

Supplement: S1 File — (ZIP) [file pone.0132101.s003.zip › ORsrc/nonortho/simu028/camx/imx039.jpg]

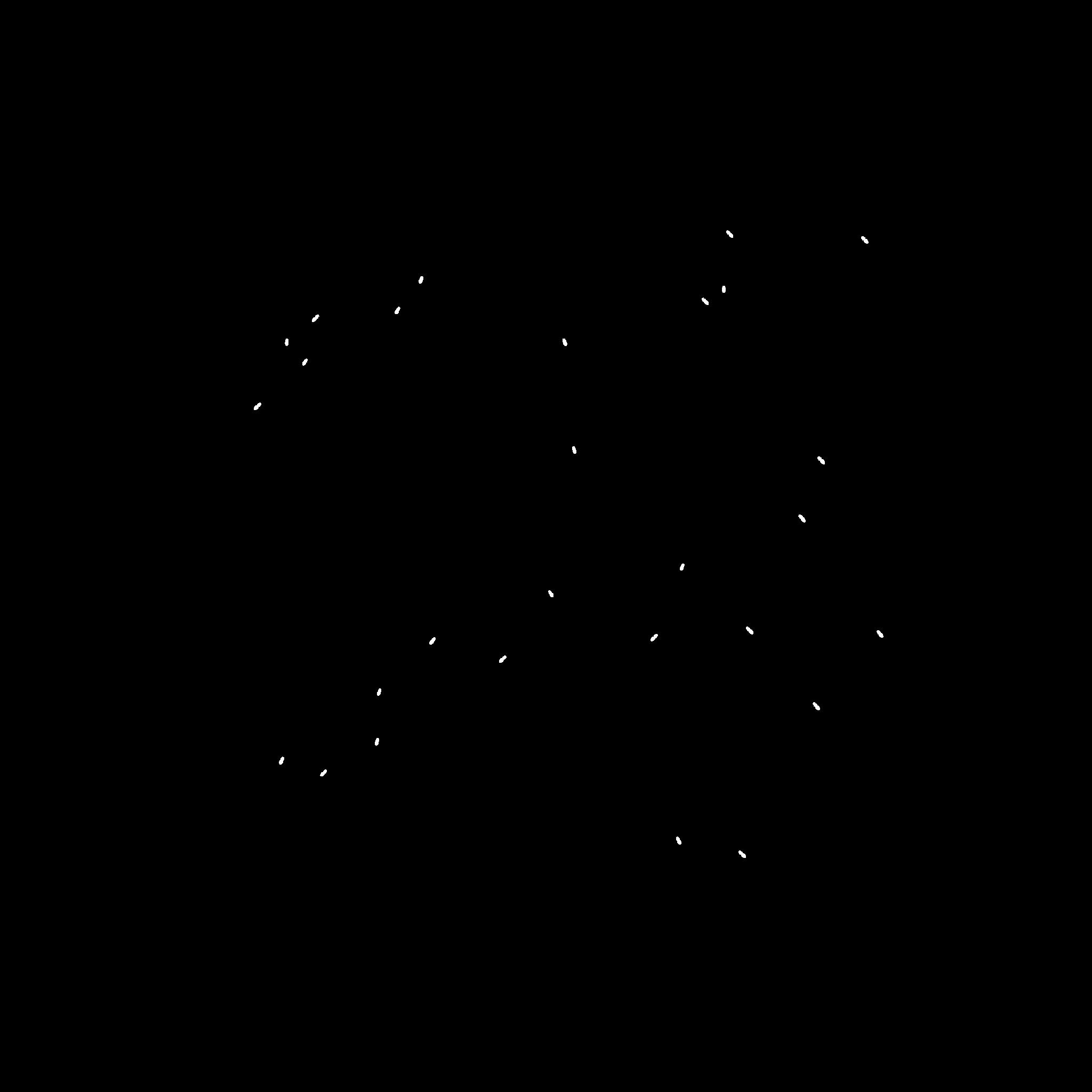

Supplement: S1 File — (ZIP) [file pone.0132101.s003.zip › ORsrc/nonortho/simu028/camx/imx040.jpg]

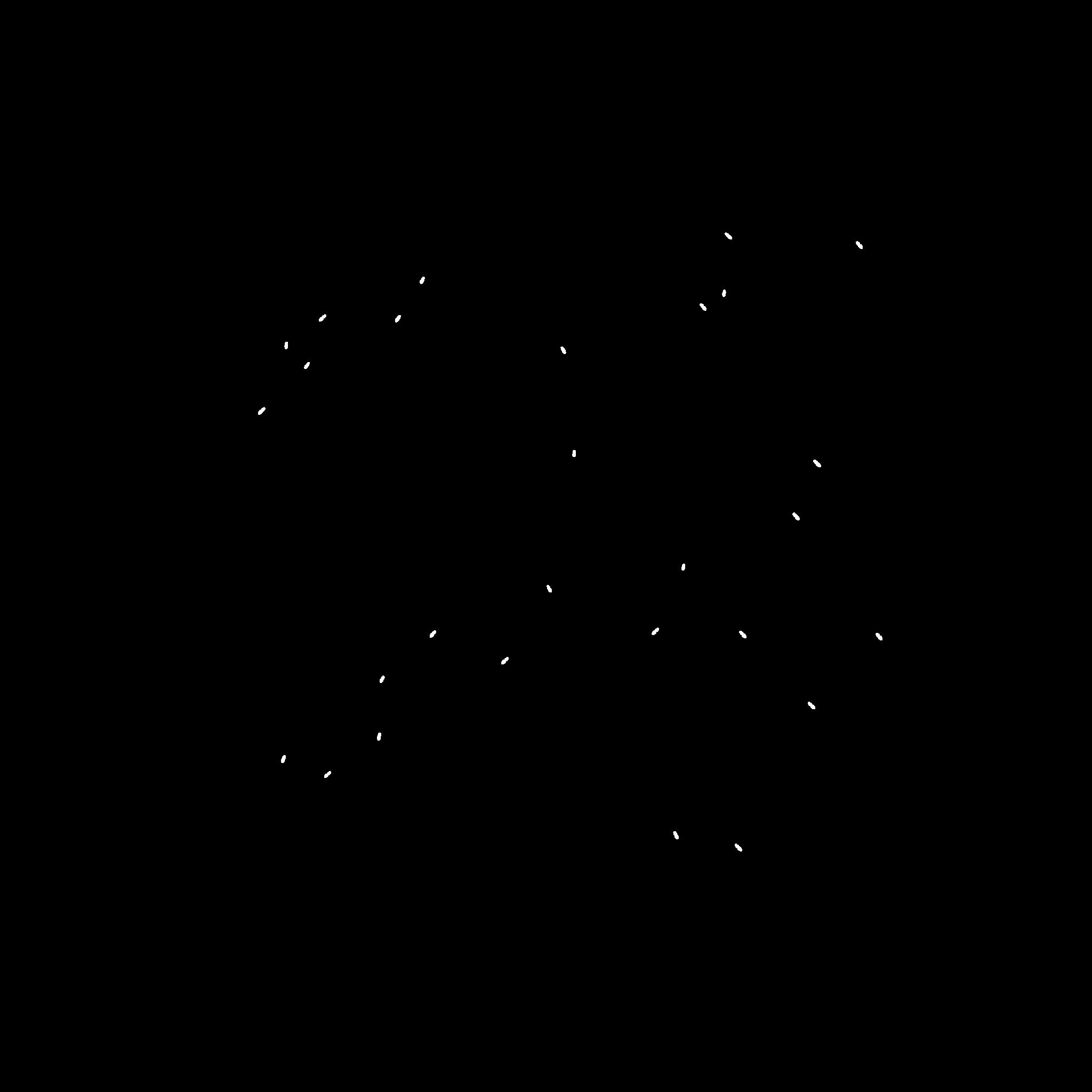

Supplement: S1 File — (ZIP) [file pone.0132101.s003.zip › ORsrc/nonortho/simu028/camx/imx041.jpg]

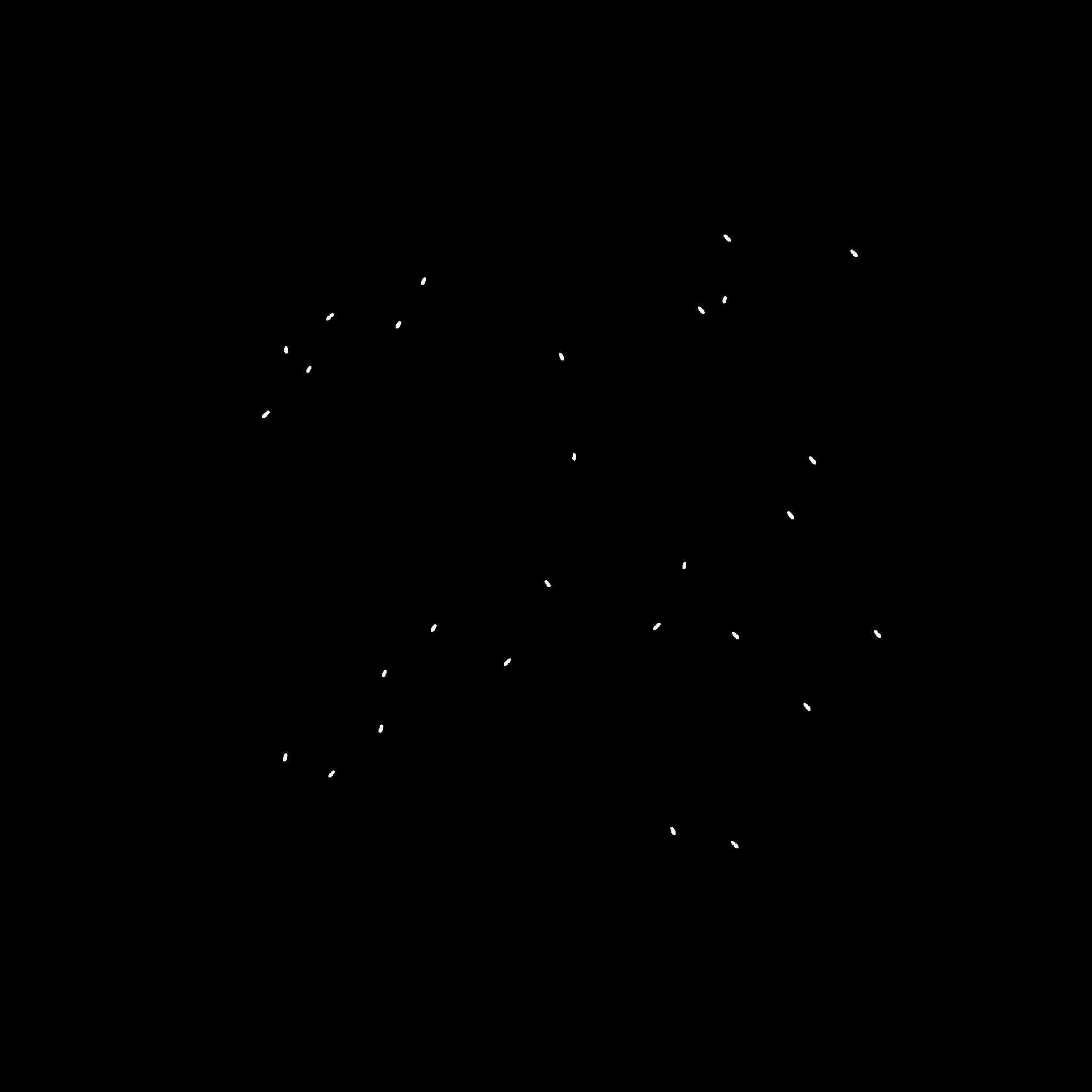

Supplement: S1 File — (ZIP) [file pone.0132101.s003.zip › ORsrc/nonortho/simu028/camx/imx042.jpg]

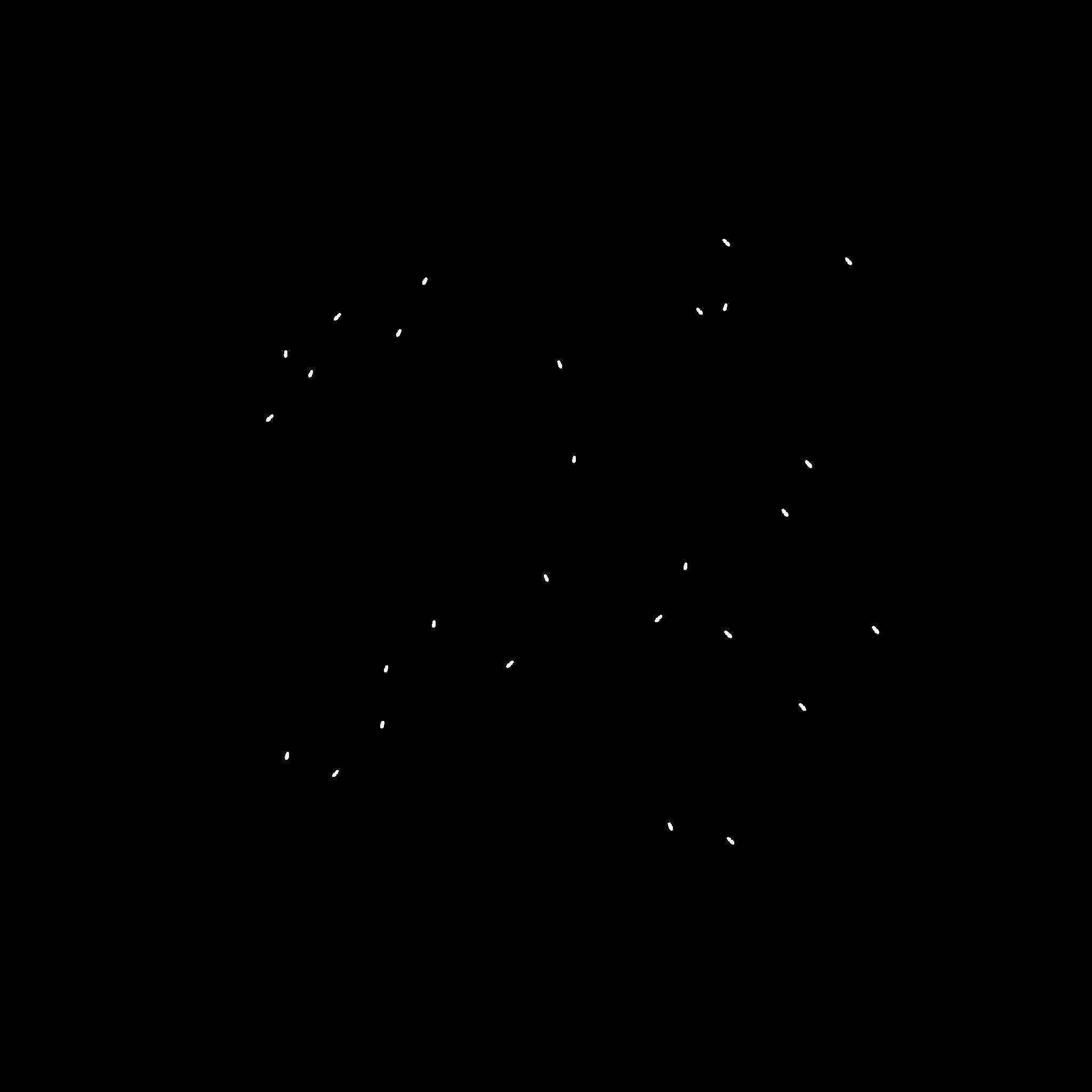

Supplement: S1 File — (ZIP) [file pone.0132101.s003.zip › ORsrc/nonortho/simu028/camx/imx043.jpg]

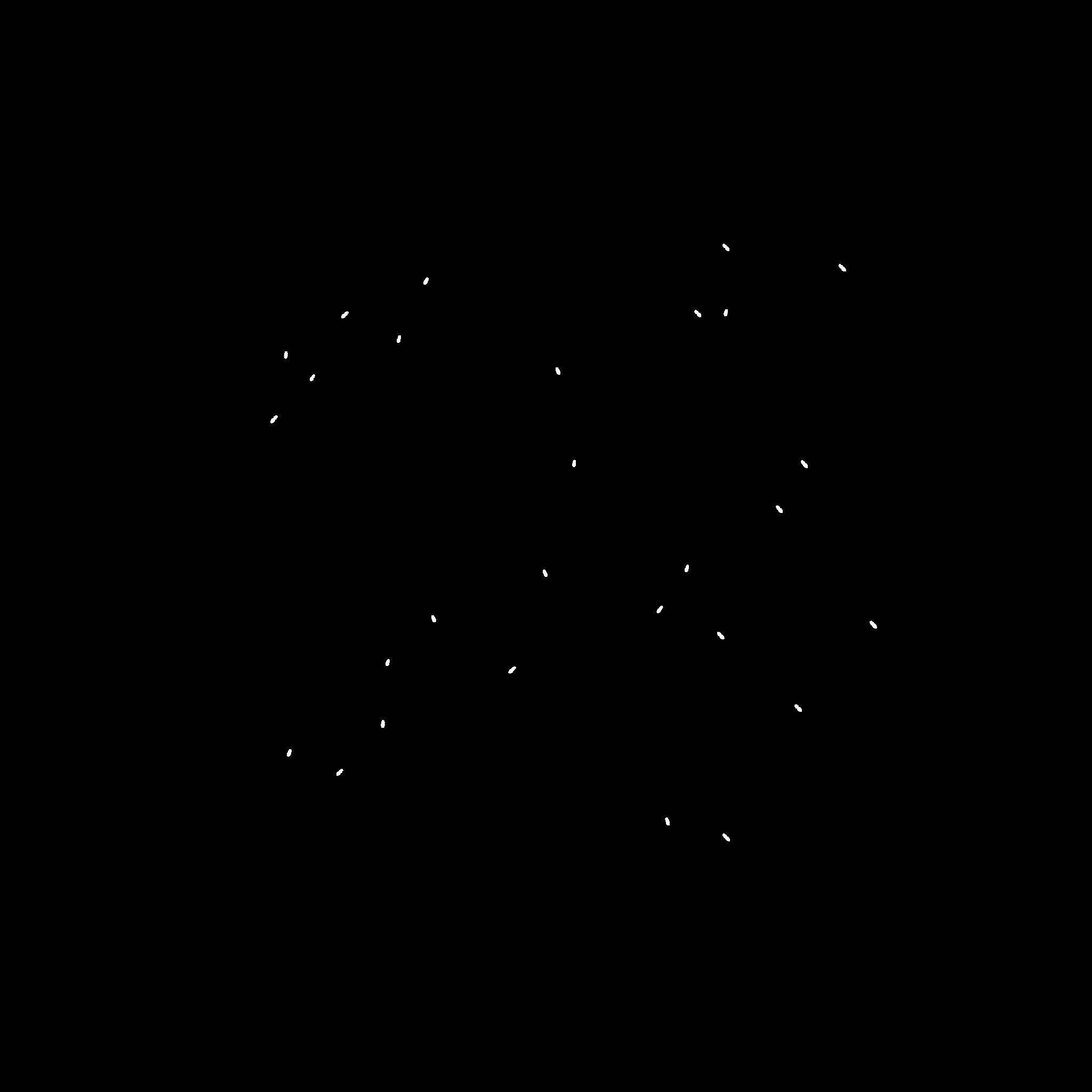

Supplement: S1 File — (ZIP) [file pone.0132101.s003.zip › ORsrc/nonortho/simu028/camx/imx044.jpg]

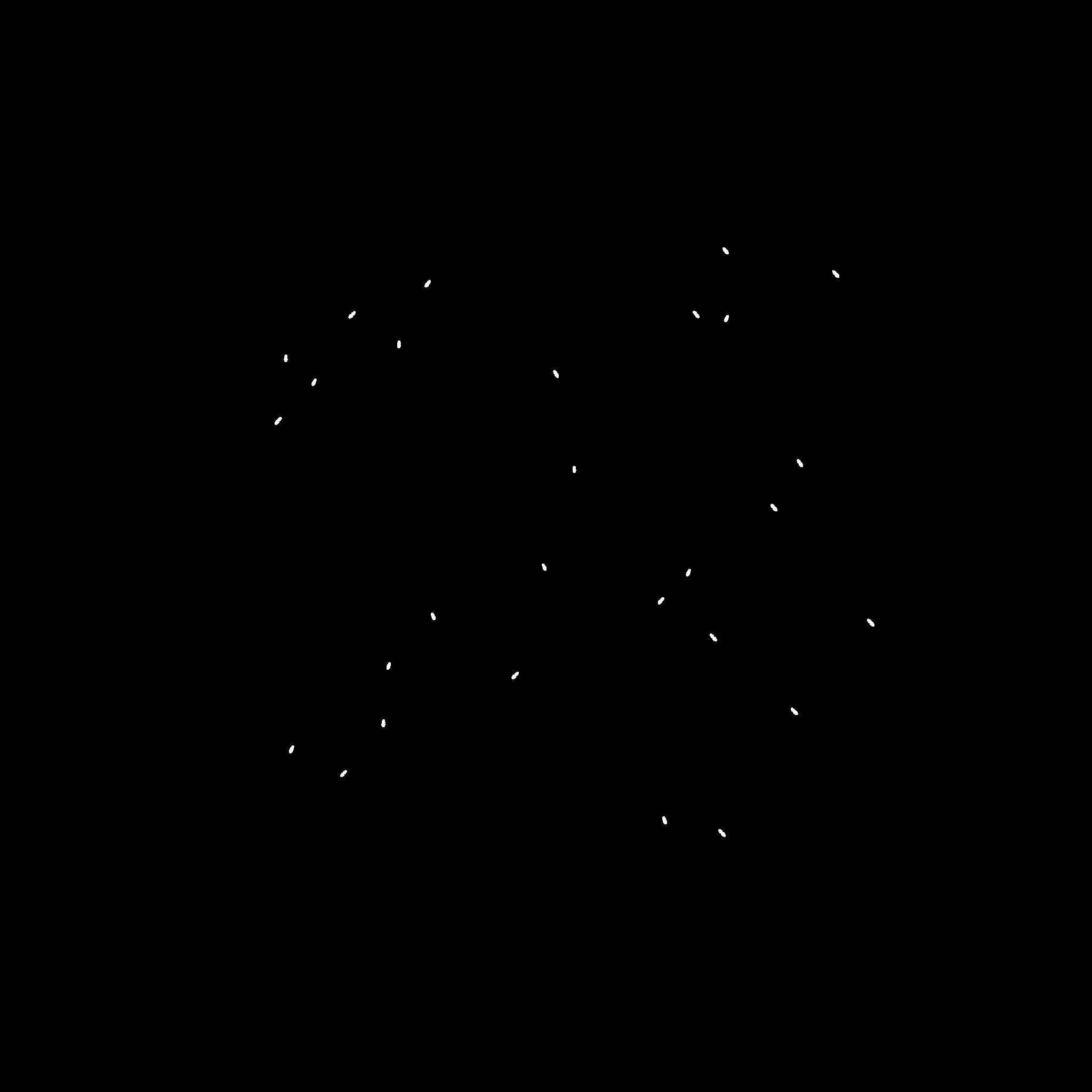

Supplement: S1 File — (ZIP) [file pone.0132101.s003.zip › ORsrc/nonortho/simu028/camx/imx045.jpg]

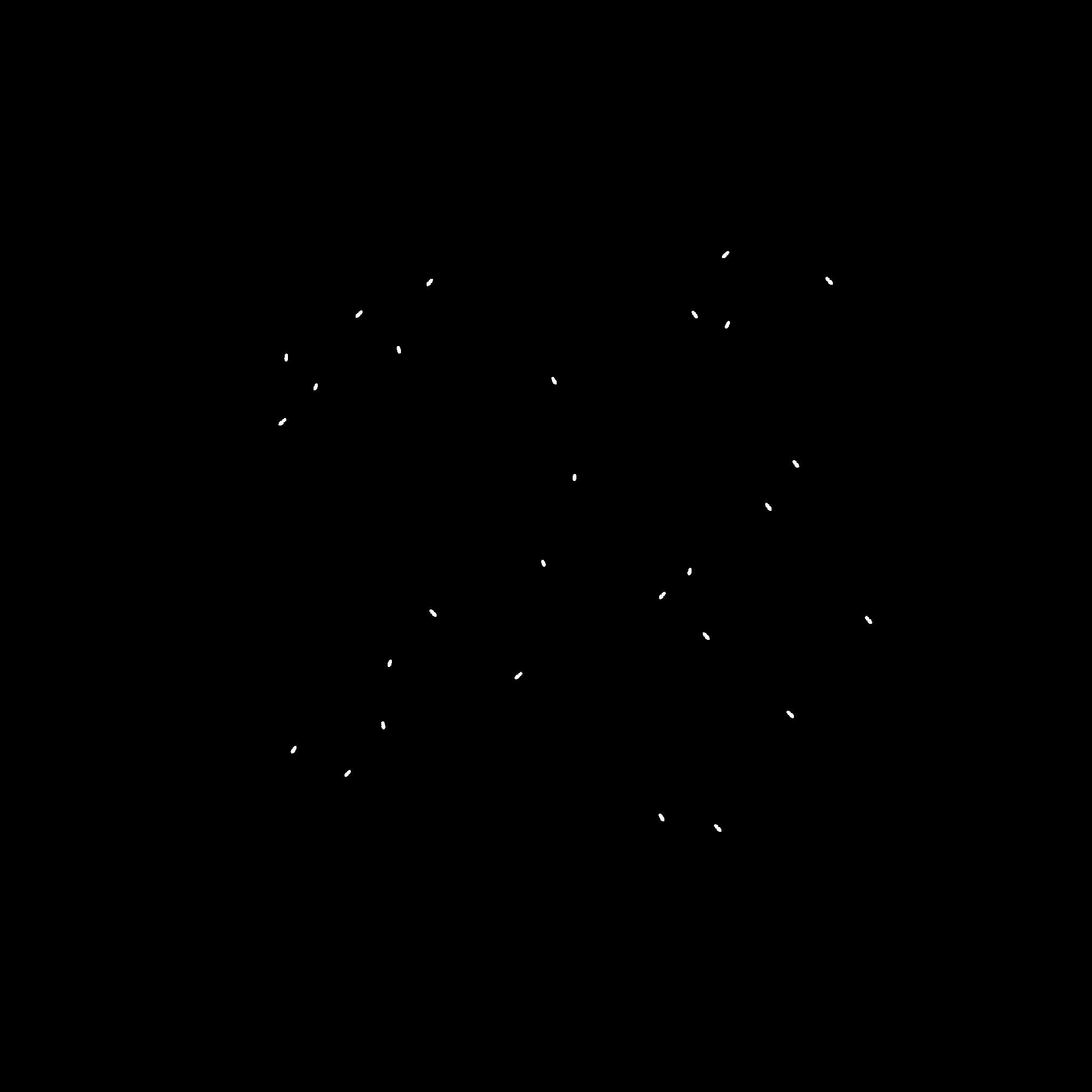

Supplement: S1 File — (ZIP) [file pone.0132101.s003.zip › ORsrc/nonortho/simu028/camx/imx046.jpg]

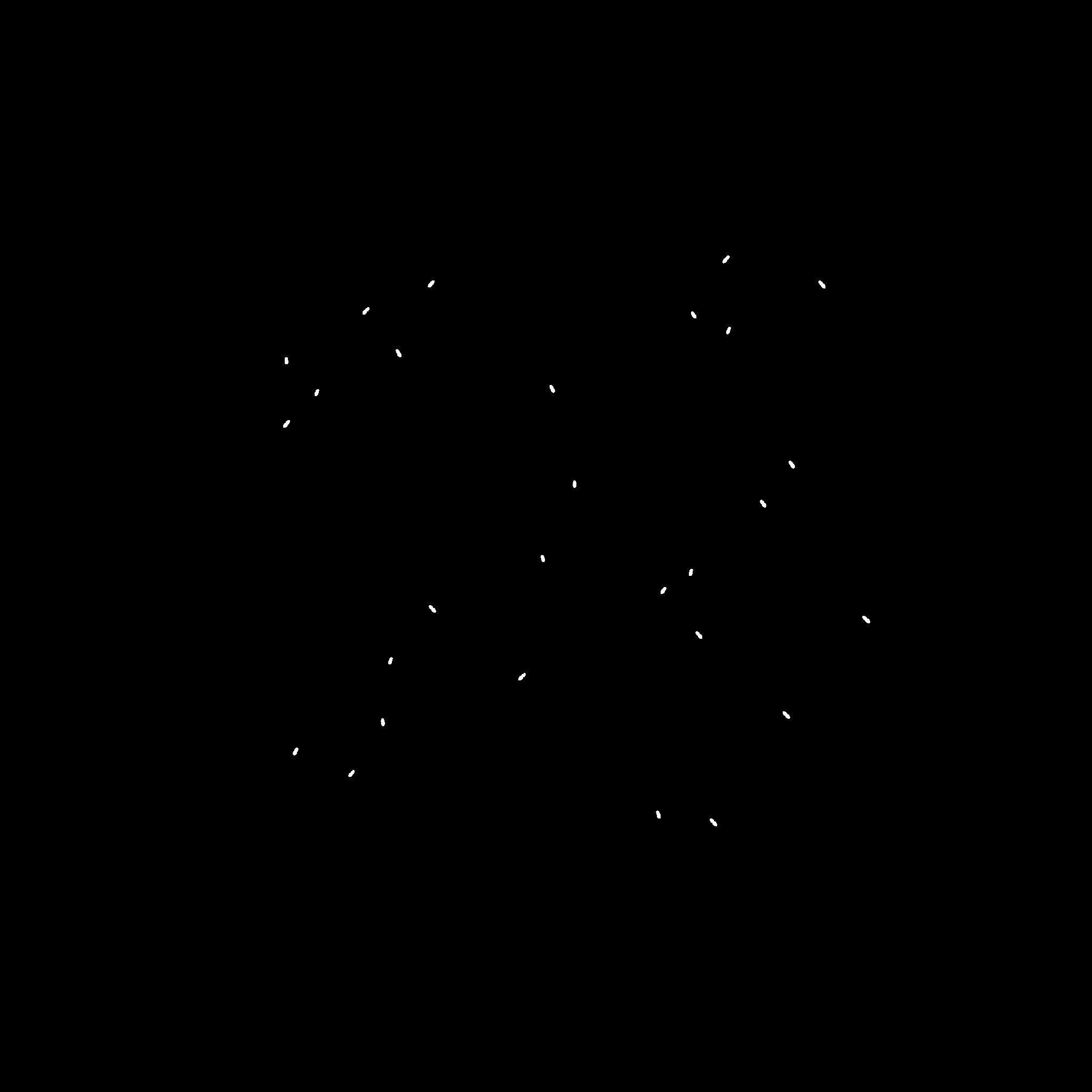

Supplement: S1 File — (ZIP) [file pone.0132101.s003.zip › ORsrc/nonortho/simu028/camx/imx047.jpg]

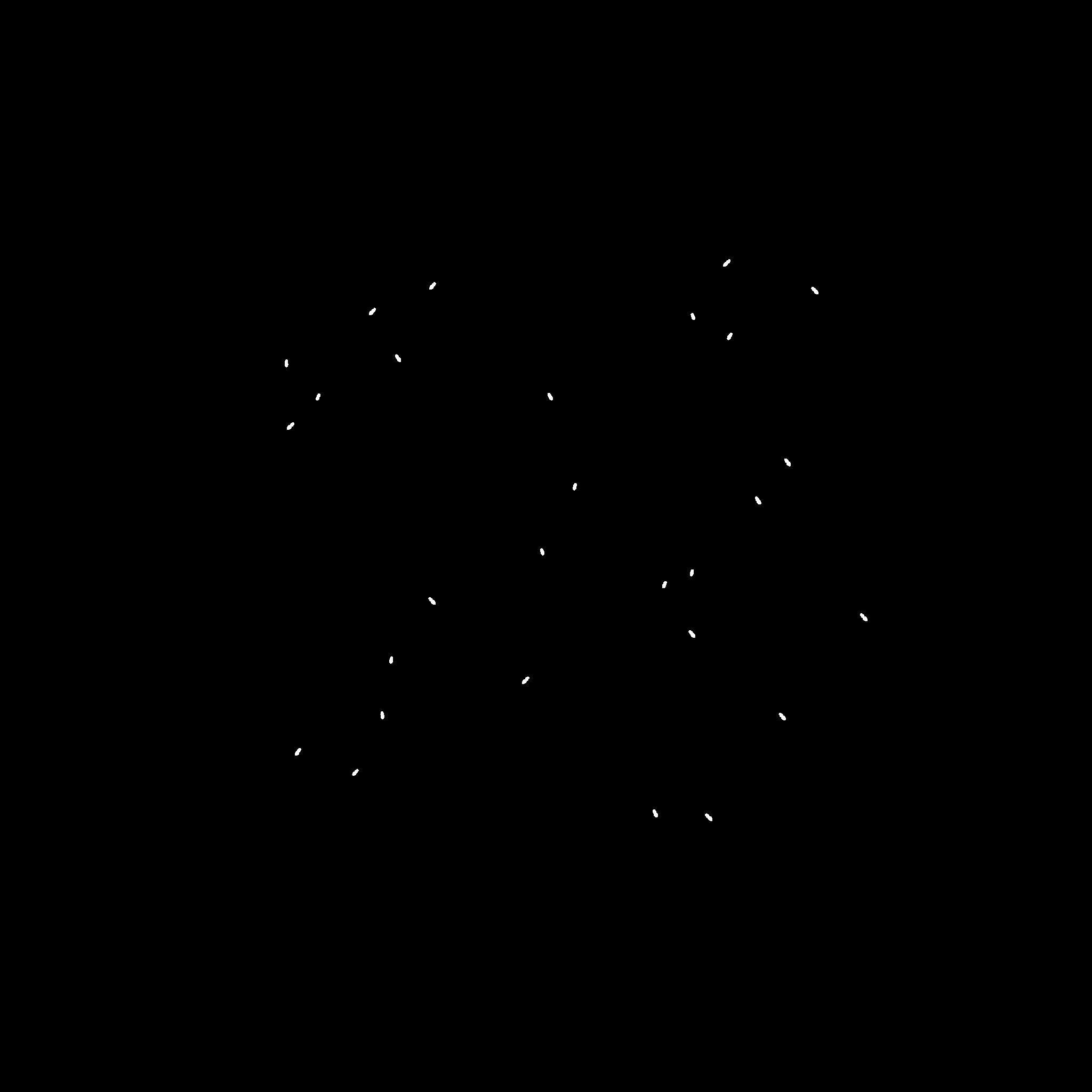

Supplement: S1 File — (ZIP) [file pone.0132101.s003.zip › ORsrc/nonortho/simu028/camx/imx048.jpg]

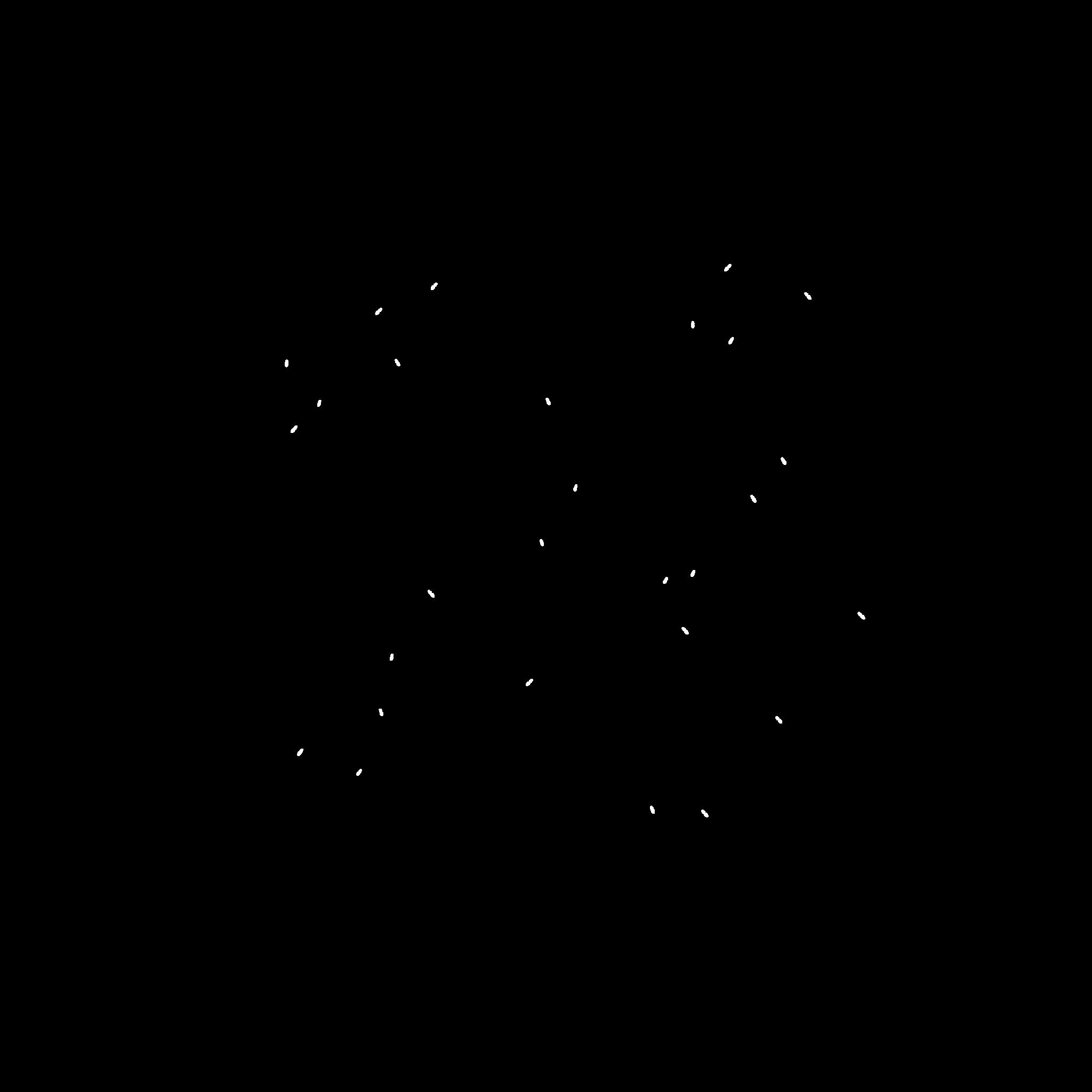

Supplement: S1 File — (ZIP) [file pone.0132101.s003.zip › ORsrc/nonortho/simu028/camx/imx049.jpg]

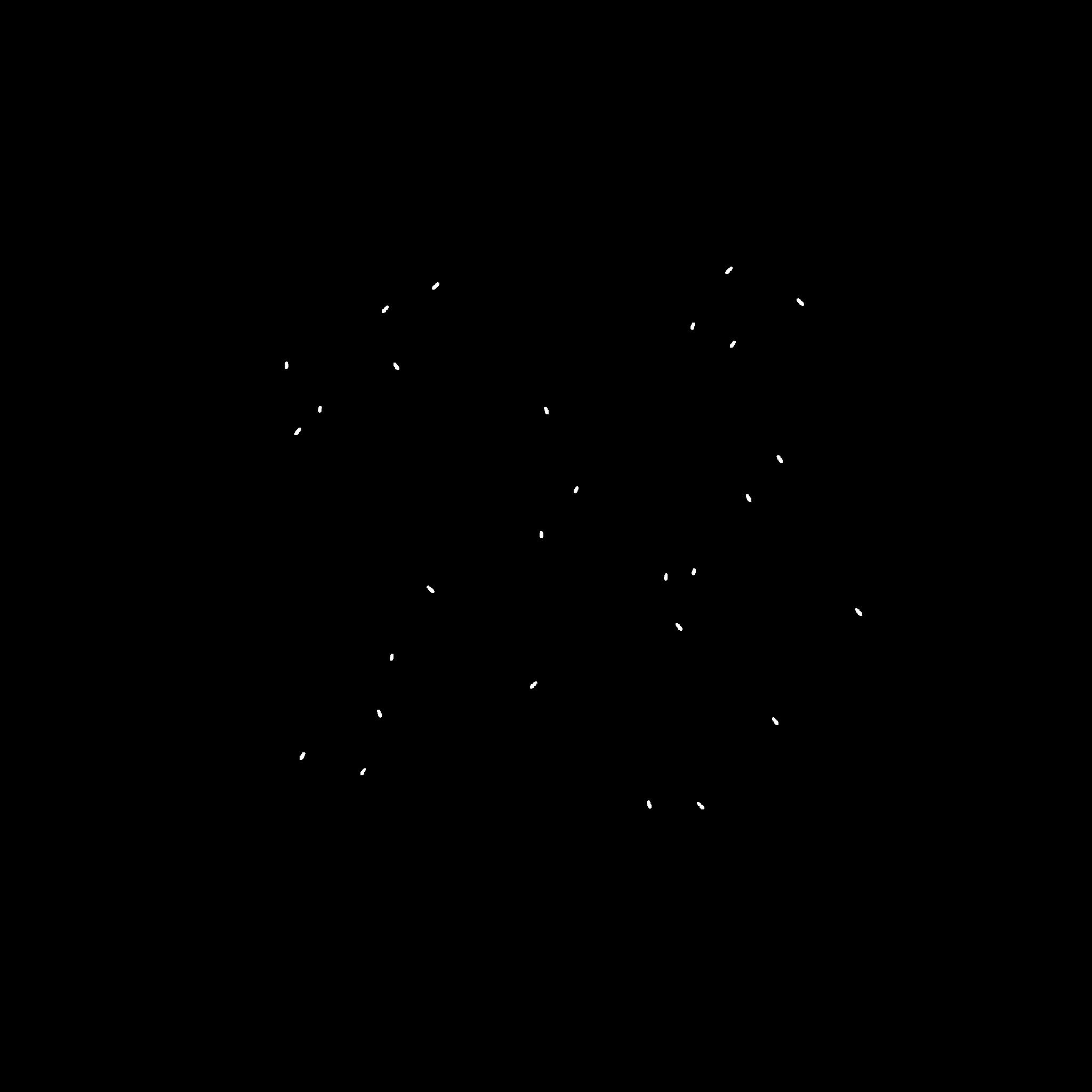

Supplement: S1 File — (ZIP) [file pone.0132101.s003.zip › ORsrc/nonortho/simu028/camx/imx050.jpg]

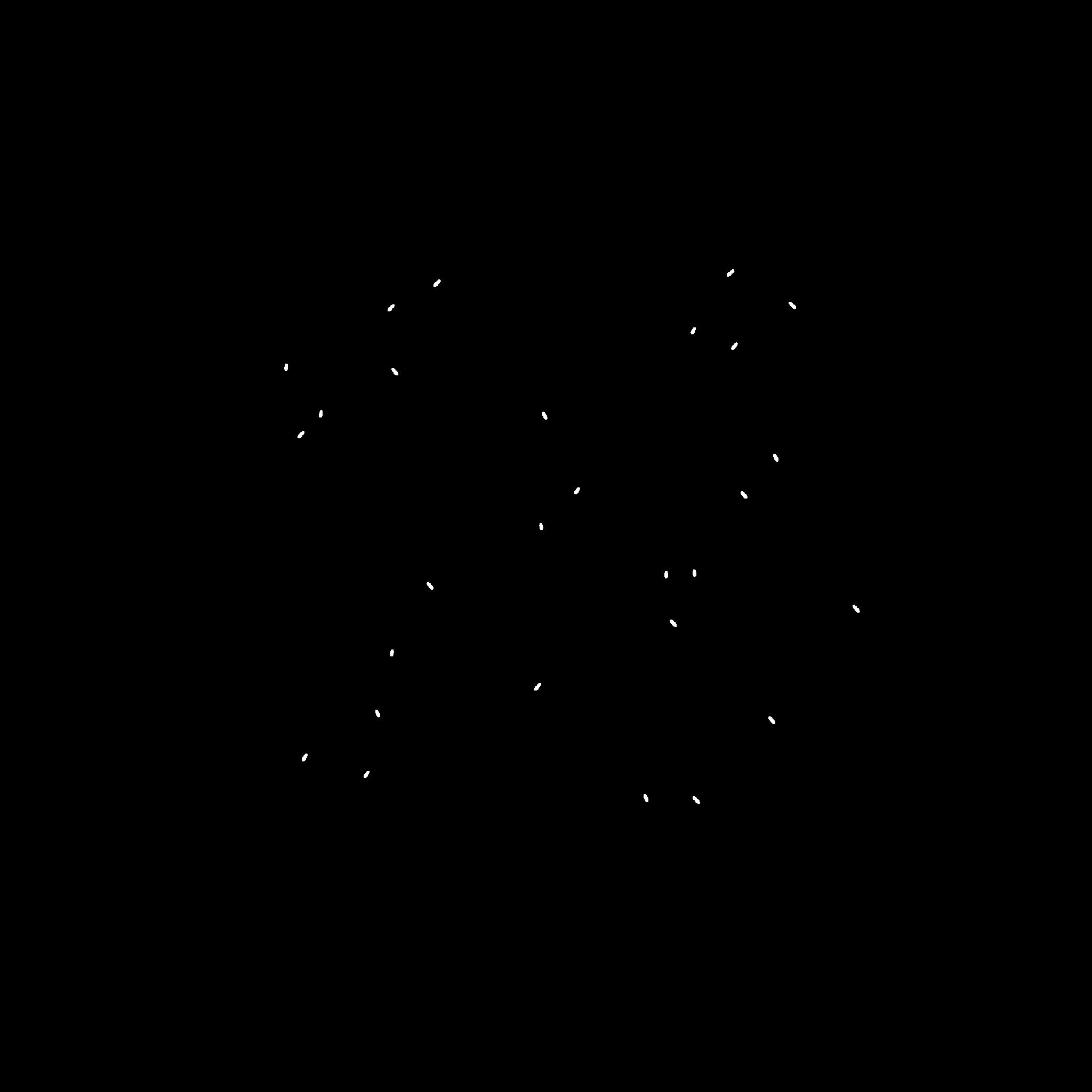

Supplement: S1 File — (ZIP) [file pone.0132101.s003.zip › ORsrc/nonortho/simu028/camx/imx051.jpg]

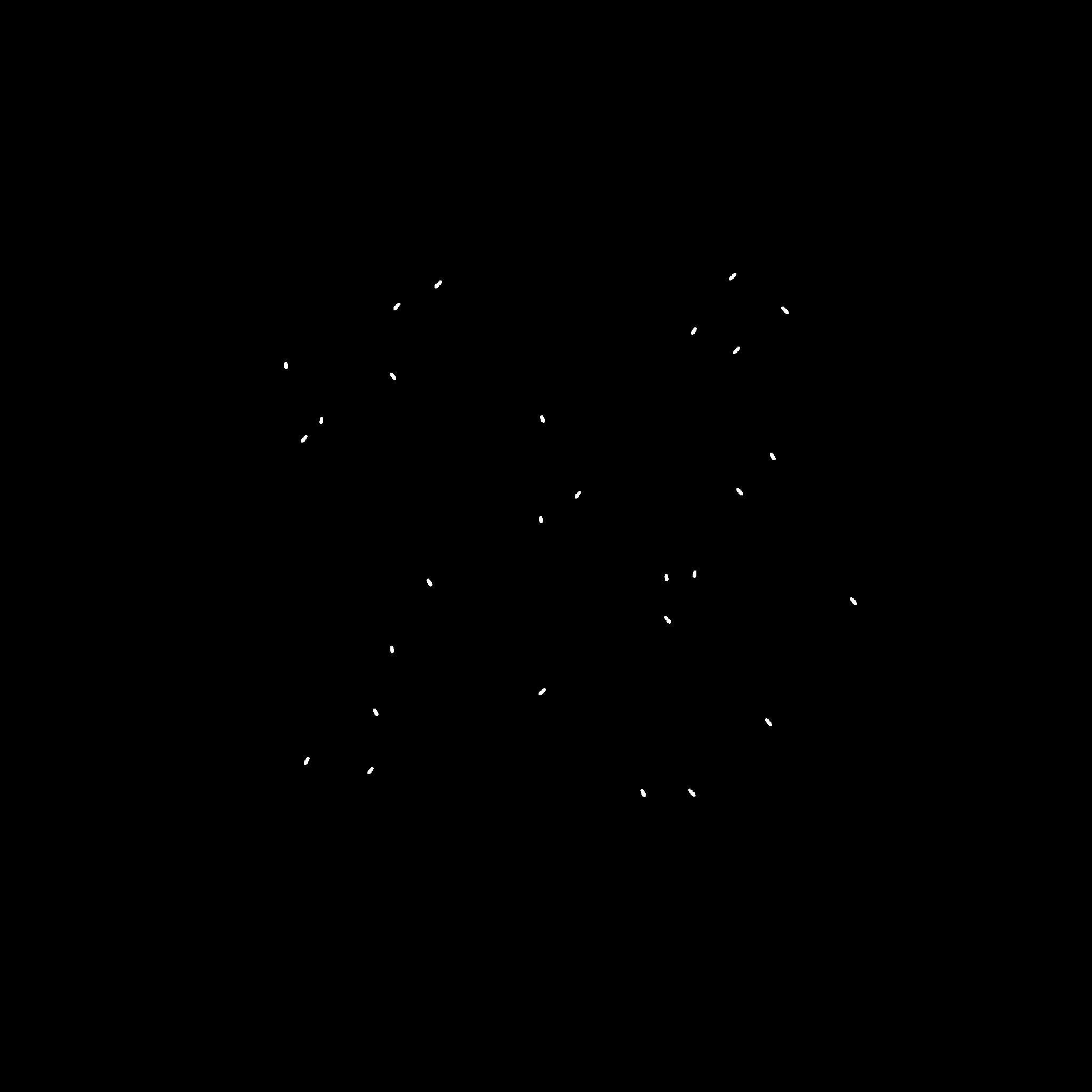

Supplement: S1 File — (ZIP) [file pone.0132101.s003.zip › ORsrc/nonortho/simu028/camx/imx052.jpg]

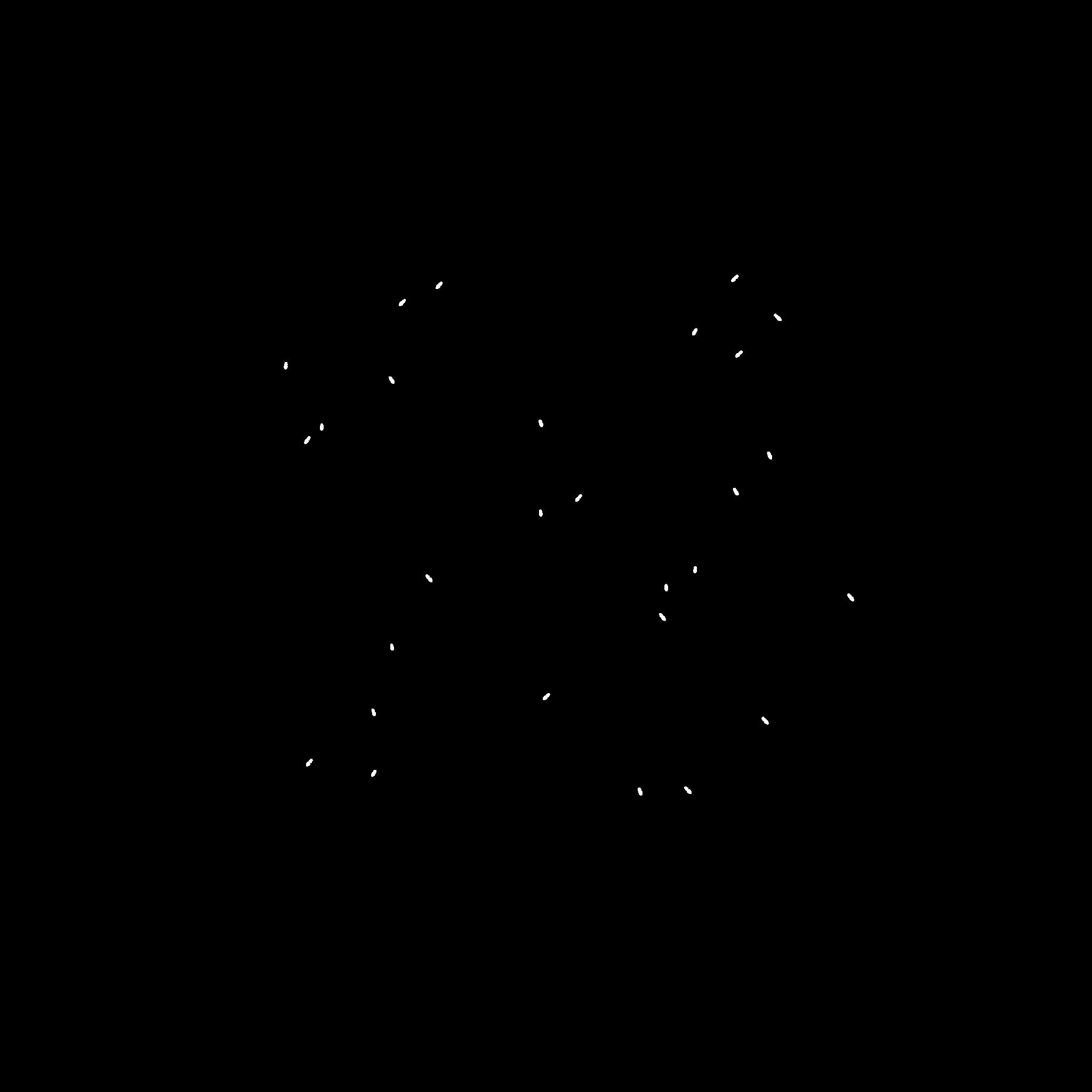

Supplement: S1 File — (ZIP) [file pone.0132101.s003.zip › ORsrc/nonortho/simu028/camx/imx053.jpg]

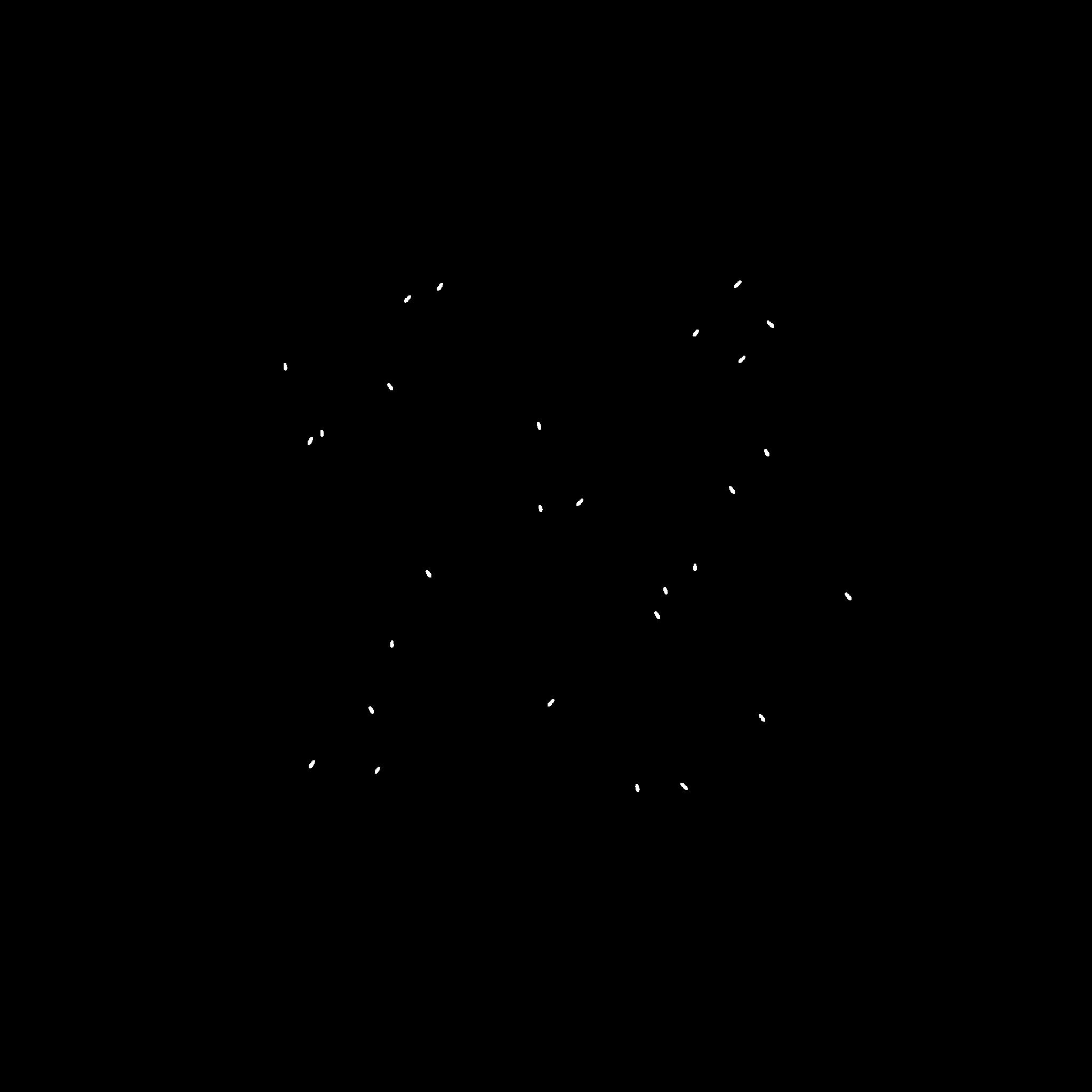

Supplement: S1 File — (ZIP) [file pone.0132101.s003.zip › ORsrc/nonortho/simu028/camx/imx054.jpg]

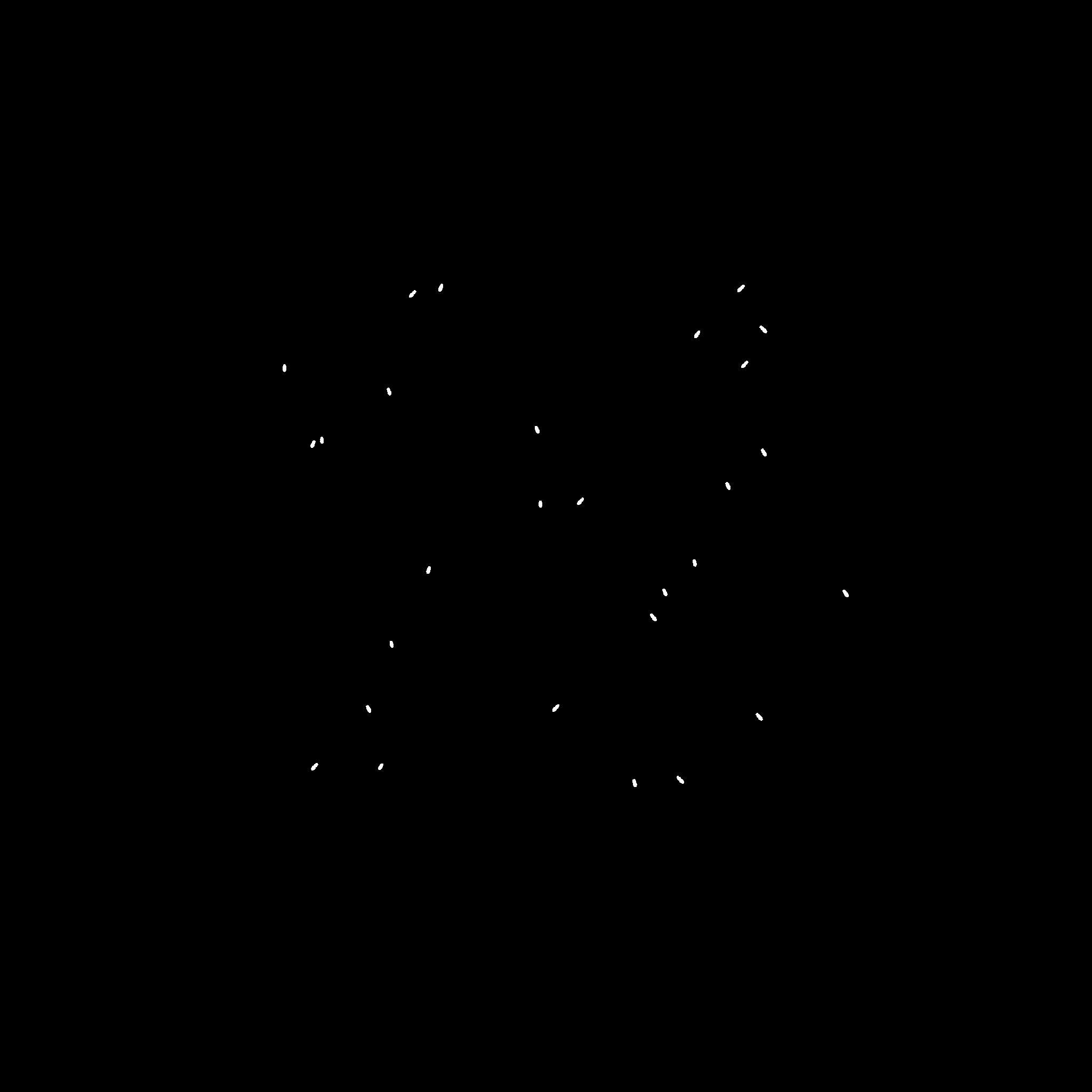

Supplement: S1 File — (ZIP) [file pone.0132101.s003.zip › ORsrc/nonortho/simu028/camx/imx055.jpg]

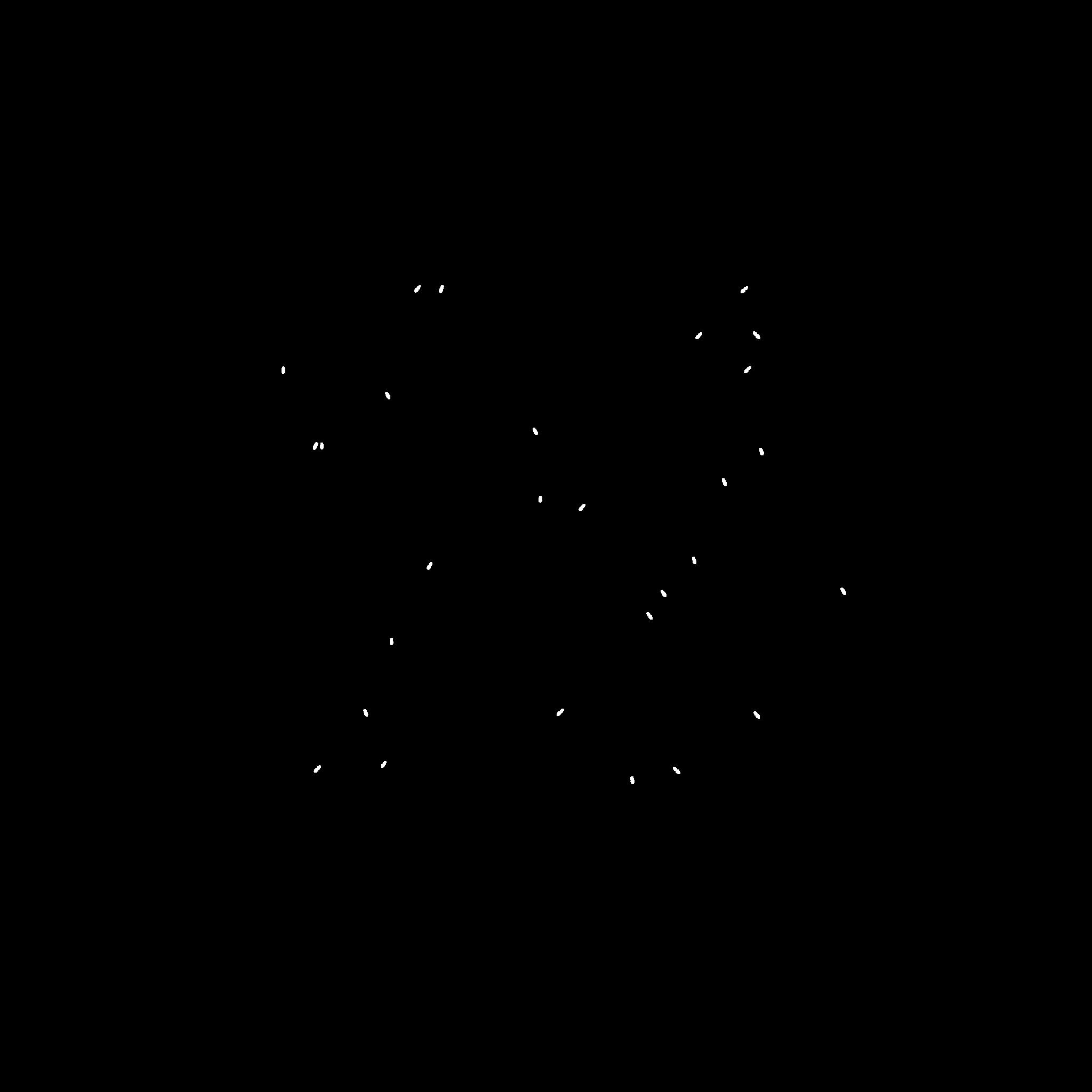

Supplement: S1 File — (ZIP) [file pone.0132101.s003.zip › ORsrc/nonortho/simu028/camx/imx056.jpg]

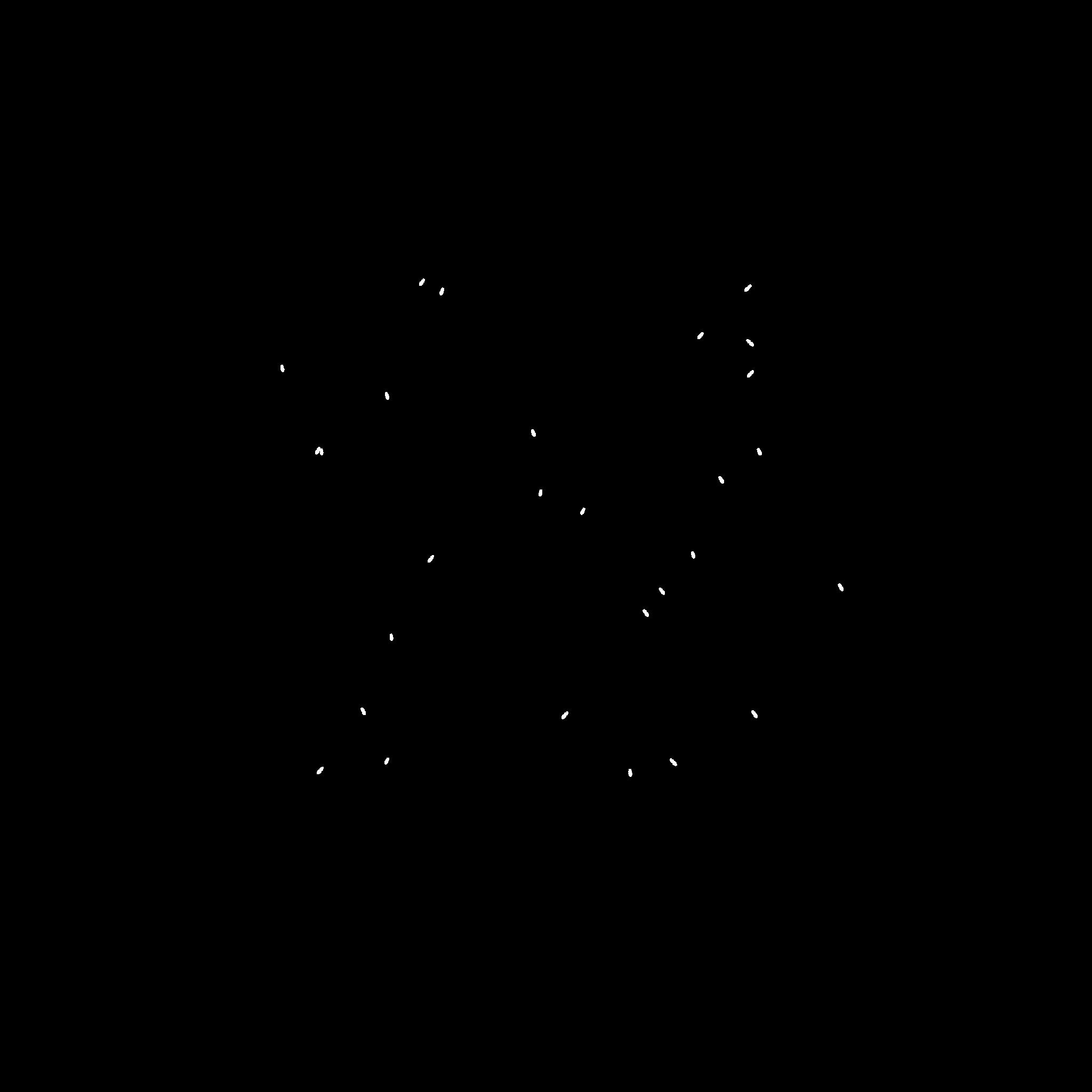

Supplement: S1 File — (ZIP) [file pone.0132101.s003.zip › ORsrc/nonortho/simu028/camx/imx057.jpg]

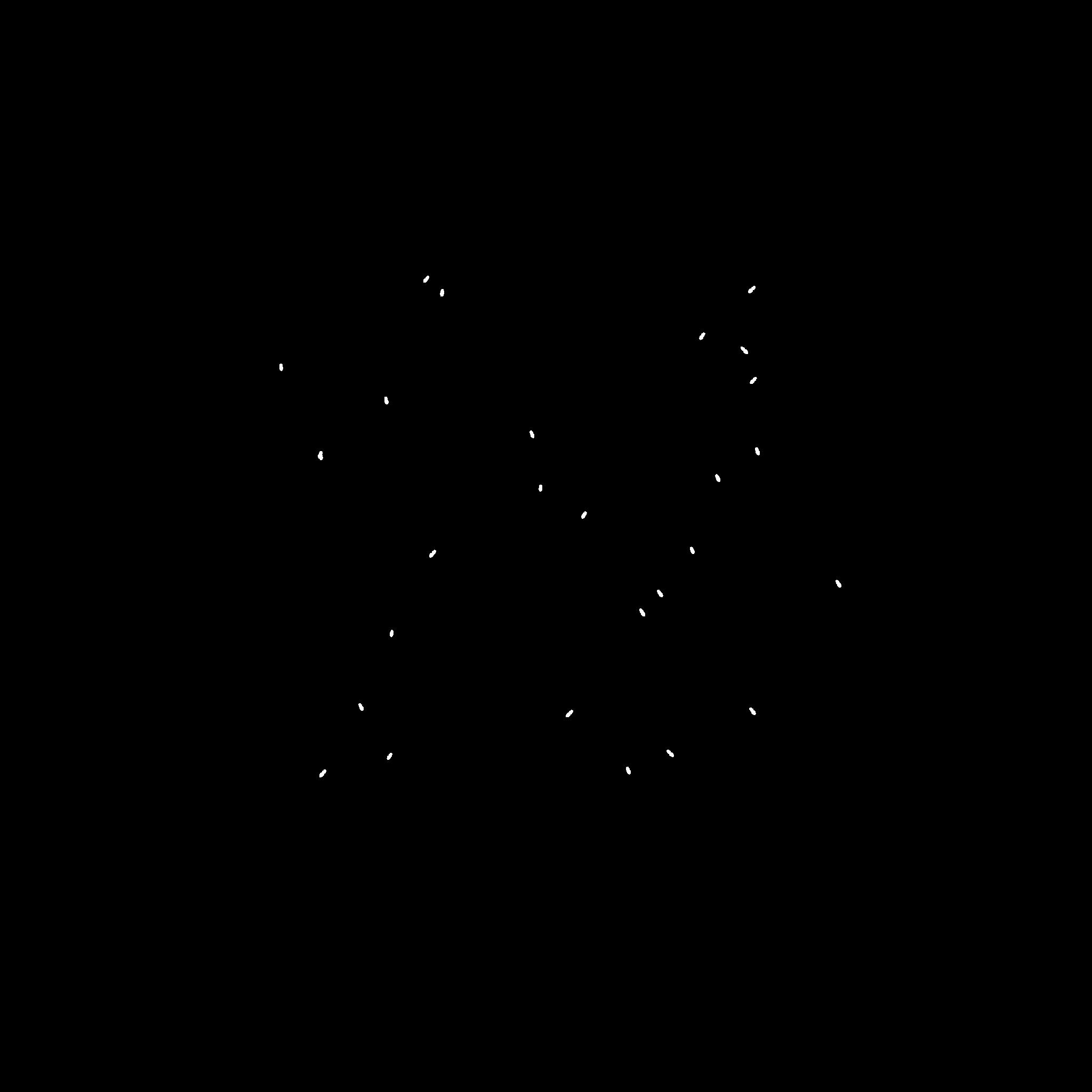

Supplement: S1 File — (ZIP) [file pone.0132101.s003.zip › ORsrc/nonortho/simu028/camx/imx058.jpg]

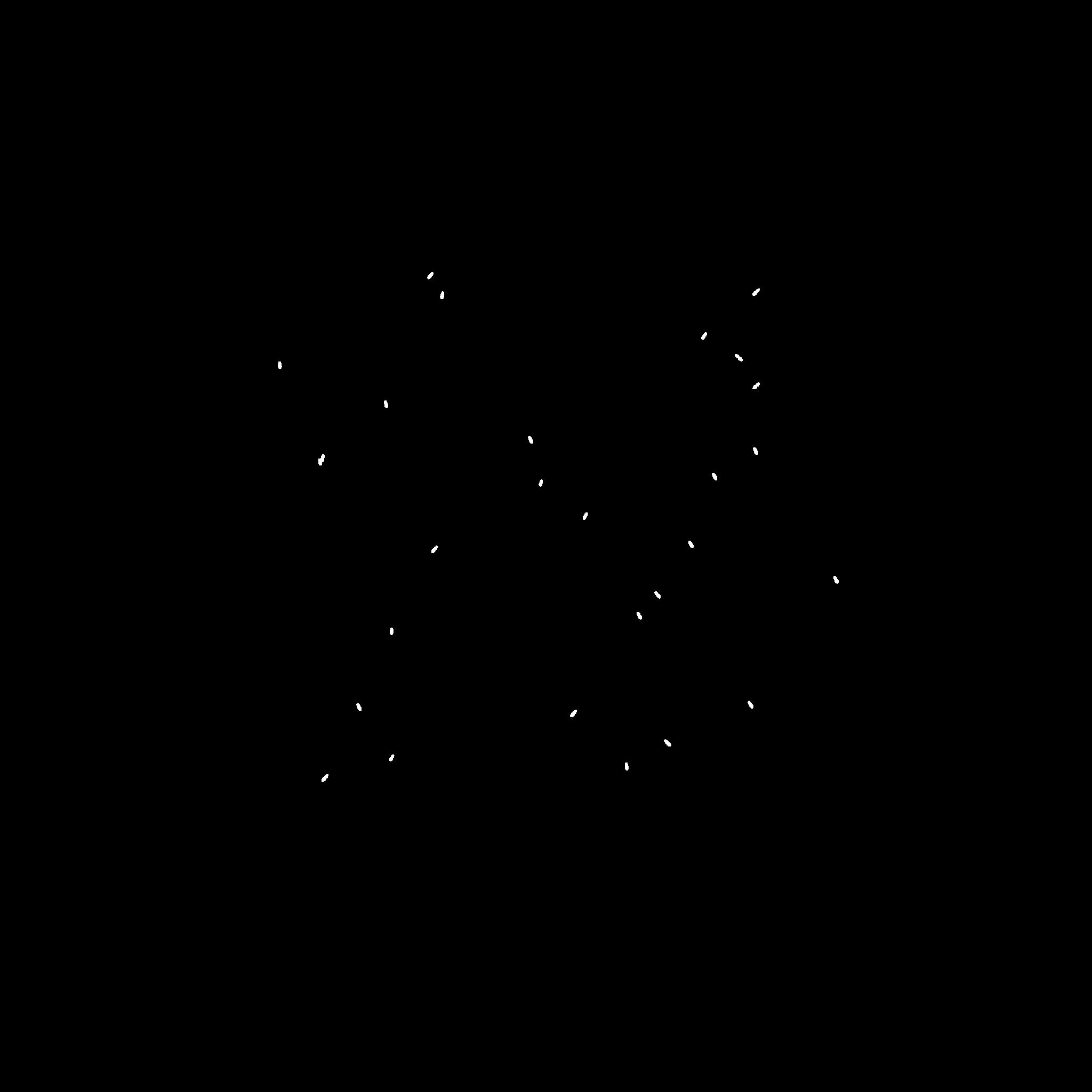

Supplement: S1 File — (ZIP) [file pone.0132101.s003.zip › ORsrc/nonortho/simu028/camx/imx059.jpg]

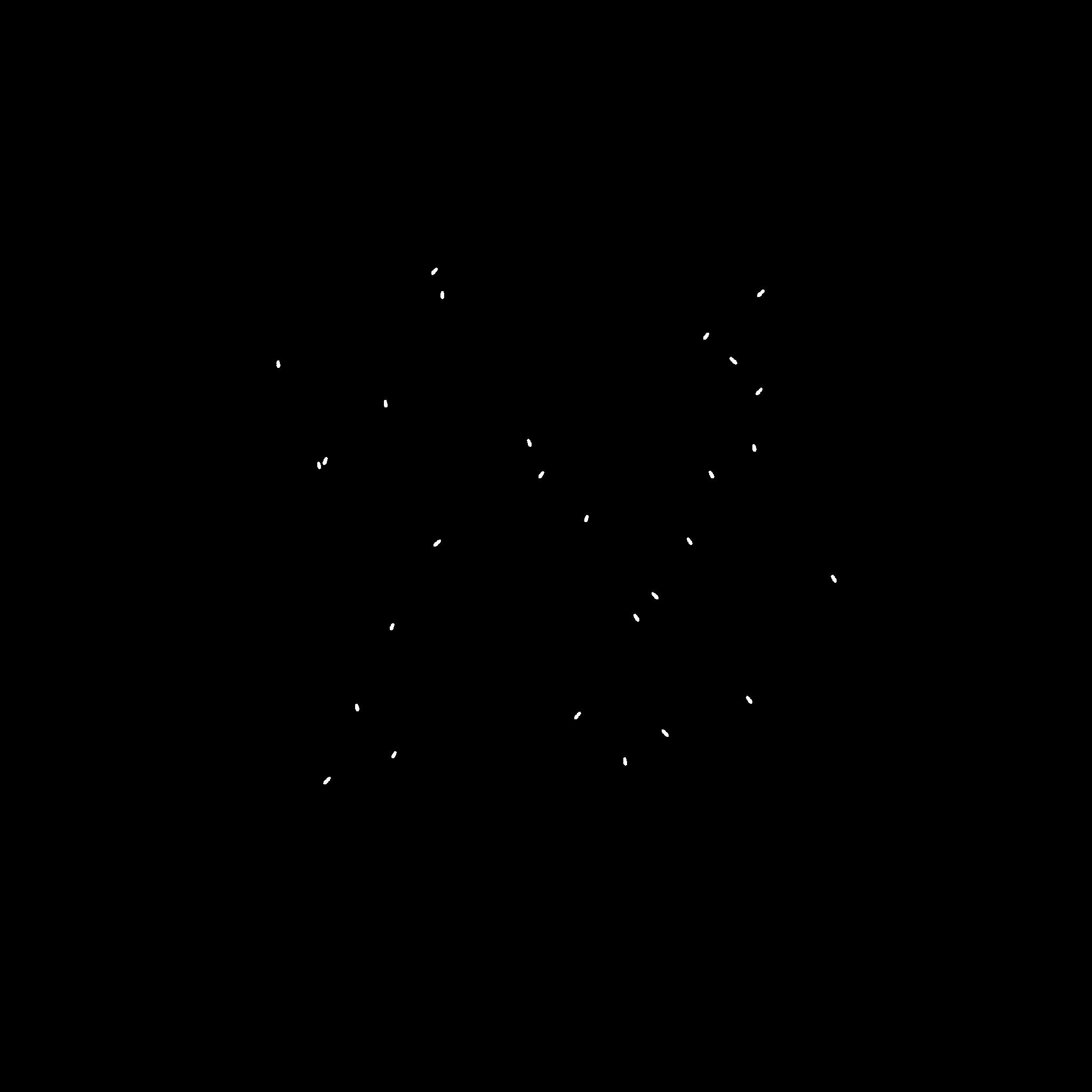

Supplement: S1 File — (ZIP) [file pone.0132101.s003.zip › ORsrc/nonortho/simu028/camx/imx060.jpg]

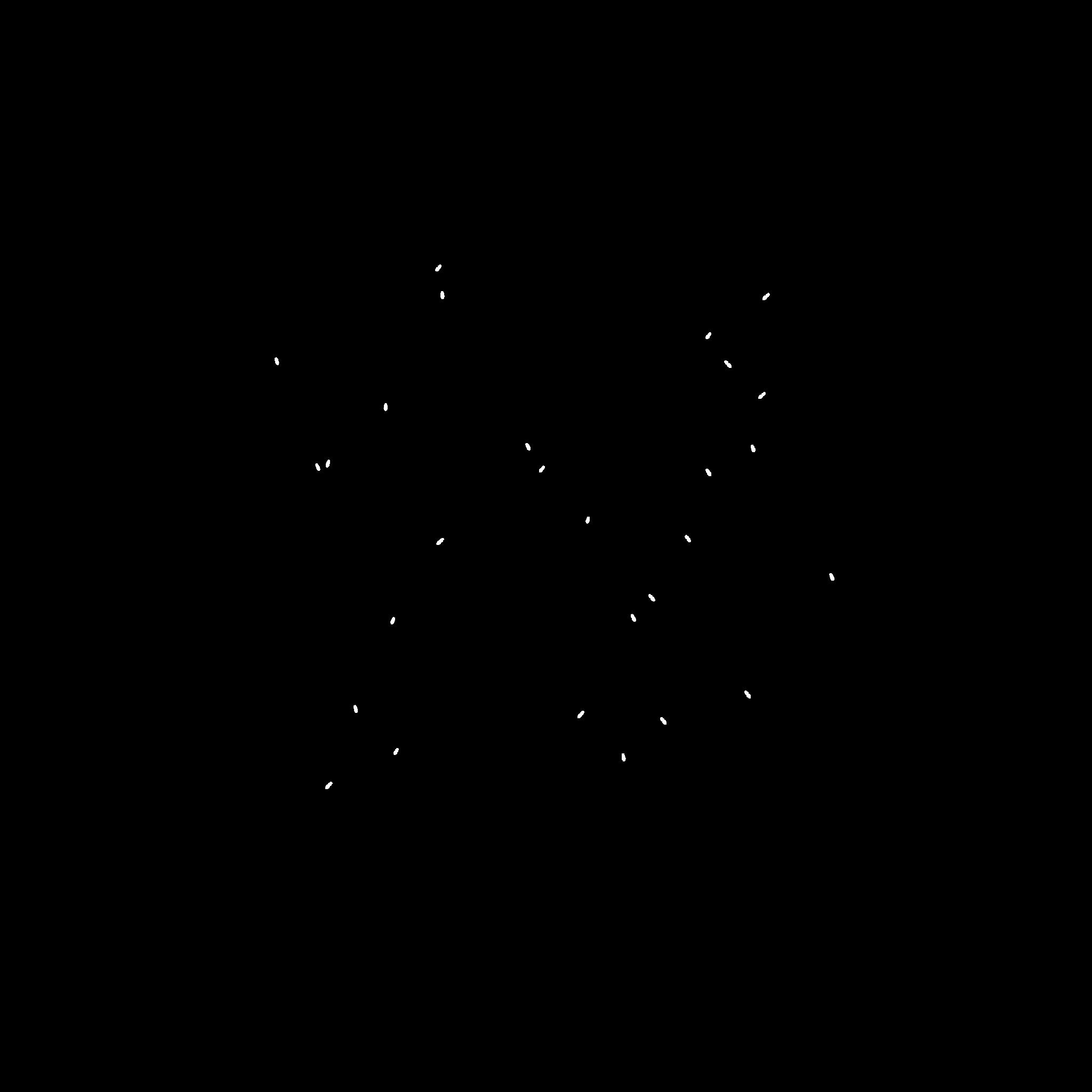

Supplement: S1 File — (ZIP) [file pone.0132101.s003.zip › ORsrc/nonortho/simu028/camx/imx061.jpg]

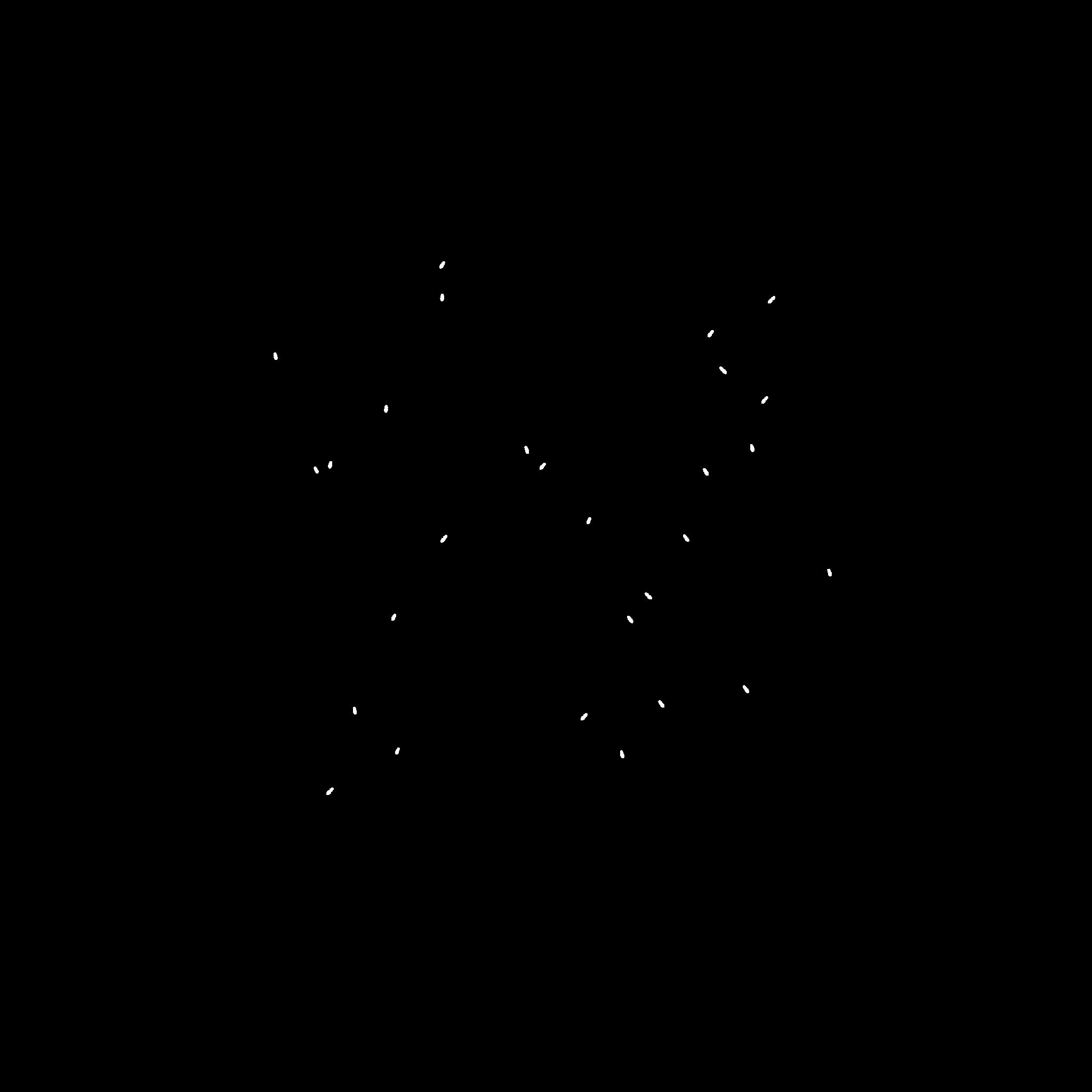

Supplement: S1 File — (ZIP) [file pone.0132101.s003.zip › ORsrc/nonortho/simu028/camx/imx062.jpg]

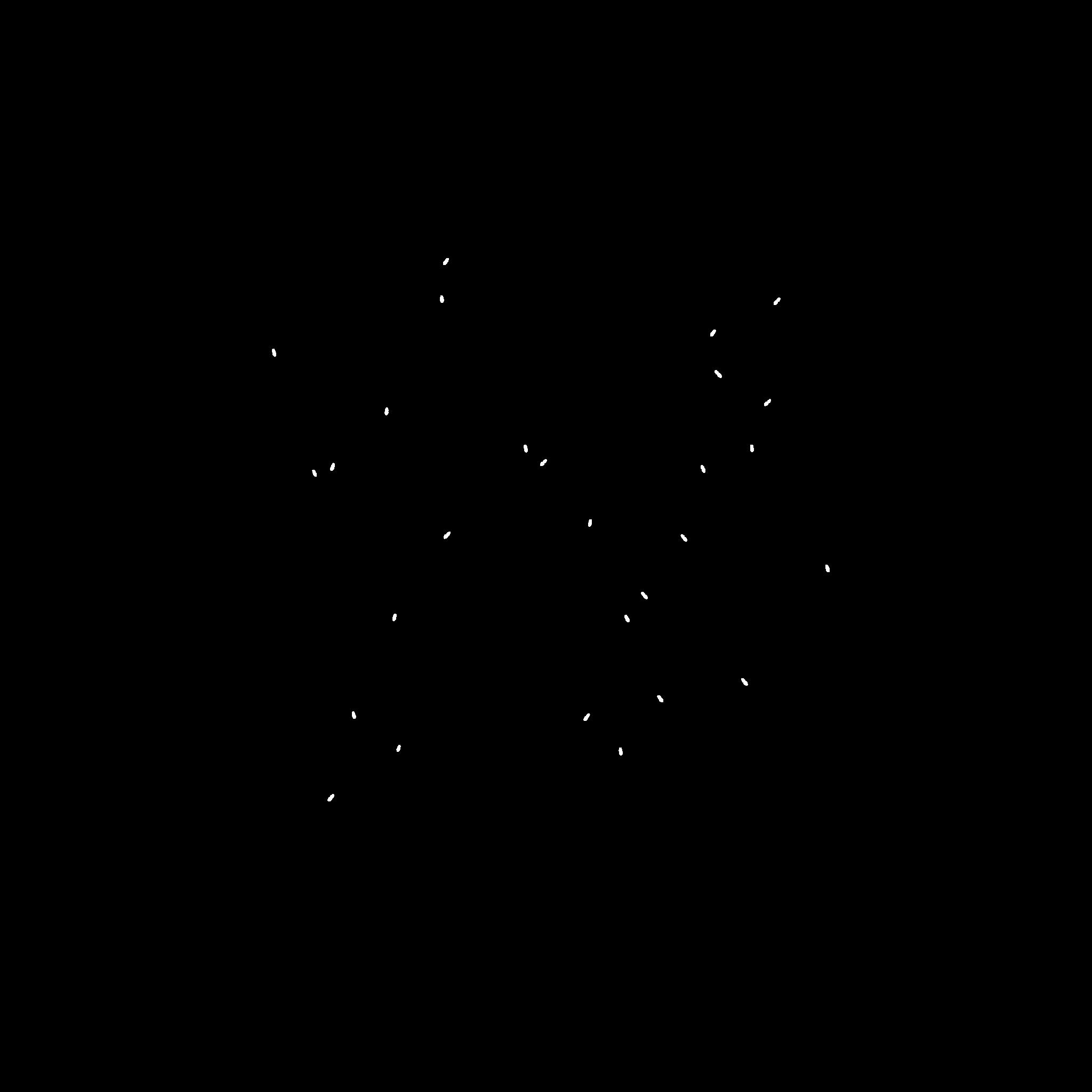

Supplement: S1 File — (ZIP) [file pone.0132101.s003.zip › ORsrc/nonortho/simu028/camx/imx063.jpg]

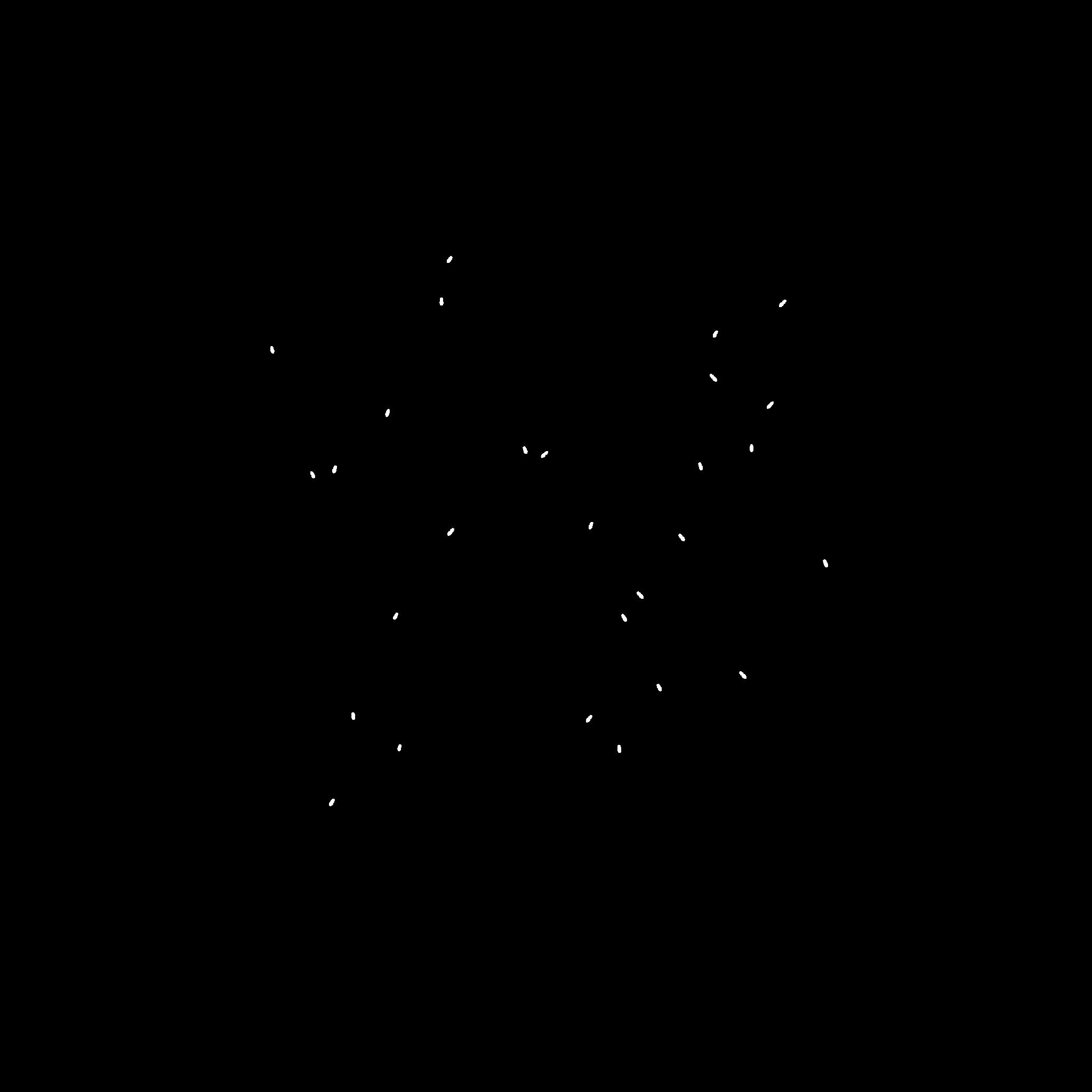

Supplement: S1 File — (ZIP) [file pone.0132101.s003.zip › ORsrc/nonortho/simu028/camx/imx064.jpg]

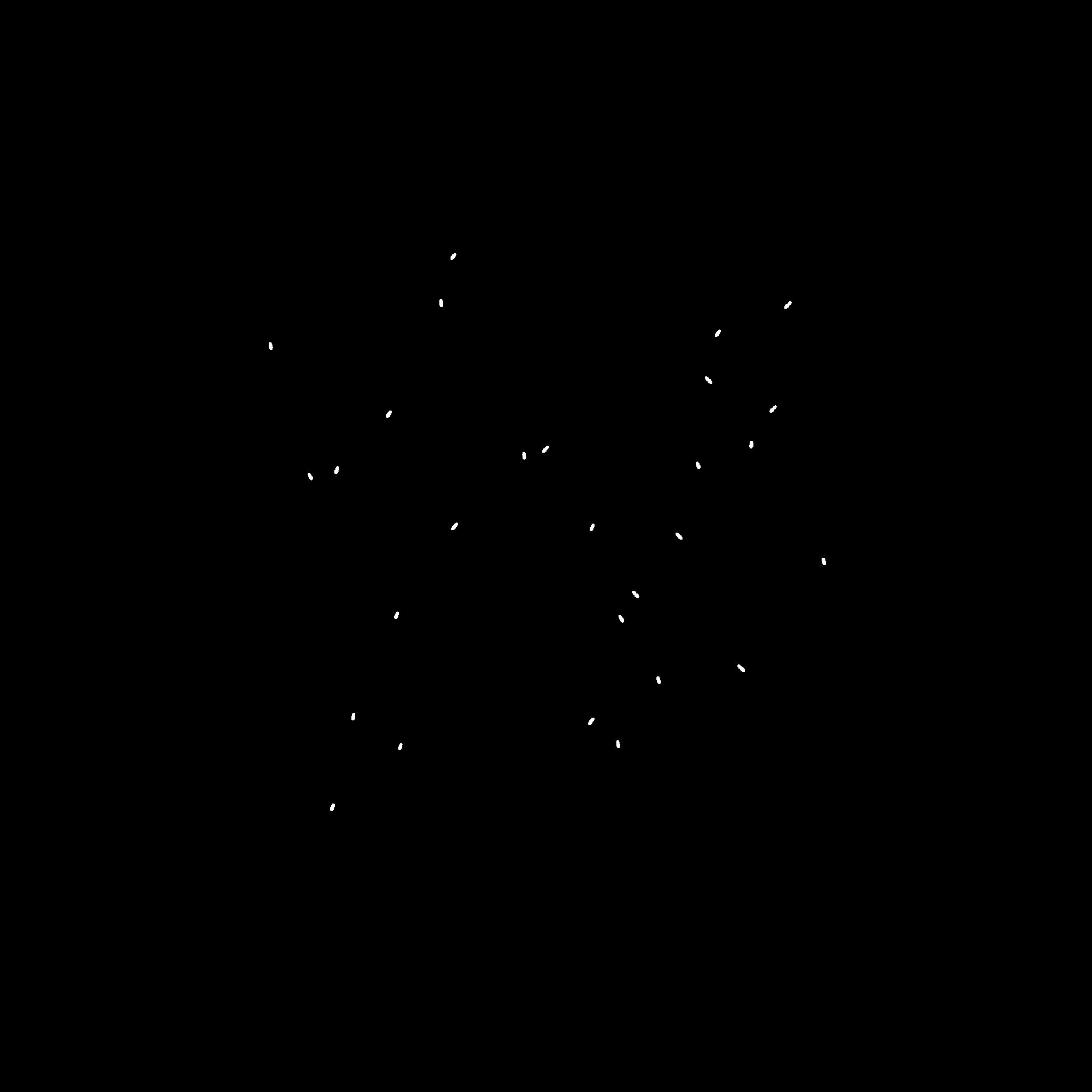

Supplement: S1 File — (ZIP) [file pone.0132101.s003.zip › ORsrc/nonortho/simu028/camx/imx065.jpg]

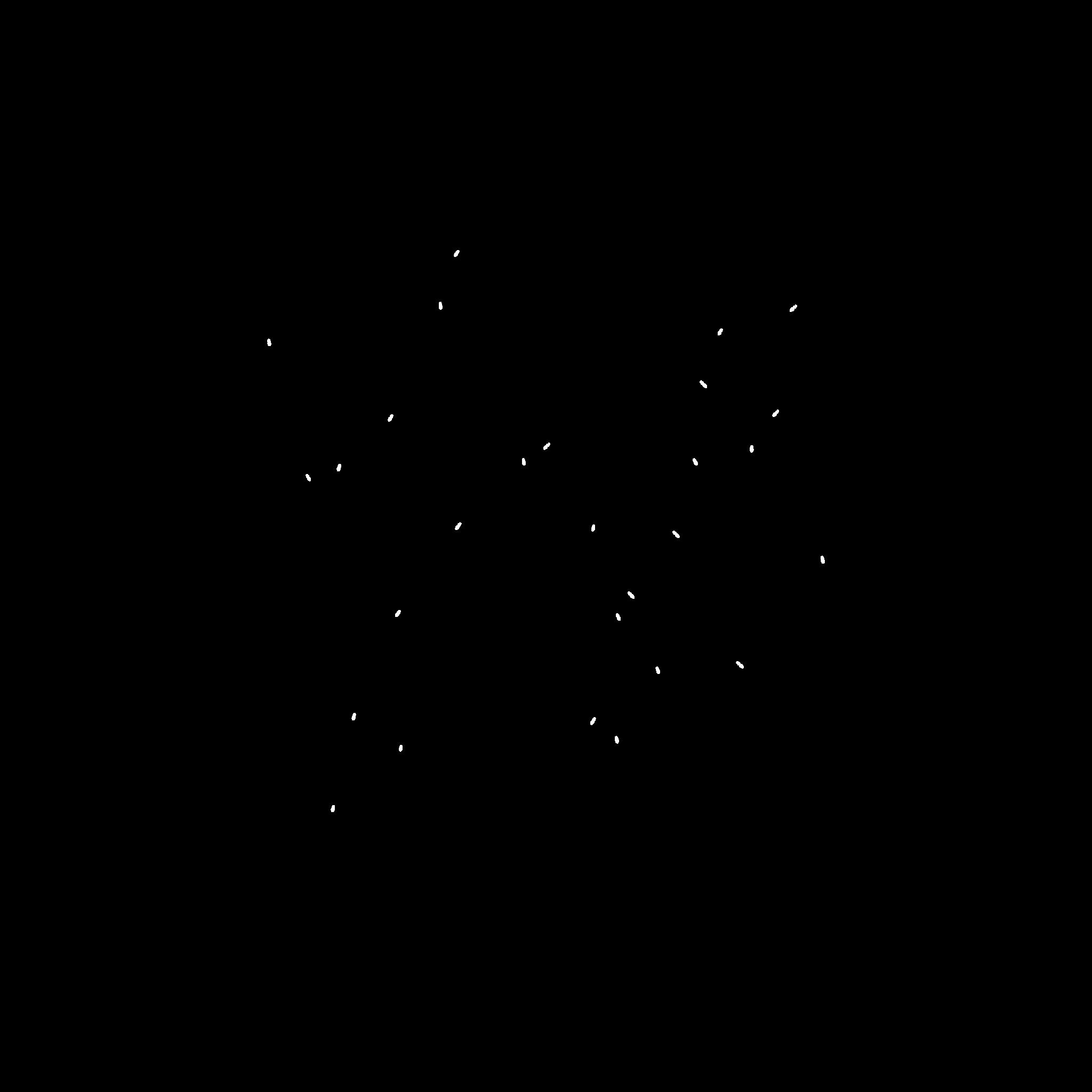

Supplement: S1 File — (ZIP) [file pone.0132101.s003.zip › ORsrc/nonortho/simu028/camx/imx066.jpg]

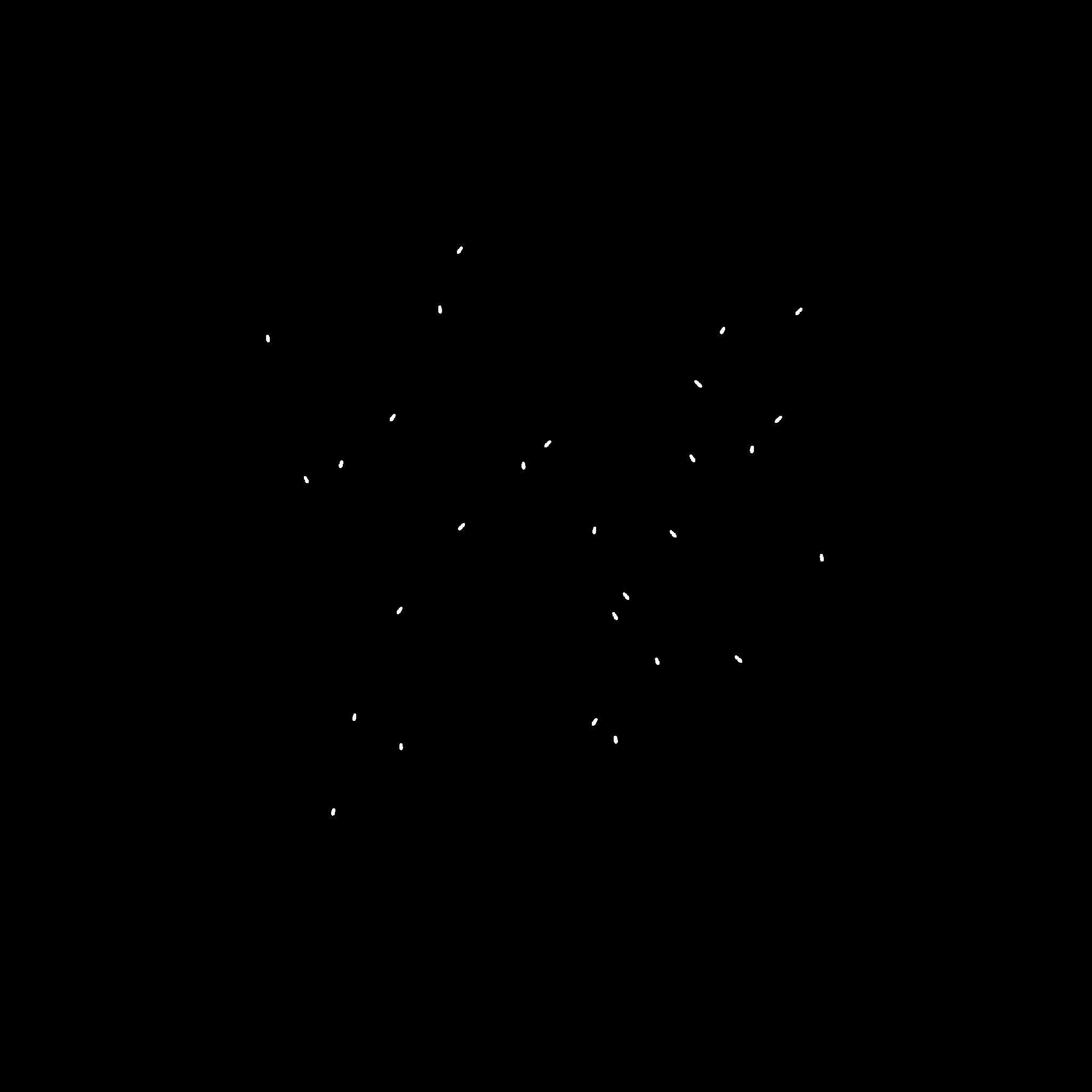

Supplement: S1 File — (ZIP) [file pone.0132101.s003.zip › ORsrc/nonortho/simu028/camx/imx067.jpg]

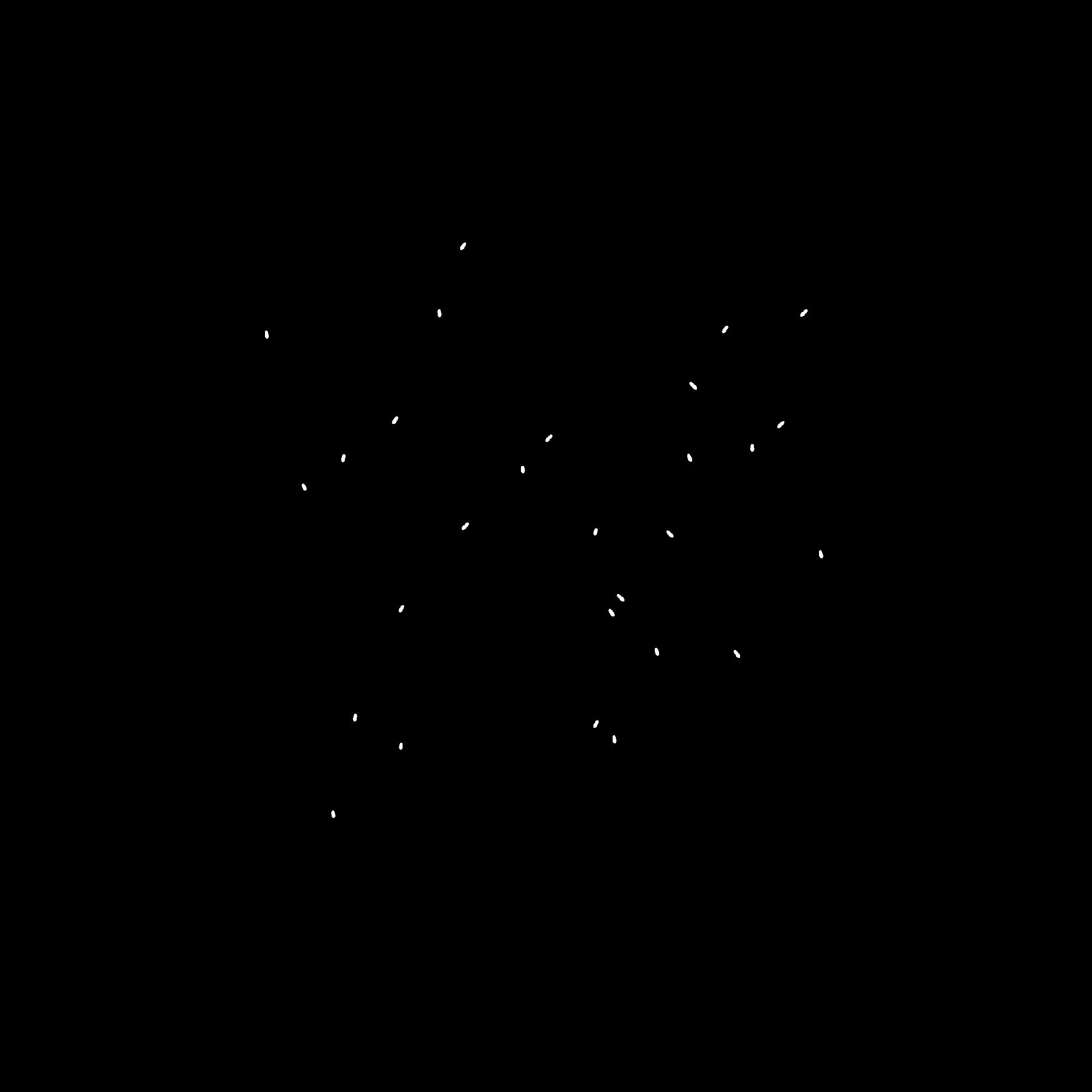

Supplement: S1 File — (ZIP) [file pone.0132101.s003.zip › ORsrc/nonortho/simu028/camx/imx068.jpg]

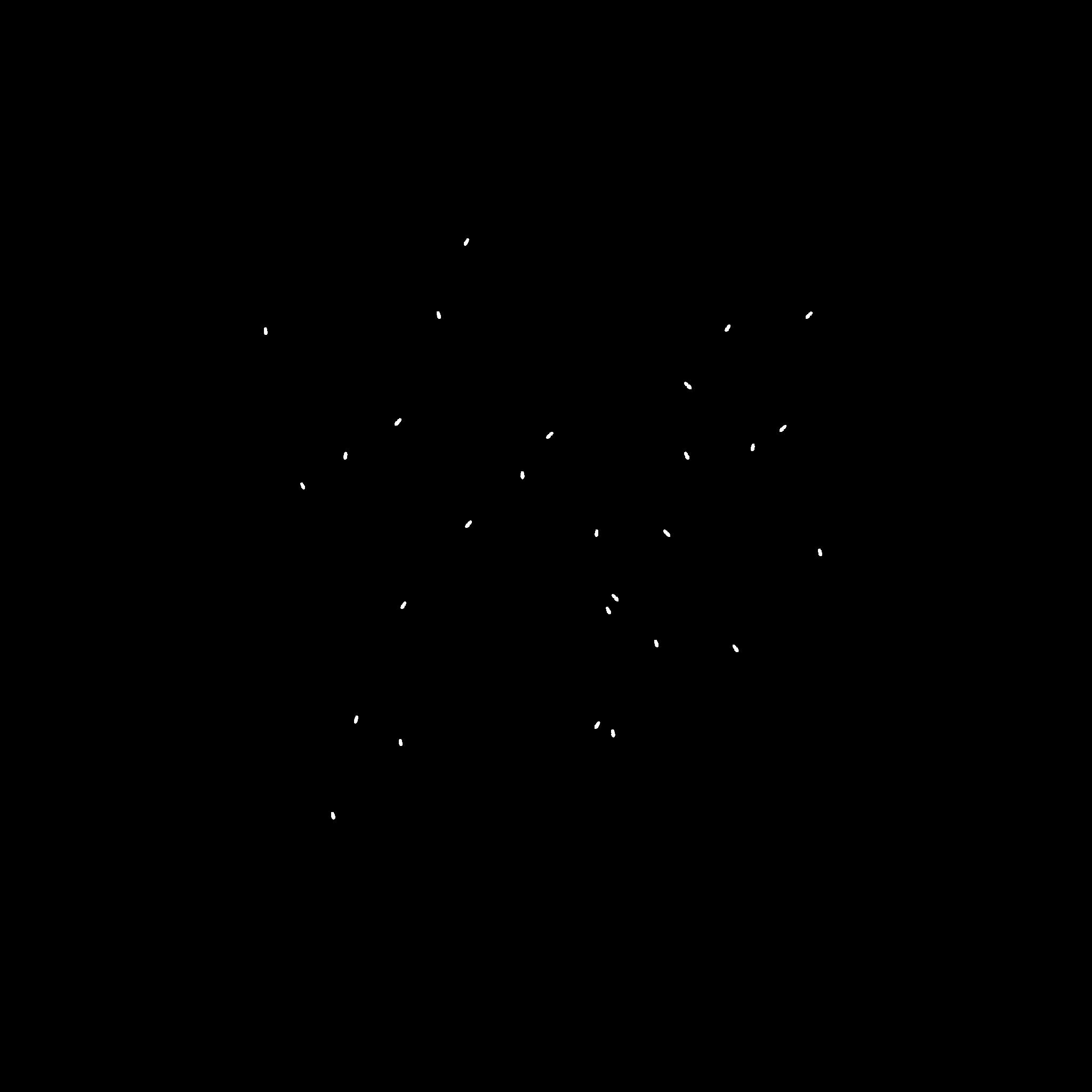

Supplement: S1 File — (ZIP) [file pone.0132101.s003.zip › ORsrc/nonortho/simu028/camx/imx069.jpg]

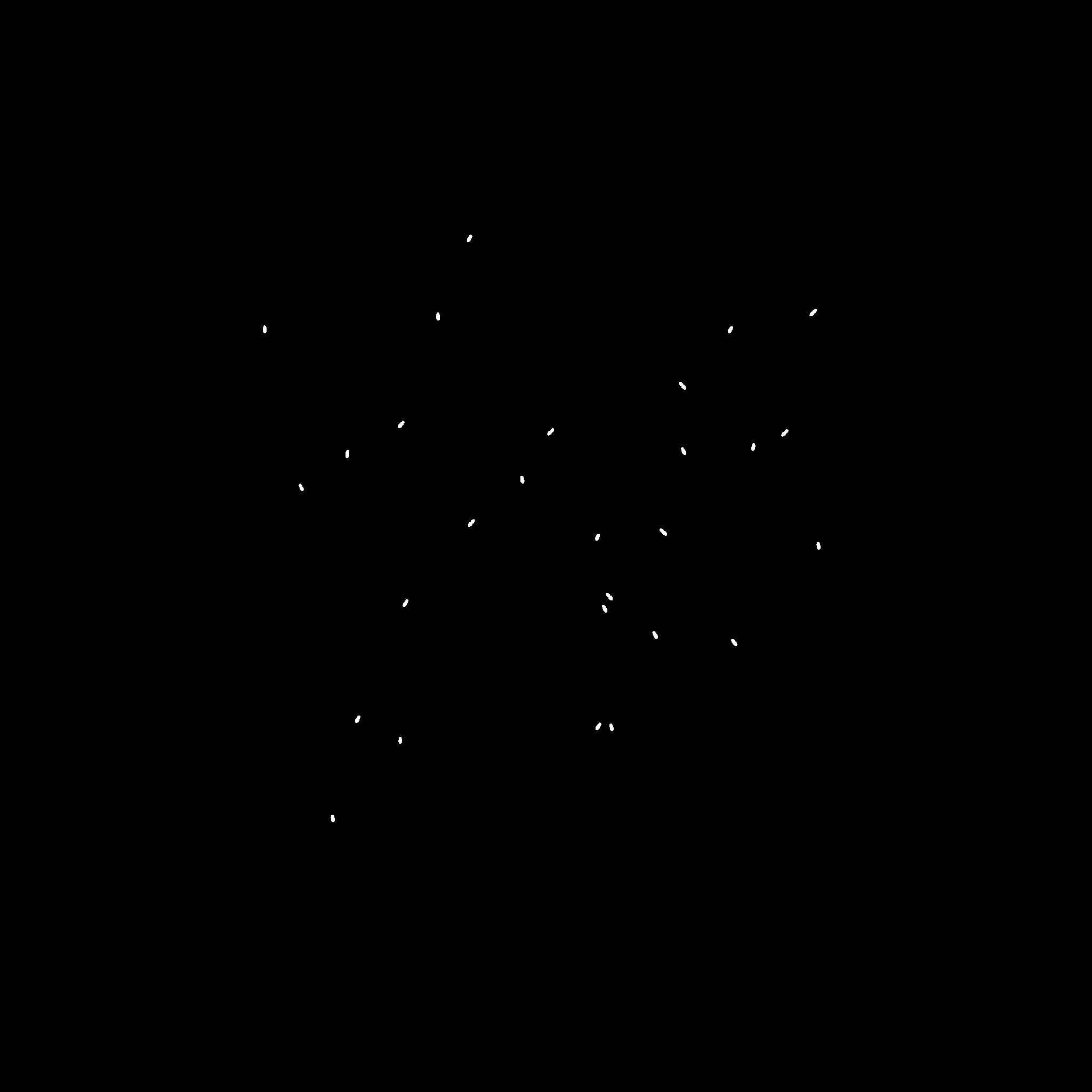

Supplement: S1 File — (ZIP) [file pone.0132101.s003.zip › ORsrc/nonortho/simu028/camx/imx070.jpg]

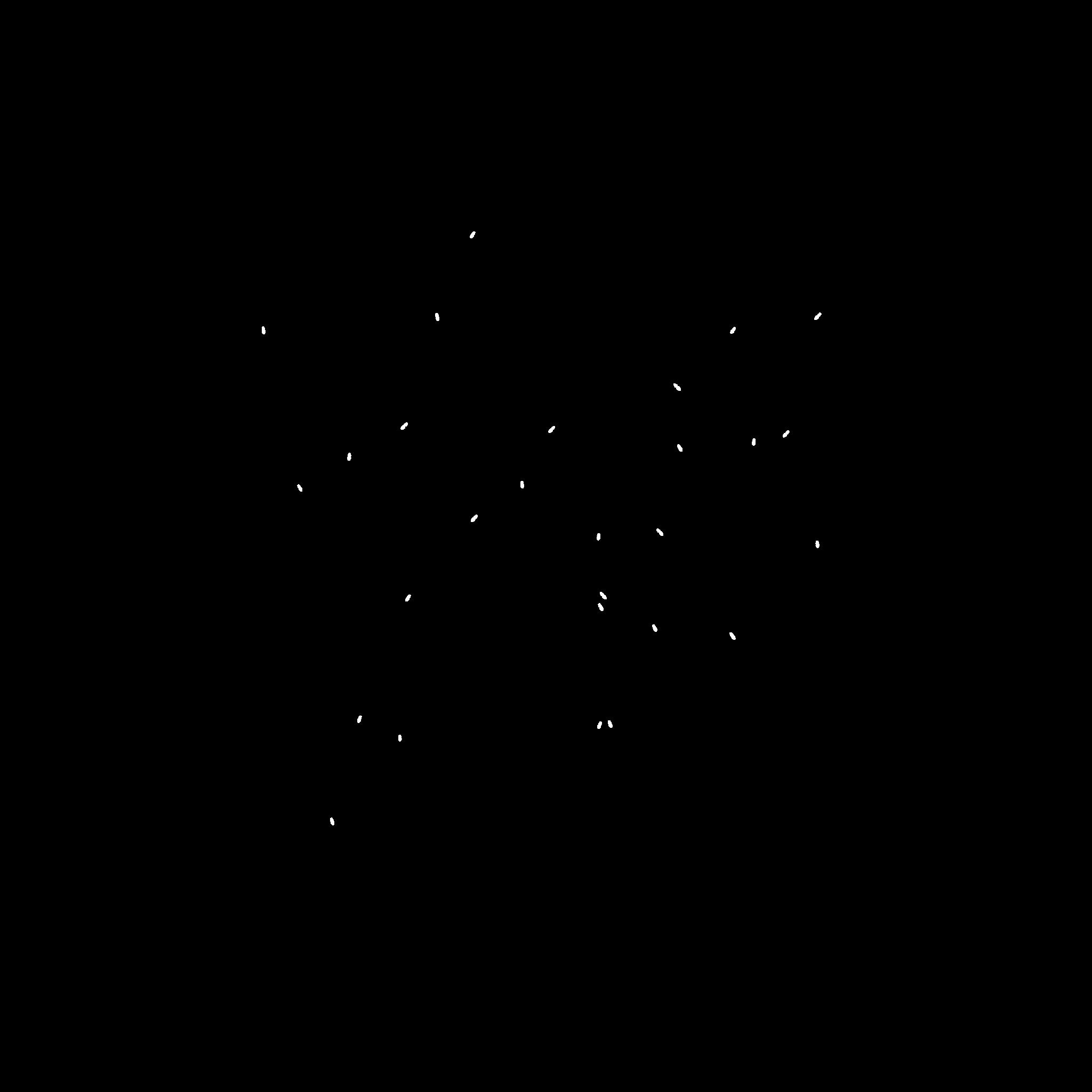

Supplement: S1 File — (ZIP) [file pone.0132101.s003.zip › ORsrc/nonortho/simu028/camx/imx071.jpg]

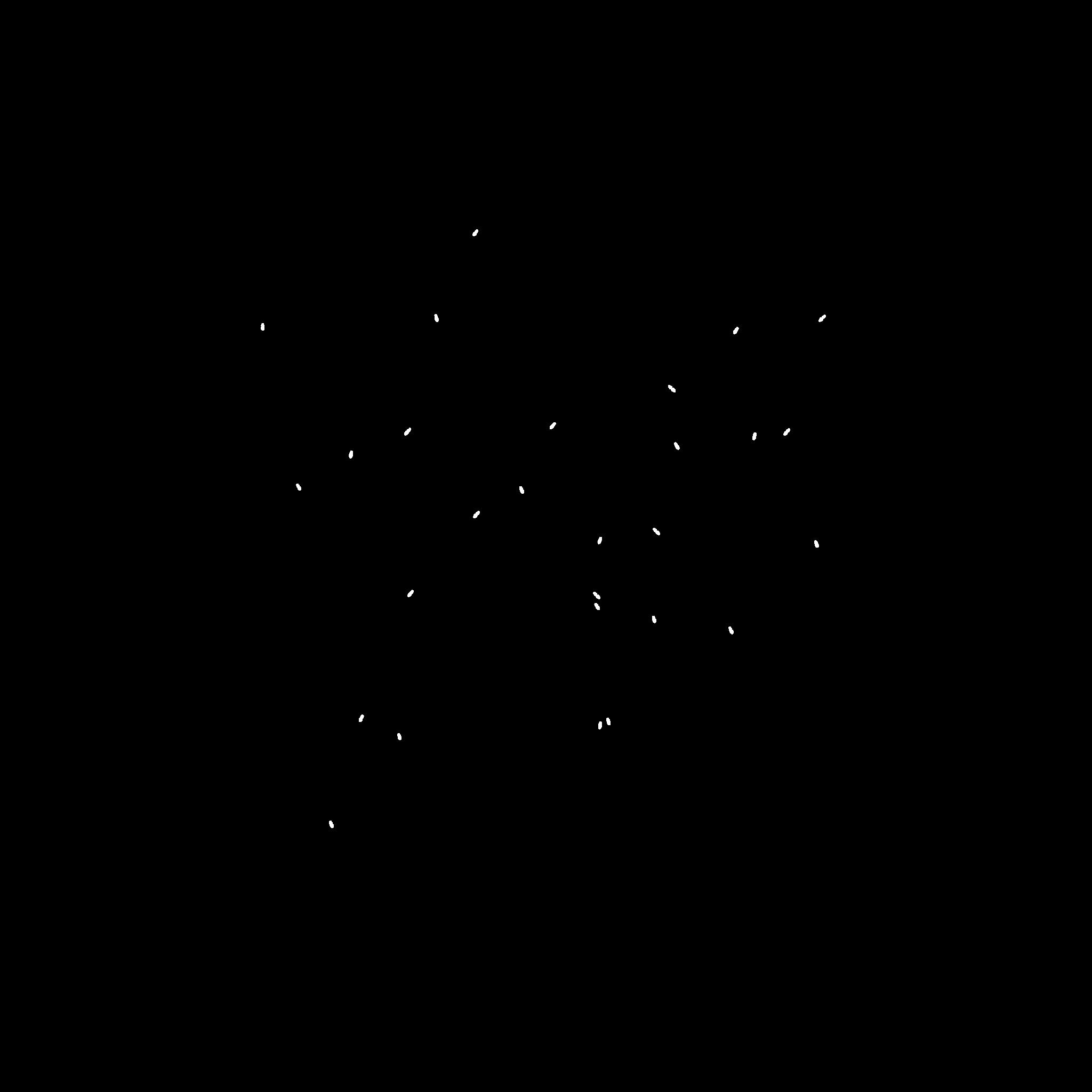

Supplement: S1 File — (ZIP) [file pone.0132101.s003.zip › ORsrc/nonortho/simu028/camx/imx072.jpg]

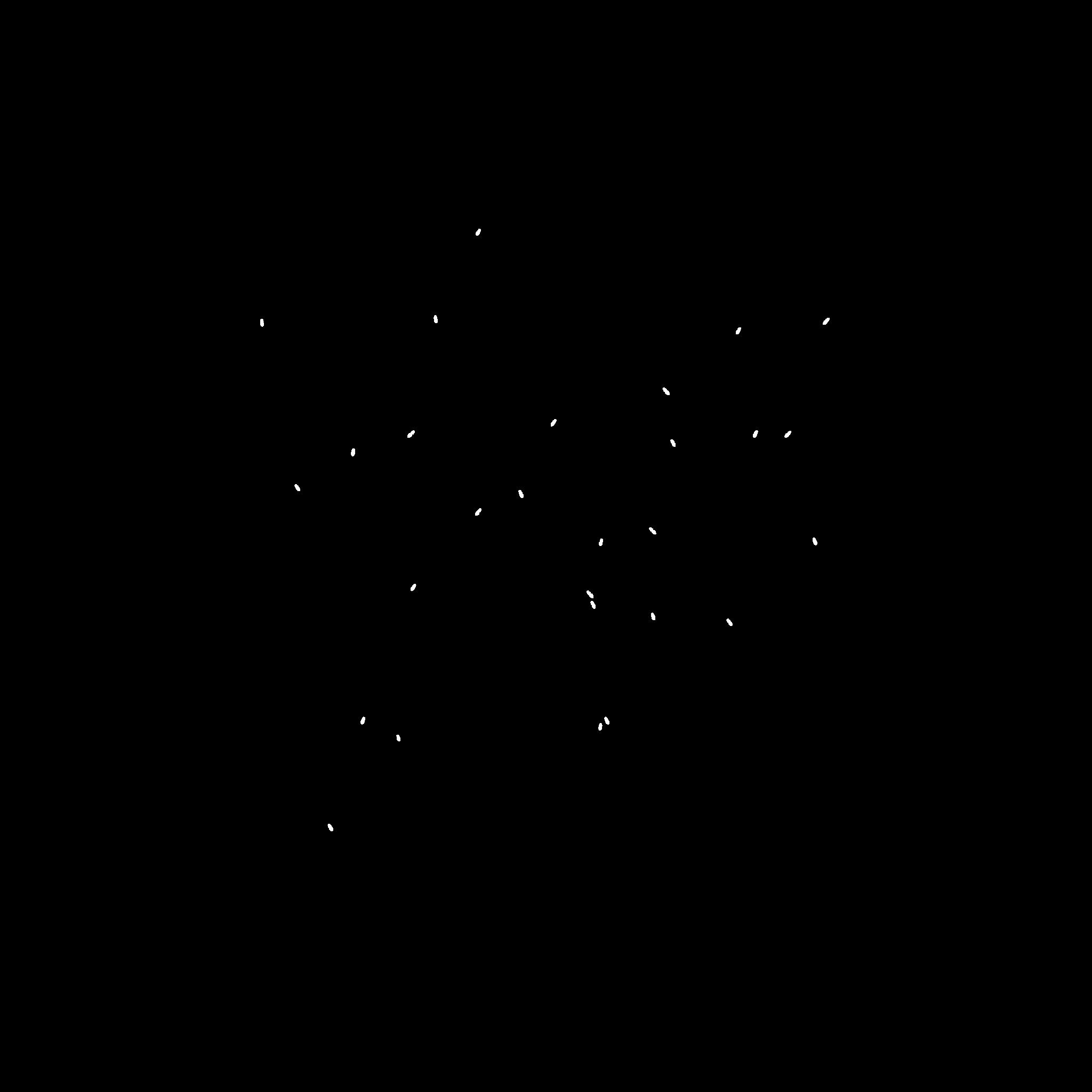

Supplement: S1 File — (ZIP) [file pone.0132101.s003.zip › ORsrc/nonortho/simu028/camx/imx073.jpg]

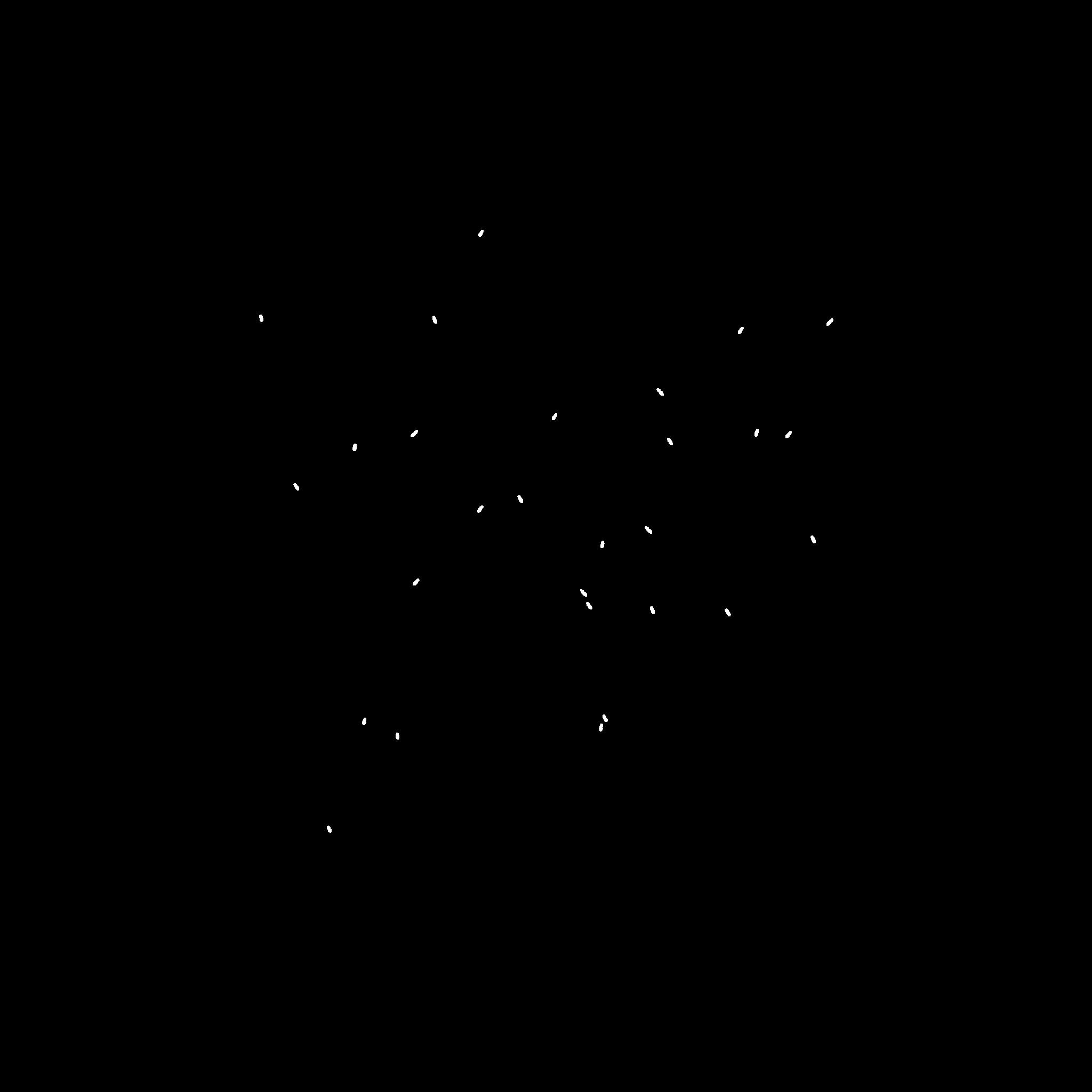

Supplement: S1 File — (ZIP) [file pone.0132101.s003.zip › ORsrc/nonortho/simu028/camx/imx074.jpg]

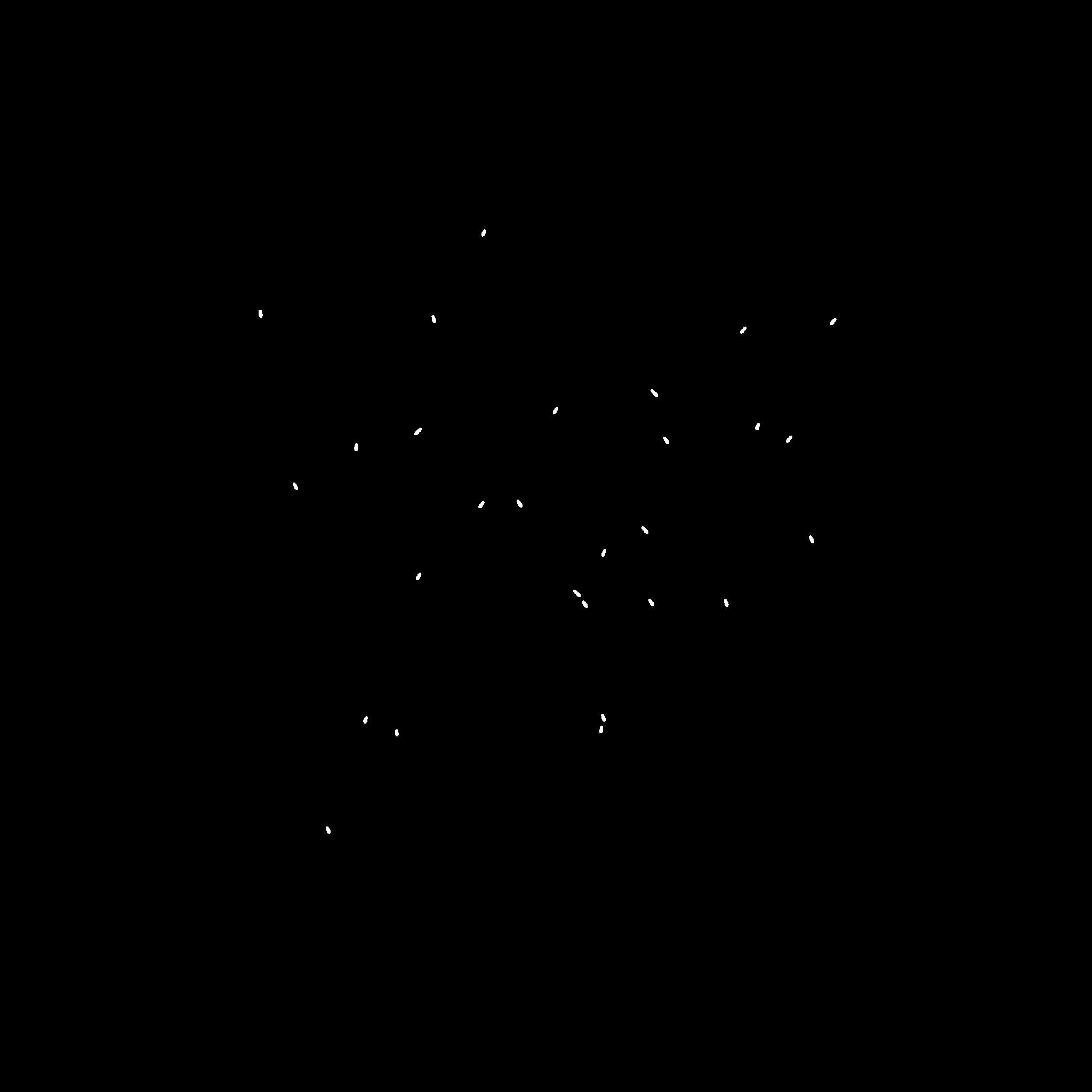

Supplement: S1 File — (ZIP) [file pone.0132101.s003.zip › ORsrc/nonortho/simu028/camx/imx075.jpg]

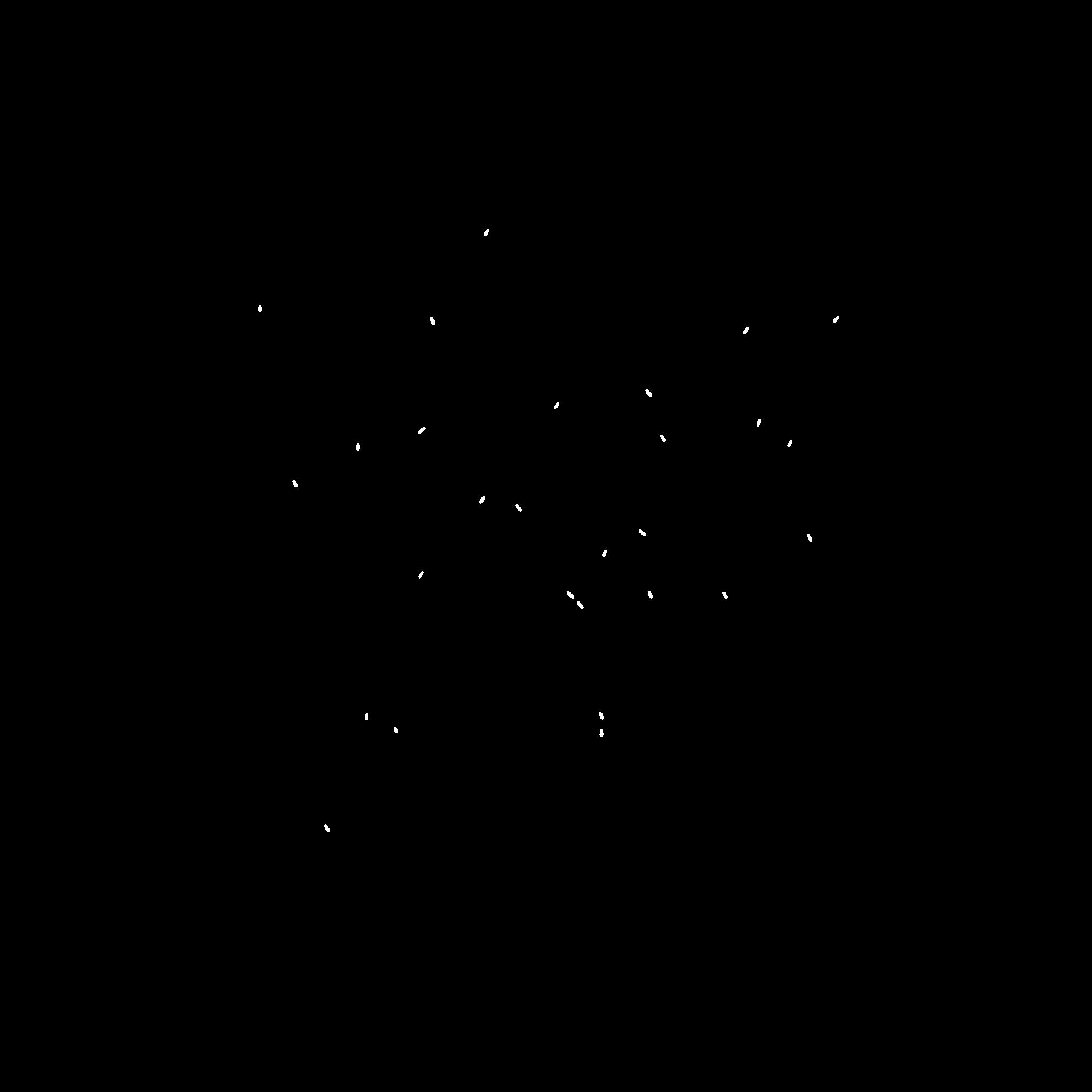

Supplement: S1 File — (ZIP) [file pone.0132101.s003.zip › ORsrc/nonortho/simu028/camx/imx076.jpg]

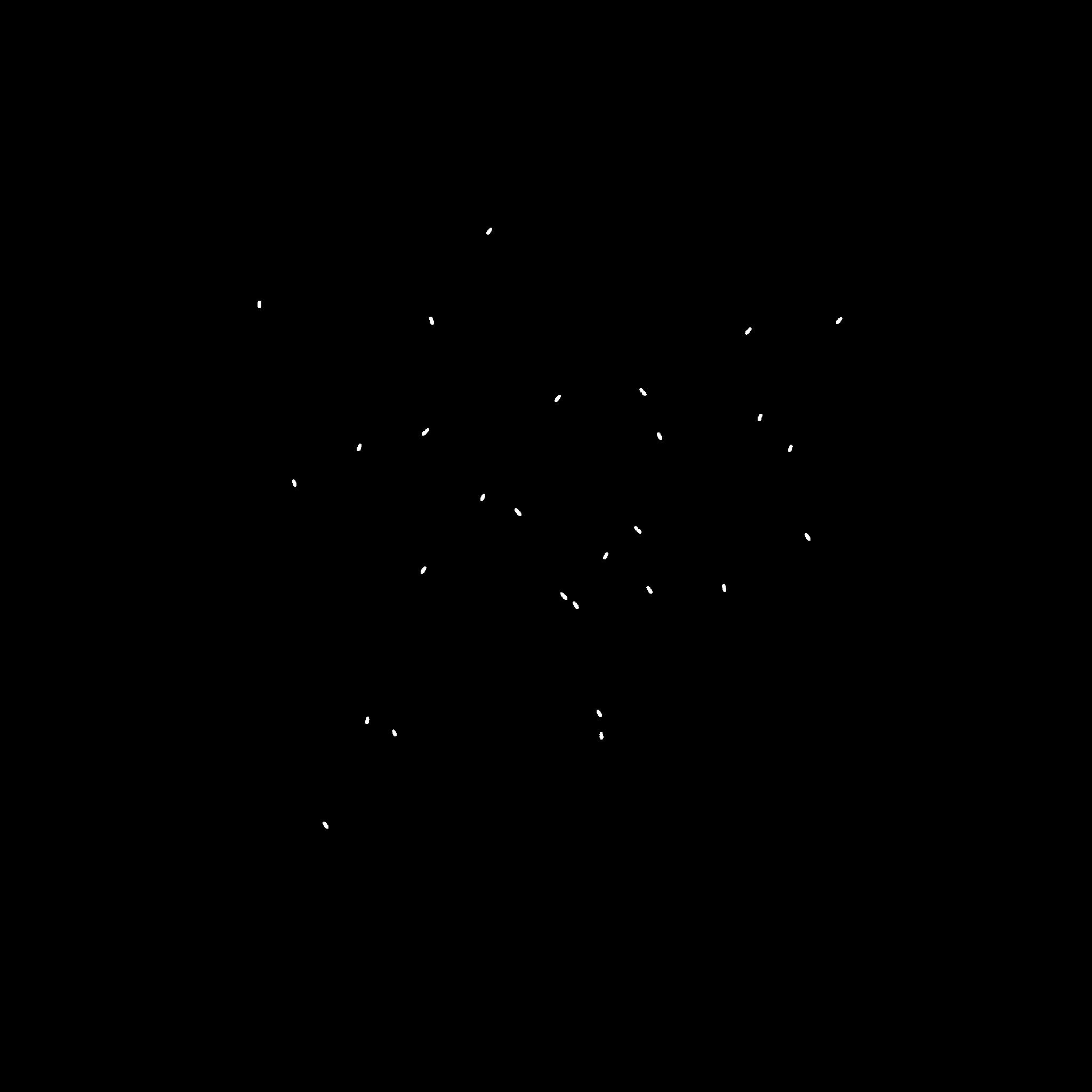

Supplement: S1 File — (ZIP) [file pone.0132101.s003.zip › ORsrc/nonortho/simu028/camx/imx077.jpg]

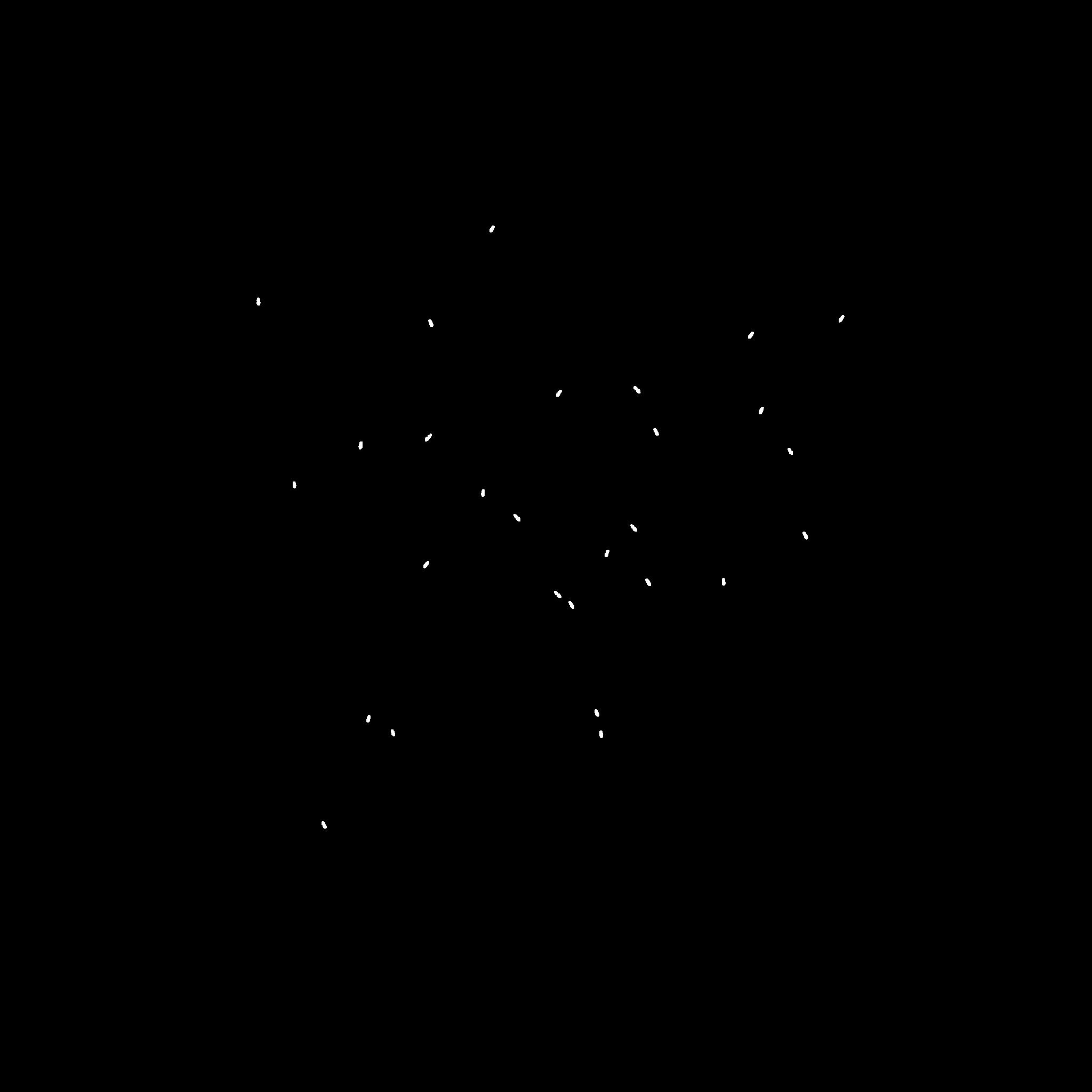

Supplement: S1 File — (ZIP) [file pone.0132101.s003.zip › ORsrc/nonortho/simu028/camx/imx078.jpg]

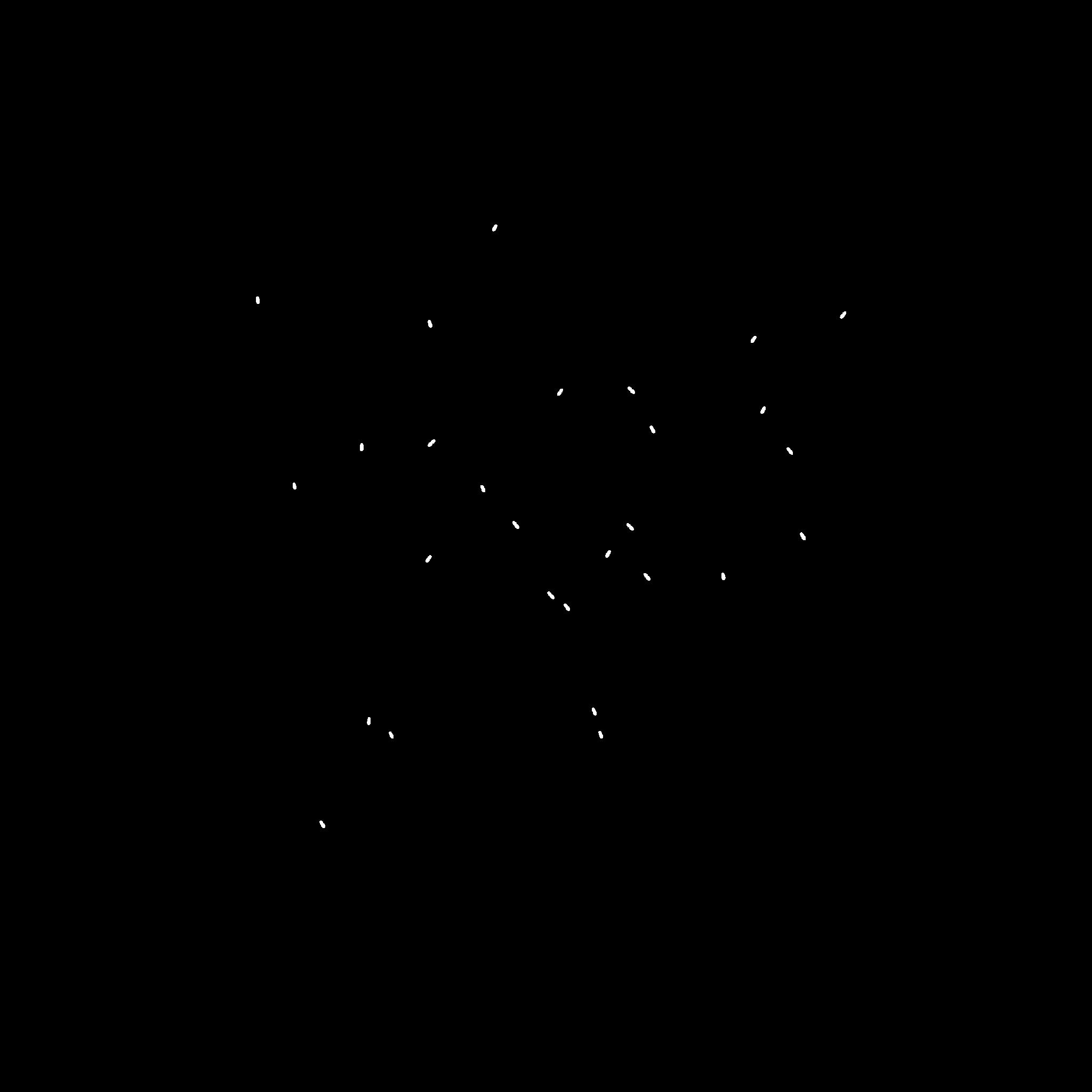

Supplement: S1 File — (ZIP) [file pone.0132101.s003.zip › ORsrc/nonortho/simu028/camx/imx079.jpg]

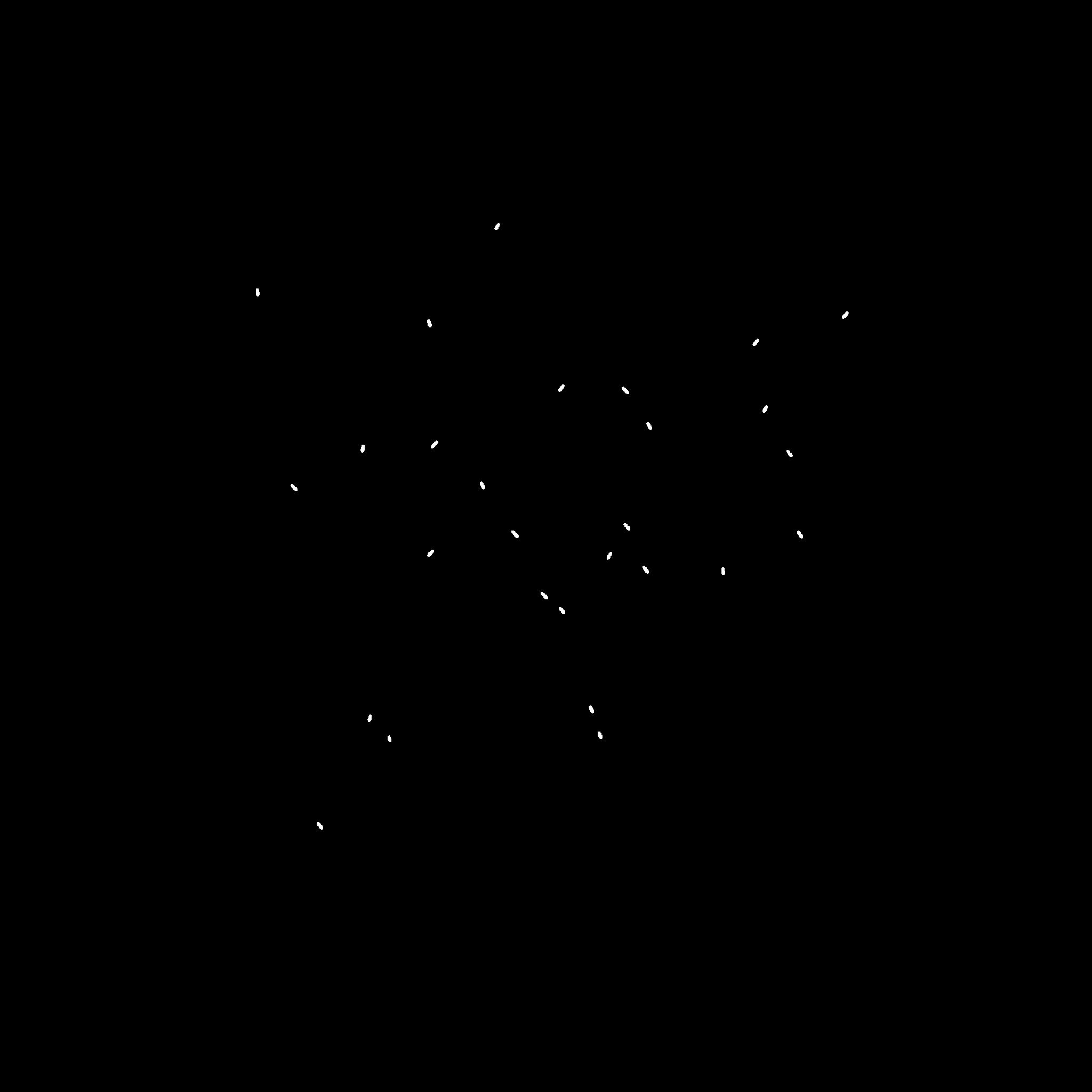

Supplement: S1 File — (ZIP) [file pone.0132101.s003.zip › ORsrc/nonortho/simu028/camx/imx080.jpg]

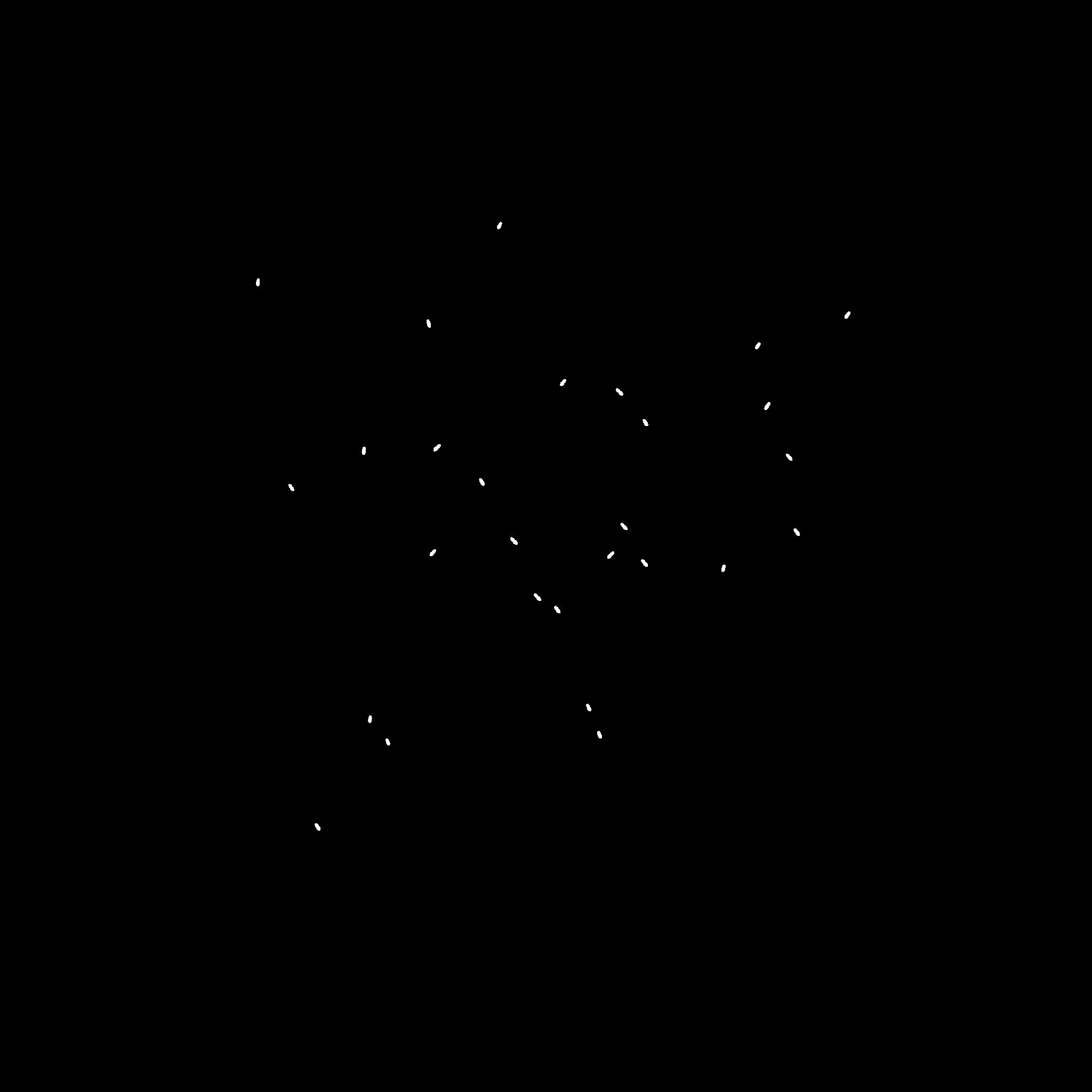

Supplement: S1 File — (ZIP) [file pone.0132101.s003.zip › ORsrc/nonortho/simu028/camx/imx081.jpg]

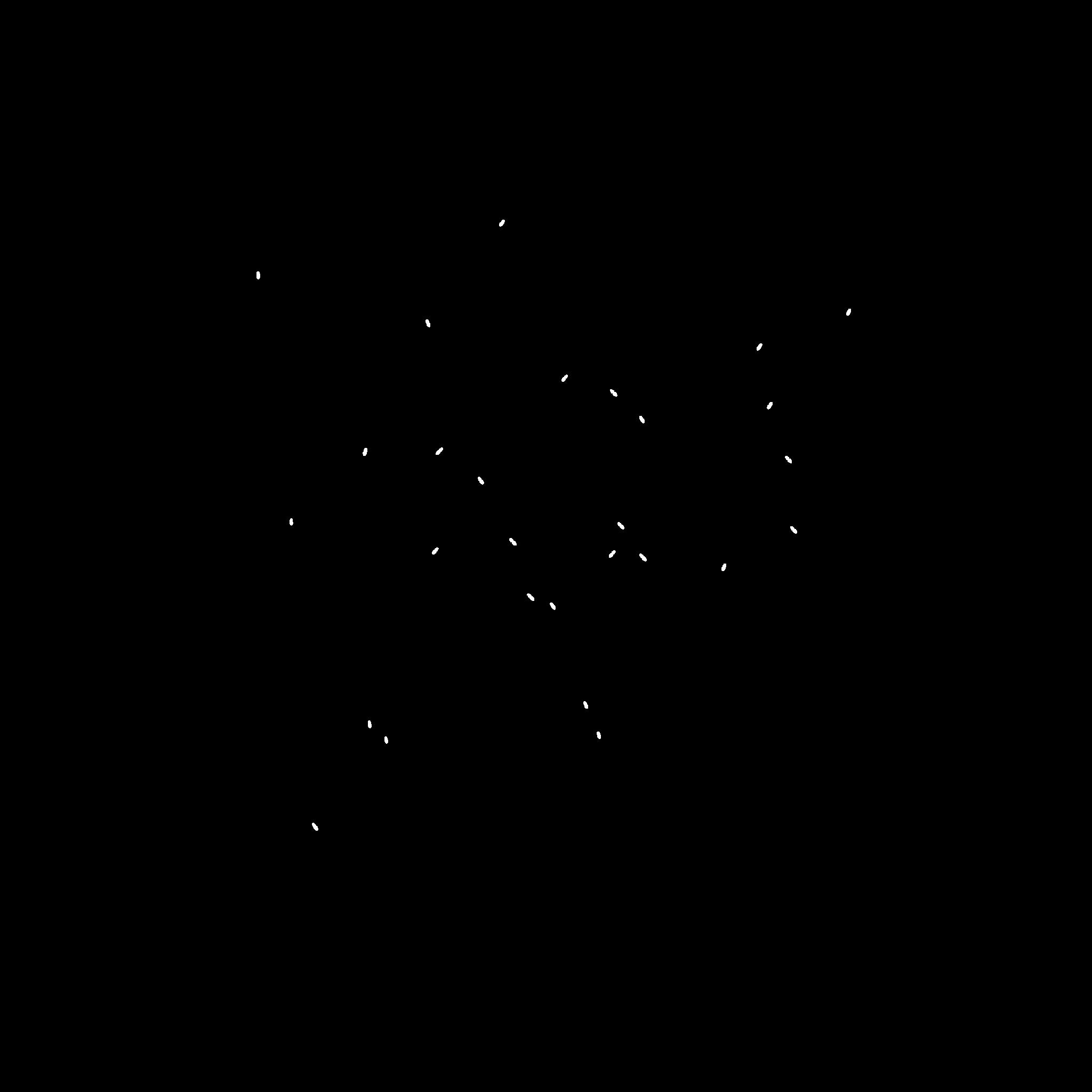

Supplement: S1 File — (ZIP) [file pone.0132101.s003.zip › ORsrc/nonortho/simu028/camx/imx082.jpg]

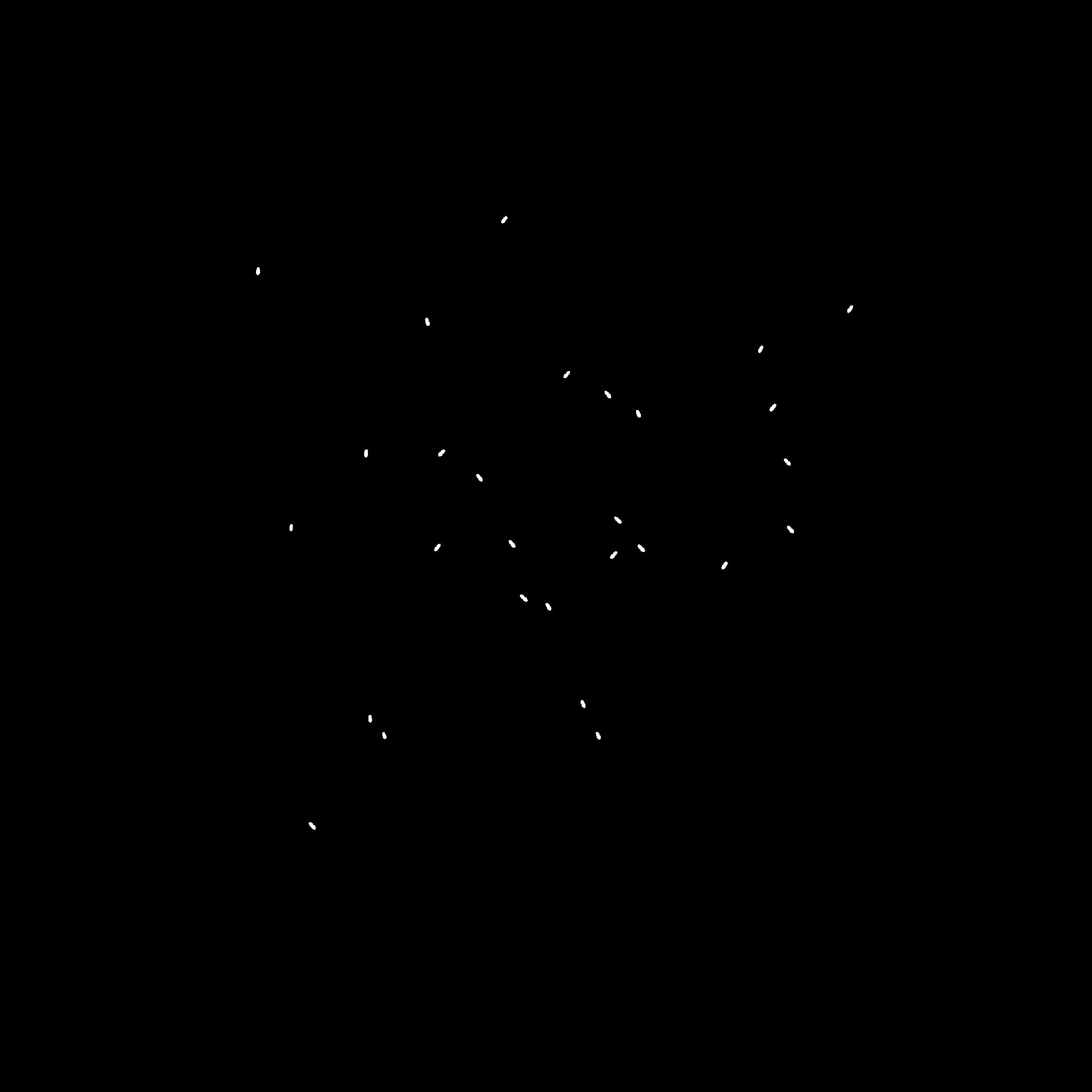

Supplement: S1 File — (ZIP) [file pone.0132101.s003.zip › ORsrc/nonortho/simu028/camx/imx083.jpg]

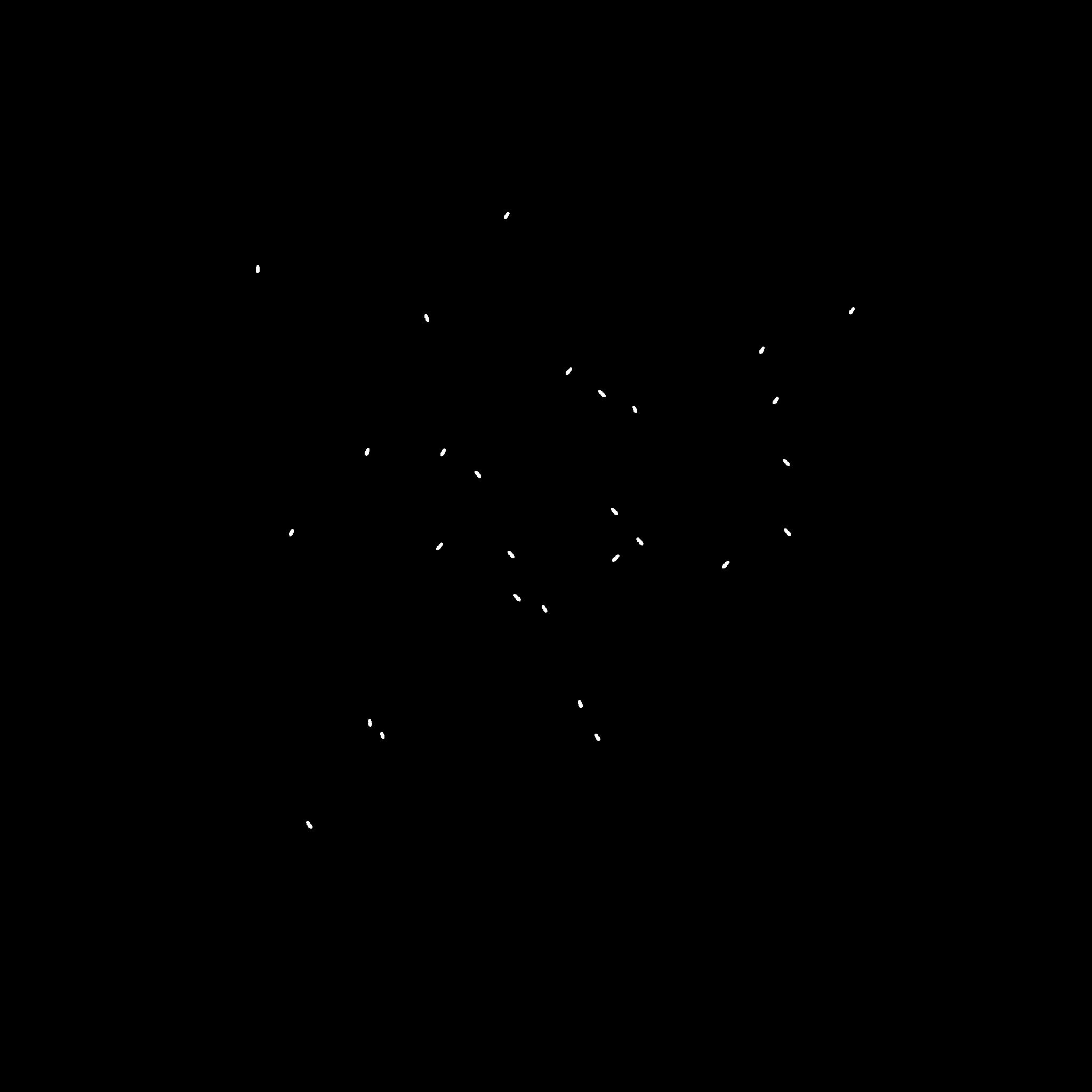

Supplement: S1 File — (ZIP) [file pone.0132101.s003.zip › ORsrc/nonortho/simu028/camx/imx084.jpg]

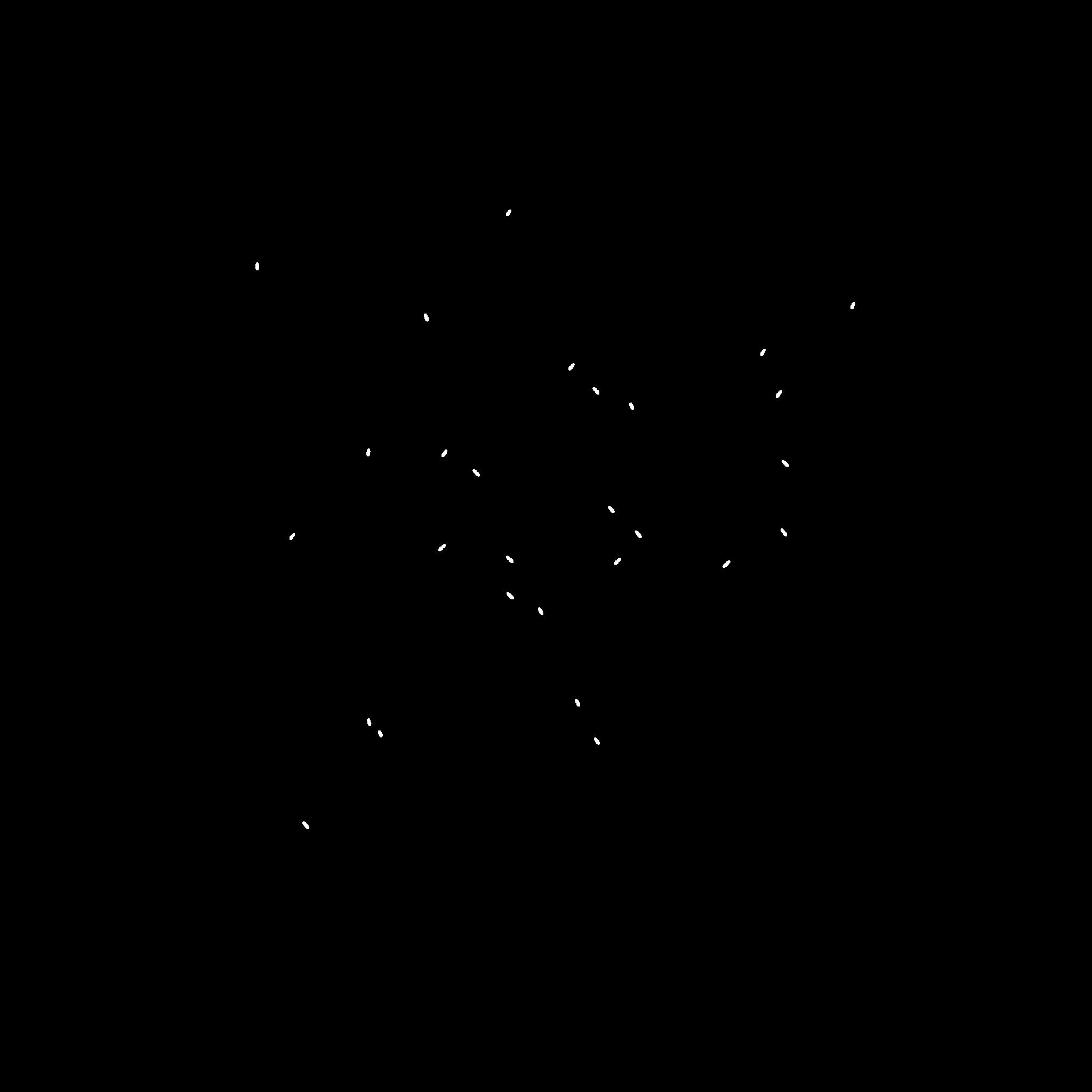

Supplement: S1 File — (ZIP) [file pone.0132101.s003.zip › ORsrc/nonortho/simu028/camx/imx085.jpg]

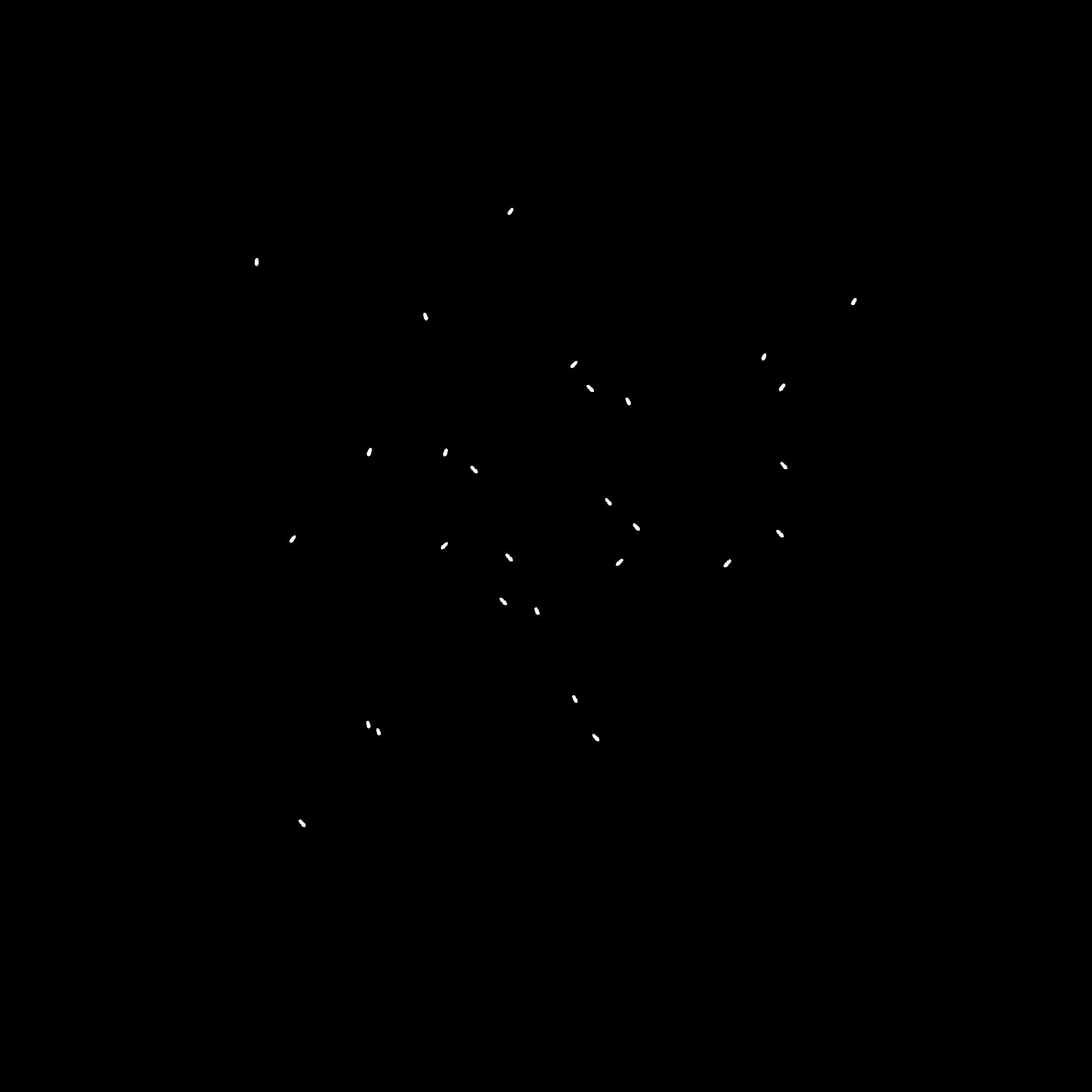

Supplement: S1 File — (ZIP) [file pone.0132101.s003.zip › ORsrc/nonortho/simu028/camx/imx086.jpg]

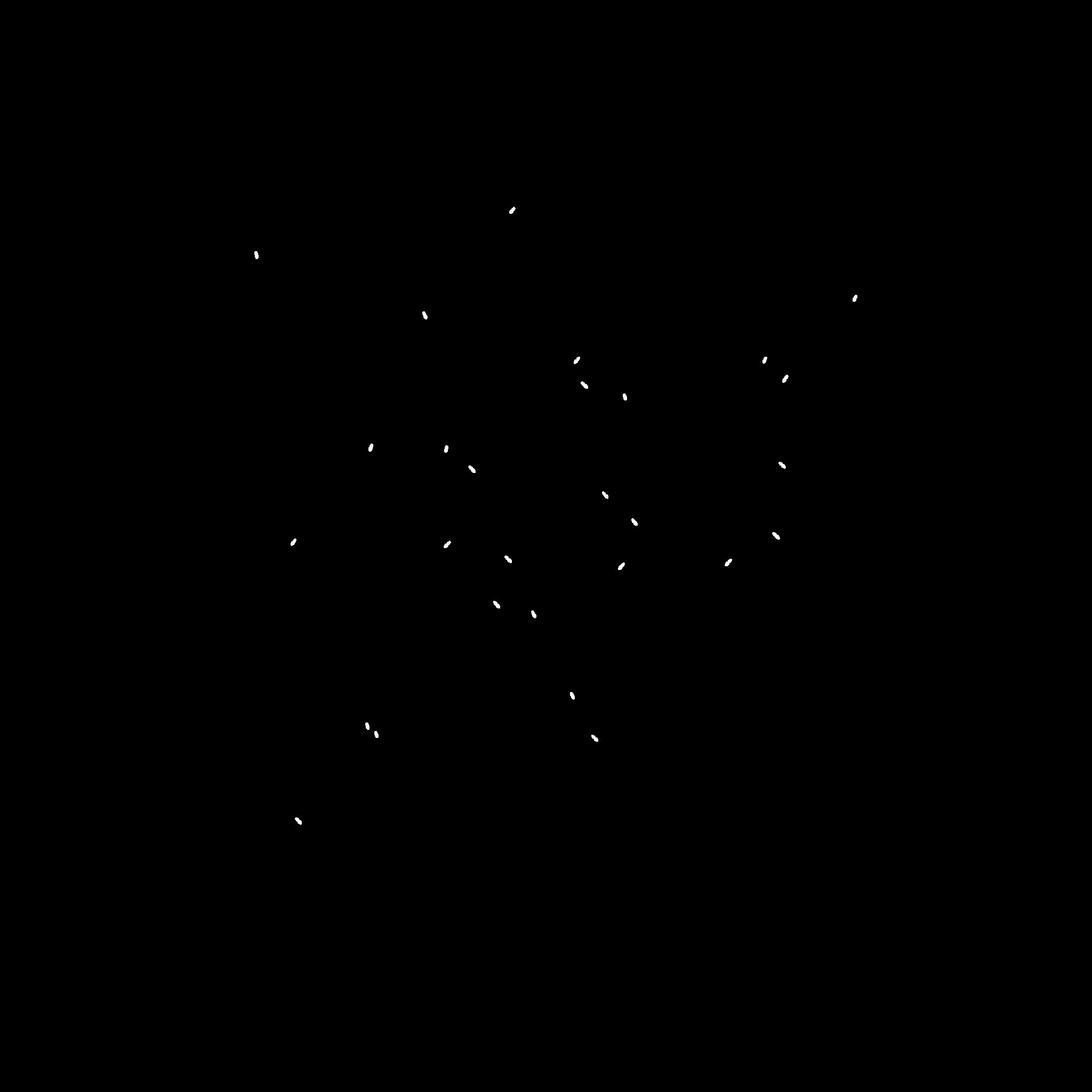

Supplement: S1 File — (ZIP) [file pone.0132101.s003.zip › ORsrc/nonortho/simu028/camx/imx087.jpg]

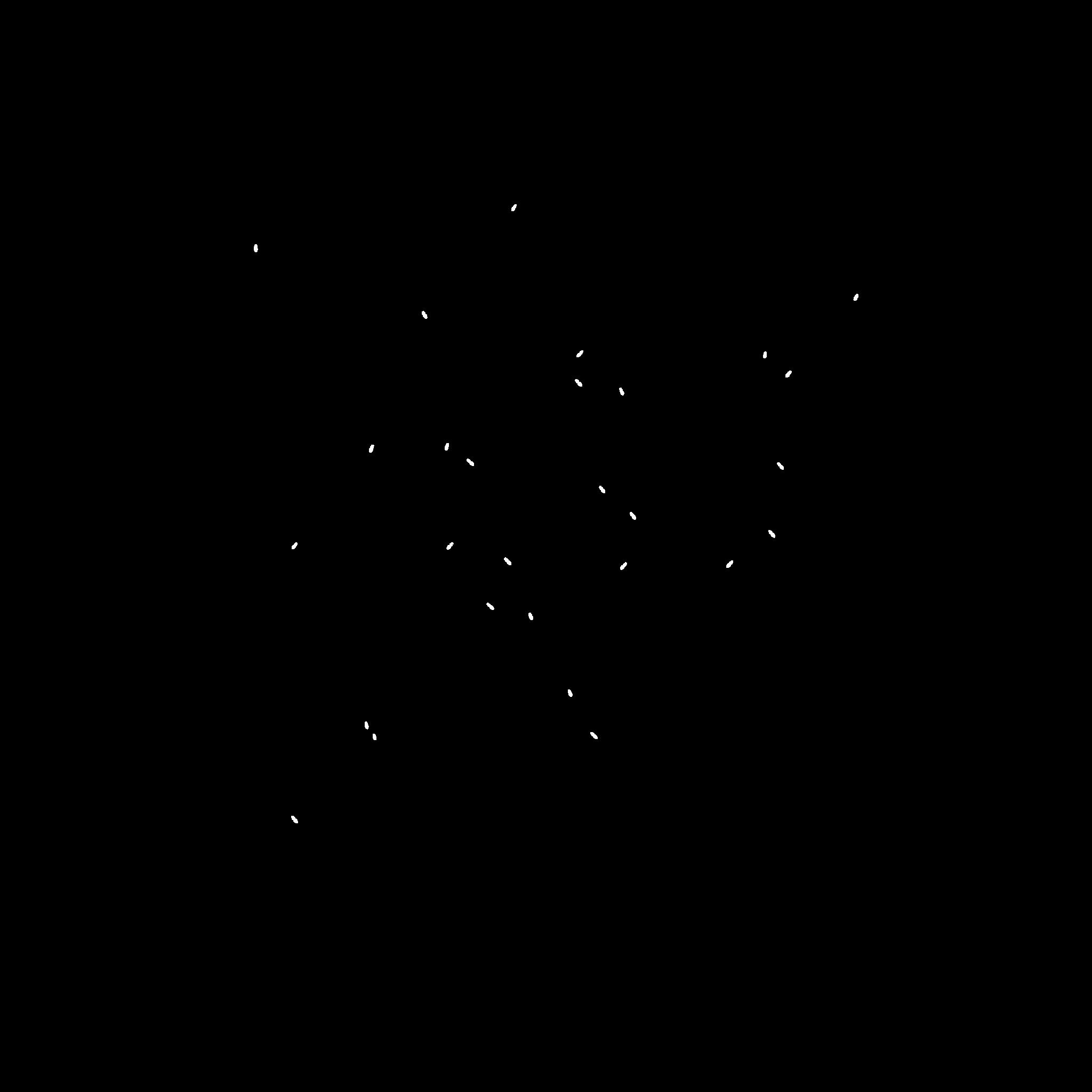

Supplement: S1 File — (ZIP) [file pone.0132101.s003.zip › ORsrc/nonortho/simu028/camx/imx088.jpg]

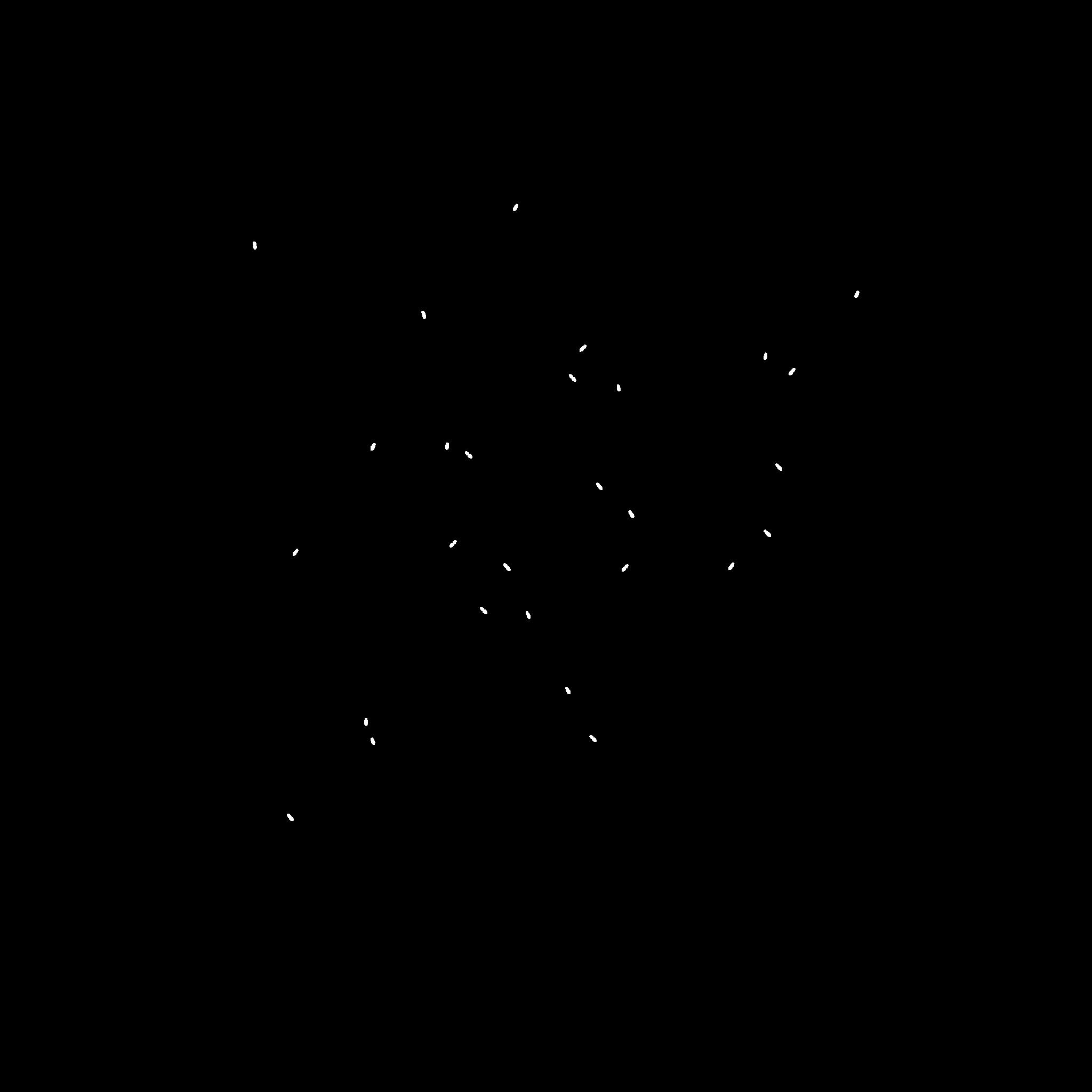

Supplement: S1 File — (ZIP) [file pone.0132101.s003.zip › ORsrc/nonortho/simu028/camx/imx089.jpg]

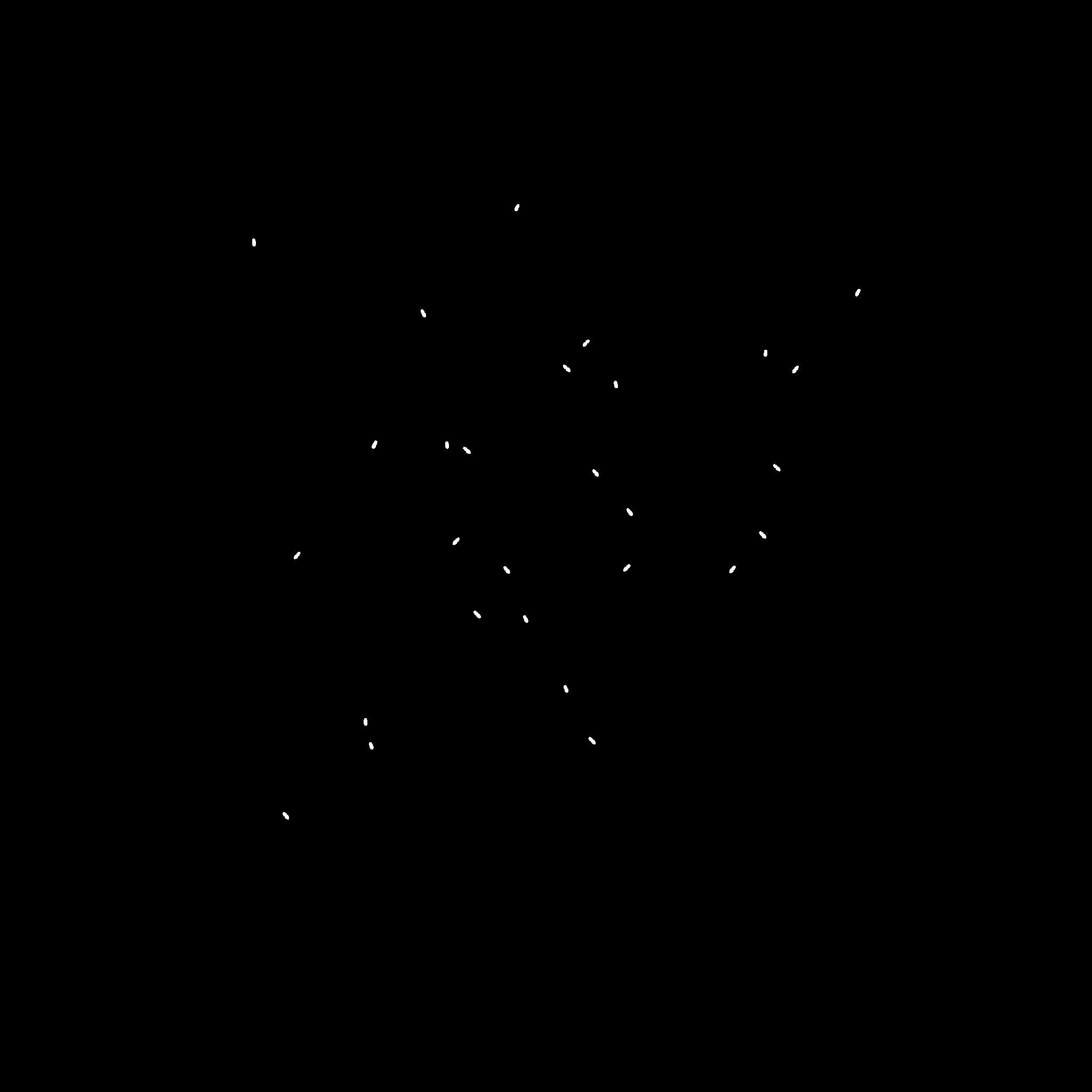

Supplement: S1 File — (ZIP) [file pone.0132101.s003.zip › ORsrc/nonortho/simu028/camx/imx090.jpg]

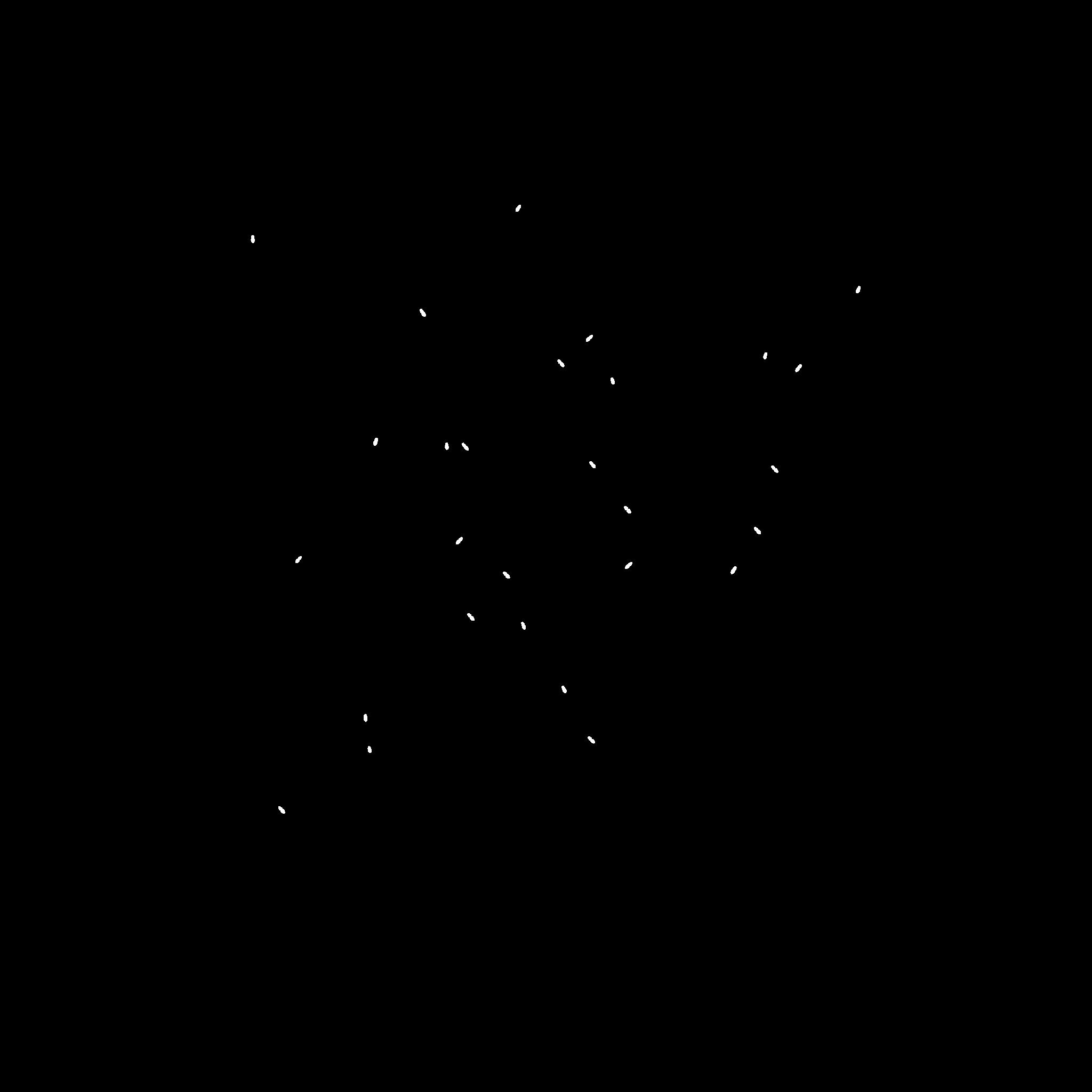

Supplement: S1 File — (ZIP) [file pone.0132101.s003.zip › ORsrc/nonortho/simu028/camx/imx091.jpg]

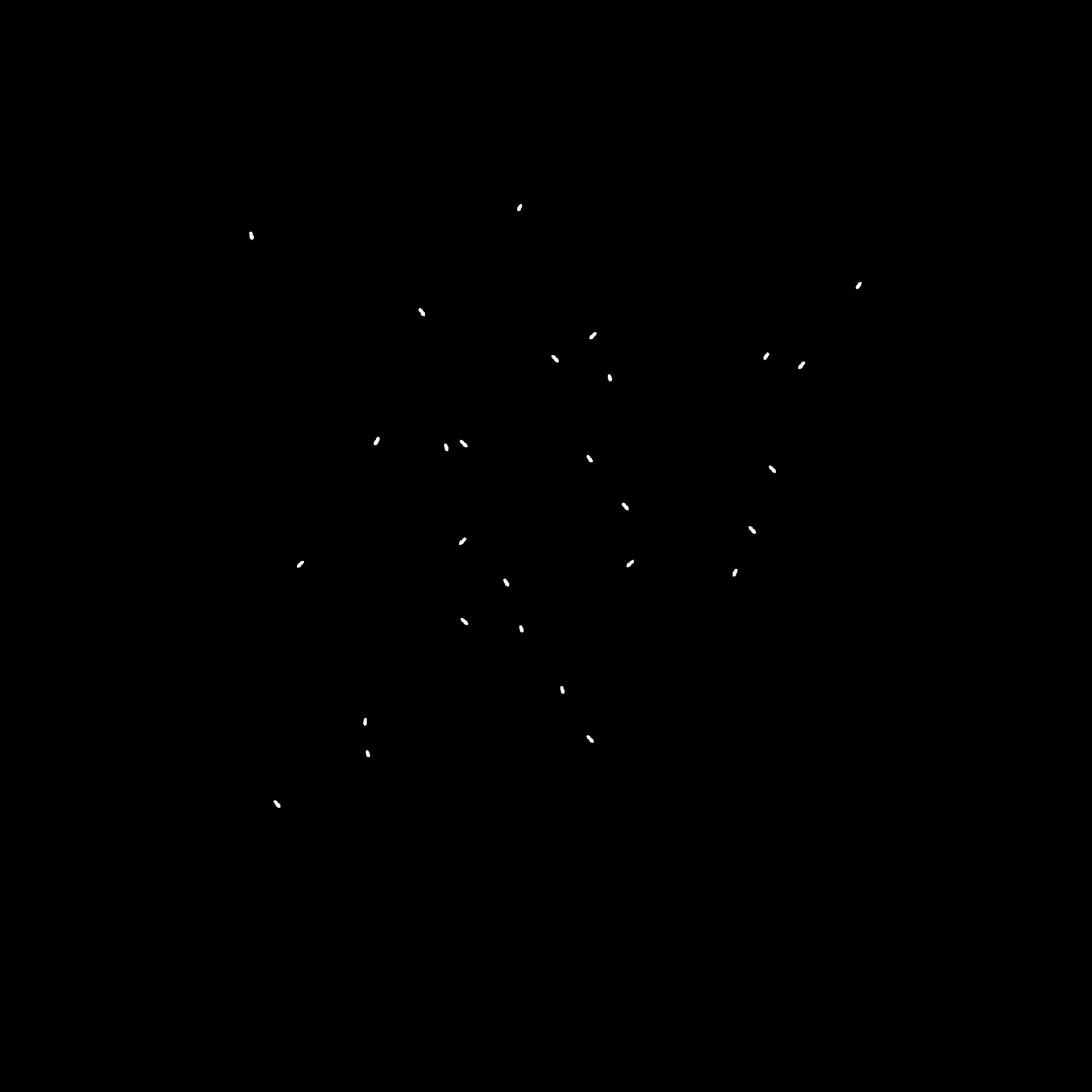

Supplement: S1 File — (ZIP) [file pone.0132101.s003.zip › ORsrc/nonortho/simu028/camx/imx092.jpg]

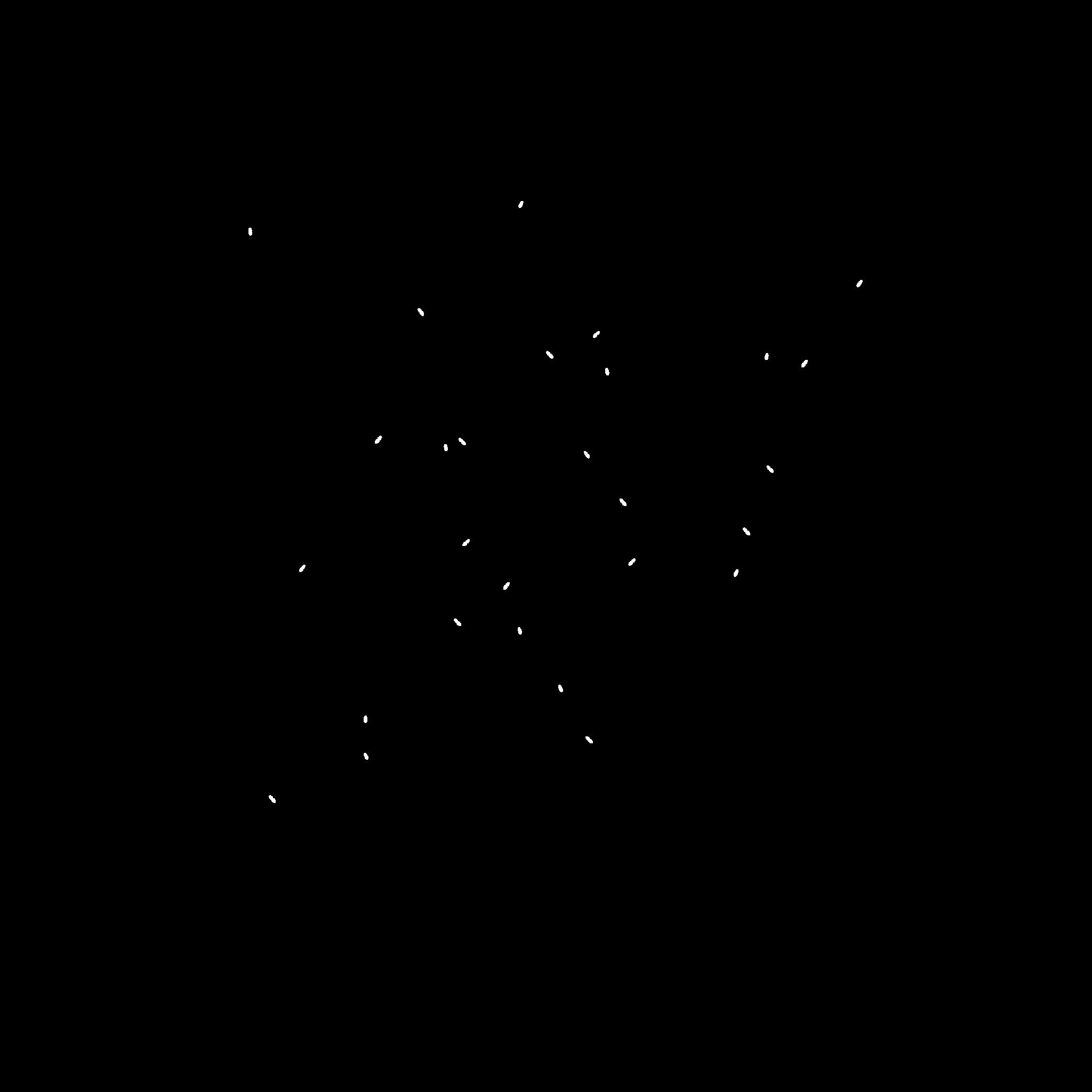

Supplement: S1 File — (ZIP) [file pone.0132101.s003.zip › ORsrc/nonortho/simu028/camx/imx093.jpg]

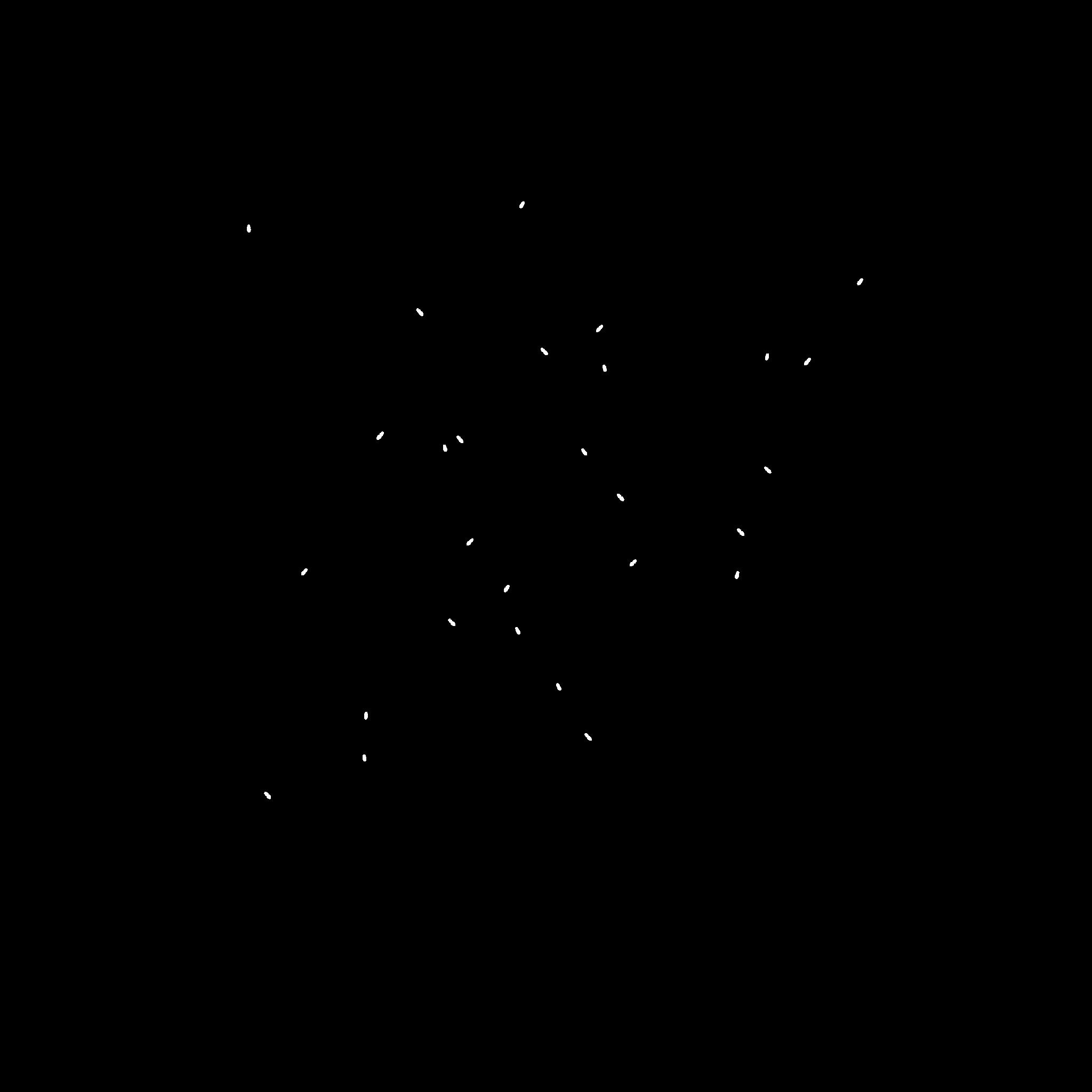

Supplement: S1 File — (ZIP) [file pone.0132101.s003.zip › ORsrc/nonortho/simu028/camx/imx094.jpg]

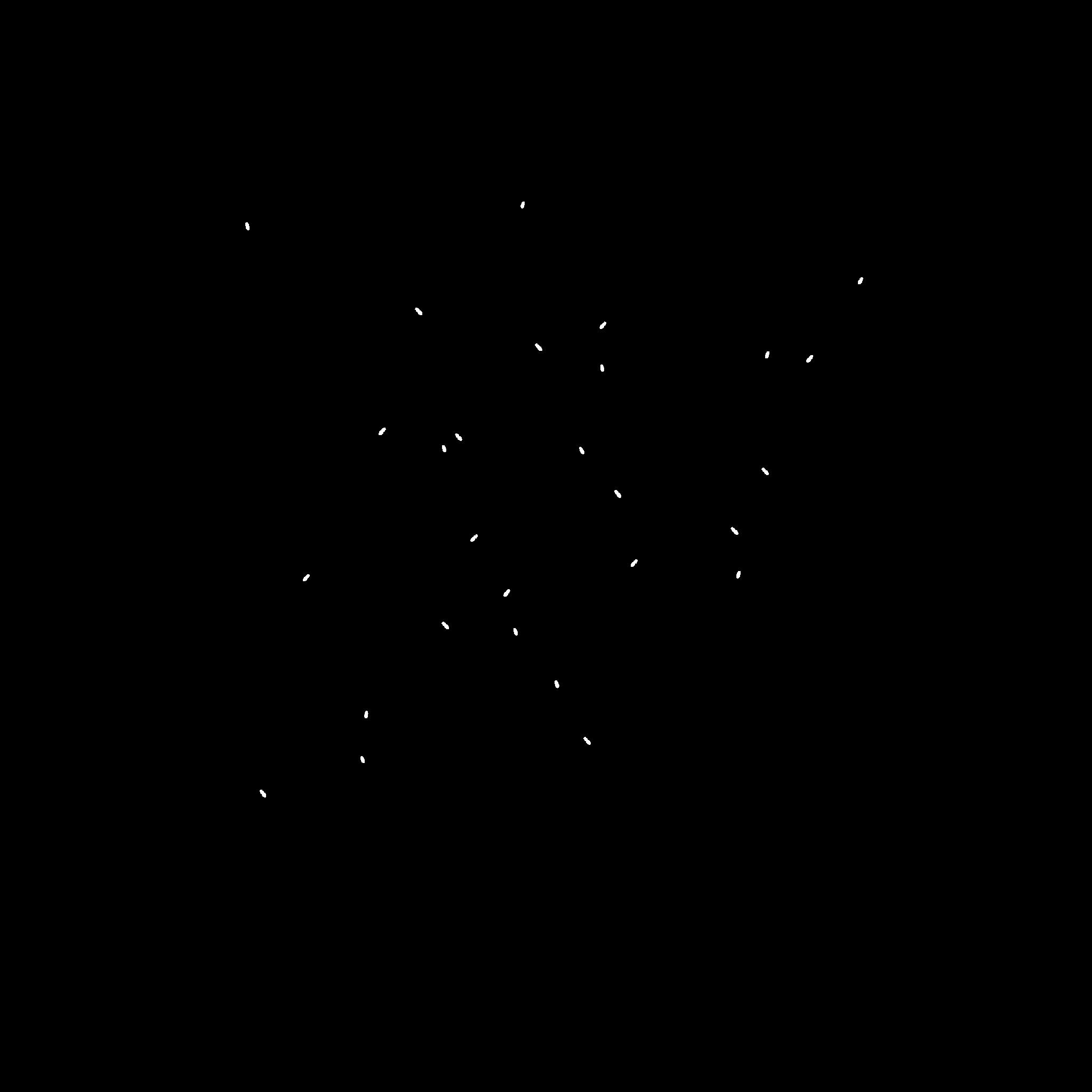

Supplement: S1 File — (ZIP) [file pone.0132101.s003.zip › ORsrc/nonortho/simu028/camx/imx095.jpg]

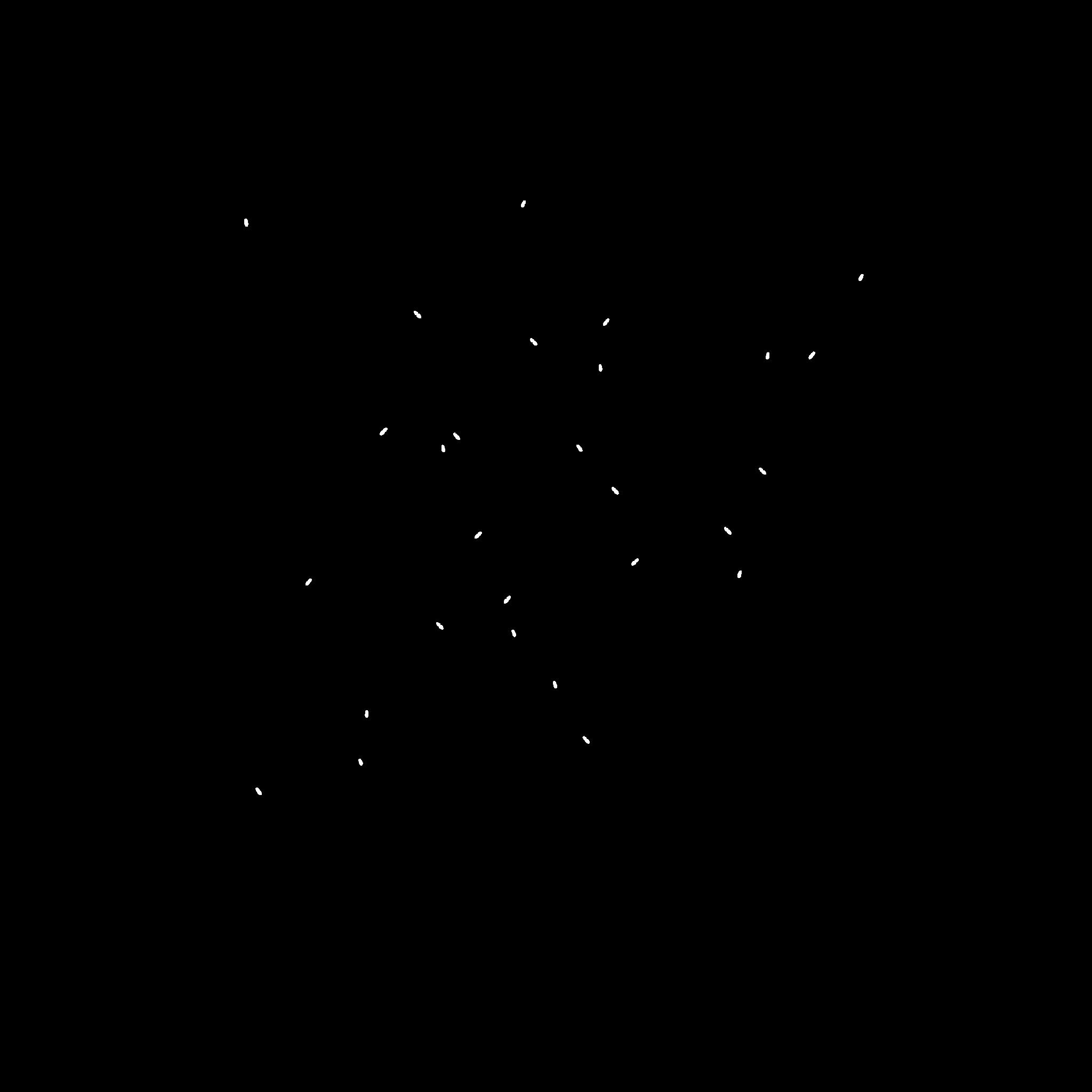

Supplement: S1 File — (ZIP) [file pone.0132101.s003.zip › ORsrc/nonortho/simu028/camx/imx096.jpg]

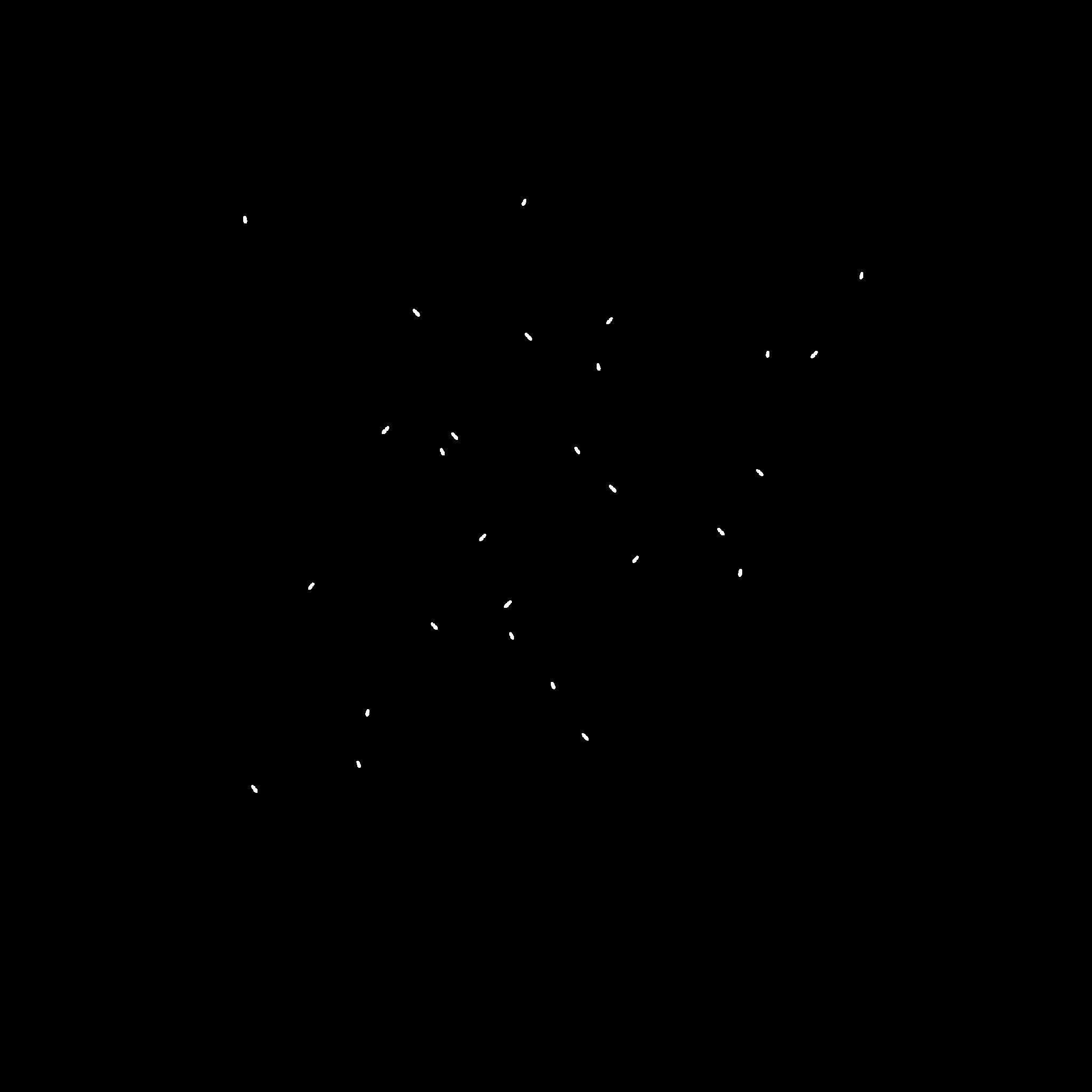

Supplement: S1 File — (ZIP) [file pone.0132101.s003.zip › ORsrc/nonortho/simu028/camx/imx097.jpg]

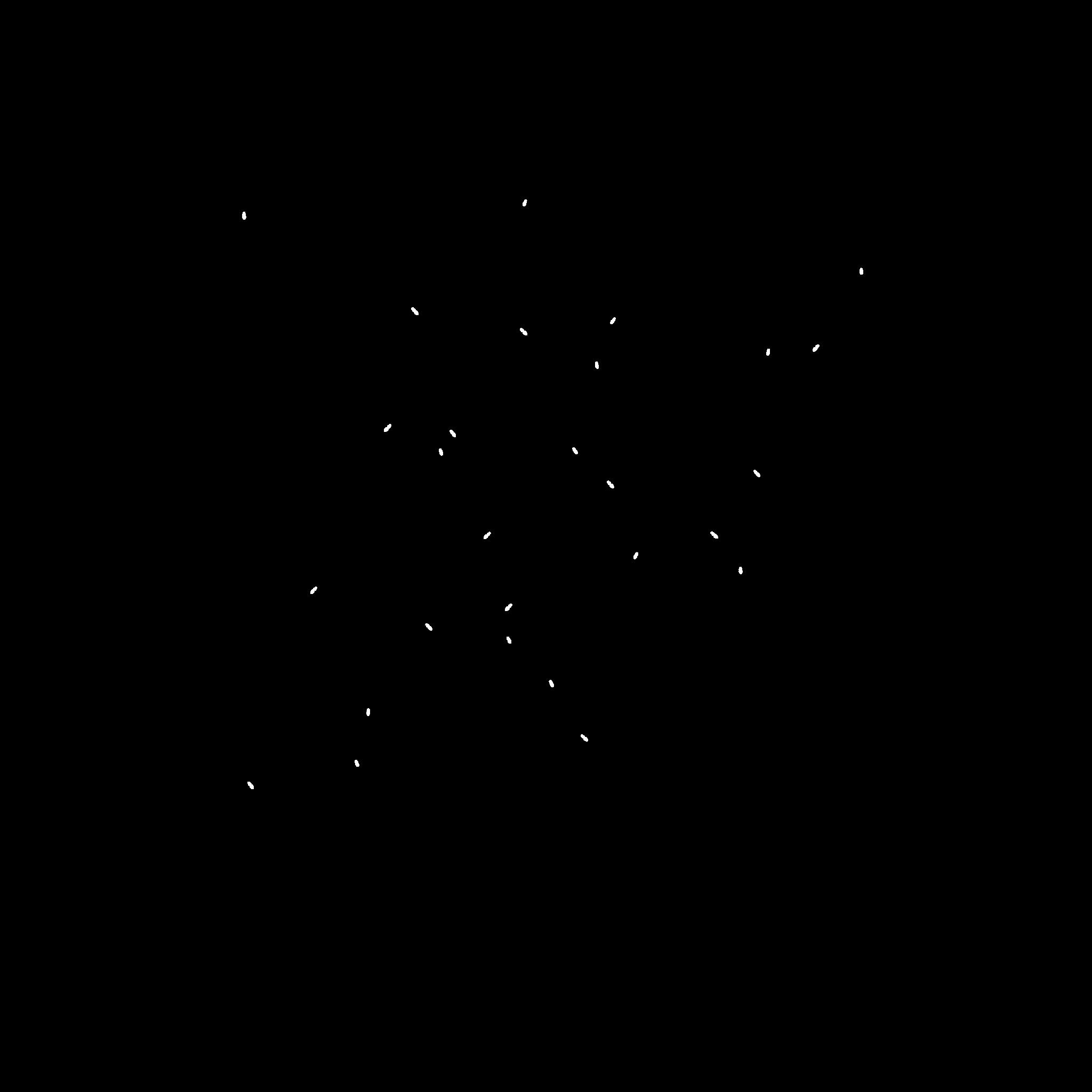

Supplement: S1 File — (ZIP) [file pone.0132101.s003.zip › ORsrc/nonortho/simu028/camx/imx098.jpg]

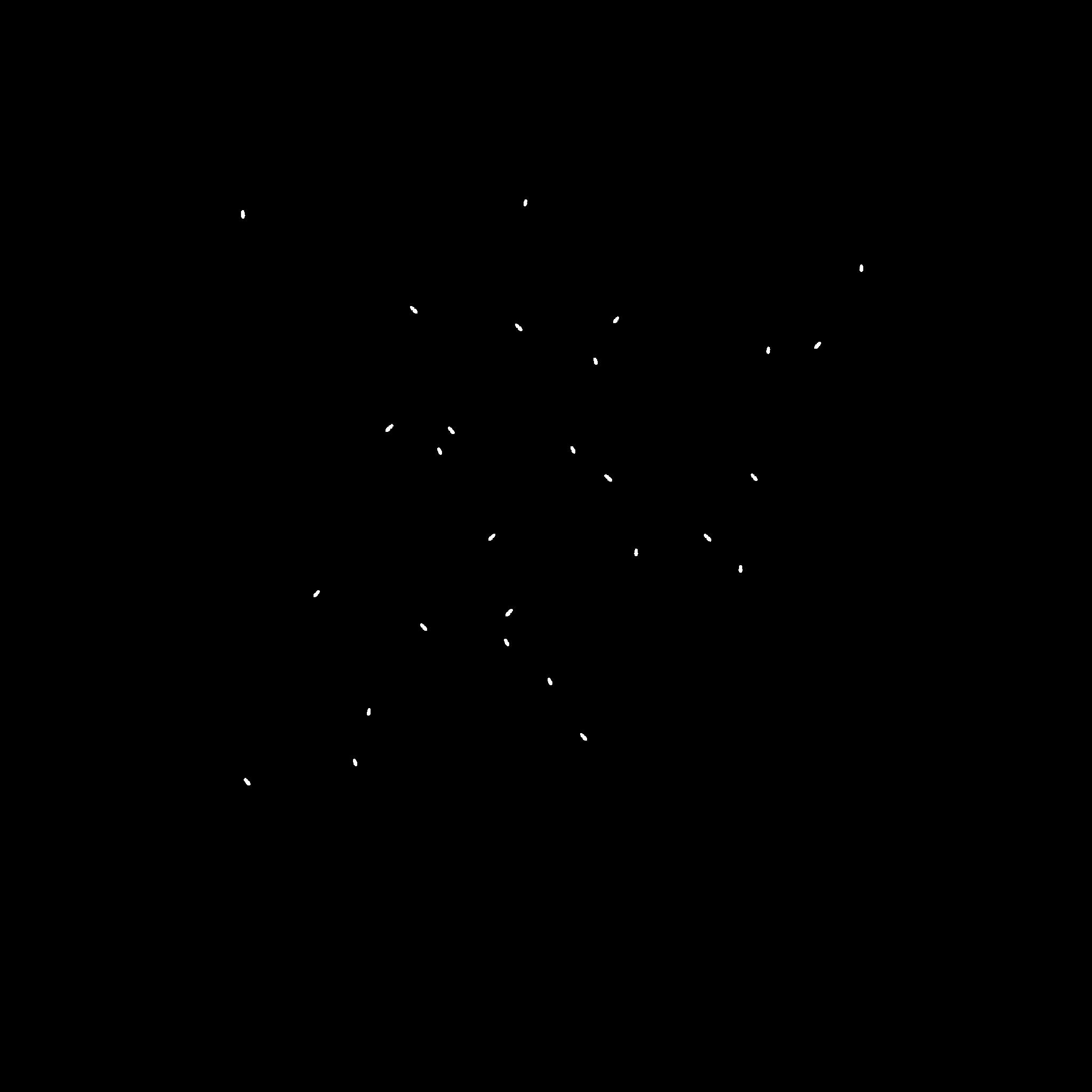

Supplement: S1 File — (ZIP) [file pone.0132101.s003.zip › ORsrc/nonortho/simu028/camx/imx099.jpg]

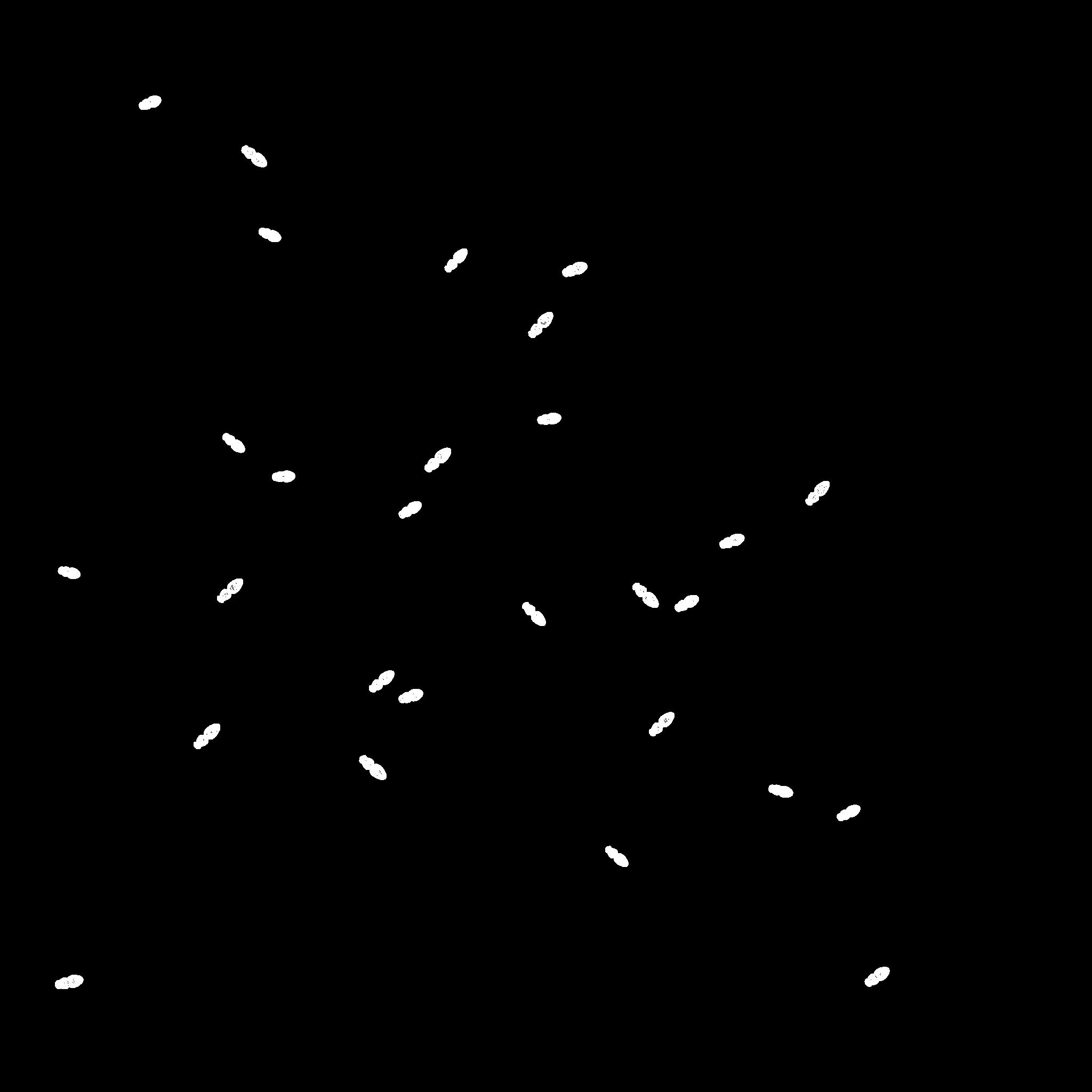

Supplement: S1 File — (ZIP) [file pone.0132101.s003.zip › ORsrc/nonortho/simu028/camx/imx100.jpg]

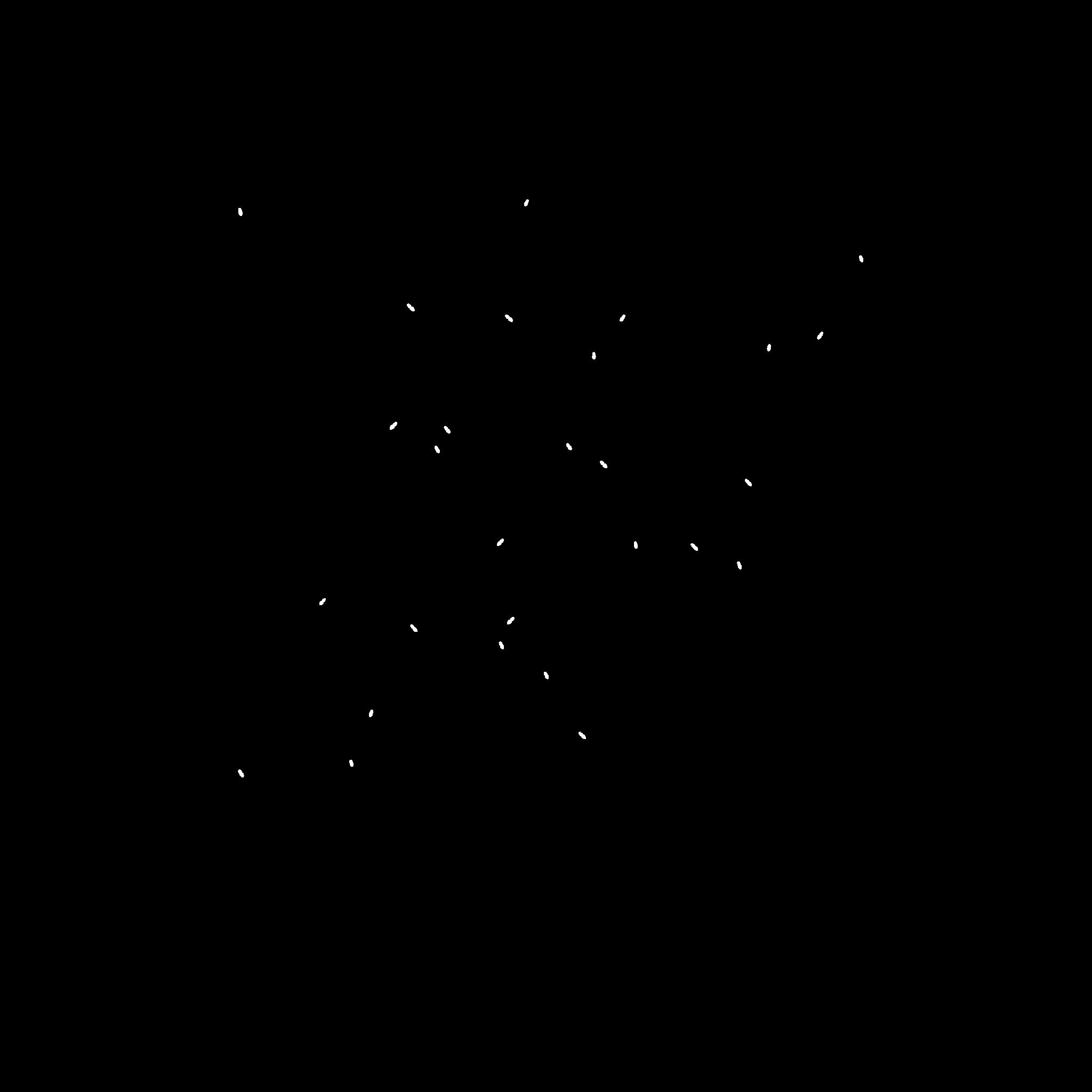

Supplement: S1 File — (ZIP) [file pone.0132101.s003.zip › ORsrc/nonortho/simu028/camx/imx101.jpg]

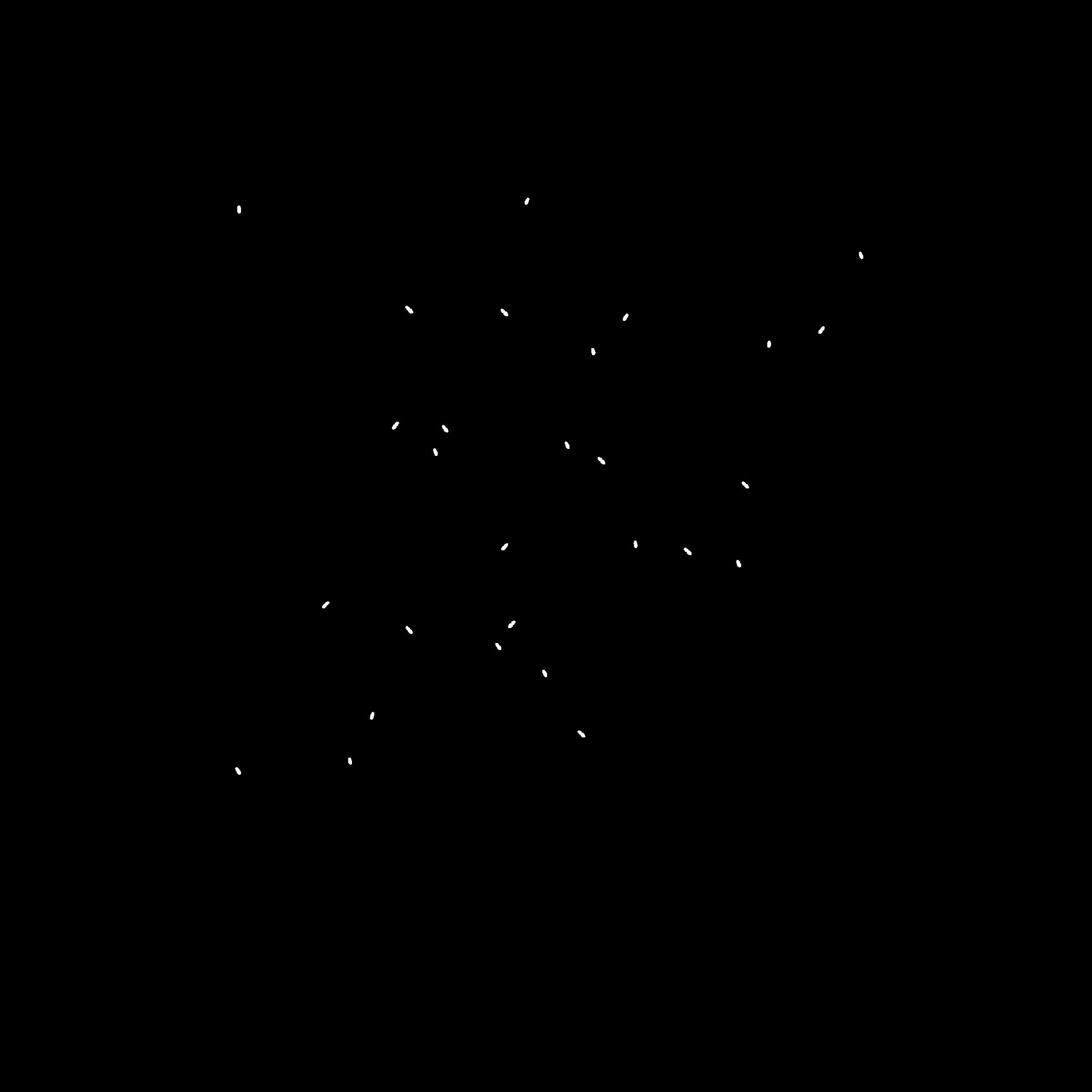

Supplement: S1 File — (ZIP) [file pone.0132101.s003.zip › ORsrc/nonortho/simu028/camx/imx102.jpg]
